# Biaryl Phosphates and Phosphonates as Selective Inhibitors of the Transcription Factor STAT4

**DOI:** 10.1002/anie.202504420

**Published:** 2025-03-27

**Authors:** Nadiya Brovchenko, Angela Berg, Sabine Schubert, Julian Gräb, Theresa Münzel, Christoph Protzel, Kalaiselvi Natarajan, Thorsten Berg

**Affiliations:** ^1^ Institute of Organic Chemistry Leipzig University Johannisallee 29 04103 Leipzig Germany

**Keywords:** Biological activity, Inhibitors, Protein–protein interactions, SH2 domains, Transcription factors

## Abstract

The transcription factor STAT4 has been implicated in the pathogenesis of autoimmune diseases, including inflammatory bowel disease, multiple sclerosis, rheumatoid arthritis, and diabetes mellitus. Here, we report *p*‐biaryl phosphates and phosphonates as the first small‐molecule inhibitors of STAT4. The most potent *p*‐biaryl phosphate inhibited the protein–protein interaction domain of STAT4, the SH2 domain, with submicromolar potency (*K*_i_ = 0.35 µM) and 14‐fold selectivity over the closely related family member STAT3, which has the same core peptide binding motif as STAT4. Further development resulted in the phosphatase‐stable inhibitor Stafori‐1, which protected STAT4 but not STAT3, against thermal denaturation in cell lysates. Its cell‐permeable prodrug Pomstafori‐1 selectively inhibited STAT4 phosphorylation in cultured human cells at low micromolar concentrations. Our data open up the possibility of exploring STAT4 as a target protein for small molecules in the treatment of unmet medical needs.

## Graphical abstract

The transcription factor STAT4 is a therapeutic target for autoimmune diseases. Here, *para*‐biaryl phosphates and phosphonates are presented as the first small‐molecule inhibitors of STAT4. The phosphonate Stafori‐1 selectively stabilizes STAT4 against thermal denaturation in cell lysates, and its cell‐permeable prodrug Pomstafori‐1 selectively inhibits STAT4 activation in cultured human cells at low micromolar concentrations.

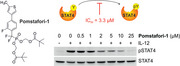

Signal transducers and activators of transcription (STATs) are latent cytoplasmic transcription factors that convey signals from the cell surface to the nucleus upon activation.^[^ ^1^ ^]^ The family member STAT4 is activated by phosphorylation at tyrosine 693 in response to receptor binding by interleukin (IL)‐12.^[^ ^2^ ^]^ Signal transduction via IL‐12/STAT4 leads to the differentiation of T‐helper cells of the Th1 subgroup^[^ ^3^ ^]^ and augments the activation of cytotoxic T cells.^[^ ^4^ ^]^ STAT4 is considered to be a key protein in the development of autoimmune diseases such as multiple sclerosis, rheumatoid arthritis, and type 1 diabetes.^[^ ^5^ ^]^ Despite the immense biological importance of STAT4, small molecules that have been experimentally shown to bind directly to STAT4, and thereby inhibit it, have not been reported. Such molecules could be used to study the role of STAT4 in the development of disease and could potentially serve as lead structures for drug discovery.

STATs contain a protein–protein interaction domain, the SH2 domain, which binds to the cytoplasmic tail of activated cytokine or growth factor receptors, or to nonreceptor tyrosine kinases. The key recognition element of all SH2 domains is phenyl phosphate, in the form of the side chain of an *O*‐phosphorylated tyrosine residue. The conserved nature and similar binding preferences of SH2 domains pose a significant challenge for the development of selective SH2 domain inhibitors.^[^ ^6^ ^]^ However, by decorating phenyl phosphate with additional functional groups, we recently developed catechol bisphosphates^[^ ^7^, ^8^, ^9^, ^10^ ^]^ and *meta*‐terphenyl phosphates^[^ ^11^, ^12^ ^]^ as selective inhibitors of the highly homologous SH2 domains of STAT5b and STAT5a, respectively. Here, we report *para*‐biphenyl phosphate (**1**, Figure 1a) as a selective inhibitor of the SH2 domain of STAT4 (*K*_i_ = 1.1 ± 0.1 µM) in fluorescence polarization (FP) assays (Figure 1b; Tables 1, S1 and S2). Compound **1** displayed 7–8‐fold selectivity over the SH2 domains of STAT1 (*K*_i_ = 8.1 ± 1.4 µM), STAT3 (*K*_i_ = 8.4 ± 1.0 µM), and STAT6 (*K*_i_ = 7.3 ± 0.4 µM). Selectivity of **1** for STAT4 over the SH2 domains of STAT5a (*K*_i_ = 22 ± 2 µM) and STAT5b (*K*_i_ = 47 ± 3 µM) was ∼20‐fold and ∼40‐fold, respectively. The specificity of **1** for STAT4 over STAT3 and STAT1 is surprising given the high sequence similarity in the SH2 domains, which is 76% and 82%, respectively, and the fact that the SH2 domains of STAT4 and STAT3 share the same preferred core binding motif, pYLPQ.^[^ ^13^, ^14^ ^]^ The optimal binding peptide for STAT4, Ac‐GpYLPQNID,^[^ ^15^ ^]^ inhibits STAT4 binding to the corresponding fluorescently labeled peptide (*K*_i_ = 0.22 ± 0.02 µM, Figure S1) to the same extent as it inhibits STAT3 binding to its preferred binding peptide (*K*_i_ = 0.24 ± 0.04 µM, Figure S1), and only slightly more than it inhibits binding of the optimal peptide to STAT1 (*K*_i_ = 0.36 ± 0.08 µM, Figure S1).

**Figure 1:**
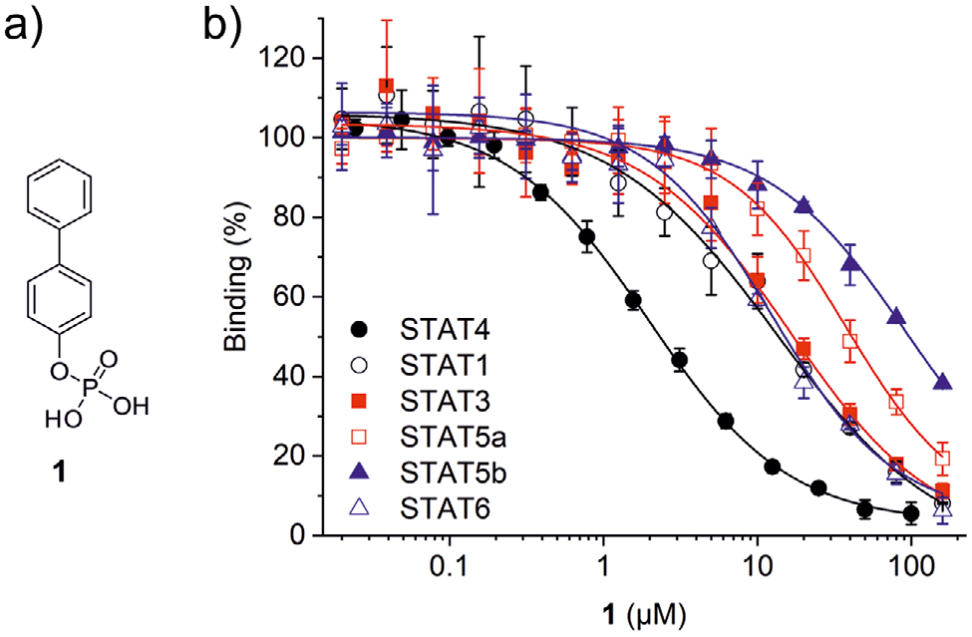
a) Structure of *para*‐biphenyl phosphate (**1**) and b) its activity profile in FP assays against STAT proteins. Error bars represent standard deviations (*n* = 3).

**Table 1:** Structures of *p*‐biaryl phosphates and their activity against STAT4 in FP assays. Mean values ± standard deviations are given (*n* = 3). *K*_i_ values were derived from *IC*_50_ values as described in the Supporting Information.^[^ ^16^ ^]^

No	Structure	STAT4 *K*_i_ (µM)	No	Structure	STAT4 *K*_i_ (µM)
**1**	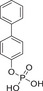	1.1 ± 0.1	**6h**	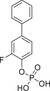	0.56 ± 0.08
**6a**	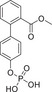	9.1 ± 0.8	**6i**	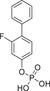	3.8 ± 0.1
**6b**	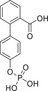	7.8 ± 0.5	**6j**	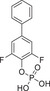	2.0 ± 0.1
**6c**	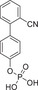	2.7 ± 0.3	**6k**	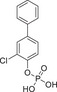	3.3 ± 1.0
**6d**	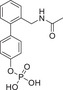	6.2 ± 0.4	**6l**	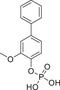	22 ± 2
**6e**	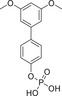	1.9 ± 0.1	**6m**	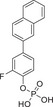	0.35 ± 0.01
**6f**	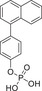	0.84 ± 0.08	**4m**	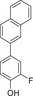	no inhibition at 200 µM
**6g**	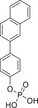	0.44 ± 0.09			

We synthesized a series of derivatives of **1** in order to understand structure–activity relationships and to identify inhibitors with improved potencies. Derivatives bearing variations on either aromatic ring were synthesized by Suzuki coupling between phenol boronic acids and aryl halides (compound types **2** and **3**, see Supporting Information), providing the biphenyl derivatives **4c**–**m** (Scheme 1a). Atherton‐Todd phosphorylation of **4a**–**m** provided the dibenzylesters **5a**–**m** (Scheme 1b), from which the phosphoric acid monoesters **6a**–**m** were obtained by catalytic hydrogenation or trimethylsilylbromide‐mediated cleavage.

**Scheme 1:**
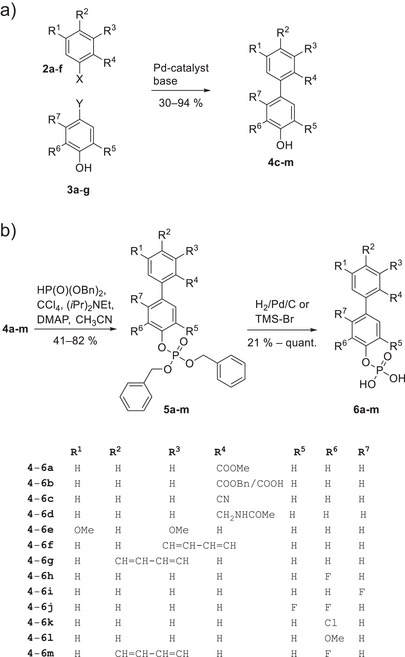
a) Synthesis of biaryl precursors **4c**–**m**. Details of the Suzuki couplings are provided in the Supporting Information. Compounds **4a** and **4b** were obtained from commercially available 4′‐hydroxybiphenyl‐2‐carboxylic acid via esterification. b) Atherton‐Todd phosphorylation and debenzylation provide biaryl phosphates **6a**–**m**.

Compounds **6a**–**e**, bearing substituents in the *ortho*‐ or *meta*‐position of the upper ring, did not show improved activity against STAT4 (Tables 1 and S1). In contrast, replacing the upper phenyl ring for a 1‐naphthyl moiety (**6f**, *K*_i_ = 0.84 ± 0.08 µM), and especially for a 2‐naphthyl moiety (**6g**, *K*_i_ = 0.44 ± 0.09 µM), resulted in improved activity and selectivity as compared to **1** (Tables S1 and S2). Structural variations of the lower ring of **1** showed that a fluorine substituent in the 2‐position (**6h**, *K*_i_ = 0.56 ± 0.08 µM, Tables 1 and S1) improved both the activity and selectivity as compared to **1** (Tables 1, S1 and S2). In contrast, a fluorine substituent in the 3‐position (**6i**, *K*_i_ = 3.8 ± 0.1 µM) decreased the activity, as did the placement of two fluorine atoms in both the 2‐ and the 5‐position (**6j**, *K*_i_ = 2.0 ± 0.1 µM, Tables 1 and S1). A chlorine atom in the 2‐position was better tolerated (**6k**, *K*_i_ = 3.3 ± 1.0 µM) than a methoxy group (**6l**, *K*_i_ = 22 ± 2 µM).

We then combined the best potency‐enhancing alterations for either ring system. The resulting compound **6m** was the most potent STAT4 inhibitor (*K*_i_ = 0.35 ± 0.01 µM), displaying ∼10–70‐fold selectivity over other STAT proteins (Figure 2a; Tables 1, S1 and S2).

**Figure 2:**
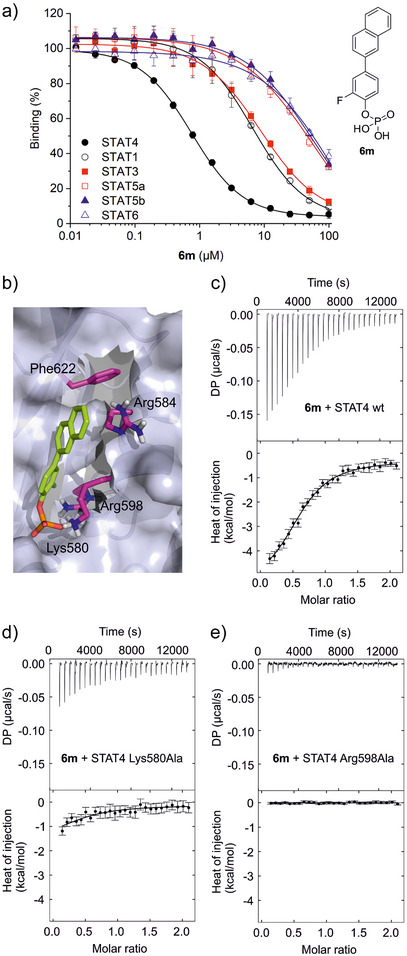
a) Structure and activity profile of **6m** in FP assays against STAT proteins. Error bars represent standard deviations (*n* = 3). b) Docking of **6m** into the AlphaFold2^[^ ^17^, ^18^ ^]^ model of the STAT4 SH2 domain using AutoDock FR.^[^ ^19^ ^]^ The side chains of Lys580, Arg584, Arg598, and Phe622 were defined as flexible. The figure was generated using PyMOL.^[^ ^20^ ^]^ c)–e) Representative ITC data for binding between **6m** and c) wild‐type STAT4, d) STAT4 Lys580Ala, and e) STAT4 Arg598Ala (*n* = 4 for each). Error bars in c)–e) represent integration errors assigned by the data analysis software NITPIC for the depicted individual experiments.^[^ ^21^ ^]^

The key interactions of phosphotyrosine‐containing peptides with STAT SH2 domains are electrostatic interactions between the phosphate group of phosphotyrosine and a conserved arginine and lysine residue pair in close proximity to each other in the SH2 domain.^[^ ^22^ ^]^ In light of the structural similarity of the *p*‐biaryl phosphates to the side chain of phosphorylated tyrosine, we postulated that the phosphate group of the *p*‐biaryl phosphates occupies the phosphotyrosine binding pocket of the STAT4 SH2 domain, with Lys580 and Arg598 as the key interaction partners. Molecular docking of **6m** into the AlphaFold2^[^ ^17^, ^18^ ^]^ model of the STAT4 SH2 domain using AutoDock FR^[^ ^19^ ^]^ was consistent with a key role of Lys580 and Arg598 in binding of the phosphate group and suggested possible polar interactions between the *ortho*‐fluorine substituent of **6m** and STAT4 Arg598 (Figure 2b). In order to validate the role of Lys580 and Arg598 experimentally, we investigated binding of **6m** to wild‐type STAT4 and to the point mutants Lys580Ala and Arg598Ala by isothermal titration calorimetry (ITC). Titration of **6m** into wild‐type STAT4 confirmed binding with a low micromolar affinity (*K*_d_ = 1.3 ± 0.3 µM, Figure 2c). In contrast, titration of **6m** into the STAT4 point mutant Lys580Ala (Figure 2d) demonstrated markedly decreased protein affinity, which was too weak for reliable quantification. Titration of **6m** into the STAT4 point mutant Arg598Ala showed no heat release (Figure 2e), similar to the control titration of **6m** into buffer (Figure S2a). This is consistent with the notion that **6m** occupies the phosphotyrosine binding pocket of the STAT4 SH2 domain and with the lack of activity of the unphosphorylated precursor molecule **4m** (Table 1). Molecular docking suggested the naphthyl group of **6m** to be located in a hydrophobic pocket (Figure 2b), stabilized by aryl–CH_2_ interactions between the phenyl ring of Phe622 in the βD‐sheet and the aliphatic part of the side chain of Arg584 in the αA‐helix of the STAT4 SH2 domain, with the guanidinium part of the Arg584 side chain pointing towards the solvent. The relevance of Phe622 for protein folding was demonstrated by the instability of the STAT4 Phe622Ala mutant, which prevented experimental validation of the binding pose. Unfortunately, extensive efforts to obtain a co‐crystal structure of STAT4 in complex with an inhibitor remained unsuccessful (Tables S3–S6).

Since STATs are intracellular proteins and phenyl phosphates can be cleaved by intracellular phosphatases, we carried out further inhibitor improvement with the synthesis of phosphatase‐stable phosphonates.^[^ ^23^ ^]^ While the benzyl phosphonate **7** (Table 2; Scheme S1) based on **6m** displayed poor activity against STAT4 (33 ± 4% inhibition at 100 µM, the highest concentration tested), the corresponding α,α‐difluorobenzyl phosphonate **8a** (Table 2; Scheme S2) retained selective activity against STAT4 (*K*_i_ = 4.0 ± 0.5 µM, Tables 2, S7 and S8).

**Table 2:** Structures of *p*‐biaryl phosphonates and their activity against STAT4 in FP assays. Mean values ± standard deviations are given (*n* = 3). *K*_i_ values were derived from *IC*_50_ values as described in the Supporting Information.^[^ ^16^ ^]^

No	Structure	STAT4 *K*_i_ (µM) or inhibition (%)	No	Structure	STAT4 *K*_i_ (µM)
**7**	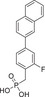	33 ± 4% inhibition at 100 µM	**8g**	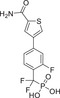	11 ± 2
**8a**	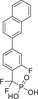	4.0 ± 0.5	**8h**	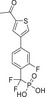	7.1 ± 0.6
**8b**	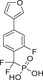	39 ± 1	**8i**	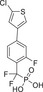	5.5 ± 0.7
**8c**	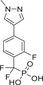	8.0 ± 0.7	**8j**	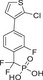	7.3 ± 0.6
**8d**	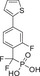	3.6 ± 0.2	**8k**	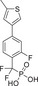	1.7 ± 0.2
**8e**	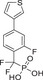	2.8 ± 0.3	**8l**	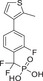	8.7 ± 0.9
**8f**	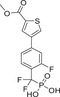	4.4 ± 0.1	**8m**	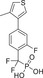	8.9 ± 0.7

In the search for a replacement for the hydrophobic naphthyl group with a lower molecular weight, we considered five‐membered aromatic rings. Furyl (**8b**, *K*_i_ = 39 ± 1 µM) or *N*‐methylpyrazolyl (**8c**, *K*_i_ = 8.0 ± 0.7 µM) substituents were not helpful, but compounds bearing a 2‐thienyl (**8d**, *K*_i_ = 3.6 ± 0.2 µM) and, especially, a 3‐thienyl substituent (**8e**, *K*_i_ = 2.8 ± 0.3 µM) displayed higher activities than **8a** (Table 2, Scheme S2). A variety of substituents on the 3‐thienyl group were explored (**8f**–**m**). While polar substituents in the 4‐position (**8f**–**h**) or a chlorine in the 4‐position (**8i**, *K*_i_ = 5.5 ± 0.7 µM) or the 2‐position (**8j**, *K*_i_ = 7.3 ± 0.6 µM) did not increase the activity, a methyl group (**8k**, *K*_i_ = 1.7 ± 0.2 µM, ligand efficiency: 0.39 kcal mol^−1^) in the 4‐position of the thienyl substituent improved the activity as compared to **8e**. This was a specific effect, since placement of a methyl group in the 2‐position (**8l**, *K*_i_ = 8.7 ± 0.9 µM) or 5‐position (**8m**, *K*_i_ = 8.9 ± 0.7 µM) reduced activity compared to **8e**. The dissociation constant of **8k** against STAT4 as determined by ITC (*K*_d_ = 7.1 ± 1.1 µM, Figures 3a and S2b) is 2‐fold higher than the *IC*_50_ value in FP assays (*IC*_50_ = 3.7 ± 0.4 µM, Table S7). A similar activity ratio between ITC and FP assays was observed for the phosphate **6m** (ITC: *K*_d_ = 1.3 ± 0.3 µM, Figure 2c; FP: *IC*_50_ = 0.76 ± 0.01 µM, Table S1). **8k** displayed good selectivity for STAT4 in FP assays (Figure 3b, Table S8).

**Figure 3:**
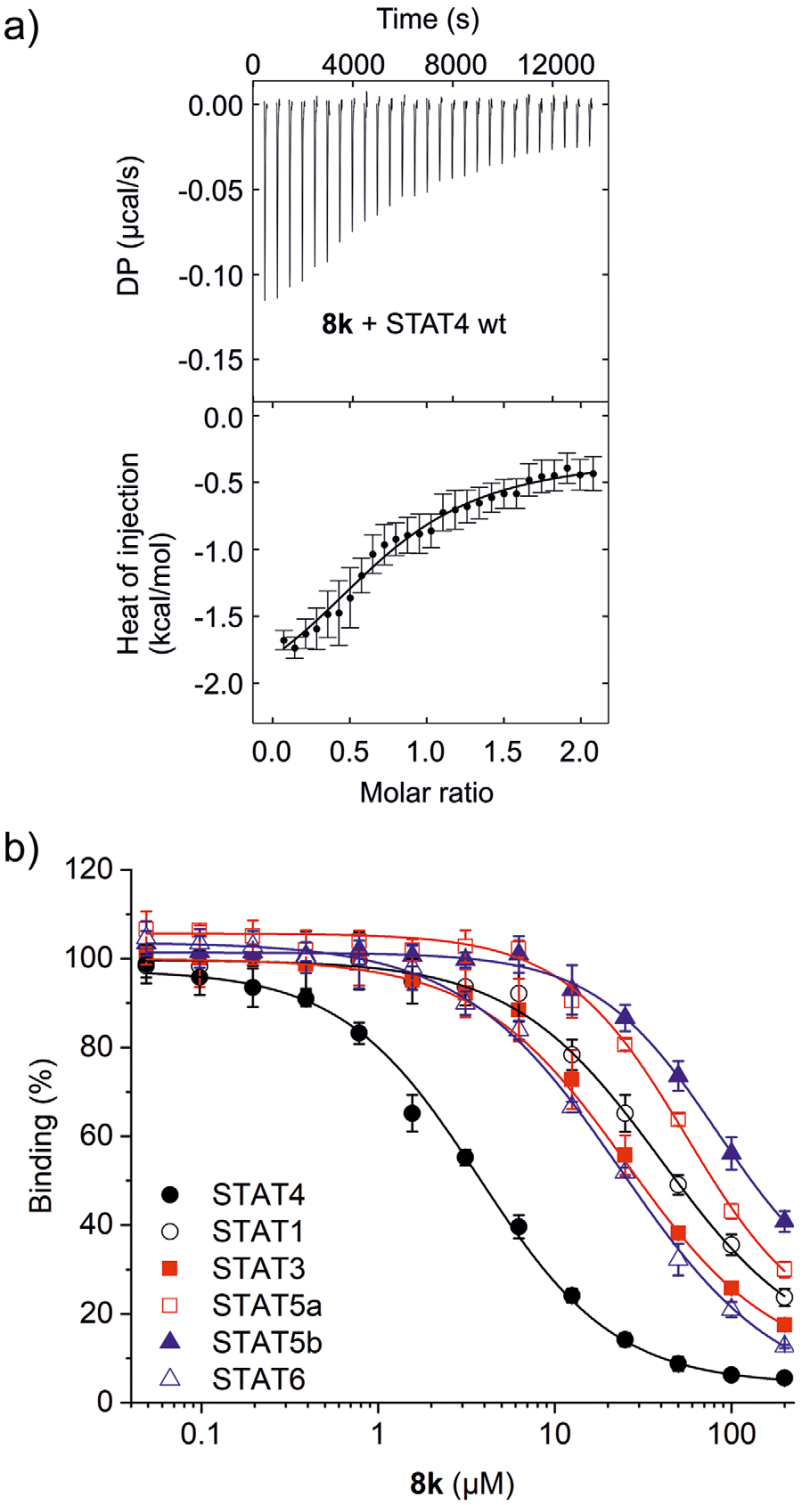
a) Representative ITC data for binding between **8k** and wild‐type STAT4 (*n* = 3). Error bars represent integration errors assigned by the data analysis software NITPIC for the depicted individual experiment.^[^ ^21^ ^]^ b) Activity profile of **8k** in FP assays against STAT proteins. Error bars represent standard deviations (*n* = 3).

The recently developed cellular thermal shift assay (CETSA)^[^ ^24^ ^]^ has been adapted for use with the STAT1, STAT3, and STAT5a proteins.^[^ ^25^ ^]^ We present here a new application of CETSA for the analysis of STAT4 protein stabilization, utilizing cellular lysates from the STAT4‐expressing natural killer cell line NK‐92. We used this assay to test the effects of the α,α‐difluorobenzyl phosphonates **8a** and **8k** on STAT4 in the intracellular milieu. Compared to the DMSO control (*T*_agg_ = 48.7 ± 0.5 °C, Figure 4a,f and Table S9), **8a** (100 µM) increased the aggregation temperature of STAT4 (*T*_agg_ = 49.8 ± 0.1 °C, Δ*T*_agg_ = 1.1 °C, *p* = 0.023, Figure 4b,f and Table S9) to a statistically significant extent. A slightly stronger effect was observed for the more potent compound **8k** (100 µM, *T*_agg_ = 50.0 ± 0.5 °C, Δ*T*_agg_ = 1.3 °C, *p* = 0.031, Figure 4c,f and Table S9), indicating target engagement of both **8a** and **8k** in cell lysates. The shift in aggregation temperature caused by **8a** and **8k** is higher than that of the high‐affinity STAT4‐binding phosphopeptide Ac‐GpYLPQNID at the same concentration (100 µM, *T*_agg_ = 49.4 ± 0.7 °C, Δ*T*_agg_ = 0.7 °C, *p* = 0.234, Figure 4d,f and Table S9). Only at a higher concentration of 500 µM is the shift conferred by Ac‐GpYLPQNID more distinct and statistically significant (*T*_agg_ = 51.8 ± 0.7 °C, Δ*T*_agg_ = 3.1 °C, *p* = 0.003, Figure 4e,f and Table S9). The relatively small changes in the aggregation temperature of STAT4 observed at a concentration of 100 µM SH2 domain ligand are consistent with the higher concentrations of positive control peptides (500 µM) used for establishing the validity of CETSA in cell lysates for STAT1, STAT3, and STAT5a as reported in the literature.^[^ ^25^ ^]^ This probably reflects the fact that the SH2 domain is only a minor part of the soluble STAT protein constructs used in this study and throughout the literature. This limits the extent to which ligand binding can counteract the thermally induced protein motions that lead to misfolding and subsequent aggregation. **8k** did not protect the closely related family member STAT3 from thermal degradation, with no significant alteration to the aggregation temperature (*T*_agg_ = 50.6 ± 0.2 °C, Δ*T*_agg_ = −0.4 °C, *p* = 0.07, Figure S3), as compared to the DMSO control (*T*_agg_ = 51.0 ± 0.3 °C, Figure S3), demonstrating inhibitor specificity in the context of the intracellular milieu.

**Figure 4:**
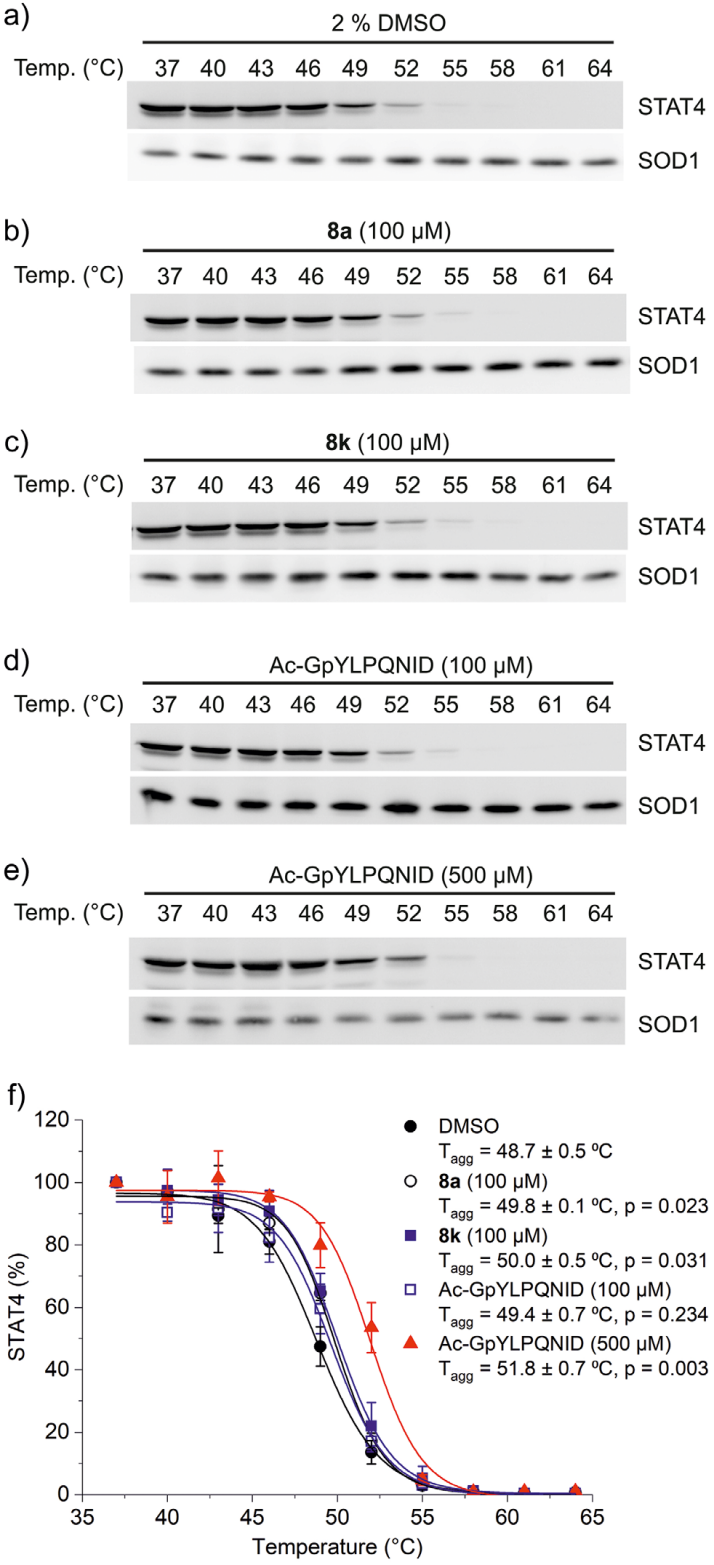
Thermal stability of STAT4 in lysates from NK‐92 cells in the presence of a) DMSO, b) **8a** (100 µM), c) **8k** (100 µM), as well as the peptide Ac‐GpYLPQNID at d) 100 µM and e) 500 µM. f) Quantitation of the STAT4 band intensities for repeated experiments as shown in a)–e) (*n* = 3 in each case). Error bars represent standard deviations. *p*‐values refer to Student's *t*‐test, two‐tailed, two‐sample equal variance. Uncropped blots are shown in Figure S7 in the Supporting Information.

STAT4 is phosphorylated on Tyr693 by activation of the interleukin (IL)‐12 receptor (Figure 5a).^[^ ^2^ ^]^ This process can be inhibited by ligands of the STAT4 SH2 domain. However, phosphonates are typically poorly cell permeable, owing to their negative charges at physiological pH. Not surprisingly, **8k** was unable to inhibit IL‐12‐induced STAT4 Tyr693 phosphorylation in NK‐92 cells (Figure S4). We therefore converted phosphonate **8k** to its pivaloyloxymethyl (POM) ester **9**, from which **8k** is liberated inside cells (Figure 5b). As a negative control, we also synthesized the POM ester **10** of the least active phosphonate **7**. Prodrug **9** inhibited IL‐12‐induced STAT4 phosphorylation with moderate activity (*IC*_50_ = 77 µM, Figure S5), while the negative control **10** was inactive. These data were obtained in the presence of 25% serum, a relatively high concentration required for culturing NK‐92 cells. In the absence of serum, the effect of **9** on STAT4 Tyr693 phosphorylation in NK‐92 cells was much stronger (*IC*_50_ = 3.3 µM, Figure 5c,d), suggesting that **9** can be inactivated by binding to serum proteins. The control compound **10** was still inactive under these conditions (Figure 5c,d). Phosphorylation of STAT3 in HCC‐827 cells under serum‐free conditions was inhibited to a lesser extent by **9** (*IC*_50_ = 19 µM, Figure 5e,f), and STAT5a/5b‐phosphorylation in K562 cells to a lesser extent still (*IC*_50_ > 25 µM, Figure 5g,h). Hence, the activity and the specificity profile of **9** observed in cellular STAT4 activation assays corresponded to those of the precursor **8k** in FP assays.

**Figure 5:**
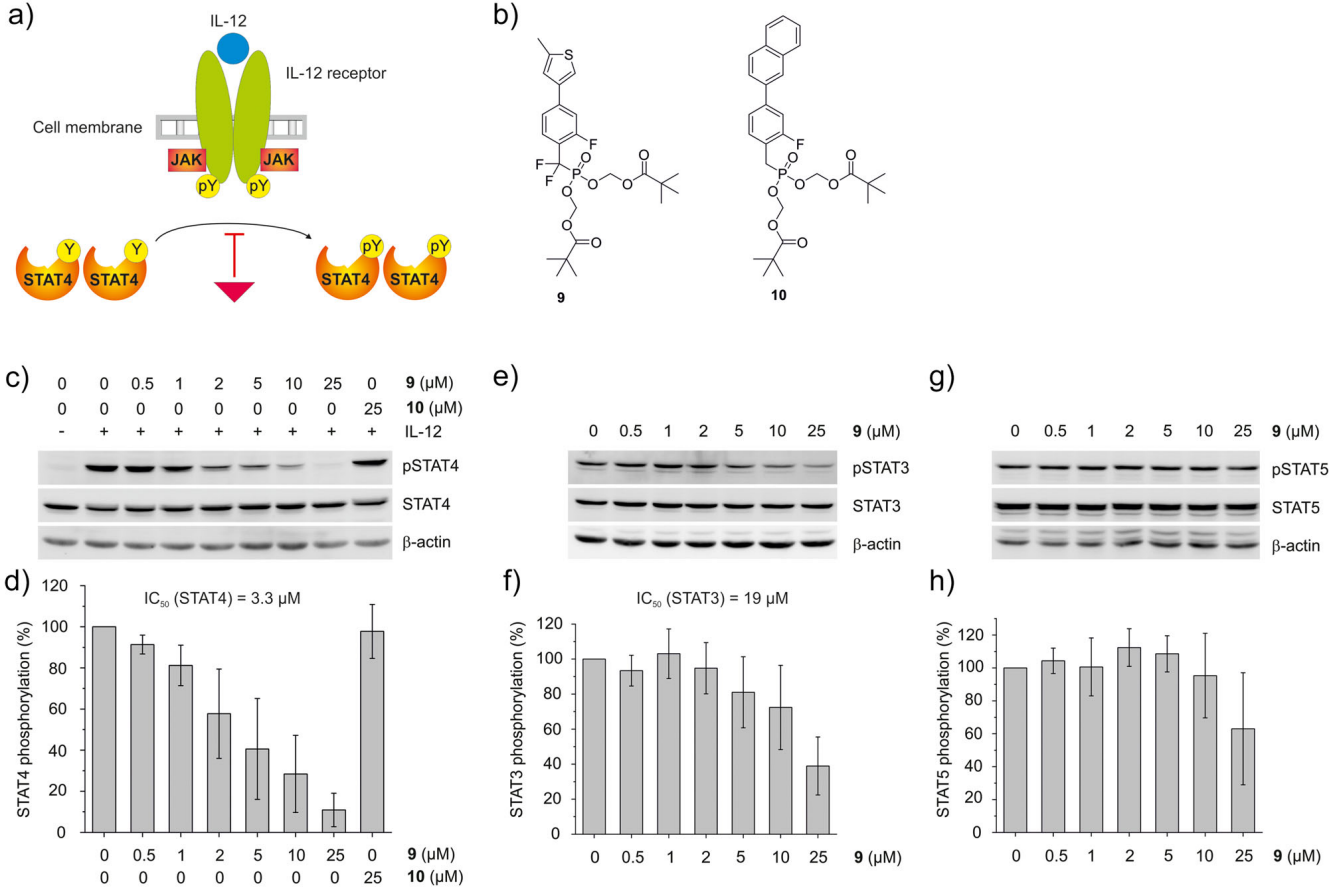
a) Induction of STAT4 Tyr693 phosphorylation upon activation of the IL‐12 receptor. The graphic is modified from the literature.^[^ ^26^ ^]^ b) Structure of prodrugs **9** and **10**. c) Inhibition of IL‐12‐induced STAT4 Tyr693 phosphorylation in NK‐92 cells by **9** but not **10**. d) Quantitation of band intensity from repeat experiments (**9**, *n* = 4; **10**, *n* = 2). Effect of **9** on e) STAT3 Tyr705 phosphorylation in HCC‐827 cells with f) quantitation of band intensity from repeat experiments (*n* = 3), and g) STAT5 phosphorylation of Tyr694 (STAT5a)/Tyr699 (STAT5b) in K562 cells with h) quantitation of band intensity from repeat experiments (*n* = 3). Error bars represent standard deviations. Uncropped blots are shown in Figure S8 in the Supporting Information.

Treatment of natural killer cells with IL‐12 and subsequent activation of STAT4 by Tyr693 phosphorylation is a prerequisite for the induction of interferon (IFN)‐γ.^[^ ^27^ ^]^ While stimulation with IL‐18 alone does not induce binding of STAT4 to the IFN‐γ promoter, co‐stimulation with both IL‐12 and IL‐18 leads to a substantially stronger IFN‐γ induction than stimulation with IL‐12 alone.^[^ ^28^ ^]^ Consistent with this, significant induction of IFN‐γ in NK‐92 cells required co‐stimulation with both IL‐12 and IL‐18 (Figure 6). IL12/IL‐18‐mediated IFN‐γ induction was strongly reduced in the presence of **9**, as was STAT4 phosphorylation.

**Figure 6:**
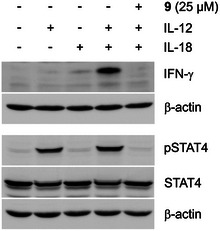
Prodrug **9** inhibits IL‐12/IL‐18‐mediated STAT4 phosphorylation and IFN‐γ induction in NK‐92 cells. Uncropped blots are shown in Figure S9 in the Supporting Information.

IL‐12 stimulation of TALL‐104 cells, derived from human T lymphoblasts, also induces phosphorylation of STAT4, but whether this leads to induction of IFN‐γ in this cell line has not been reported.^[^ ^29^ ^]^ Our experiments demonstrate that stimulation of TALL‐104 cells with IL‐12 is sufficient to observe induction of IFN‐γ after 4 hours, but that substantially higher IFN‐γ levels are induced in the presence of additional IL‐18 (Figure S6). STAT4 phosphorylation stimulated by IL‐12 alone or co‐stimulated by IL‐12 and IL‐18 was inhibited by **9**, as was IFN‐γ induction. The combined data from NK‐92 (Figure 6) and TALL‐104 cells (Figure S6) validate **9** as a useful tool compound for studying the relevance of STAT4 for IL‐12/IL‐18‐mediated signaling.

In summary, we present *para*‐biaryl phosphates and phosphonates as the first small‐molecule inhibitors of STAT4. The phosphonate inhibitor **8k**, which was dubbed Stafori‐1 (STAT four inhibitor‐1), selectively stabilized STAT4 against thermal denaturation in cell lysates. Its cell‐permeable prodrug **9,** dubbed Pomstafori‐1, selectively inhibited STAT4 activation and IFN‐γ induction in cultured human cells. Our data demonstrate that small‐molecule inhibitors of STAT SH2 domains can differentiate between highly homologous STAT family members that share a high‐affinity core peptide binding motif and provide the proof‐of‐principle that it is possible to directly and selectively target the STAT4 SH2 domain with a small molecule. On a wider note, we believe that our findings will have implications for the scientific community's perception of the druggability of SH2 domains, which remain an important class of cellular signal transduction modules for which no drugs are as yet available in the clinic.

## Supporting Information

The authors have cited additional references within the Supporting Information.^[^ ^30^, ^31^, ^32^, ^33^, ^34^, ^35^, ^36^, ^37^, ^38^, ^39^, ^40^, ^41^ ^]^

## Conflict of Interests

The authors declare no conflict of interest.

## Supporting information



Supporting Information

## Data Availability

The data that support the findings of this study are available in the supplementary material of this article.
