# Supplementary material for: Biaryl Phosphates and Phosphonates as Selective Inhibitors of the Transcription Factor STAT4
Source: Angew Chem Int Ed Engl. 2025 Mar 27;64(21):e202504420. doi: 10.1002/anie.202504420 (PMC12087874; doi:10.1002/anie.202504420)
Supplement: Supplementary file 1 — Supporting Information [file ANIE-64-e202504420-s001.pdf]

Supporting Information

Biaryl Phosphates and Phosphonates as Selective inhibitors of the Transcription factor STAT4

Nadiya Brovchenko,[a]# Angela Berg,[a]# Sabine Schubert,[a] Julian Gräb,[a] Theresa Münzel,[a] Christoph Protzel,[a] Kalaiselvi Natarajan,[a] and Thorsten Berg*[a]

Table of Contents

Figure S1 3
Figure S2 3
Figure S3 4
Figure S4 5
Figure S5 5
Figure S6 6
Figure S7 7
Figure S8 8
Figure S9 9
Figure S10 10
Figure S11 11
Figure S12 12
Table S1 13
Table S2 14
Table S3 15
Table S4 15
Table S5 15
Table S6 16
Table S7 17
Table S8 18
Table S9 18
Scheme S1 18
Scheme S2 19
Methods 19
Fluorescence polarization assays 19
Isothermal Titration Calorimetry 20

Cell culture experiments 21
Lysates for CETSA 21
CETSA 21
Western blotting 22
General synthetic methods 23
Synthesis and spectroscopic characterization of compounds 25
NMR spectra 84
Supporting References 139

Figure S1

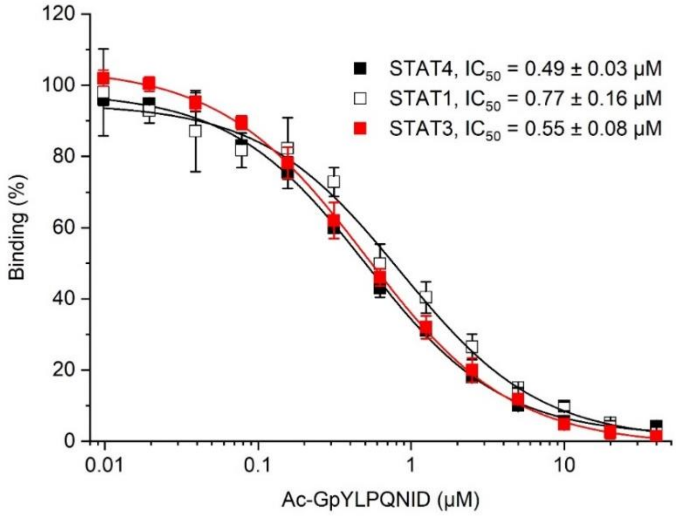

Figure S1: Inhibitory effect of the peptide Ac-GpYLPQNID against STAT4, STAT1 and STAT3 peptide binding in fluorescence polarization assays. The following fluorescent peptides were used as tracers: 5-carboxyfluorescein-GpYLPQNID-OH for STAT4; 5-carboxyfluorescein-GpYDKPHVL for STAT1, and 5-carboxyfluorescein-GpYLPQTV-NH_2 for STAT3. Error bars represent standard deviations (n = 3).

Figure S2

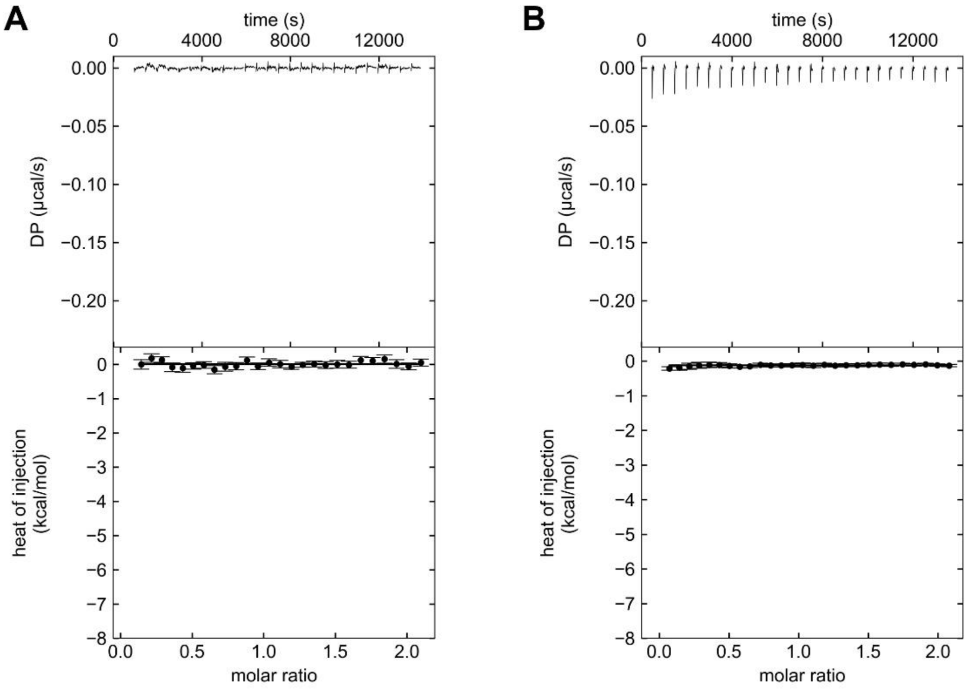

Figure S2: A) Titration of 100 µM **6m** dissolved in ITC buffer to a final DMSO concentration of 0.1% (v/v) into ITC buffer supplemented with 0.1% (v/v) DMSO. B) Titration of 100 µM **8k** dissolved in ITC buffer to a final DMSO concentration of 2 % (v/v) into ITC buffer supplemented with 2 % (v/v) DMSO. Error bars represent integration errors assigned by the data analysis software NITPIC for the depicted individual experiments.[1]

Figure S3

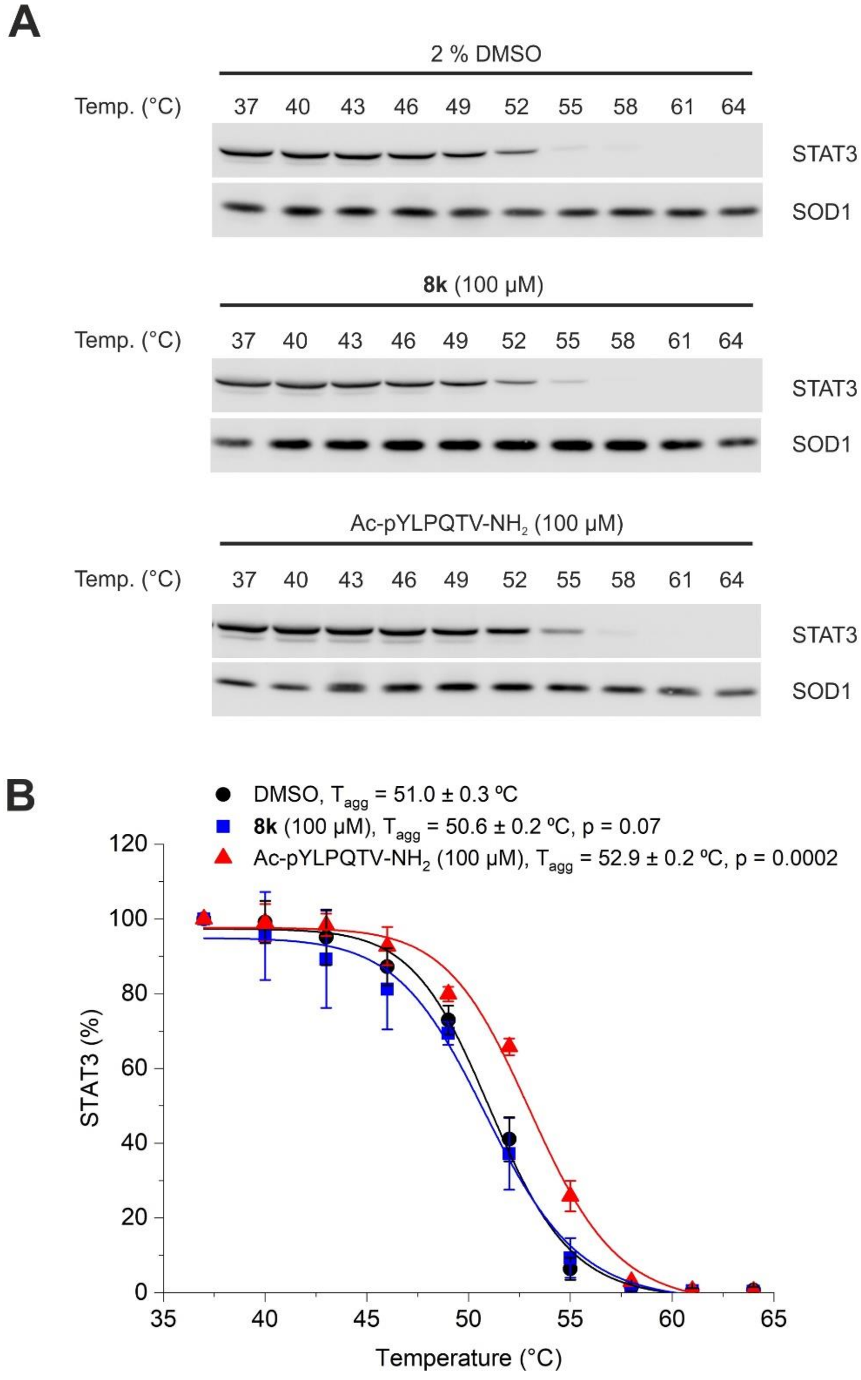

Figure S3: A) **8k** does not stabilize STAT3 against thermal degradation in lysates from NK-92 cells, while Ac-pYLPQTV-NH_2 has a stabilizing effect at 100 µM. B) Quantitation of the STAT3 band intensities for each of the blots in A) (n = 3). Error bars represent standard deviations. p-values refer to Student's t-test, two-tailed, two-sample equal variance. Uncropped blots are shown in Figure S10.

Figure S4

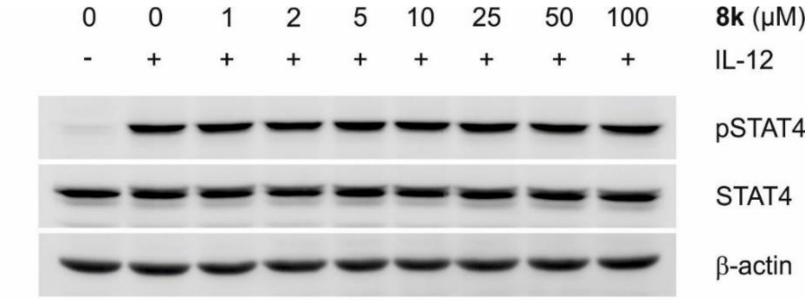

Figure S4: Treatment of NK-92 cells with **8k** for 2 h does not inhibit IL-12-induced STAT4 Tyr693 phosphorylation. Uncropped blots are shown in Figure S11.

Figure S5

A

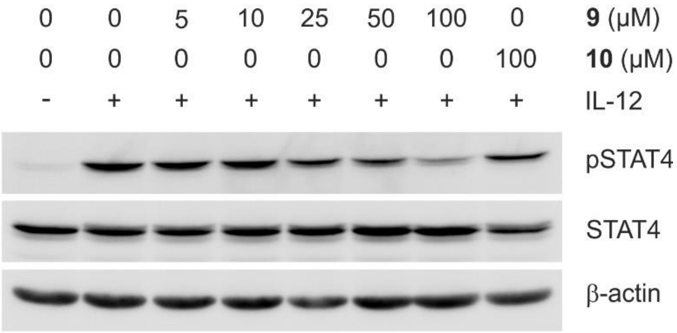

B

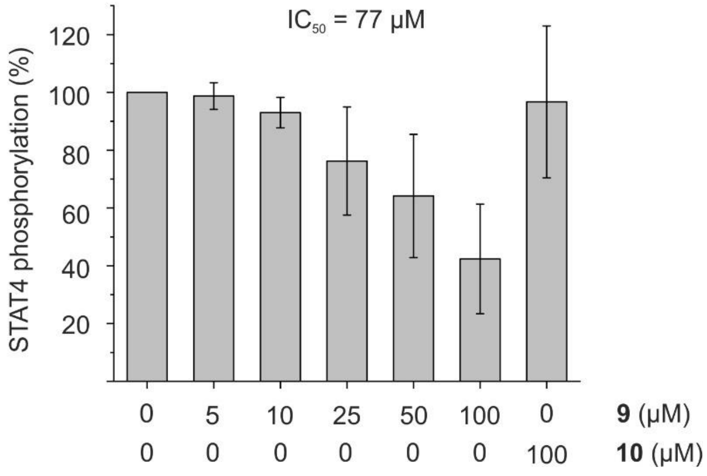

Figure S5: Treatment of NK-92 cells with **9** for 2 h in the presence of 25% serum moderately inhibits IL-12-induced STAT4 Tyr693 phosphorylation, while treatment with **10** does not. Error bars represent standard deviations (n=3). Uncropped blots are shown in Figure S11.

Figure S6

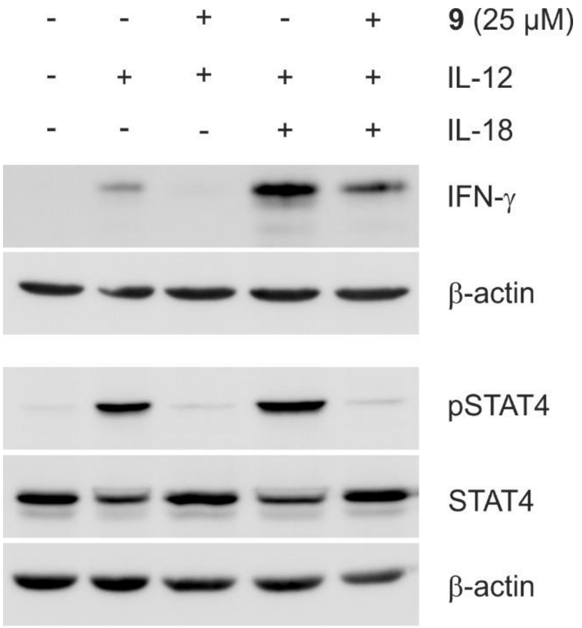

Figure S6. **9** inhibits IL-12- and IL-12/IL-18-mediated STAT4 phosphorylation and IFN-γ induction in TALL-104 cells. Uncropped blots are shown in Figure S12.

Figure S7

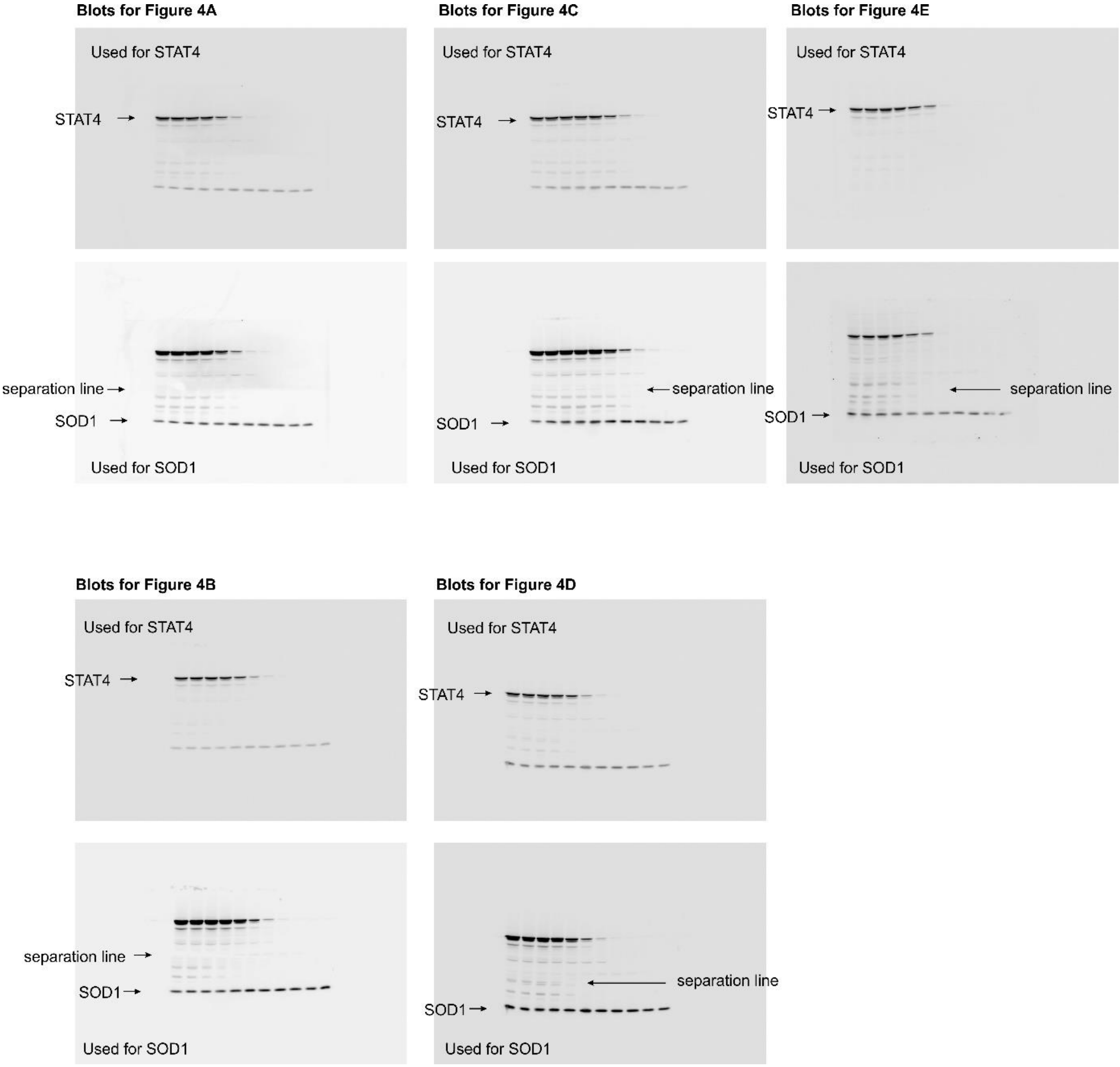

Figure S7: Uncropped blots used for generation of Figure 4. Membranes were cut approximately in half to obtain a section with the higher molecular weight proteins, containing STAT4, and a section with the lower molecular weight proteins, containing SOD1, and were probed with the respective antibodies (against rabbit α-STAT4 or rabbit α-SOD1). After probing with the same secondary α-rabbit antibody, upper and lower sections of the blots were aligned to allow for unambiguous assignment of the individual lanes. Shorter exposures of ECL-treated membranes were used for the STAT4 blots (upper blot pictures) and longer ones for the fainter SOD1 blots (lower blot pictures). The approximate location of the separation line is indicated on the longer exposures.

Figure S8

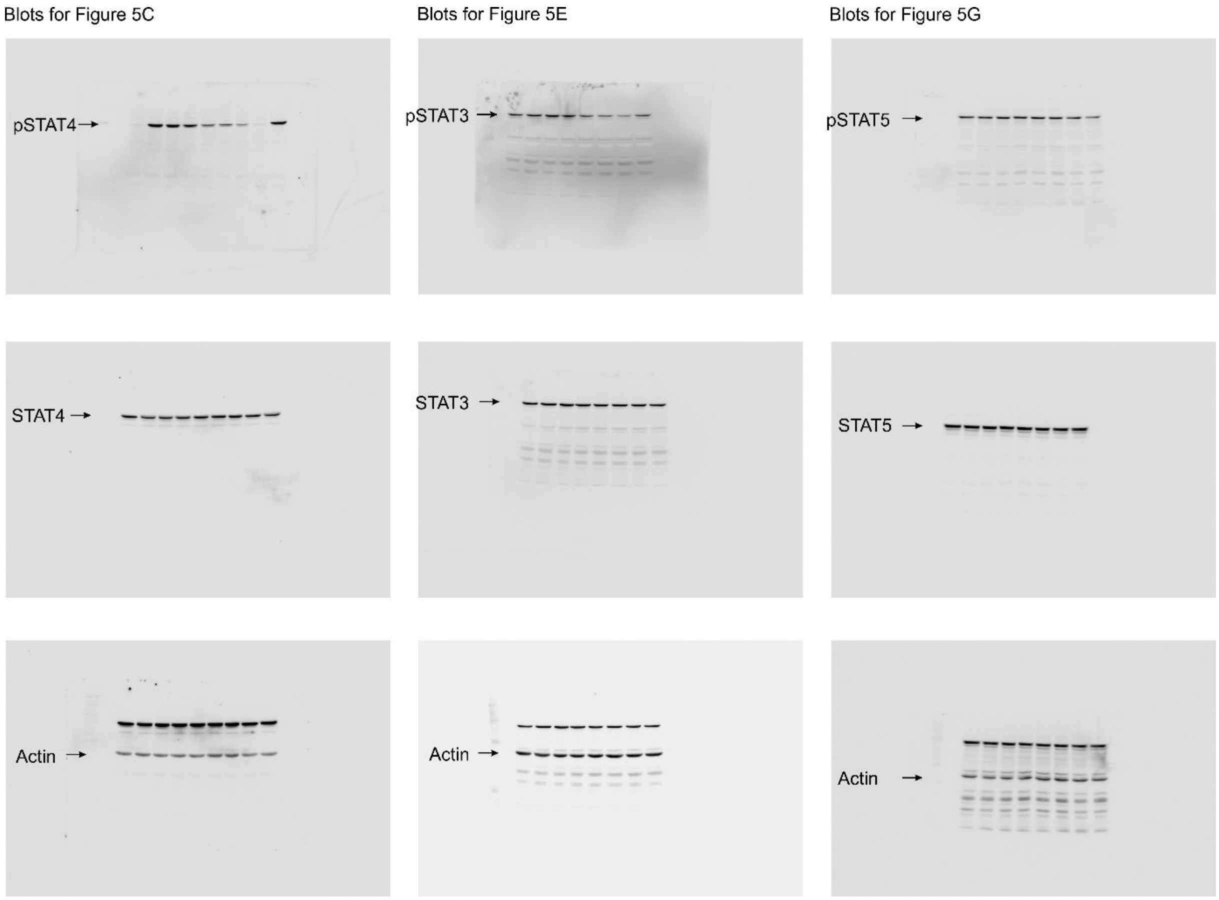

Figure S8: Uncropped blots used for generation of Figure 5.

Figure S9

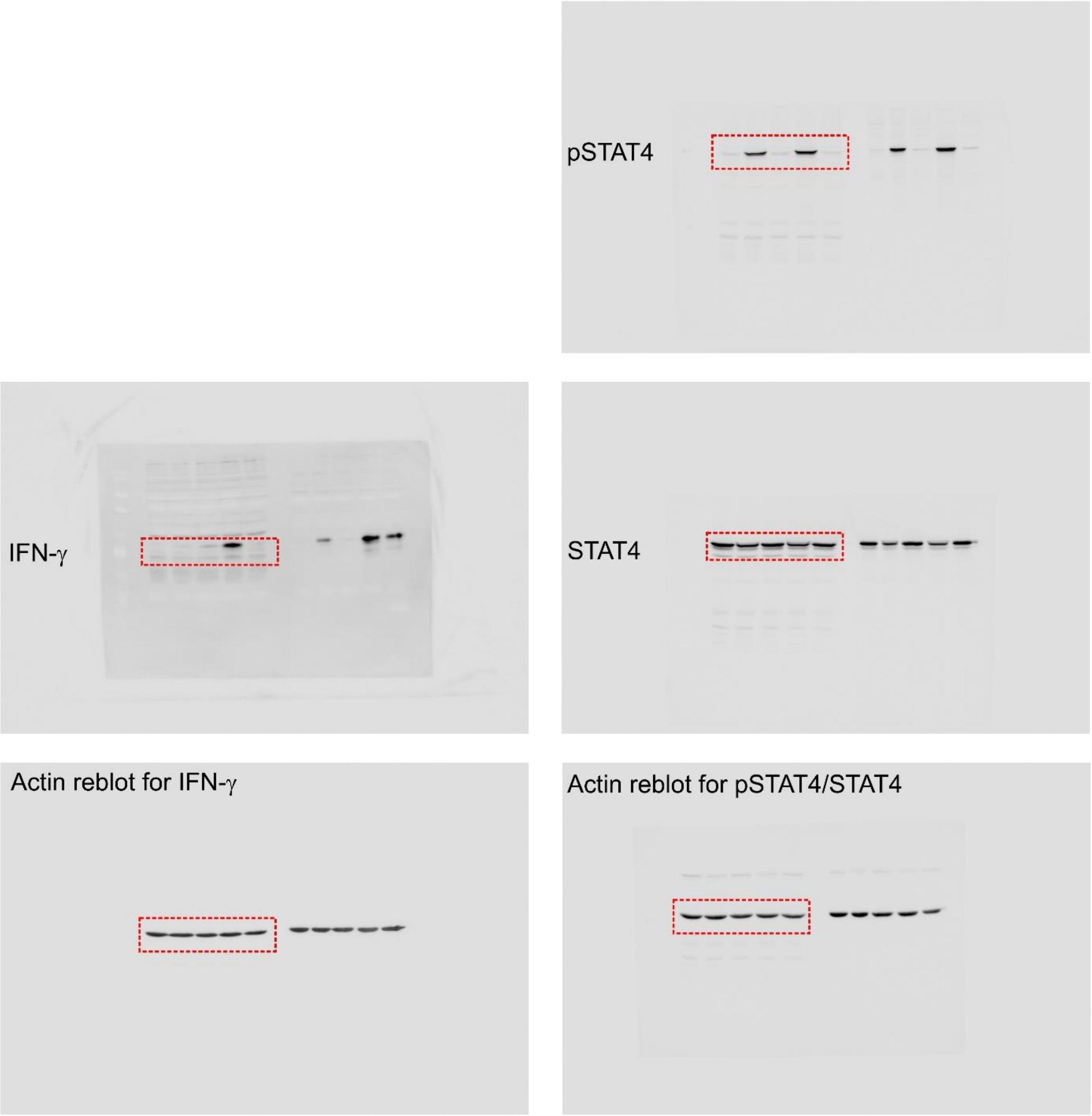

Figure S9: Uncropped blots used for generation of Figure 6.

Figure S10

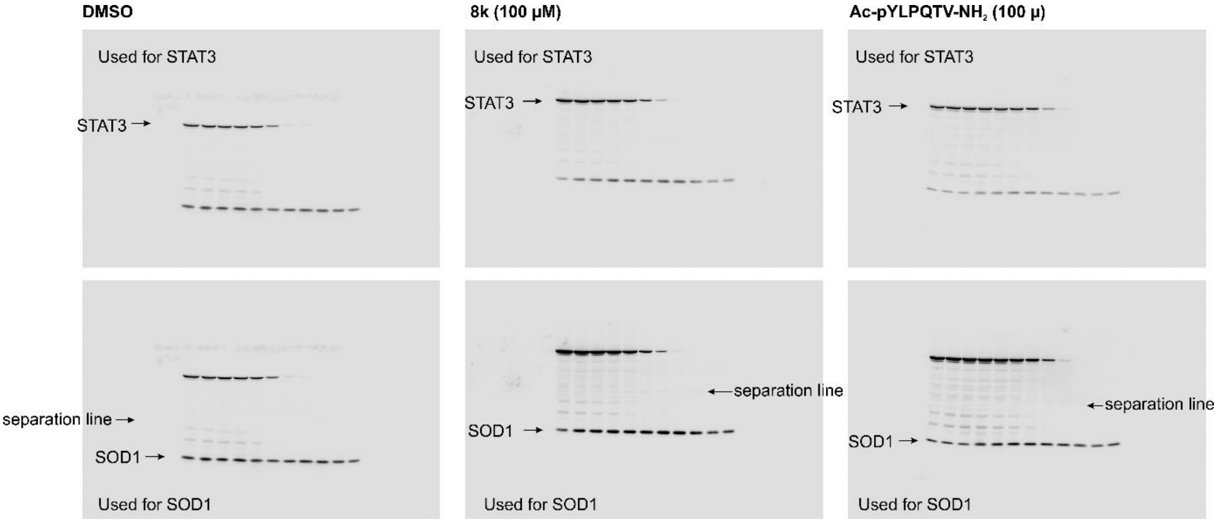

Figure S10: Uncropped blots used for generation of Figure S3. Membranes were cut approximately in half to obtain a section with the higher molecular weight proteins, containing STAT3, and a section with the lower molecular weight proteins, containing SOD1, and were probed with the respective antibodies (against rabbit α-STAT3 or rabbit α-SOD1). After probing with the same secondary α-rabbit antibody, upper and lower sections of the blots were aligned to allow for unambiguous assignment of the individual lanes. Shorter exposures of ECL-treated membranes were used for the STAT3 blots (upper blot pictures) and longer ones for the fainter SOD1 blots (lower blot pictures). The approximate location of the separation line is indicated on the longer exposures.

Figure S11

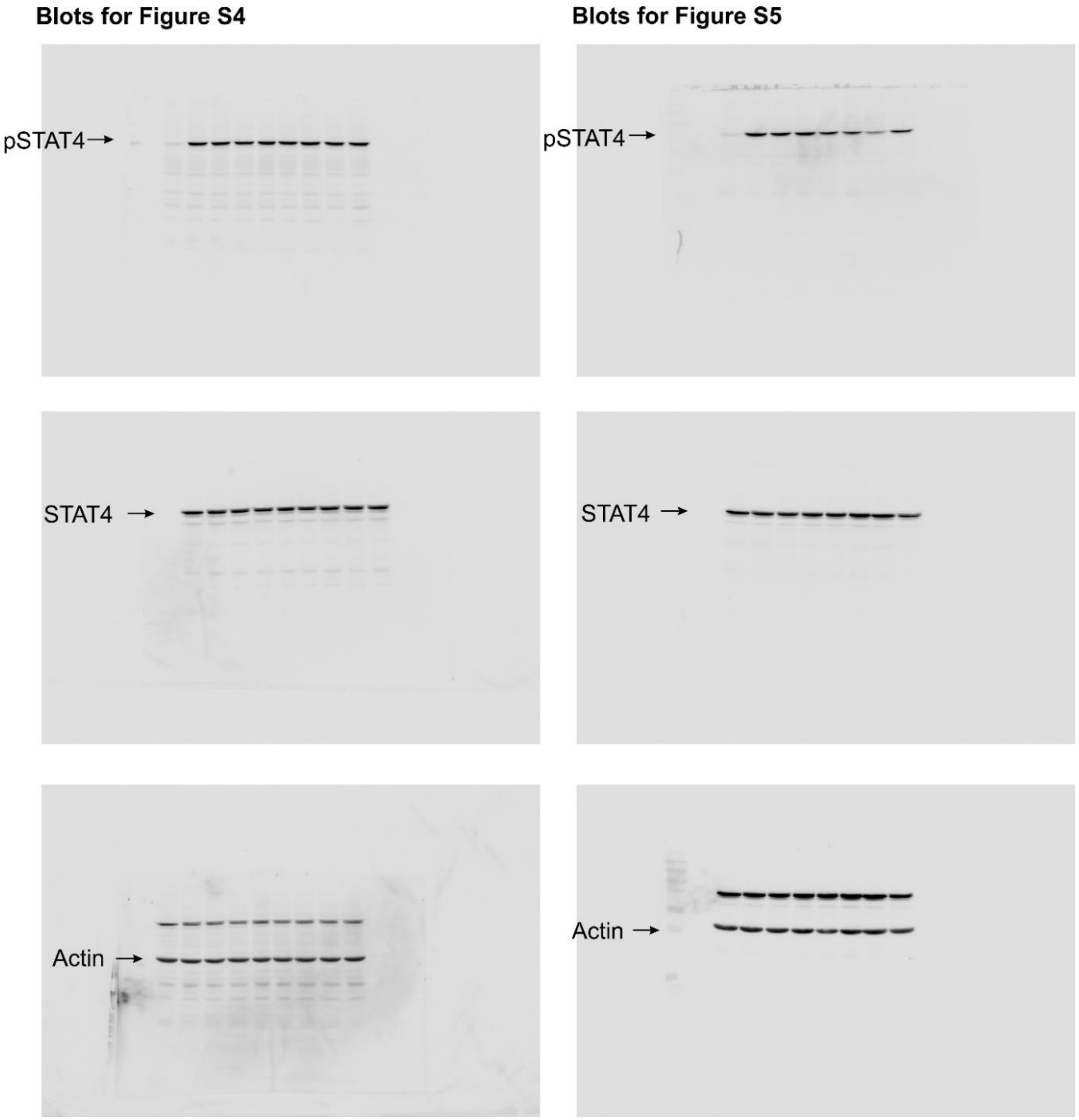

Figure S11: Uncropped blots used for generation of Figure S4 and S5.

Figure S12

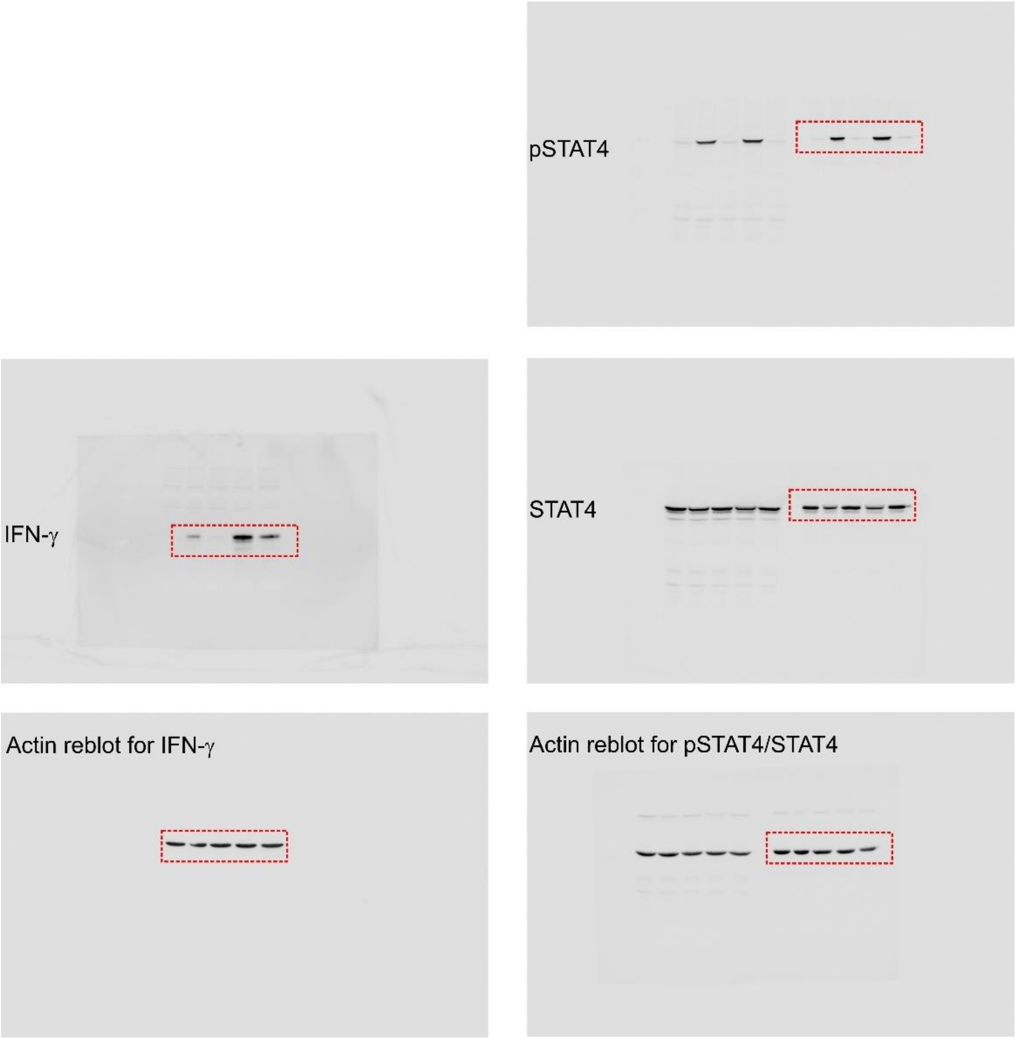

Figure S12: Uncropped blots used for generation of Figure S6.

Table S1: Structures of *p*-biaryl phosphates and their activity against STAT4 in FP assays. Mean values ± standard deviations are given (n = 3).

No	Structure	STAT4 IC_{50} (µM)	STAT4 K_i (µM)	No	Structure	STAT4 IC_{50} (µM)	STAT4 K_i (µM) or inhibition [%] at 200 µM
**1**	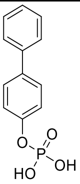	2.3 ± 0.2	1.1 ± 0.1	**6h**	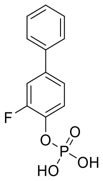	1.2 ± 0.2	0.56 ± 0.08
**6a**	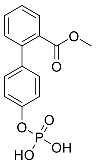	18 ± 2	9.1 ± 0.8	**6i**	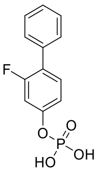	8.1 ± 0.1	3.8 ± 0.1
**6b**	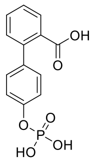	17 ± 1	7.8 ± 0.5	**6j**	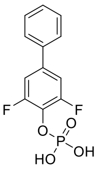	4.3 ± 0.3	2.0 ± 0.1
**6c**	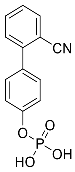	5.2 ± 0.6	2.7 ± 0.3	**6k**	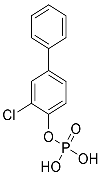	7.0 ± 2.0	3.3 ± 1.0
**6d**	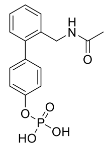	12 ± 1	6.2 ± 0.4	**6l**	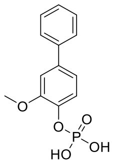	47 ± 5	22 ± 2
**6e**	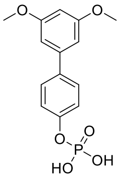	3.9 ± 0.2	1.9 ± 0.1	**6m**	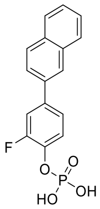	0.76 ± 0.01	0.35 ± 0.01
**6f**	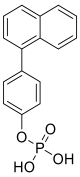	1.8 ± 0.2	0.84 ± 0.08	**4m**	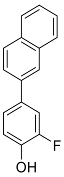	no inhibition at 200 µM	n.a.
**6g**	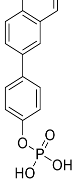	0.95 ± 0.19	0.44 ± 0.09				

n.a.: not applicable

Table S2: Structures of *p*-biaryl phosphates and their activity against STATs in FP assays. Mean values ± standard deviations are given (n = 3).

No	Structure	STAT4	STAT1	STAT3	STAT5a	STAT5b	STAT6
**1**	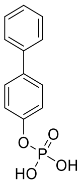	IC_{50} = 2.3 ± 0.2 µM<br>K_i = 1.1 ± 0.1 µM	IC_{50} = 16 ± 3 µM<br>K_i = 8.1 ± 1.4 µM	IC_{50} = 18 ± 3 µM<br>K_i = 8.4 ± 1.0 µM	IC_{50} = 42 ± 4 µM<br>K_i = 22 ± 2 µM	IC_{50} = 95 ± 6 µM<br>K_i = 47 ± 3 µM	IC_{50} = 15 ± 1 µM<br>K_i = 7.3 ± 0.4 µM
**6g**	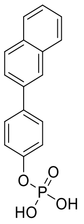	IC_{50} = 0.95 ± 0.19 µM<br>K_i = 0.44 ± 0.09 µM	IC_{50} = 22 ± 2 µM<br>K_i = 11 ± 1 µM	IC_{50} = 37 ± 2 µM<br>K_i = 18 ± 1 µM	IC_{50} = 55 ± 4 µM<br>K_i = 27 ± 2 µM	IC_{50} = 109 ± 11 µM<br>K_i = 53 ± 5 µM	IC_{50} = 19 ± 2 µM<br>K_i = 9.3 ± 1.1 µM
**6h**	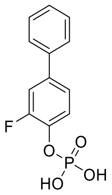	IC_{50} = 1.2 ± 0.2 µM<br>K_i = 0.56 ± 0.08 µM	IC_{50} = 13 ± 1 µM<br>K_i = 6.3 ± 0.6 µM	IC_{50} = 9.1 ± 0.6 µM<br>K_i = 4.5 ± 0.3 µM	IC_{50} = 57 ± 2 µM<br>K_i = 28 ± 1 µM	IC_{50} = 91 ± 14 µM<br>K_i = 45 ± 7 µM	IC_{50} = 90 ± 7 µM<br>K_i = 46 ± 3 µM
**6m**	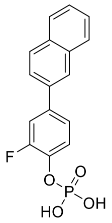	IC_{50} = 0.76 ± 0.01 µM<br>K_i = 0.35 ± 0.01 µM	IC_{50} = 7.8 ± 0.6 µM<br>K_i = 3.8 ± 0.3 µM	IC_{50} = 10 ± 1 µM<br>K_i = 5.0 ± 0.5 µM	IC_{50} = 45 ± 2 µM<br>K_i = 22 ± 1 µM	IC_{50} = 52 ± 4 µM<br>K_i = 26 ± 2 µM	IC_{50} = 57 ± 4 µM<br>K_i = 29 ± 2 µM

Table S3: Conditions tested for crystallization of the STAT4-SUMO fusion protein. See Table S7 for the composition of the sparse matrix screens.

Protein	Concentration (mg/ml)	Screens (Mastermix, M)	Inhibitor	Conc. Inhibitor (µM)	Molar excess inhibitor
SUMO-STAT4 Glu128-Ser676	5.0	M1-M7	-	-	-
SUMO-STAT4 Glu128-Ser676	5.0	M1-M7	**6m**	80	1/1
SUMO-STAT4 Glu128-Ser676	3.0	M1-M7	-	-	-
SUMO-STAT4 Glu128-Ser676	3.0	M1-M7	**6m**	59	1/1

Table S4: Conditions tested for crystallization of the tag-free STAT4 protein. See Table S7 for the composition of the sparse matrix screens.

Protein	Concentration (mg/ml)	Screens (Mastermix, M)	Inhibitor	Conc. Inhibitor (µM)	Molar excess inhibitor
STAT4 Glu128-Ser676	3.6	M1-M7	-	-	-
STAT4 Glu128-Ser676	3.6	M1-M7	**6m**	57	1/1
STAT4 Glu128-Ser676	6.4	M1-M7	-	-	-
STAT4 Glu128-Ser676	6.4	M1-M7	**6m**	100	1/1
STAT4 Ser130-Ser676	5.0	M1-M7	-	-	-
STAT4 Ser130-Ser676	5.0	M1-M7	**6m**	160	2/1
STAT4 Ser130-Ser676	5.0	M1-M7	**6h**	160	2/1
STAT4 Ser130-Ser676	5.0	M1-M7	IL12 receptor peptide	160	2/1
STAT4 Ser130-Ser676	7.2	M1-M7	-	-	-

Table S5: Conditions tested for crystallization of surface entropy-reduced STAT4 mutant proteins. See Table S7 for the composition of the sparse matrix screens.

Protein	Concentration (mg/ml)	Screens (Mastermix, M)	Inhibitor	Conc. Inhibitor (µM)	Molar excess inhibitor
STAT4 Ser130-Ser676 Glu280Ala/Glu281Ala/Gln282Ala	5.0	M1-M7	-	-	-
STAT4 Ser130-Ser676 Glu280Ala/Glu281Ala/Gln282Ala	5.0	M1-M7	**6m**	160	2/1
STAT4 Ser130-Ser676 Glu280Ala/Glu281Ala/Gln282Ala	5.0	M1-M7	**6h**	160	2/1
STAT4 Ser130-Ser676 Glu388Ala/Glu389Ala	5.0	M1-M7	**6h**	160	2/1

Protein	Concentration (mg/ml)	Screens (Mastermix, M)	Inhibitor	Conc. Inhibitor (µM)	Molar excess inhibitor
STAT4 Ser130-Ser676 Glu424Ala/Glu425Ala	5.0	M1-M7	-	-	-
STAT4 Ser130-Ser676 Glu424Ala/Glu425Ala	5.0	M1-M7	**6m**	160	2/1
STAT4 Ser130-Ser676 Glu424Ala/Glu425Ala	5.0	M1-M7	**6h**	160	2/1
STAT4 Ser130-Ser676 Lys210Ala/Glu211Ala	5.0	M1-M7	-	-	-
STAT4 Ser130-Ser676 Lys210Ala/Glu211Ala	5.0	M1-M7	**6m**	160	2/1
STAT4 Ser130-Ser676 Lys210Ala/Glu211Ala	5.0	M1-M7	**6h**	160	2/1
STAT4 Ser130-Ser676 Lys405Ala/Glu406Ala/Lys408Ala	5.0	M1-M7	-	-	-
STAT4 Ser130-Ser676 Lys405Ala/Glu406Ala/Lys408Ala	5.0	M1-M7	**6m**	160	2/1
STAT4 Ser130-Ser676 Lys405Ala/Glu406Ala/Lys408Ala	5.0	M1-M7	**6h**	160	2/1

Table S6: Conditions of the sparse matrix screens M1 to M7.

Name	Description
Master 1	Crystal Screen HT* (96 conditions)
Master 2	Index Screen HT* (96)
Master 3	SaltRx HT* (96)
Master 4	PEG/Ion Screen* (48), Grid Screen PEG6000* (24), Grid Screen PEG6000 LiCl* (24)
Master 5	Grid Screen MPD* (24), Grid screen PEG 3350* (24)), JBScreen Classic 8** (24), JBScreen Classic 9** (24)
Master 6	Grid Screen Ammonium Sulfate* (24), Grid Screen NaCl* (24), Grid Screen Malonate* (24), Quik Screen* (24)
Master 7	Natrix Screen* (48), MembFac Screen* (48)

*Hampton research, Aliso Viejo, California USA, **Jena Bioscience, Jena, Germany

Table S7: Structures of *p*-biaryl phosphonates and their activity against STAT4 in FP assays. Mean values ± standard deviations are given (n = 3).

No	Structure	STAT4 IC_{50} (µM) or inhibition at 100 µM (%)	STAT4 K_i (µM)	No	Structure	STAT4 IC_{50} (µM)	STAT4 K_i (µM)
**7**	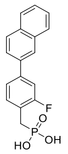	33 ± 4 % inhibition at 100 µM	n.a.	**8g**	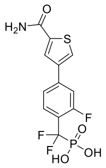	24 ± 4	11 ± 2
**8a**	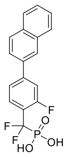	8.5 ± 1.0	4.0 ± 0.5	**8h**	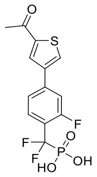	15 ± 1	7.1 ± 0.6
**8b**	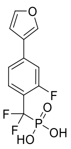	83 ± 2	39 ± 1	**8i**	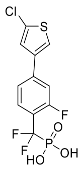	12 ± 2	5.5 ± 0.7
**8c**	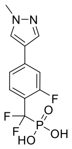	15 ± 1	8.0 ± 0.7	**8j**	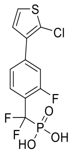	15 ± 1	7.3 ± 0.6
**8d**	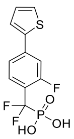	7.7 ± 0.4	3.6 ± 0.2	**8k**	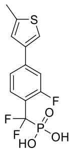	3.7 ± 0.4	1.7 ± 0.2
**8e**	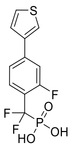	6.0 ± 0.6	2.8 ± 0.3	**8l**	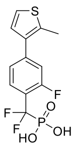	18 ± 2	8.7 ± 0.9
**8f**	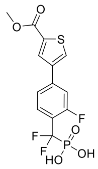	9.4 ± 0.2	4.4 ± 0.1	**8m**	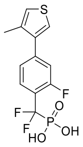	19 ± 1	8.9 ± 0.7

n.a.: not applicable

Table S8: Structures of *p*-biaryl phosphonates and their activity against STATs in FP assays. Mean values ± standard deviations are given (n = 3).

No	Structure	STAT4	STAT1	STAT3	STAT5a	STAT5b	STAT6
**8a**	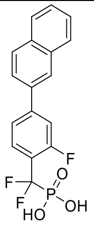	IC_{50} = 8.5 ± 1.0 µM<br>K_i = 4.0 ± 0.5 µM	IC_{50} = 63 ± 3 µM<br>K_i = 36 ± 2 µM	IC_{50} = 44 ± 2 µM<br>K_i = 22 ± 1 µM	IC_{50} = 54 ± 3 µM<br>K_i = 27 ± 1 µM	IC_{50} = 79 ± 3 µM<br>K_i = 39 ± 1 µM	IC_{50} = 30 ± 3 µM<br>K_i = 15 ± 2 µM
**8k**	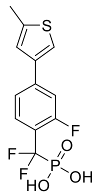	IC_{50} = 3.7 ± 0.4 µM<br>K_i = 1.7 ± 0.2 µM	IC_{50} = 48 ± 4 µM<br>K_i = 24 ± 2 µM	IC_{50} = 31 ± 4 µM<br>K_i = 15 ± 2 µM	IC_{50} = 79 ± 2 µM<br>K_i = 39 ± 1 µM	IC_{50} = 130 ± 14 µM<br>K_i = 64 ± 7 µM	IC_{50} = 26 ± 2 µM<br>K_i = 13 ± 1 µM

Table S9: Aggregation temperatures T_{agg} of STAT4 in the individual CETSA experiments in the presence of DMSO, test compounds and positive control peptide. p-values were calculated by Student's t-test (n = 3, two-tailed, two-sample equal variance).

	**T_{agg} (STAT4)** 3 independent experiments			**average (T_{agg})**	**standard deviation (T_{agg})**	**ΔT_{agg}**	**p**
DMSO	48.2	49.2	48.7	48.7	0.5		
**8a** (100 µM)	49.9	49.7	49.8	49.8	0.1	1.1	0.023
**8k** (100 µM)	50.5	49.6	49.8	50.0	0.5	1.3	0.031
Ac-GpYLPQNID (100 µM)	49.7	49.9	48.6	49.4	0.7	0.7	0.234
Ac-GpYLPQNID (500 µM)	52.0	51.0	52.4	51.8	0.7	3.1	0.003

Scheme S1

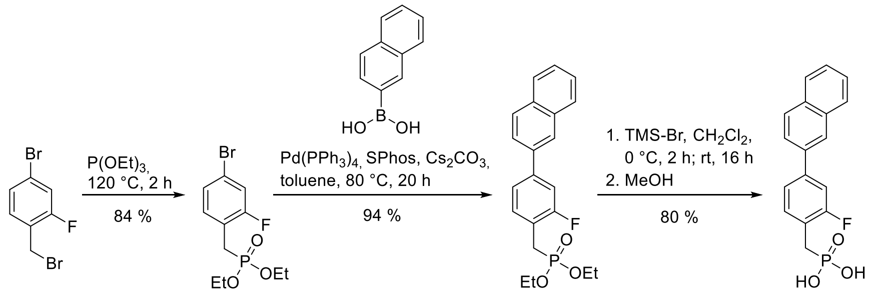

7a **7b** **7c** **7**

Scheme S1: Synthesis of benzyl phosphonate **7**.

Scheme S2

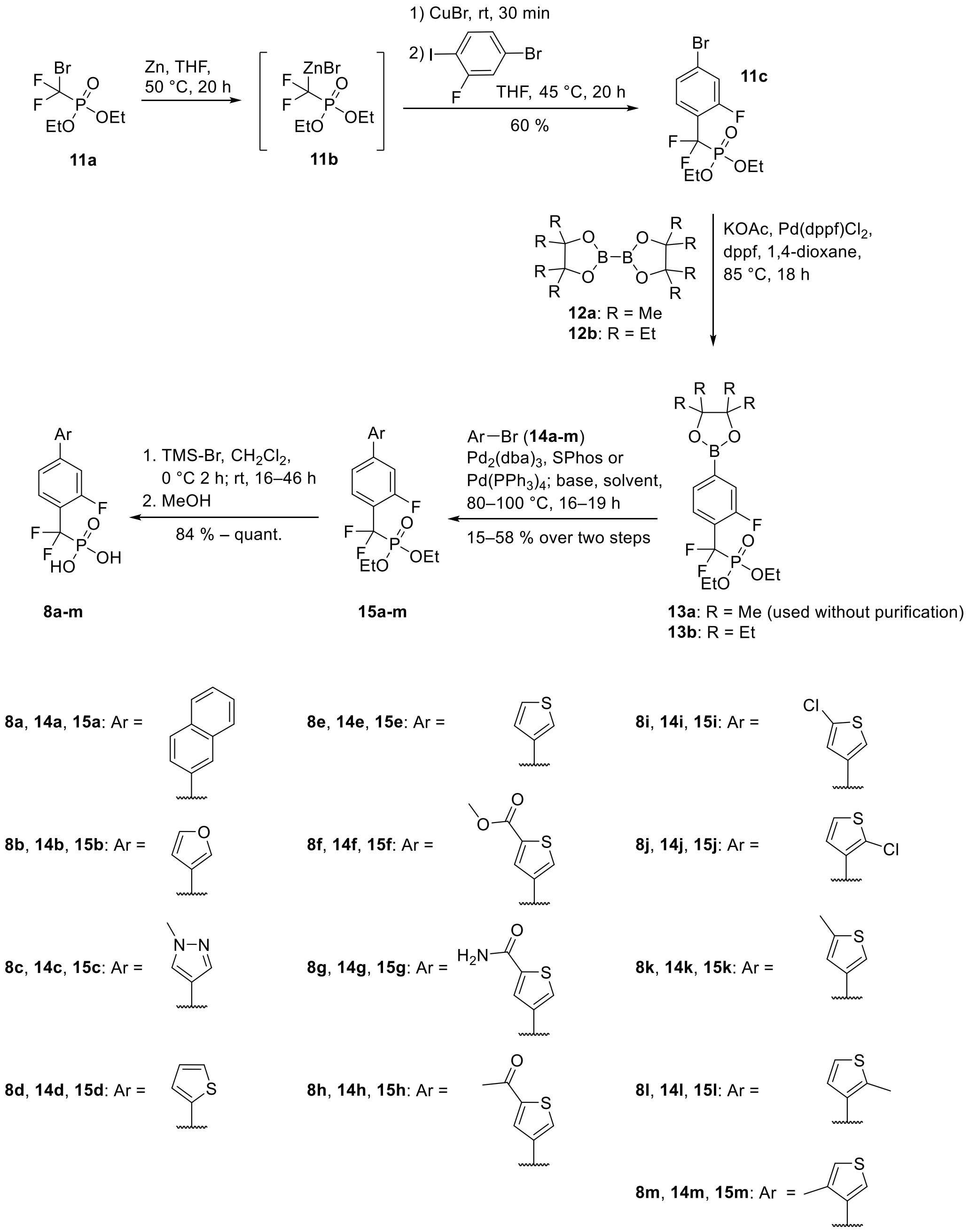

Scheme S2: Synthesis of α,α-difluorobenzyl phosphonates **8a-m**.

Methods

Fluorescence polarization assays

Competitive fluorescence polarization assays, which measure the abilities of test compounds (dissolved in DMSO) to displace a fluorescent-labeled peptide from the SH2 domain of a STAT protein, were carried out essentially as described.[2] Proteins were used at the following final concentrations: 23 nM or 28 nM for STAT4; between 56 nM and 91 nM for STAT1; 107 nM or 110 nM for STAT3; between 131 nM and 177 nM for STAT5a; between 62 nM and 85 nM for STAT5b; and 101 nM or 106 nM for STAT6. These concentrations represent the approximate

dissociation constant for peptide binding of the respective protein batches used in the assays. The following peptides were used: 5-carboxyfluorescein-GpYLPQNID-OH for STAT4; 5-carboxyfluorescein-GpYDKPHVL for STAT1; 5-carboxyfluorescein-GpYLPQTV-NH_2 for STAT3; 5-carboxyfluorescein-GpYLVLDKW for STAT5a/b, and 5-carboxyfluorescein-GpYVPWQDLI-OH for STAT6. All peptides were used at a final concentration of 10 nM. The buffer consisted of 10 mM Tris (pH 8.0), 50 mM NaCl, 1 mM EDTA, 1 mM DTT, 0.1 % Nonidet P-40 substitute, and 2 % DMSO. After incubation of protein and test compound for 1 h at room temperature, the fluorescent-labeled peptide was added. Samples were transferred to 384-well microtiter plates and fluorescence polarization was read after an additional 60 min of incubation time. Measurements were performed in triplicate. Percent binding was calculated based on a logarithmic curve fit using OriginPro 8G software. IC_{50} values were converted to K_i-values via the published equation[3] using the corresponding Excel spread sheet downloaded from
https://websites.umich.edu/~shaomengwanglab/software/calc_ki/index.html.

Error bars represent standard deviations. Ligand efficiencies (LE) were calculated by the formula LE = -1,37 x log K_i / number of non-hydrogen atoms.[4]

Isothermal Titration Calorimetry

Purification of 6xHis-tagged proteins for ITC experiments was carried out by affinity chromatography on His-Bind resin. The eluting fractions were analyzed by SDS-PAGE. Proteins were dialyzed against ITC buffer (10 mM Tris pH 8.0, 50 mM NaCl) using dialysis tubing with a 50 kDa cut-off. ITC experiments were performed using a VP-ITC Micro Calorimeter (MicroCal). **6m** was dissolved as a 100 mM stock in DMSO and diluted 1/1000 in ITC buffer (Tris pH 8.0 and 50 mM NaCl), resulting in a final concentration of 100 µM. **8k** was dissolved as a 5 mM stock in ITC buffer and diluted 1/25 in ITC buffer with DMSO, resulting in a final concentration of 200 µM **8k**. DMSO was added to a final concentration of 2% (v/v). To avoid errors caused by the detection of dilution heat, 0.1% (v/v) DMSO for **6m** or 2% (v/v) DMSO for **8k** experiments was added to the buffers containing the proteins and to the reference buffer. Proteins were degassed before the experiments using a ThermoVac sample degassing station. The following conditions were used for ITC experiments: 10 µM STAT4 protein for **6m** or 20 µM STAT4 protein for **8k** experiments, 100 µM **6m** or 200 µM **8k**, 28 injections with 10 µL single injection volume, 250 s initial delay, 500 s spacing between injections, 250 µcal/s reference power, 300 rpm stirring at 25 °C for **6m** or 220 rpm stirring at 25 °C for **8k**. Data analysis was performed using NITPIC[1, 5] and SEDPHAT,[6] applying a low-noise integration approach. Data were fitted using a one-site binding model. Figures were generated using GUSSI.[7] Experiments were carried out in quadruplicate for **6m** and triplicate for **8k**.

Cell culture experiments

NK-92 cells (DSMZ) were cultured in α-MEM containing 12.5% fetal bovine serum (FBS), 12.5% horse serum, 2 mM L-glutamine, penicillin/streptomycin and 50 U/ml IL-2. TALL-104 cells (ATCC, LGC Standards) were cultured in RPMI 1640 medium containing 20% FBS, 2 mM L-glutamine, penicillin/streptomycin and 100 U/ml IL-2. HCC-827 cells (DSMZ) and K562 cells (DSMZ) were cultured in RPMI 1640 medium containing 10% FBS, 2 mM L-glutamine and penicillin/streptomycin. HCC-827 cells were subcultured by briefly rinsing with sterile PBS followed by incubation with 0.25% Trypsin-EDTA solution before inactivation by addition of cell culture medium (all reagents apart from cell lines were from Gibco at Thermo Fisher Scientific). NK-92 cells were treated with test compound or DMSO in 1.5 ml medium in 12-well plates (Corning) for 2 h (final DMSO concentration 0.2%), in the presence of 50 ng/ml IL-12 (PeproTech). For IFN-γ experiments (Figure 6 and S6), NK-92 cells or TALL-104 cells were treated for 4 h in the presence of 50 ng/ml IL-12 and 10 ng/ml IL-18 (Bio-Techne). HCC-827 and K562 cells were treated with test compound or DMSO in the absence of IL-12. Cells were harvested by washing twice with ice-cold TBS, followed by resuspension in TBS supplemented with protease/phosphatase inhibitors (100 ng/ml aprotinin, 1 mM Na_3VO_4, 10 mM NaF, 1 mM PMSF). The adherent HCC-827 cells were mechanically separated from the culture surface using plastic cell scrapers (A. Hartenstein). Cells were lysed using three freeze-thaw cycles with liquid nitrogen and lysates cleared by centrifugation.

Lysates for CETSA

NK-92 cell lysates were prepared for CETSA experiments by washing cultured cells once with TBS and resuspending in TBS supplemented with Halt Protease Inhibitor Cocktail, EDTA-free (Fisher Scientific) at 3 x 10^6 cells per 100 µl. Cells were lysed using three freeze-thaw cycles with liquid nitrogen and lysates cleared by centrifugation.

CETSA

CETSA experiments were performed essentially using the published protocol.[8] Briefly, NK-92 cell lysates were incubated with test compound or DMSO for 15 min at room temperature (final DMSO concentration 2%). During this time, the treated lysate was divided into 10x 50 µl aliquots in 0.2 ml reaction tubes. Samples were then heated to the designated temperatures in a Veriti Pro Thermocycler (Thermo Fisher Scientific) for 3 min, followed by 3 min incubation at room temperature, and then placed on ice. The samples were transferred to 1.5 ml tubes before centrifugation at 20,000 g for 20 min at 4 °C, to remove aggregated proteins. The soluble fractions were subsequently analyzed by Western blotting.

Western blotting

The components of cell lysates from treated cells, or heat-treated lysates from CETSA, were separated by SDS-PAGE (10%), transferred to nitrocellulose membrane (Bio-Rad) and detected using monoclonal rabbit primary antibodies (Cell Signaling), α-rabbit-HRP secondary antibody (Dako) and Western Lightning Plus chemiluminescence reagent (Perkin Elmer). Bands were visualized using an ImageQuant system (GE Healthcare) and quantitated using ImageJ software (NIH).[9]

Attempted X-ray crystallography

STAT4 amino acids Glu128-Ser676 were cloned into the bacterial expression vector pE-SUMO-pro Amp. After expression in *E.coli* Rosetta, the 6His-SUMO-STAT4 fusion protein was purified by affinity chromatography. Traceless cleavage of the SUMO tag with 6His-Ulp-1 (SUMO-Protease, Sigma) was followed by a second chromatography step with a Ni-column that retains the 6xHis-SUMO fragment and leaves the STAT4 fragment in the flow-through.

Protein preparations were characterized by dynamic light scattering (DLS) and thermal shift assay prior to the crystallization experiments. Screening of crystallization conditions was carried out using the sitting drop vapor diffusion method in 96-well CrystalQuick™ round well plates. 672 different reservoir solutions (90 µL) were tested in each screening. Crystallization was attempted in the absence or presence of 1 or 2 equivalents of inhibitors. Pipetting was carried out using a mosquito®crystal robot (SPT Labtech). The sitting drop was composed of 0.1 µl protein solution and 0.1 µl reservoir solution. Plates were stored at 19°C in a protein crystallization imager.

In the first set of experiments, we tried to crystallize STAT4 as a SUMO-STAT4 fusion protein in the absence or presence of the most active biaryl phosphate **6m** at two different protein concentrations. Since none of the experiments resulted in crystals (Table S3), the SUMO tag was cleaved off as described above.

Screening for crystallization conditions for tag-free STAT4 was performed both in the absence or presence of 1 equivalent of **6m** or **6h**. Since the chosen protein concentration of 3.6 mg/ml appeared to be too low, screening was repeated with a higher concentration of 6.4 mg/ml STAT4 for all conditions (corresponding to 100 µM **6m**). Both the SUMO-STAT4 and the tag-free STAT4 preferentially precipitated under conditions containing polyethylene glycol. In one set of experiments, we also screened for conditions in the presence of the high-affinity peptide GY(PO_3H_2)LPQNID adapted from the IL-12 receptor. However, none of the conditions resulted in the growth of STAT4 crystals (Table S4).

The third set of experiments was carried out with point mutant STAT4 proteins with reduced surface entropy, based on predictions made by the surface entropy reduction prediction server (SERp server).[10] This resulted in the cloning, expression and purification of 5 double or triple mutants. Mutant STAT4 proteins were screened for crystallization in the absence or the presence of 2 equivalents of **6m** or **6h**, with the exception of the mutant STAT4 Glu388Ala/Glu389Ala, which was screened with **6h** only due to the low expression yield. Unfortunately, none of the conditions tested resulted in the growth of STAT4 crystals (Table S5).

General synthetic methods

Method 1: Suzuki coupling

__**1A:**__ A *Schlenk* flask was charged with aryl bromide (1.0 eq., selected from **3a**, **3c**-**e**, **3g**), the corresponding boronic acid (1.5 eq., selected from **2e**-**f**), K_3PO_4, $Pd(OAc)_2$ (1.0–3.0 mol%), and SPhos (L:Pd=2:1) under inert gas atmosphere. Dry toluene degassed by sonication was added to the mixture, which was stirred at 100° C for 16 h. The reaction was cooled and diluted with ethyl acetate, and the catalyst was removed by filtration through a thin layer of silica gel. The solvents were evaporated *in vacuo*, and the crude product was purified by flash column chromatography.

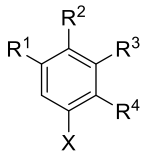

2a-f

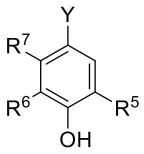

3a-g

	R^1	R^2	R^3	R^4	X
**2a**	H	H	H	CN	$B(OH)_2$
**2b**	H	H	H	$CH_2NHCOMe$	Br
**2c**	OMe	H	OMe	H	$B(OH)_2$
**2d**	H	H	CH=CH-CH=CH		$B(OH)_2$
**2e**	H	CH=CH-CH=CH		H	$B(OH)_2$
**2f**	H	H	H	H	$B(OH)_2$

	R^5	R^6	R^7	Y
**3a**	H	H	H	Br
**3b**	H	H	H	$B(OH)_2$
**3c**	H	F	H	Br
**3d**	H	H	F	Br
**3e**	F	F	H	Br
**3f**	H	Cl	H	Br
**3g**	H	OMe	H	Br

<u>1B:</u> A *Schlenk* flask was charged with aryl bromide (1.0 eq., selected from **14a**, **14d**-**e**, **14h**), K_3PO_4 (3.0 eq.), $Pd_2(dba)_3$ (0.50–1.0 mol%), and SPhos (L:Pd=4:1) under inert gas atmosphere. Crude aryl boronic ester **13a** (1.4 eq.) dissolved in dry toluene degassed by sonication was added to the mixture, which was stirred at 100 °C for 18 h. The reaction was subsequently cooled, diluted with water, and extracted three times with ethyl acetate. The combined organic layers were washed twice with water, after which they were dried over Na_2SO_4 and filtered. The volatiles were removed under reduced pressure, and the crude product was purified by flash column chromatography.

<u>1C:</u> A *Schlenk* flask was charged with aryl bromide (1.0 eq., selected from **14f**-**g**, **14k**-**m**), K_3PO_4 (3.0 eq.), and SPhos (L:Pd=4:1) under inert gas atmosphere. Aryl boronic ester **13b** (1.2–1.4 eq.) dissolved in dry toluene was added to the mixture, which was then degassed three times by the freeze-pump-thaw method. $Pd_2(dba)_3$ (0.50–1.0 mol%) was introduced, and the suspension was stirred at 100 °C for 19 h. Once cool, it was diluted with acetone and filtered through a thin layer of silica gel to remove the catalyst. The volatiles were evaporated under reduced pressure, and the residue was purified by flash column chromatography.

Method 2: Benzyl phosphorylation

To prepare the desired benzyl-protected phosphate, the corresponding phenol (1.0 eq.) was dissolved in dry acetonitrile (0.125 M), followed by the addition of CCl_4 (5.0–10 eq.), DIPEA (2.0–3.0 eq.), and catalytic amounts of DMAP (0.10 eq.). Dibenzyl phosphite (1.5–4.0 eq.) was added to the mixture at 0 °C and then allowed to warm to room temperature. Variations were applied as follows:

<u>2A:</u> CCl_4 (10 eq.), DIPEA (2.0 eq.), and dibenzyl phosphite (1.5 eq.)

<u>2B:</u> CCl_4 (5.0 eq.), DIPEA (2.0 eq.), and dibenzyl phosphite (1.5 eq.)

<u>2C:</u> CCl_4 (10 eq.), DIPEA (3.0 eq.), and dibenzyl phosphite (4.0 eq.)

Once the reaction was complete, as determined by TLC (usually after 0.5–3 h), a 0.5 M KH_2PO_4 solution (3 mL) was introduced, and the mixture was extracted with ethyl acetate. The combined organic phases were washed with brine and water, dried over Na_2SO_4, and filtered. The solvents were removed under reduced pressure, and the crude product was purified by flash column chromatography.

Method 3: Debenzylation by hydrogenolysis

10 wt. % Pd/C (25 mg) was added to a solution of the corresponding benzyl-protected phosphate in ethanol. The mixture was kept under an inert gas atmosphere, which was later replaced with hydrogen. After being stirred for 1–2 hours (TLC control), the reaction mixture was filtered through celite and washed with ethanol. The solvent was evaporated under reduced pressure, and C_{18} reversed-phase chromatography was used if additional purification was necessary. The product was dissolved in water and then lyophilized for isolation.

Method 4: Debenzylation by TMS-Br

Dibenzyl phosphate or ethyl-protected phosphonate dissolved in dry DCM (0.065 M) was cooled to 0 °C. TMS-Br (10–20 eq.) was added dropwise to the mixture, which was concurrently stirred for 2 h at the same temperature. After reaching ambient conditions, the mixture was stirred for a further 16–46 h. The reaction was then quenched with methanol, and the volatiles were removed under reduced pressure. The resulting residue was washed with methanol several times, and C_{18} reversed-phase column chromatography was used if additional purification was necessary. The product was dissolved in water and then lyophilized for isolation.

Synthesis and spectroscopic characterization of compounds

Dibenzyl [1,1'-biphenyl]-4-yl phosphate (**1a**)

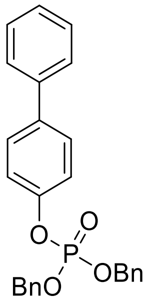

Compound **1a** was prepared from 4-phenylphenol (102 mg, 0.599 mmol, 1.0 eq.) according to **Method 2A**. The crude product was purified by flash column chromatography (ethyl acetate/hexane 1:4, *v/v*) to afford **1a** as a colorless oil.

Yield: 176 mg (68 %).

^{1}H-NMR (400 MHz, $CDCl_3$) δ 7.57 – 7.45 (m, 4H), 7.46 – 7.41 (m, 2H), 7.37 – 7.32 (m, 11H), 7.23 – 7.19 (m, 2H), 5.15 (d, J = 8.4 Hz, 4H; OCH_2Ph) ppm.

^{31}P-NMR (162 MHz, $CDCl_3$) δ = -6.08 (s) ppm.

^{13}C-NMR (101 MHz, $CDCl_3$) δ 150.20 (d, $^{2}J_{C\text{-}P}$ = 6.9 Hz; *C*OP), 140.4, 138.4, 135.6 (d, $^{3}J_{C\text{-}P}$ = 7.1 Hz), 129.0, 128.80, 128.75, 128.5, 128.2, 127.5, 127.2, 120.5 (d, $^{3}J_{C\text{-}P}$ = 5.0 Hz), 70.2 (d, $^{2}J_{C\text{-}P}$ = 5.8 Hz; O*C*H_2Ph).

HRMS (ESI) *m/z* $[M+H]^+$ calculated for $C_{26}H_{24}O_4P^+$: 431.1407, found: 431.1407.

IR (KBr): ṽ 3034 (w), 2959 (w), 1487 (m), 1281 (s), 1230 (m), 1053 (s), 1034 (s), 1024 (s), 950 (s), 938 (m), 741 (m), 698 (m) cm^{-1}.

UV/Vis (DCM): λ_{max} = 253 nm.

R_f = 0.31 (ethyl acetate/hexane 1:4, *v*/*v*).

[1,1'-Biphenyl]-4-yl phosphate (**1**)

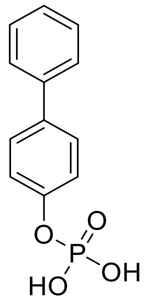

Compound **1** was synthesized from compound **1a** (94 mg, 0.22 mmol) according to **Method 3**. The product was obtained as a colorless oil.

Yield: 55 mg (quant).

^{1}H-NMR (400 MHz, CD_3OD) δ 7.63 – 7.56 (m, 4H), 7.45 – 7.39 (m, 2H), 7.35 – 7.26 (m, 3H) ppm.

^{31}P-NMR (162 MHz, CD_3OD) δ = -5.81 (s) ppm.

^{13}C-NMR (101 MHz, CD_3OD) δ 152.4 (d, $^{2}J_{C\text{-}P}$ = 7.1 Hz; COP), 141.6, 139.0, 129.9, 129.1, 128.3, 127.9, 121.7 (d, $^{3}J_{C\text{-}P}$ = 4.7 Hz).

HRMS (ESI) *m/z* $[2M\text{-}H]^-$ calculated for $C_{24}H_{21}O_8P_2^-$: 499.0717, found: 499.0700.

IR (KBr): ṽ 3434 (br), 3062 (w), 2921 (w), 2852 (w), 1632 (m), 1519 (w), 1488 (m), 1385 (w), 1254 (m), 968 (w), 842 (w), 761 (m), 693 (w) cm^{-1}.

UV/Vis (DCM): λ_{max} = 207; 253 nm.

Methyl 4'-hydroxy[1,1'-biphenyl]-2-carboxylate (**4a**)

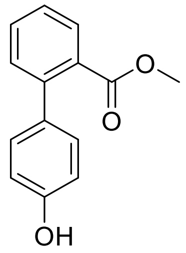

To a solution of 4'-hydroxybiphenyl-2-carboxylic acid (250 mg, 1.17 mmol) dissolved in 3.0 mL of methanol, 0.025 mL of concentrated H_2SO_4 was added. The reaction mixture was refluxed for 6 h before the solvent was evaporated. The crude product was purified by flash column chromatography (ethyl acetate/hexane 1:3, *v/v*) to give **4a** as a yellow oil, which solidified during storage.

Yield: 127 mg (48 %).

^{1}H-NMR (300 MHz, $CDCl_3$) δ = 7.81 – 7.76 (m, 1H), 7.54 – 7.46 (m, 1H), 7.41 – 7.33 (m, 2H), 7.22 – 7.15 (m, 2H), 6.88 – 6.80 (m, 2H), 5.05 (s, 1H; O*H*), 3.68 (s, 3H; OCH_3) ppm.

^{13}C-NMR (101 MHz, $CDCl_3$) δ = 169.7 (*C*O), 155.3 (*C*OH), 142.2, 133.8, 131.4, 130.91, 130.87, 129.9, 129.8, 127.0, 115.3, 52.2 (O*C*H_3) ppm.

HRMS (ESI) *m/z* $[M+H]^+$ calculated for $C_{14}H_{13}O_3^+$: 251.0678, found: 251.0687.

IR (KBr): ṽ 3567 (br), 3359 (w), 3273 (w), 2954 (w), 1715 (s), 1701 (s), 1612 (m), 1519 (m), 1307 (m), 1285 (m), 839 (m), 765 (m) cm^{-1}.

UV/Vis (DCM): λ_{max} = 232; 259 nm.

R_f = 0.38 (ethyl acetate/hexane 1:3, *v/v*).

m.p.: 100 °C, (lit. 105 °C).[11]

<u>Methyl 4'-[(dibenzylphosphono)oxy][1,1'-biphenyl]-2-carboxylate (**5a**)</u>

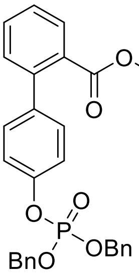

Compound **5a** was prepared from compound **4a** (100 mg, 0.440 mmol, 1.0 eq.) according to **Method 2B**. The crude product was purified by flash column chromatography (ethyl acetate/hexane 1:3, *v/v*) to give **5a** as a colorless oil, which solidified during storage.

Yield: 168 mg (78 %).

^{1}H-NMR (400 MHz, $CDCl_3$) δ = 7.85 – 7.79 (m, 1H), 7.56 – 7.50 (m, 1H), 7.44 – 7.38 (m, 1H), 7.34 (s, 11H), 7.25 – 7.16 (m, 4H), 5.15 (d, *J* = 8.2 Hz, 4H; OCH_2Ph), 3.61 (s, 3H; OCH_3) ppm.

^{31}P-NMR (162 MHz, $CDCl_3$) δ = -6.25 (s) ppm.

^{13}C-NMR (75 MHz, $CDCl_3$) δ = 169.1 (*C*O), 150.1 (d, $^2J_{C\text{-}P}$ = 7.1 Hz; *C*OP), 141.6, 138.5, 135.6 (d, $^3J_{C\text{-}P}$ = 6.9 Hz), 131.5, 130.9, 130.9, 129.8, 128.81, 128.77, 128.2, 127.5, 119.8 (d, $^3J_{C\text{-}P}$ = 5.1 Hz), 70.2 (d, $^2J_{C\text{-}P}$ = 6.0 Hz; O*C*H_2Ph), 52.1 (O*C*H_3) ppm.

IR (KBr): ṽ 3063 (w), 3034 (w), 2919 (w), 2849 (w), 1724 (s), 1607 (w), 1510 (m), 1498 (w) 1279 (m), 1213 (m) 1020 (s), 962 (s), 742 (m), 697 (m) cm^{-1}.

UV/Vis (DCM): λ_{max} = 230; 257; 292 nm.

R_f = 0.30 (ethyl acetate/hexane 1:3, *v/v*).

Methyl 4'-(phosphonooxy)[1,1'-biphenyl]-2-carboxylate (**6a**)

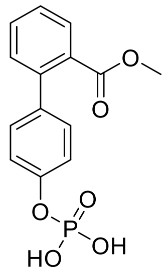

Compound **6a** was synthesized from compound **5a** (160 mg, 0.328 mmol) according to **Method 3**. The product was obtained as a sticky oil.

Yield: 101 mg (quant).

^{1}H-NMR (400 MHz, DMSO-d_6) δ = 7.70 – 7.65 (m, 1H), 7.61 – 7.54 (m, 1H), 7.47 – 7.36 (m, 2H), 7.24 – 7.11 (m, 4H), 3.58 (s, 3H; OC*H*$_3$) ppm.

^{31}P-NMR (162 MHz, DMSO-d_6) δ = -5.33 (s) ppm.

^{13}C-NMR (75 MHz, DMSO-d_6) δ = 168.7 (*C*O), 152.1 – 151.8 (m; *C*OP), 140.7, 135.2, 131.4, 130.8, 130.5, 129.2, 129.0, 127.2, 119.9 – 119.6 (m), 52.0 (O*C*H$_3$) ppm.

HRMS (ESI) *m/z* [M-H]$^-$ calculated for $C_{14}H_{12}O_6P^-$: 307.0377, found: 307.0379.

IR (KBr): $\tilde{\nu}$ 3437 (br), 3063 (w), 3034 (w), 2952 (w), 1719 (s), 1606 (m), 1514 (m), 1481 (m), 1292 (s), 1261 (m), 1246 (m), 1223 (m), 1091 (m), 965 (s), 845 (m), 763 (m) cm^{-1}.

UV/Vis (DCM): λ_{max} = 230; 285 nm.

Benzyl 4'-hydroxy[1,1'-biphenyl]-2-carboxylate (**4b**)

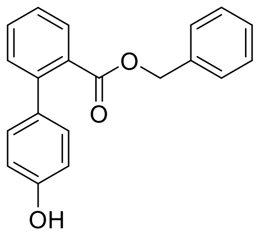

Under an argon atmosphere, 4'-hydroxybiphenyl-2-carboxylic acid (250 mg, 1.17 mmol, 1.0 eq.) was dissolved in 5 mL dry of DMF. $KHCO_3$ (140 mg, 1.40 mmol, 1.2 eq.) and benzyl bromide (210 µL, 302 mg, 1.75 mmol, 1.5 eq.) were added to the solution, and the reaction mixture was stirred for 16 h at 30 °C and then for 3 h at 40 °C. Ethyl acetate was subsequently added, followed by several washes with brine and water. The organic layer was then dried over Na_2SO_4, filtered, and evaporated under reduced pressure. The resulting residue was purified by flash column chromatography (ethyl acetate/hexane 1:3, *v/v*) to give **4b** as a semi-solid.

Yield: 211 mg (60 %).

^{1}H-NMR (400 MHz, $CDCl_3$) δ = 7.84 – 7.79 (m, 1H), 7.53 – 7.47 (m, 1H), 7.39 – 7.32 (m, 2H), 7.31 – 7.27 (m, 3H), 7.21 – 7.14 (m, 2H), 7.14 – 7.07 (m, 2H), 6.80 – 6.74 (m, 2H), 5.12 (s, 2H; OCH_2Ph) ppm.

^{13}C-NMR (76 MHz, $CDCl_3$) δ = 169.0 (*C*O), 155.1 (*C*OH), 142.0, 135.5, 133.8, 131.3, 131.0, 130.7, 129.8, 129.7, 128.4, 128.3, 128.1, 126.9, 115.1, 66.9 (OCH_2Ph) ppm.

HRMS (ESI) *m/z* $[M-H]^-$ calculated for $C_{20}H_{15}O_3^-$: 303.1026, found: 303.1030.

IR (film): ṽ 3392 (br), 3063 (w), 3032 (w), 2951 (w), 2927 (w), 1706 (s), 1613 (m), 1519 (m), 1283 (s), 836 (m), 763 (m), 739 (m), 697 (m) cm^{-1}.

UV/Vis (acetone): λ_{max} = 213; 262; 292 nm.

R_f = 0.61 (ethyl acetate/hexane 1:3, *v*/*v*).

Benzyl 4'-[(dibenzylphosphono)oxy][1,1'-biphenyl]-2-carboxylate (**5b**)

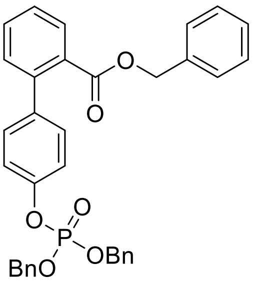

Compound **5b** was synthesized from compound **4b** (198 mg, 0.651 mmol, 1.0 eq.) according to **Method 2B**. The crude product was purified by flash column chromatography (ethyl acetate/hexane 1:3, *v/v*) to afford **5b** as an oily, colorless substance.

Yield: 150 mg (41 %).

^{1}H-NMR (400 MHz, $CDCl_3$) δ = 7.84 (dd, *J* = 7.8, 1.5 Hz, 1H), 7.54 – 7.49 (m, 1H), 7.44 – 7.38 (m, 1H), 7.38 – 7.27 (m, 10H), 7.26 – 7.18 (m, 3H), 7.14 – 7.02 (m, 3H), 5.14 (d, *J* = 8.3 Hz, 4H; OPO(OCH_2Ph)$_2$), 5.07 (s, 2H; OCH_2Ph) ppm.

^{31}P-NMR (162 MHz, $CDCl_3$) δ = -6.24 (s) ppm.

^{13}C-NMR (101 MHz, $CDCl_3$) δ = 168.5 (*C*O), 150.1 (d, $^2J_{C\text{-}P}$ = 6.9 Hz; *C*OP), 141.6, 138.4, 135.6 (d, $^3J_{C\text{-}P}$ = 6.9 Hz), 135.4, 131.5, 131.0, 130.9, 130.1, 129.9, 128.81, 128.76, 128.6, 128.4, 128.3, 128.2, 127.5, 119.8 (d, $^3J_{C\text{-}P}$ = 5.0 Hz), 70.1 (d, $^2J_{C\text{-}P}$ = 5.8 Hz; OPO(O*C*H_2Ph)$_2$), 67.1 (O*C*H_2Ph) ppm.

HRMS (ESI) *m/z* $[M+H]^+$ calculated for $C_{34}H_{30}O_6P^+$: 587.1594 , found: 587.1597.

IR (film): ṽ 3064 (w), 3033 (m), 3009 (w), 2957 (w), 2893 (w), 1721 (s), 1605 (m), 1512 (s), 1277 (s), 1242 (s), 1215 (s), 1019 (s), 954 (s), 748 (s), 697 (s) cm^{-1}.

UV/Vis (DCM): λ_{max} = 231; 282 nm.

R_f = 0.34 (ethyl acetate/hexane 1:3, *v*/*v*).

4'-(Phosphonooxy)[1,1'-biphenyl]-2-carboxylic acid (**6b**)

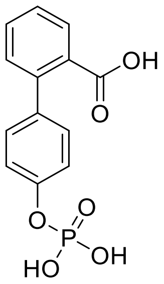

Compound **6b** was synthesized from compound **5b** (180 mg, 0.320 mmol) according to **Method 3**. The product was obtained as an off-white solid.

Yield: 95 mg (quant.)

^{1}H-NMR (400 MHz, DMSO-d_6) δ = 7.68 (dd, *J* = 7.7, 1.4 Hz, 1H), 7.56 – 7.50 (m, 1H), 7.45 – 7.39 (m, 1H), 7.38 – 7.32 (m, 1H), 7.29 – 7.11 (m, 4H) ppm.

^{31}P-NMR (162 MHz, DMSO-d_6) δ = -5.25 (s) ppm.

^{13}C-NMR (101 MHz, DMSO-d_6) δ = 169.8 (*C*O), 152.0 (d, $^{2}J_{C\text{-}P}$ = 6.2 Hz; *C*OP), 140.4, 135.4, 132.4, 130.7, 130.4, 129.1, 128.9, 127.0, 119.8 – 119.5 (m) ppm.

HRMS (ESI) *m/z* $[M-H]^-$ calculated for $C_{13}H_{10}O_6P^-$: 293.0220 , found: 293.0221.

IR (KBr): ṽ 3446 (br), 1717 (m), 1635 (w), 1513 (w), 1481 (w), 1237 (m), 1223 (m), 1139 (w), 1089 (w), 981 (w), 842 (w), 762 (m) cm^{-1}.

UV/Vis (DCM): λ_{max} = 231; 276 nm.

m.p.: 143 °C.

4'-Hydroxy[1,1'-biphenyl]-2-carbonitrile (**4c**)

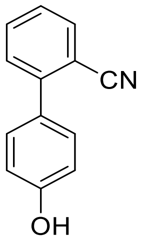

$Pd(PPh_3)_4$ (6.0 mg, 5.2 µmol, 3.1 mol%) and SPhos (3.0 mg, 7.3 µmol, L:Pd = 1.4:1) were suspended in degassed 1,4-dioxane under an argon atmosphere. 0.17 mL of a 2 M degassed aqueous Na_2CO_3 solution as well as 4-bromophenol (**3a**, 30 mg, 0.17 mmol, 1.0 eq.) and 2-cyanophenyl boronic acid (**2a**, 28 mg, 0.19 mmol, 1.1 eq.) were added to the reaction mixture, which was stirred at 90 °C for 24 h. The solvent was then evaporated, and the residue was taken up in water and extracted with ethyl acetate. The combined organic phases were dried over Na_2SO_4, filtered, and evaporated under reduced pressure. The crude product was purified by flash column chromatography (ethyl acetate/hexane 3:17, *v/v*) to give **4c** as a colorless solid.

Yield: 211 mg (60 %).

^{1}H-NMR (400 MHz, CD_3OD) δ = 7.76 (dd, *J* = 7.8, 1.4 Hz, 1H), 7.67 (td, *J* = 7.7, 1.4 Hz, 1H), 7.52 (dd, *J* = 8.0, 1.2 Hz, 1H), 7.44 (td, *J* = 7.6, 1.2 Hz, 1H), 7.42 – 7.38 (m, 2H), 6.93 – 6.88 (m, 2H) ppm.

^{13}C-NMR (101 MHz, CD_3OD) δ = 159.4 (*C*OH), 146.9, 134.8, 134.2, 131.1, 131.0, 130.7, 128.2, 119.9 (C*C*N), 116.5, 111.8 (*C*CN) ppm.

HRMS (ESI) *m/z* $[M-H]^-$ calculated for $C_{13}H_8NO^-$: 194.0611, found: 194.0608.

IR (KBr): ṽ 3368 (br), 3063 (w), 3026 (w), 2235 (m), 1615 (m), 1521 (m), 1447 (s), 1224 (s), 827 (m), 758 (s) cm^{-1}.

UV/Vis (DCM): λ_{max} = 272; 302 nm.

R_f = 0.27 (ethyl acetate/hexane 3:17, *v*/*v*).

m.p.: 187 °C, (lit. 166 – 167 °C).[12]

Dibenzyl 2'-cyano[1,1'-biphenyl]-4-yl phosphate (**5c**)

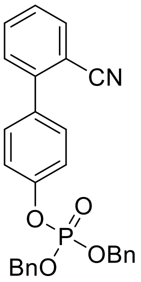

Compound **5c** was prepared from compound **4c** (75 mg, 0.38 mmol, 1.0 eq.) according to **Method 2B**. The crude product was purified by flash column chromatography (ethyl acetate/hexane 3:17, *v/v*) to afford a colorless oil.

Yield: 143 mg (82 %).

^{1}H-NMR (400 MHz, $CDCl_3$) δ = 7.76 (dd, *J* = 7.7, 1.4 Hz, 1H), 7.64 (td, *J* = 7.7, 1.4 Hz, 1H), 7.55 – 7.42 (m, 4H), 7.39 – 7.30 (m, 10H), 7.28 – 7.23 (m, 1H), 5.16 (d, $^3J_{H\text{-}P}$ = 8.6 Hz, 4H; OCH_2Ph) ppm.

^{31}P-NMR (162 MHz, $CDCl_3$) δ = -6.37 (p, $^3J_{P\text{-}H}$ = 8.5 Hz) ppm.

^{13}C-NMR (101 MHz, $CDCl_3$) δ = 151.2 (d, $^2J_{C\text{-}P}$ = 6.9 Hz; *C*OP), 144.6, 135.5 (d, $^3J_{C\text{-}P}$ = 6.8 Hz), 135.1 (d, $^5J_{C\text{-}P}$ = 1.1 Hz), 133.9, 133.0, 130.3 (d, $^4J_{C\text{-}P}$ = 0.8 Hz), 130.2, 128.9, 128.8, 128.3, 127.8, 120.5 (d, $^3J_{C\text{-}P}$ = 5.0 Hz), 118.7 (C*C*N), 111.4 (*C*CN), 70.3 (d, $^2J_{C\text{-}P}$ = 5.7 Hz; O*C*H_2Ph) ppm.

HRMS (ESI) *m/z* $[M+Na]^+$ calculated for $C_{27}H_{25}NO_4PNa^+$: 478.1178 , found: 478.1191.

IR (film): ṽ 3064 (w), 3034 (w), 2224 (w), 1606 (m), 1513 (m), 1478 (s), 1285 (s), 1217 (s), 1018 (s), 951 (s), 767 (m), 738 (m), 697 (m) cm^{-1}.

UV/Vis (DCM): λ_{max} = 231; 256; 290 nm.

R_f = 0.22 (ethyl acetate/hexane 3:17, *v*/*v*).

2'-Cyano[1,1'-biphenyl]-4-yl dihydrogen phosphate (**6c**)

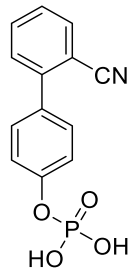

Compound **5c** (30 mg, 0.066 mmol, 1.0 eq.) in 2 mL of dry DCM was cooled down to 0 °C, TMS-Br (0.017 mL, 20 mg, 0.13 mmol, 2.0 eq.) was then added to the solution, which was stirred for 2 h. The reaction mixture was quenched with methanol, filtered through celite, and washed with dichloromethane and methanol. The volatiles were removed *in vacuo*, and **6c** was lyophilized to give an off-white powder.

Yield: 18 mg (quant.)

^{1}H-NMR (400 MHz, CD_3OD) δ = 7.80 (dd, *J* = 7.7, 1.3 Hz, 1H), 7.71 (td, *J* = 7.7, 1.4 Hz, 1H), 7.59 – 7.46 (m, 4H), 7.41 – 7.33 (m, 2H) ppm.

^{31}P-NMR (162 MHz, CD_3OD) δ = -5.23 (s) ppm.

^{13}C-NMR (101 MHz, CD_3OD) δ = 153.5 (d, $^2J_{C\text{-}P}$ = 6.6 Hz; *C*OP), 146.0, 136.0, 134.8, 134.4, 131.3, 131.2, 129.0, 121.6 (d, $^3J_{C\text{-}P}$ = 4.7 Hz), 119.5 (C*C*N), 112.2 (*C*CN) ppm.

HRMS (ESI) *m*/*z* $[M\text{-}H]^-$ calculated for $C_{13}H_9NO_4P^-$: 274.0275 , found: 274.0267.

IR (KBr): ṽ 3444 (br), 2925 (w), 2855 (w), 2251 (m), 1608 (s), 1514 (s), 1478 (s), 1241 (s), 1224 (s), 1173 (s), 980 (s), 845 (m), 765 (s) cm^{-1}.

UV/Vis (acetone): λ_{max} = 236; 254; 289 nm.

m.p.: 112 – 115 °C.

N-[(4'-hydroxy[1,1'-biphenyl]-2-yl)methyl]acetamide (**4d**)

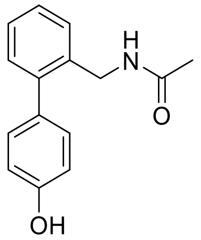

Compound **4d** was synthesized from *N*-(2-bromobenzyl)acetamide (**2b**, 200 mg, 0.880 mmol,1.0 eq.) and (4-hydroxyphenyl)boronic acid (**3b**, 130 mg, 0.960 mmol, 1.1 eq.) under an argon atmosphere. The starting material was dissolved in 2 mL of degassed 1,4-dioxane, to which 0.9 mL of degassed aqueous 2 M Na_2CO_3 solution, $Pd(PPh_3)_4$ (30 mg, 0.026 mmol, 3.0 mol%), and SPhos (16 mg, 0.039 mmol, L:Pd = 1.5:1) were added. The reaction mixture was stirred at 80 °C for 16 h, before being diluted with water and extracted with ethyl acetate. The combined organic phases were dried over Na_2SO_4, and the solvents

were removed under reduced pressure. The crude product was purified by flash column chromatography (methanol/dichloromethane 1:49, *v/v*) to give **4d** as a semi-solid.

Yield: 138 mg (65 %).

^{1}H-NMR (400 MHz, $CDCl_3$) δ = 7.40 – 7.36 (m, 1H), 7.35 – 7.30 (m, 2H), 7.25 – 7.23 (m, 1H), 7.20 – 7.13 (m, 2H), 6.92 – 6.86 (m, 2H), 5.73 (s, 1H), 5.48 (s, 1H), 4.44 (d, *J* = 5.5 Hz, 2H; C*H*$_2$NH), 1.93 (s, 3H; C*H*$_3$) ppm.

^{13}C-NMR (101 MHz, $CDCl_3$) δ = 169.9 (*C*O), 155.4 (*C*OH), 141.5, 135.3, 132.7, 130.5, 130.2, 128.8, 127.6, 127.5, 115.4, 41.9 (*C*H$_2$NH), 23.2 (*C*H$_3$) ppm.

HRMS (ESI) *m/z* $[M+Na]^+$ calculated for $C_{15}H_{15}NO_2Na^+$: 264.0995, found: 264.0994.

IR (KBr): ṽ 3333 (br), 3267 (br), 3093 (w), 3067 (w), 3024 (m), 2962 (w), 2917 (w), 1621 (s), 1258 (s), 1226 (s), 843 (s), 823 (m) cm^{-1}.

UV/Vis (DCM): λ_{max} = 231; 246 nm.

R_f = 0.80 (methanol/dichloromethane 1:19, *v/v*).

2'-[(Acetylamino)methyl][1,1'-biphenyl]-4-yl dibenzyl phosphate (**5d**)

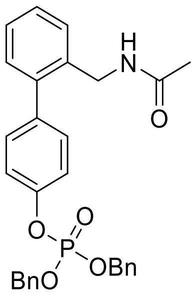

Compound **5d** was synthesized from compound **4d** (50 mg, 0.21 mmol, 1.0 eq.) according to **Method 2B**. The crude product was purified by flash column chromatography (methanol/dichloromethane 3:97, *v/v*) to afford an oil, which solidified during storage.

Yield: 56 mg (56 %).

^{1}H-NMR (400 MHz, $CDCl_3$) δ = 7.45 – 7.39 (m, 1H), 7.38 – 7.29 (m, 13H), 7.24 – 7.17 (m, 5H), 5.16 (d, *J* = 8.4 Hz, 4H; OC*H*$_2$Ph), 4.38 (d, *J* = 5.5, 2H; C*H*$_2$NH), 1.93 (s, 3H; C*H*$_3$) ppm.

^{31}P-NMR (162 MHz, $CDCl_3$) δ = -6.15 (s) ppm.

HRMS (ESI) *m/z* $[M+Na]^+$ calculated for $C_{29}H_{28}NO_5PNa^+$: 524.1597 , found: 524.1607.

IR (KBr): ṽ 3455 (br), 3065 (w), 3034 (w), 2955 (w), 1637 (w), 1275 (m), 1022 (s), 860 (w), 739 (m), 698 (m) cm^{-1}.

UV/Vis (DCM): λ_{max} = 228; 258; 307 nm.

R_f = 0.26 (methanol/dichloromethane 3:97, *v/v*).

m.p.: 64 °C.

2'-[(Acetylamino)methyl][1,1'-biphenyl]-4-yl dihydrogen phosphate (**6d**)

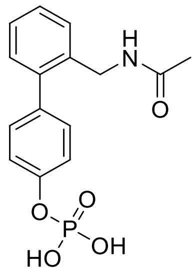

Compound **5d** (44 mg, 0.089 mmol, 1.0 eq.) was dissolved in 1.8 mL of dry DCM and cooled to 0 °C. TMS-Br (0.117 mL, 136 mg, 0.890 mmol, 10 eq) was added dropwise to the solution, which was warmed to room temperature and stirred for 16 h. The procedure was continued according to **Method C** to give **6d** as a powder.

Yield: 29 mg (quant.)

^{1}H-NMR (400 MHz, DMSO-d_6) δ = 8.23 (s, br, 1H; N*H*), 7.38 – 7.26 (m, 3H), 7.25 – 7.20 (m, 4H), 7.19 – 7.11 (m, 1H), 4.17 (d, *J* = 5.5 Hz, 2H; CH_2NH), 1.84 (s, 3H; CH_3) ppm.

^{31}P-NMR (162 MHz, DMSO-d_6) δ = -4.90 (s) ppm.

^{13}C-NMR (101 MHz, MeOD) δ = 173.0 (*C*O), 153.6 (*C*OP), 142.7, 137.3, 136.8, 131.2, 131.0, 129.4, 128.6, 128.3, 121.3, 42.4 (*C*H_2NH), 22.4 (*C*H_3) ppm.

HRMS (ESI) *m*/*z* $[M-H]^-$ calculated for $C_{15}H_{15}NO_5P^-$: 320.0693 , found: 320.0709.

IR (KBr): ṽ 3520 (br), 3290 (br), 3140 (w), 1636 (s), 1612 (s), 1513 (m), 1484 (m), 1237 (m), 953 (m), 848 (m), 763 (m) cm^{-1}.

UV/Vis (DMSO): λ_{max} = 262 nm.

m.p.: 142 °C.

3',5'-Dimethoxy[1,1'-biphenyl]-4-ol (**4e**)

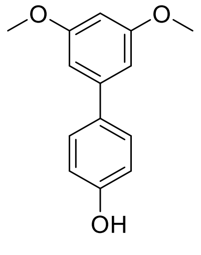

4-Bromophenol (**3a**, 300 mg, 1.73 mmol, 1.0 eq.), (3,5-dimethoxyphenyl) boronic acid (**2c**, 473 mg, 2.60 mmol, 1.5 eq.), SPhos (89 mg, 0.22 mmol, L:Pd = 2.4:1) and K_3PO_4 (734 mg, 3.47 mmol, 2.0 eq.) were suspended in 5 mL of toluene. The suspension was degassed by sonication and put under inert gas. $Pd(OAc)_2$ (20 mg, 0.090 mmol, 5.2 mol%) was then added, and the mixture was heated to 90 °C and stirred for 16 h. Once at room temperature, it was filtered through celite, and washed with water and ethyl acetate. The phases were separated, and ethyl acetate was used to extract the aqueous phase. The combined organic layers were washed with brine, dried over Na_2SO_4, and filtered. The solvents were evaporated under

reduced pressure, and the resulting residue was chromatographed on silica gel (ethyl acetate/hexane 3:17, *v/v*) to afford **4e** as a colorless solid.

Yield: 254 mg (64 %).

^{1}H-NMR (400 MHz, $CDCl_3$) δ = 7.49 – 7.44 (m, 2H), 6.92 – 6.87 (m, 2H), 6.68 (d, *J* = 2.3 Hz, 2H), 6.43 (t, *J* = 2.3 Hz, 1H), 4.87 (s, 1H; O*H*), 3.84 (s, 6H; OC*H*$_3$) ppm.

^{13}C-NMR (75 MHz, $CDCl_3$) δ = 161.2 (*C*OCH$_3$), 155.5 (*C*OH), 143.2, 134.1, 128.6, 115.7, 105.3, 98.9, 55.6 (O*C*H$_3$) ppm.

HRMS (ESI) *m/z* $[M+H]^+$ calculated for $C_{14}H_{15}O_3^+$: 231.1016, found: 231.1023.

IR (KBr): ṽ 3388 (br), 3032 (w), 3001 (m), 2940 (m), 2836 (m), 1598 (s), 1519 (s), 1207 (s), 923 (m), 862 (m), 817 (s) cm^{-1}.

UV/Vis (DCM): λ_{max} = 232; 264 nm.

R$_f$ = 0.13 (ethyl acetate/hexane 1:9, *v/v*).

m.p.: 64°C.

Dibenzyl 3',5'-dimethoxy[1,1'-biphenyl]-4-yl phosphate (**5e**)

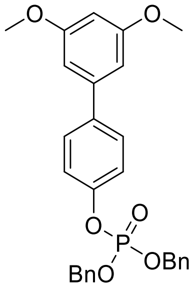

Compound **5e** was prepared from compound **4e** (140 mg, 0.608 mmol, 1.0 eq.) according to **Method 2B**. The crude product was purified by flash column chromatography (ethyl acetate/hexane 3:17, *v/v*) to give a colorless oil.

Yield: 132 mg (45 %).

^{1}H-NMR (400 MHz, $CDCl_3$) δ = 7.51 – 7.45 (m, 2H), 7.39 – 7.28 (m, 10H), 7.21 – 7.16 (m, 2H), 6.67 (d, *J* = 2.3 Hz, 2H), 6.46 (t, *J* = 2.2 Hz, 1H), 5.15 (d, $^3J_{H-P}$ = 8.4 Hz, 4H; OC*H*$_2$Ph), 3.85 (s, 6H; OC*H*$_3$) ppm.

^{31}P-NMR (162 MHz, $CDCl_3$) δ = -6.09 (p, $^3J_{P-H}$ = 8.4 Hz) ppm.

^{13}C-NMR (101 MHz, $CDCl_3$) δ = 161.2 (*C*OCH$_3$), 150.3 (d, $^2J_{C-P}$ = 7.0 Hz; *C*OP), 142.6, 138.4, 135.6 (d, $^3J_{C-P}$ = 6.9 Hz), 128.80, 128.75, 128.6, 128.2, 120.4 (d, *J* = 4.9 Hz), 105.5, 99.4, 70.2 (d, $^2J_{C-P}$ = 5.7 Hz; O*C*H$_2$Ph), 55.6 (O*C*H$_3$) ppm.

HRMS (ESI) *m/z* $[M+Na]^+$ calculated for $C_{28}H_{27}O_6PNa^+$: 513.1437, found: 513.1433.

IR (film): ṽ 3090 (w), 3065 (w), 3034 (w), 3004 (w), 2957 (w), 2940 (w), 2897 (w), 2838 (w), 1597 (s), 1510 (s), 1457 (s), 1283 (s), 1216 (s), 1156 (s), 1013 (s), 955 (s), 831 (m), 738 (m), 696 (s) cm^{-1}.

UV/Vis (DCM): λ_{max} = 230; 258 nm.

R_f = 0.22 (ethyl acetate/hexane 3:17, *v/v*).

3',5'-Dimethoxy[1,1'-biphenyl]-4-yl dihydrogen phosphate (**6e**)

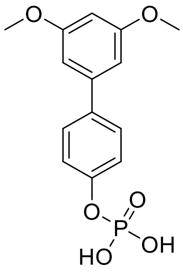

Compound **6e** was synthesized from compound **5e** (132 mg, 0.270 mmol) according to **Method 3**. The product was obtained as a sticky oil.

Yield: 84 mg (quant).

^{1}H-NMR (400 MHz, DMSO-d_6) δ = 7.64 – 7.56 (m, 2H), 7.26 – 7.18 (m, 2H), 6.73 (d, *J* = 2.2 Hz, 2H), 6.47 (t, *J* = 2.2 Hz, 1H), 3.79 (s, 6H; OC*H*$_3$) ppm.

^{31}P-NMR (162 MHz, DMSO-d_6) δ = -5.59 (s) ppm.

^{13}C-NMR (101 MHz, DMSO-d_6) δ = 160.8 (*C*OCH$_3$), 141.9 (*C*OP), 135.4, 127.8, 120.4, 104.7, 99.1, 55.3 (O*C*H$_3$) ppm.

HRMS (ESI) *m/z* $[M-H]^-$ calculated for $C_{14}H_{14}O_6P^-$: 309.0533 , found: 309.0534.

IR (film): ṽ 3445 (br), 3004 (w), 2958 (w), 2937 (w), 2838 (w), 1597 (s), 1511 (s), 1458 (m), 1205 (s), 1155 (s), 975 (s), 828 (m) cm^{-1}.

UV/Vis (acetone): λ_{max} = 206, 225, 259 nm.

4-(Naphthalen-1-yl)phenol (**4f**)

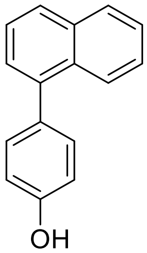

A mixture of 4-bromophenol (**3a**, 150 mg, 0.867 mmol, 1.0 eq.), 1-naphthylboronic acid (**2d**, 301 mg, 1.74 mmol, 2.0 eq.), K_3PO_4 (554 mg, 2.61 mmol, 3.0 eq.), $Pd_2(dba)_2$ (4.2 mg, 4.5 µmol, 0.52 mol%), and SPhos (7.2 mg, 18 µmol, L:Pd=4:1) was heated at 100 °C for 16 h in 4 mL of dry toluene degassed by sonication. The reaction mixture was diluted with water and extracted three times with ethyl acetate. The combined organic layers were washed with water, dried over Na_2SO_4, and filtered, after which the volatiles were removed under reduced pressure. The crude product was purified by two successive flash column chromatographies (ethyl acetate/hexane 1:9 *v*/*v*, then ethanol/hexane 1:19, *v*/*v*) to give **4f** as a light-yellow semi-solid.

Yield: 180 mg (94 %).

^{1}H-NMR (400 MHz, $CDCl_3$) δ = 7.99 – 7.81 (m, 3H), 7.59 – 7.32 (m, 6H), 7.02 – 6.92 (m, 2H), 5.29 (s, 1H; O*H*) ppm.

^{13}C-NMR (126 MHz, $CDCl_3$) δ = 155.0 (*C*OH), 140.0, 134.0, 133.4, 131.9, 131.5, 128.4, 127.5, 127.0, 126.2, 126.1, 125.8, 125.5, 115.3 ppm.

HRMS (ESI) *m*/*z* $[M-H]^-$ calculated for $C_{16}H_{11}O^-$: 219.0815, found: 219.0816.

IR (KBr): ṽ 3304 (br), 3045 (w), 3009 (w), 1610 (m), 1596 (m), 1515 (s), 1505 (m), 1226 (s), 835 (m), 800 (s), 774 (s) cm^{-1}.

UV/Vis ($CHCl_3$): λ_{max} = 317 nm.

R_f = 0.21 (ethyl acetate/hexane 1:9, *v*/*v*).

Dibenzyl 4-(naphthalen-1yl)phenyl phosphate (**5f**)

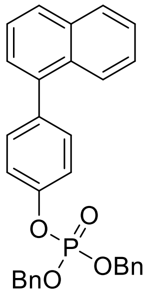

Compound **5f** was synthesized from compound **4f** (100 mg, 0.452 mmol, 1.0 eq.) according to **Method 2A**. The crude product was purified by flash column chromatography (ethyl acetate/hexane 1:19 to 3:7, *v*/*v*) to give **5f** as a light-yellow solid.

Yield: 156 mg (72 %).

^{1}H-NMR (400 MHz, $CDCl_3$) δ = 7.94 – 7.89 (m, 1H), 7.89 – 7.82 (m, 2H), 7.55 – 7.48 (m, 2H), 7.47 – 7.39 (m, 4H), 7.39 – 7.33 (m, 10H), 7.32 – 7.26 (m, 2H), 5.24 – 5.15 (m, 4H; OCH_2Ph) ppm.

^{31}P-NMR (162 MHz, $CDCl_3$) δ = -6.05 (s) ppm.

^{13}C-NMR (101 MHz, $CDCl_3$) δ = 150.1 (d, $^2J_{C-P}$ = 7.0 Hz; *C*OP), 139.3, 137.8, 135.6 (d, $^3J_{C-P}$ = 6.8 Hz), 133.9, 131.7, 131.4, 128.82, 128.76, 128.5, 128.2, 128.0, 127.1, 126.3, 125.98, 125.96, 125.5, 120.0 (d, $^3J_{C-P}$ = 4.9 Hz), 70.2 (d, $^2J_{C-P}$ = 5.9 Hz; O*C*H_2Ph) ppm.

HRMS (ESI) *m*/*z* $[M+H]^+$ calculated for $C_{30}H_{26}O_4P^+$: 481.1563, found: 481.1569.

IR (KBr): ṽ 3057 (m), 3037 (m), 3010 (w), 2955 (w), 2895 (w), 1606 (m), 1498 (s), 1275 (s), 1209 (s), 1050 (s), 1008 (s), 949 (s), 847 (s), 741 (s), 697 (s) cm^{-1}.

UV/Vis ($CHCl_3$): λ_{max} = 301 nm.

R_f = 0.44 (ethyl acetate/hexane 3:7, *v*/*v*).

m.p.: 92 – 95 °C.

4-(Naphthalen-1-yl)phenyl dihydrogen phosphate (**6f**)

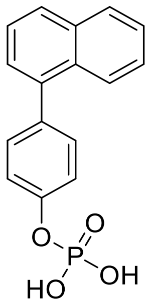

Compound **6f** was prepared from compound **5f** (53 mg, 0.11 mmol) according to **Method 3**. The crude product was purified by C_{18} reversed-phase column chromatography (acetonitrile/water 3:7, *v*/*v*). Lyophilization afforded **6f** as a beige solid.

Yield: 31 mg (97 %).

^{1}H-NMR (400 MHz, DMSO-d$_6$) 8.08 (m, 1H), 7.71 – 7.62 (m, 3H), 7.51 – 7.42 (m, 2H), 7.41 – 7.24 (m, 5H) ppm.

^{31}P-NMR (162 MHz, DMSO-d$_6$) δ = -4.74 (s) ppm.

^{13}C-NMR (101 MHz, DMSO-d$_6$) δ = 154.0 (d, $^{2}J_{C\text{-}P}$ = 5.8 Hz; *C*OP), 139.6, 133.5, 132.7, 131.0, 130.1, 128.3, 127.1, 126.7, 126.2, 125.8, 125.6, 125.5, 119.8 (d, $^{3}J_{C\text{-}P}$ = 4.9 Hz) ppm.

HRMS (ESI) *m*/*z* $[M\text{-}H]^-$ calculated for $C_{16}H_{12}O_4P^-$: 299.0479, found: 299.0479.

IR (KBr): ṽ 3201 (br), 3061 (w), 3045 (w), 3012 (w), 1607 (m), 1505 (m), 1397 (s), 1385 (m), 920 (m), 800 (m), 775 (m) cm^{-1}.

UV/Vis (H_2O): λ_{max} = 223, 291 nm.

R_f = 0.45 (acetonitrile/water 1:3, *v*/*v*).

m.p.: 143 – 150 °C.

4-(Naphthalen-2-yl)phenol (**4g**)

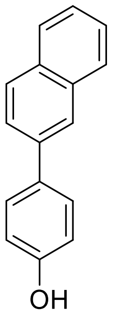

Compound **4g** was synthesized from 4-bromophenol (**3a**) (86.5 mg, 0.500 mmol, 1.0 eq.), 2-naphthylboronic acid (**2e**) (129 mg, 0.750 mmol, 1.5 eq.), K_3PO_4 (212 mg, 1.00 mmol, 3.0 eq.), $Pd(OAc)_2$ (3.37 mg, 15.0 µmol, 3.0 mol%), and SPhos (12.3 mg, 30.0 µmol, L:Pd=2:1) in 1 mL of dry and degassed toluene according to **Method 1A**. The crude product was chromatographed on silica gel (ethyl acetate/hexane 1:9, *v*/*v*) to give **4g** as a colorless oil.

Yield: 89 mg (78 %).

^{1}H-NMR (400 MHz, $CDCl_3$) δ = 7.94 – 7.88 (m, 2H), 7.86 – 7.82 (m, 1H), 7.54 – 7.36 (m, 6H), 6.99 – 6.94 (m, 2H), 4.92 (s, 1H; O*H*) ppm.

^{13}C-NMR (101 MHz, $CDCl_3$) δ = 154.9 (*C*OH), 139.8, 133.8, 133.3, 131.8, 131.3, 128.3, 127.4, 126.9, 126.0, 125.9, 125.7, 125.4, 115.2 ppm.

HRMS (ESI) *m/z* $[M\text{-}H]^-$ calculated for $C_{16}H_{11}O^-$: 219.0815, found: 219.0810.

IR (film): ṽ 3258 (br), 3041 (w), 1609 (m), 1514 (s), 1239 (s), 800 (s), 777 (s) cm^{-1}.

UV/Vis (DCM): λ_{max} = 237; 293 nm.

R_f = 0.25 (ethyl acetate/hexane 1:9, *v*/*v*).

Dibenzyl 4-(naphthalen-2-yl)phenyl phosphate (**5g**)

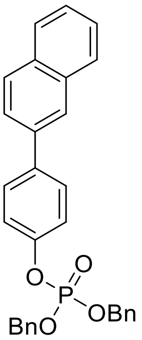

Compound **5g** was prepared from compound **4g** (75 mg, 0.34 mmol, 1.0 eq.) according to **Method 2C.** The crude product was purified by flash column chromatography (ethyl acetate/hexane 1:4, *v*/*v*) to afford a colorless oil.

Yield: 96 mg (59 %).

^{1}H-NMR (400 MHz, $CDCl_3$) δ = 7.91 (d, *J* = 8.3 Hz, 1H), 7.86 (t, *J* = 8.3 Hz, 2H), 7.55 – 7.48 (m, 2H), 7.47 – 7.39 (m, 4H), 7.36 (s, 2H), 7.29 – 7.24 (m, 10H), 5.22 – 5.17 (m, 4H; OCH_2Ph) ppm.

^{31}P-NMR (162 MHz, $CDCl_3$) δ = -6.06 (s) ppm.

^{13}C-NMR (101 MHz, $CDCl_3$) δ = 149.9 (d, $^{2}J_{C\text{-}P}$ = 6.8 Hz; *C*OP), 139.2, 137.6, 135.5 (d, $^{3}J_{C\text{-}P}$ = 7.2 Hz), 133.8, 131.5, 131.3, 128.7, 128.3, 128.1, 127.8, 127.0, 126.1, 125.8, 125.3, 119.9 (d, $^{3}J_{C\text{-}P}$ = 4.9 Hz), 70.1 (d, $^{2}J_{C\text{-}P}$ = 5.6 Hz; OCH_2Ph) ppm.

HRMS (ESI) *m/z* $[M+H]^+$ calculated for $C_{30}H_{26}FO_4P^+$: 481.1563, found: 481.1565.

IR (film): ṽ 3035 (w), 2955 (w), 2895 (w), 1605 (w), 1500 (s), 1285 (s), 1214 (s), 1016 (s), 969 (s), 951 (s), 738 (m), 697 (m) cm^{-1}.

UV/Vis ($CHCl_3$): λ_{max} = 241; 292 nm.

R_f = 0.36 (ethyl acetate/hexane 1:4, *v*/*v*).

4-(Naphthalen-2-yl)phenyl dihydrogen phosphate (**6g**)

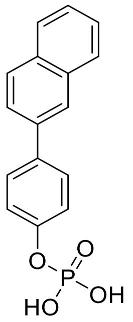

Compound **6g** was synthesized from compound **5g** (58 mg, 0.12 mmol) according to **Method 3**. The crude product was purified by C_{18} reversed-phase column chromatography (acetonitrile/water 3:7, *v*/*v*). Lyophilization afforded **6g** as a colorless solid.

Yield: 30 mg (83 %).

^{1}H-NMR (400 MHz, DMSO) δ = 7.97 (d, *J* = 8.2 Hz, 1H), 7.88 (dd, *J* = 16.3 Hz, 8.2 Hz, 2H), 7.58 – 7.45 (m, 2H), 7.39 (d, *J* = 7.0 Hz, 1H), 7.29 (s, 4H) ppm.

^{31}P-NMR (162 MHz, DMSO) δ = -5.77 (s) ppm.

^{13}C-NMR (101 MHz, DMSO) δ = 154.1 (d, $^{2}J_{C\text{-}P}$ = 5.4 Hz; *C*OP), 139.8, 133.7, 133.0, 131.2, 130.3, 128.5, 127.4, 127.0, 126.4, 126.0, 125.8, 125.6, 120.1 (d, $^{3}J_{C\text{-}P}$ = 5.0 Hz) ppm.

HRMS (ESI) *m*/*z* $[M\text{-}H]^-$ calculated for $C_{16}H_{12}FO_4P^-$: 299.0484, found: 299.0483.

IR (KBr): ṽ 3125 (br), 3012 (w), 2930 (w), 1608 (m), 1506 (s), 1397 (s), 1235 (s), 1186 (m), 1018 (w), 922 (m), 800 (m), 775 (s) cm^{-1}.

UV/Vis (H_2O): λ_{max} = 223; 291 nm.

R_f = 0.36 (acetonitrile/water 3:7, *v*/*v*).

m.p.: 144 – 146 °C.

3-Fluoro[1,1'-biphenyl]-4-ol (**4h**)

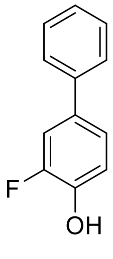

Compound **4h** was prepared from 4-bromo-2-fluorophenol (**3c**, 109 µL, 190 mg, 1.00 mmol, 1.0 eq.), phenylboronic acid (**2f**, 183 mg, 1.50 mmol, 1.5 eq.), K_3PO_4 (424 mg, 2.00 mmol, 2.0 eq.), $Pd(OAc)_2$ (2.2 mg, 0.010 µmol, 1.0 mol%), and SPhos (8.2 mg, 0.020 mmol, L:Pd = 2:1) in 2 mL of dry and degassed toluene according to **Method 1A**. The crude product was chromatographed on silica gel (ethyl acetate/hexane 1:9, *v*/*v*) to give **4h** as a colorless solid.

Yield: 120 mg (64 %).

^{1}H-NMR (400 MHz, $CDCl_3$) δ = 7.56 – 7.50 (m, 2H), 7.46 – 7.39 (m, 2H), 7.37 –7.25 (m, 3H), 7.10 – 7.04 (m, 1H), 5.13 (s, 1H; O*H*) ppm.
^{19}F-NMR (377 MHz, $CDCl_3$) δ = -140.82 (dd, $^3J_{F\text{-}H}$ = 12.0 Hz, $^4J_{F\text{-}H}$ = 9.0 Hz) ppm.
^{13}C-NMR (76 MHz, $CDCl_3$) δ = 151.4 (d, $^1J_{C\text{-}F}$ = 237.1 Hz; *C*F), 143.1 (d, $^2J_{C\text{-}F}$ = 14.4 Hz; *C*OH), 139,9 (d, $^4J_{C\text{-}F}$ = 2.5 Hz), 134.7 (d, $^3J_{C\text{-}F}$ = 6.2 Hz), 129.0, 127.4, 126.9, 123.5 (d, $^3J_{C\text{-}F}$ = 3.9 Hz), 117.6 (d, $^4J_{C\text{-}F}$ = 2.1 Hz), 114.3 (d, $^2J_{C\text{-}F}$ = 18.8 Hz) ppm.
HRMS (ESI) *m/z* $[M\text{-}H]^-$ calculated for $C_{12}H_8FO^-$: 187.0565, found: 187.0563.
IR (KBr): ṽ 3398 (s), 3037 (w), 1602 (s), 1531 (s), 1496 (s), 1309 (s), 1125 (s), 756 (s), 688 (s) cm^{-1}.
UV/Vis (DCM): λ_{max} = 257 nm.
R_f = 0.28 (ethyl acetate/hexane 1:9, *v*/*v*).
m.p.: 109 – 110 °C, (lit. 102 – 105 °C).[13]

Dibenzyl 3-fluoro[1,1'-biphenyl]-4-yl phosphate (**5h**)

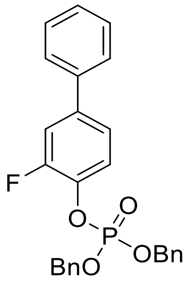

Compound **5h** was synthesized from phenol **4h** (84.8 mg, 0.456 mmol, 1.0 eq.) according to **Method 2C**. The crude product was purified by flash column chromatography (ethyl acetate/hexane 1:4, *v*/*v*) to afford a colorless oil.
Yield: 127 mg (63 %).
^{1}H-NMR (400 MHz, $CDCl_3$) δ = 7.54 – 7.50 (m, 2H), 7.47 – 7.42 (m, 2H), 7.38 – 7.31 (m, 13H), 7.28 – 7.25 (m, 1H), 5.19 (d, *J* = 8.3 Hz, 4H; OCH_2Ph) ppm.
^{19}F-NMR (377 MHz, $CDCl_3$) δ = -130.29 – -130.36 (m) ppm.
^{31}P-NMR (162 MHz, $CDCl_3$) δ = -6.03 (d, $^4J_{P\text{-}F}$ = 1.5 Hz) ppm.
^{13}C-NMR (101 MHz, $CDCl_3$) δ = 153.7 (dd, $^1J_{C\text{-}F}$ = 249.0 Hz, $^3J_{C\text{-}P}$ = 4.9 Hz; *C*F),139.8 (dd, $^3J_{C\text{-}F}$ = 6.6 Hz, $^5J_{C\text{-}P}$ = 1.7 Hz), 139.3 (d, $^4J_{C\text{-}F}$ = 2.1 Hz), 137.5 (dd, $^2J_{C\text{-}F}$ = 18.9 Hz, $^2J_{C\text{-}P}$ = 7.6 Hz; *C*OP),135.5 (d, $^3J_{C\text{-}P}$ = 6.9 Hz), 129.1, 128.8, 128.7, 128.2, 128.0, 127.1, 123.1 (dd, $^3J_{C\text{-}F}$ = 3.5 Hz, $^3J_{C\text{-}P}$ = 1.6 Hz), 122.7 (d, $^4J_{C\text{-}F}$ = 2.7 Hz, $^4J_{C\text{-}P}$ = 0.8 Hz), 115.7 (dd, $^2J_{C\text{-}F}$ = 19.1 Hz, $^4J_{C\text{-}P}$ = 1.1 Hz), 70.4 (d, $^2J_{C\text{-}P}$ = 5.9 Hz; O*C*H_2Ph) ppm.
HRMS (ESI) *m/z* $[M+H]^+$ calculated for $C_{26}H_{23}FO_4P^+$: 449.1313, found: 449.1322.
IR (film): ṽ 3038 (m), 2956 (m), 2894 (w), 1585 (m), 1520 (s), 1490 (s), 1286 (s), 1224 (s), 1124 (m), 1021 (s), 948 (s), 742 (s), 695 (s) cm^{-1}.
UV/Vis (DCM): λ_{max} = 252 nm.

R$_f$ = 0.29 (ethyl acetate/hexane 1:4, *v*/*v*).

3-Fluoro[1,1'-biphenyl]-4-yl dihydrogen phosphate (**6h**)

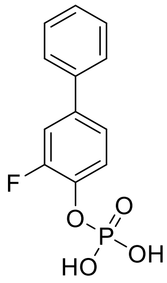

Compound **6h** was prepared from compound **5h** (45 mg, 0.10 mmol) according to **Method 3**. The crude product was purified by C_{18} reversed-phase column chromatography (acetonitrile/water 1:4, *v*/*v*). Lyophilization afforded **6h** as a colorless solid.

Yield: 20 mg (75 %).

^{1}H-NMR (400 MHz, D_2O) δ = 7.70 (d, $^4J_{H\text{-}F}$ = 7.3 Hz, 2H), 7.58 – 7.51 (m, 3H), 7.50 – 7.41 (m, 3H) ppm.

^{19}F-NMR (377 MHz, D_2O) δ = -131.42 (dd, $^3J_{F\text{-}H}$ = 11.8 Hz, $^4J_{F\text{-}H}$ = 7.8 Hz) ppm.

^{31}P-NMR (162 MHz, D_2O) δ = -3.67 (d, $^4J_{P\text{-}F}$ = 1.0 Hz) ppm.

^{13}C-NMR (101 MHz, D_2O) δ = 154.0 (dd, $^1J_{C\text{-}F}$ = 245.4 Hz, $^3J_{C\text{-}P}$ = 5.0 Hz; *C*F), 139.0 (d, $^4J_{C\text{-}F}$ = 1.8 Hz), 138.9 (dd, $^2J_{C\text{-}F}$ = 12.5 Hz, $^2J_{C\text{-}P}$ = 6.8 Hz; *C*OP), 137.7 (dd, $^3J_{C\text{-}F}$ = 6.5 Hz, $^5J_{C\text{-}P}$ = 1.7 Hz), 129.1, 127.8, 126.7, 122.9 (dd, $^4J_{C\text{-}F}$ = 2.9 Hz, $^4J_{C\text{-}P}$ = 1.3 Hz), 122.8 (dd, $^3J_{C\text{-}F}$ = 3.5 Hz, $^3J_{C\text{-}P}$ = 1.5 Hz), 114.9 (dd, $^2J_{C\text{-}F}$ = 19.6 Hz, $^4J_{C\text{-}P}$ = 1.1 Hz) ppm.

HRMS (ESI) *m*/*z* $[M\text{-}H]^-$ calculated for $C_{12}H_9FO_4P^-$: 267.0228, found: 267.0216.

IR (KBr): ṽ 3445 (br), 1635 (w), 1524 (w), 1491 (w), 1417 (w), 1311 (w), 1125 (w), 1019 (w), 935 (w), 759 (w), 696 (w) cm^{-1}.

UV/Vis (DCM): λ_{max} = 229 nm.

R$_f$ = 0.52 (acetonitrile/water 1:4, *v*/*v*).

m.p.: 191 – 193 °C.

2-Fluoro[1,1'-biphenyl]-4-ol (**4i**)

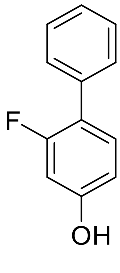

Compound **4i** was synthesized from 4-bromo-3-fluorophenol (**3d**, 191 mg, 1.00 mmol, 1.0 eq.), phenylboronic acid (**2f**, 183 mg, 1.50 mmol, 1.5 eq.), K_3PO_4 (424 mg, 2.00 mmol, 2.0 eq.), $Pd(OAc)_2$ (6.7 mg, 0.030 mmol, 3.0 mol%), and SPhos (25 mg, 0.060 mmol, L:Pd=2:1) in 2 mL

of dry and degassed toluene according to **Method 1A**. The crude product was purified by flash column chromatography (ethyl acetate/hexane 1:9, *v*/*v*) to give **4i** as a colorless solid.

Yield: 55 mg (30 %).

^{1}H-NMR (400 MHz, $CDCl_3$) δ = 7.53 – 7.47 (m, 2H), 7.46 – 7.39 (m, 2H), 7.37 – 7.30 (m, 2H), 6.73 – 6.64 (m, 2H), 4.98 (s, 1H, O*H*) ppm.

^{19}F-NMR (377 MHz, $CDCl_3$) δ = -115.65 – -115.78 (m) ppm.

^{13}C-NMR (101 MHz, $CDCl_3$) δ = 160.4 (d, $^{1}J_{C\text{-}F}$ = 248.4 Hz; *C*F), 156.2 (d, $^{3}J_{C\text{-}F}$ = 11.5 Hz; *C*OH), 135.8 (d, $^{3}J_{C\text{-}F}$ =1.2 Hz), 131.4 (d, $^{2}J_{C\text{-}F}$ = 5.5 Hz), 128.9 (d, $^{4}J_{C\text{-}F}$ = 2.7 Hz), 128.6, 127.3, 122.0 (d, $^{3}J_{C\text{-}F}$ = 13.9 Hz), 111.7 (d, $^{4}J_{C\text{-}F}$ = 3.4 Hz), 104.0 (d, $^{2}J_{C\text{-}F}$ = 26.3 Hz) ppm.

HRMS (ESI) *m*/*z* $[M\text{-}H]^-$ calculated for $C_{12}H_8FO^-$: 187.0565, found: 187.0559.

IR (KBr): ṽ 3432 (br), 3074 (w), 1626 (s), 1514 (m), 1485 (m), 1295 (m), 1115 (m), 765 (s), 701 (s) cm^{-1}.

UV/Vis ($CHCl_3$): λ_{max} = 254 nm.

R_f = 0.20 (ethyl acetate/hexane 1:9, *v*/*v*).

m.p.: 142 – 146 °C, (lit. 136 – 137 °C).[11]

<u>Dibenzyl 2-fluoro[1,1'-biphenyl]-4-yl phosphate (**5i**)</u>

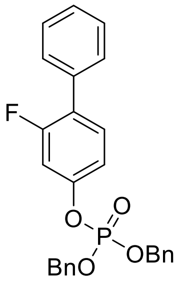

Compound **5i** was prepared from compound **4i** (30 mg, 0.16 mmol, 1.0 eq.) according to **Method 2C**. The crude product was purified by flash column chromatography (ethyl acetate/hexane 1:4, *v*/*v*) to afford a colorless oil.

Yield: 39 mg (55 %).

^{1}H-NMR (400 MHz, $CDCl_3$) δ = 7.52 – 7.47 (m, 2H), 7.47 – 7.41 (m, 2H), 7.39 – 7.32 (m, 12H), 7.03 – 6.99 (m, 1H), 6.97 – 6.92 (m, 1H), 5.16 (d, *J* = 8.7 Hz, 4H; OCH_2Ph) ppm.

^{19}F-NMR (377 MHz, $CDCl_3$) δ = -114.38 – -114.49 (m) ppm.

^{31}P-NMR (162 MHz, $CDCl_3$) δ = -6.30 (s) ppm.

^{13}C-NMR (101 MHz, $CDCl_3$) δ = 159.5 (d, $^{1}J_{F\text{-}C}$ = 250.0 Hz; *C*F), 150.3 (dd, $^{3}J_{C\text{-}F}$ = 11.1 Hz, $^{2}J_{C\text{-}P}$ = 6.7 Hz; *C*OP), 135.3 (d, $^{3}J_{P\text{-}C}$ = 6.6 Hz), 135.0 (d, $^{3}J_{C\text{-}F}$ = 1.4 Hz), 131.2 (d, $^{2}J_{C\text{-}F}$ = 5.4 Hz),128.9 (d, $^{4}J_{C\text{-}F}$ = 2.7 Hz), 128.8, 128.6, 128.5, 128.1, 127.8, 126.1 (dd, $^{3}J_{C\text{-}F}$ = 13.8 Hz, $^{2}J_{P\text{-}C}$ = 1.2 Hz), 116.1 (dd, $^{3}J_{C\text{-}P}$ = 4.8 Hz, $^{4}J_{C\text{-}F}$ = 3.8 Hz), 108.7 (dd, $^{2}J_{C\text{-}F}$ = 26.1 Hz, $^{3}J_{C\text{-}P}$ = 4.8 Hz), 70.2 (d, $^{2}J_{P\text{-}C}$ = 5.8 Hz; O*C*H_2Ph) ppm.

HRMS (ESI) *m*/*z* $[M+Na]^+$ calculated for $C_{26}H_{22}O_4PNa^+$: 471.1132, found: 471.1126.

IR (film): ṽ 3035 (w), 2955 (w), 2895 (w), 1618 (m), 1485 (s), 1281 (s), 1224 (m), 989(s), 895 (s), 741 (s), 698 (s) cm^{-1}.

UV/Vis ($CHCl_3$): λ_{max} = 246 nm.

$\mathbf{R_f}$ = 0.23 (ethyl acetate/hexane 1:4, *v*/*v*).

2-Fluoro[1,1'-biphenyl]-4-yl dihydrogen phosphate (**6i**)

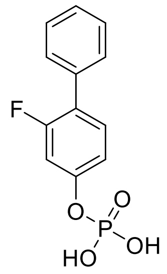

Compound **6i** was synthesized from compound **5i** (19.3 mg, 0.040 mmol) according to **Method 3**. The crude product was purified by C_{18} reversed-phase column chromatography (acetonitrile/water 1:4, *v*/*v*). Lyophilization afforded the product as a colorless solid.

Yield: 8.7 mg (77 %).

^{1}**H-NMR** (400 MHz, D_2O) δ = 7.64 (d, *J* = 8.5 Hz, 2H), 7.55 (t, *J* = 7.5 Hz, 2H), 7.52 – 7.44 (m, 2H), 7.17 – 7.09 (m, 2H) ppm.

^{19}**F-NMR** (377 MHz, D_2O) δ = -116.86 – -116.97 (m) ppm.

^{31}**P-NMR** (162 MHz, D_2O) δ = -2.05 (s) ppm.

^{13}**C-NMR** (76 MHz, D_2O) δ = 159.4 (d, $^{1}J_{C\text{-}F}$ = 245.5 Hz; *C*F), 153.6 (dd, $^{3}J_{C\text{-}F}$ = 11.5 Hz, $^{2}J_{C\text{-}P}$ = 7.0 Hz; *C*OP), 135.5 (d, $^{3}J_{C\text{-}F}$ = 1.1 Hz), 130.9 (d, $^{2}J_{C\text{-}F}$ = 5.0 Hz), 128.9 (d, $^{4}J_{C\text{-}F}$ = 2.9 Hz), 128.8, 127.8 , 123,3 (d, $^{3}J_{C\text{-}F}$ = 13.6 Hz), 116.6 (dd, $^{3}J_{C\text{-}P}$ = 4.7 Hz, $^{4}J_{C\text{-}F}$ = 3.4 Hz), 108.3 (dd, $^{2}J_{C\text{-}F}$ = 25.6 Hz, $^{3}J_{C\text{-}P}$ = 4.8 Hz) ppm.

HRMS (ESI) *m*/*z* $[M\text{-}H]^-$ calculated for $C_{12}H_9FO_4P^-$: 267.0228, found: 267.0226.

IR (KBr): ṽ 3446 (br), 1624 (w), 1486 (w), 1276 (w), 1158 (w), 1114 (m), 1038 (w), 990 (w), 762 (w), 696 (w) cm^{-1}.

UV/Vis (H_2O): λ_{max} = 200; 246 nm.

$\mathbf{R_f}$ = 0.34 (acetonitrile/water 1:4, *v*/*v*).

m.p.: 195 – 200 °C.

3,5-Difluoro[1,1'-biphenyl]-4-ol (**4j**)

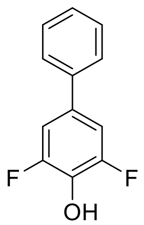

Compound **4j** was prepared from 4-bromo-2,6-difluorophenol (**3e**, 209 mg, 1.00 mmol, 1.0 eq.), phenylboronic acid (**2f**, 183 mg, 1.50 mmol, 1.5 eq.), K_3PO_4 (424 mg, 2.00 mmol, 2.0 eq.), $Pd(OAc)_2$ (2.2 mg, 0.010 mmol, 1.0 mol%), and SPhos (8.2 mg, 0.020 mmol, L:Pd=2:1) in 1 mL of dry and degassed toluene according to **Method 1A**. The crude product was purified by flash column chromatography (ethyl acetate/hexane 1:9, *v*/*v*) to give **4j** as a colorless solid.

Yield: 137 mg (66 %).

^{1}H-NMR (400 MHz, $CDCl_3$) δ = 7.50 (d, $^3J_{H\text{-}H}$ = 7.4 Hz, 2H), 7.43 (t, $^3J_{H\text{-}H}$ = 7.4 Hz, 2H), 7.35 (t, $^3J_{H\text{-}H}$ = 7.4 Hz, 1H), 7.19 (d, $^3J_{H\text{-}F}$ = 9.1 Hz, 2H), 5.09 (s, 1H; O*H*) ppm.

^{19}F-NMR (377 MHz, $CDCl_3$) δ = -135.34 (d, $^3J_{F\text{-}H}$ = 9.1 Hz) ppm.

^{13}C-NMR (76 MHz, $CDCl_3$) δ = 152.0 (dd, $^1J_{C\text{-}F}$ = 248.4 Hz, $^3J_{C\text{-}F}$ = 6.6 Hz; *C*F), 138.9 (t, $^4J_{C\text{-}F}$ = 2.2 Hz), 133.6 (t, $^3J_{C\text{-}F}$ = 8.1 Hz), 132.2 (t, $^2J_{C\text{-}F}$ = 15.6 Hz; *C*OH), 129.1, 127.9, 126.8, 110 5 – 110.2 (m) ppm.

HRMS (ESI) *m*/*z* $[M\text{-}H]^-$ calculated for $C_{12}H_7F_2O^-$: 205.0470, found: 205.0464.

IR (KBr): ṽ 3166 (br), 3054 (m), 1604 (s), 1536 (s), 1493 (s), 1354 (s), 1260 (s), 1017 (s), 761 (s), 699 (s) cm^{-1}.

UV/Vis (DCM): λ_{max} = 258 nm.

R_f = 0.25 (ethyl acetate/hexane 1:9, *v*/*v*).

m.p.: 71 – 73 °C.

Dibenzyl 3,5-difluoro[1,1'-biphenyl]-4-yl phosphate (**5j**)

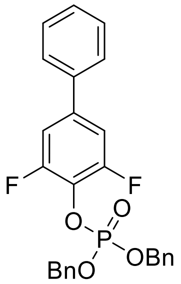

Compound **5j** was synthesized from compound **4j** (80 mg, 0.39 mmol, 1.0 eq.) according to **Method 2C**. The crude product was purified by flash column chromatography (ethyl acetate/hexane 1:4, *v/v*), affording a colorless oil.

Yield: 144 mg (79 %).

^{1}H-NMR (400 MHz, $CDCl_3$) δ = 7.54 – 7.49 (m, 2H), 7.48 – 7.43 (m, 2H), 7.43 – 7.33 (m, 11H), 7.18 (d, $^3J_{H\text{-}F}$ = 9.1 Hz, 2H), 5.26 (d, *J* = 7.6 Hz, 4H; OC*H*$_2$Ph) ppm.

^{19}F-NMR (377 MHz, $CDCl_3$) δ = -126.24 (dd, $^3J_{F\text{-}H}$ = 9.1 Hz, $^4J_{F\text{-}P}$ = 2.8 Hz) ppm.

^{31}P-NMR (162 MHz, $CDCl_3$) δ = -5.81 (t, $^4J_{P\text{-}F}$ = 2.8 Hz) ppm.

^{13}C-NMR (101 MHz, $CDCl_3$) δ = 155.1 (ddd, $^1J_{C\text{-}F}$ = 250.1 Hz, $^3J_{C\text{-}F}$ = 4.2 Hz, $^3J_{C\text{-}P}$ = 4.2 Hz; *C*F), 139.5 (td, $^3J_{C\text{-}F}$ = 8.5 Hz, $^5J_{C\text{-}P}$ = 2.0 Hz), 138.5 (dt, $^4J_{C\text{-}F}$ = 2.3 Hz, $^6J_{C\text{-}P}$ = 1.1 Hz), 135.4

(d, $^{3}J_{C\text{-}P}$ = 7.7 Hz), 129.2, 128.8, 128.7, 128.6, 128.1, 127.0, 126.9 (td, $^{2}J_{C\text{-}F}$ = 15.9 Hz, $^{2}J_{C\text{-}P}$ = 7.7 Hz; *C*OP), 111.2 – 110.8 (m), 70.5 (d, $^{2}J_{C\text{-}P}$ = 5.9 Hz; O*C*H_2Ph) ppm.

HRMS (ESI) *m*/*z* $[M+Na]^+$ calculated for $C_{26}H_{21}F_2O_4PNa^+$: 489.1038, found: 489.1038.

IR (film): $\tilde{\nu}$ 3065 (w), 3035 (w), 2959 (w), 2896 (w), 1601 (m), 1527 (s), 1493 (s), 1297 (s), 1243 (m), 1040 (s), 932 (s), 877 (s), 742 (s), 696 (s) cm^{-1}.

UV/Vis (DCM): λ_{max} = 253 nm.

R_f = 0.25 (ethyl acetate/hexane 1:4, *v*/*v*).

3,5-Difluoro[1,1'-biphenyl]-4-yl dihydrogen phosphate (**6j**)

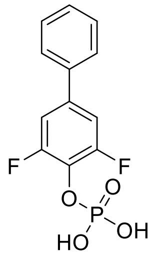

Compound **6j** was prepared from compound **5j** (103 mg, 0.221 mmol) according to **Method 3**. The crude product was purified by C_{18} reversed-phase column chromatography (acetonitrile/water 1:4, *v*/*v*). Lyophilization afforded **6j** as a colorless solid.

Yield: 46 mg (73 %).

^{1}H-NMR (400 MHz, D_2O) δ = 7.66 (d, $^{3}J_{H\text{-}H}$ = 7.6 Hz, 2H), 7.52 (t, $^{3}J_{H\text{-}H}$ = 7.4 Hz, 2H), 7.45 (t, $^{3}J_{H\text{-}H}$ = 7.3 Hz, 1H), 7.35 (d, $^{3}J_{H\text{-}F}$ = 9.3 Hz, 2H) ppm.

^{19}F-NMR (377 MHz, D_2O) δ = -127.38 (dd, $^{3}J_{F\text{-}H}$ = 9.1 Hz, $^{4}J_{F\text{-}P}$ = 2.0 Hz) ppm.

^{31}P-NMR (162 MHz, D_2O) δ = 2.81 – -2.94 (m) ppm.

^{13}C-NMR (101 MHz, D_2O) δ = 155.4 (ddd, $^{1}J_{C\text{-}F}$ = 246.8 Hz, $^{3}J_{C\text{-}F}$ / $^{3}J_{C\text{-}P}$ = 5.4, 3.4 Hz; *C*F), 143.9, 138.3 (t, $^{3}J_{C\text{-}F}$ = 1.9 Hz), 137.5 – 137.2 (m; *C*OP), 129.2, 128.4, 126.8, 110.7 – 110.4 (m) ppm.

HRMS (ESI) *m*/*z* $[M-H]^-$ calculated for $C_{12}H_8F_2O_4P^-$: 285.0134, found: 285.0135.

IR (KBr): $\tilde{\nu}$ 3434 (br), 2925 (w), 1595 (w), 1533 (w), 1494 (w), 1263 (w), 1037 (w), 920 (w), 858 (w), 760 (w), 696 (w) cm^{-1}.

UV/Vis (H_2O): λ_{max} = 200; 253 nm.

R_f = 0.29 (acetonitrile/water 1:4, *v*/*v*).

m.p.: 168 – 171°C.

3-Chloro[1,1'-biphenyl]-4-ol (**4k**)

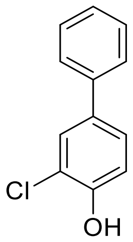

A mixture of 4-bromo-2-chlorophenol (**3f**, 104 mg, 0.500 mmol, 1.0 eq.), phenylboronic acid (**2f**, 122 mg, 1.00 mmol, 2.0 eq.), $Pd(PPh_3)_4$ (35 mg, 0.030 mmol, 6.0 mol%), and 1.5 mL of a degassed aqueous 1 M Na_2CO_3 solution in 2 mL of dry and degassed dimethoxyethane was stirred at 80 °C for 16 h. After being cooled to room temperature, the mixture was transferred to 25 mL of a 1 M NH_4Cl solution and extracted three times with ethyl acetate. The combined organic phases were dried over Na_2SO_4, and the solvents were removed under reduced pressure. The resulting concentrate was chromatographed on silica gel (ethyl acetate/hexane 1:4, *v*/*v*) to give **4k** as a colorless solid.

Yield: 58 mg (57 %).

^{1}H-NMR (400 MHz, $CDCl_3$) δ = 7.56 (d, *J* = 2.2 Hz, 1H), 7.54 – 7.50 (m, 2H), 7.46 – 7.39 (m, 3H), 7.37 – 7.30 (m, 1H), 7.09 (d, *J* = 8.4 Hz, 1H), 5.63 (s, 1H; O*H*) ppm.

^{13}C-NMR (76 MHz, $CDCl_3$) δ = 150.9 (*C*OH), 139.7, 135.1, 129.0, 127.6, 127.4, 127.3, 126.8, 120.4, 116.6 ppm.

HRMS (ESI) *m*/*z* $[M-H]^-$ calculated for $C_{12}H_8ClO^-$: 203.0269, found: 203.0271.

IR (KBr): ṽ 3334 (br), 3055 (w), 3034 (w), 1606 (m), 1516 (s), 1490 (s), 1280 (s), 1231 (s), 763 (s), 742 (s), 698 (s) cm^{-1}.

UV/Vis ($CHCl_3$): λ_{max} = 260 nm.

R_f = 0.35 (ethyl acetate/hexane 1:4, *v*/*v*).

m.p.: 65 – 68 °C (lit. 61 – 63 °C).[11]

Dibenzyl 3-chloro[1,1'-biphenyl]-4-yl phosphate (**5k**)

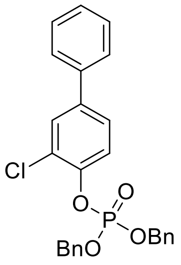

Compound **5k** was prepared from phenol **4k** (38 mg, 0.19 mmol, 1.0 eq.) according to **Method 2C**. The crude product was purified by flash column chromatography (ethyl acetate/hexane 1:4, *v*/*v*) to give a colorless oil.

Yield: 50 mg (57 %).

^{1}H-NMR (400 MHz, $CDCl_3$) δ = 7.60 (s, 1H), 7.54 – 7.49 (m, 2H), 7.47 – 7.41 (m, 3H), 7.39 (s, 1H), 7.37 – 7.32 (m, 11H), 5.21 (d, *J* = 8.3 Hz, 4H; OCH_2Ph) ppm.
^{31}P-NMR (162 MHz, $CDCl_3$) δ = -6.64 (s) ppm.
^{13}C-NMR (101 MHz, $CDCl_3$) δ = 145.99 (d, $^{2}J_{C\text{-}P}$ = 6.2 Hz; *C*OP), 145.96, 139.5, 139.2, 135.5 (d, $^{3}J_{C\text{-}P}$ = 7.0 Hz), 135.4, 129.2, 129.1, 128.82, 128.75, 128.2, 128.0, 127.1, 126.6, 125.9, 125.8, 121.8, 70.5 (d, $^{2}J_{C\text{-}P}$ = 6.1 Hz; OCH_2Ph) ppm.
HRMS (ESI) *m*/*z* $[M+Na]^+$ calculated for $C_{26}H_{22}ClO_4PNa^+$: 487.0836, found: 487.0842.
R_f = 0.33 (ethyl acetate/hexane 1:4, *v*/*v*).

3-Chloro[1,1'-biphenyl]-4-yl dihydrogen phosphate (**6k**)

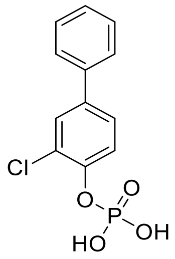

Compound **6k** was prepared from compound **5k** (46 mg, 0.10 mmol) according to **Method 3**. The crude product was purified by C_{18} reversed-phase column chromatography (acetonitrile/water 1:4, *v*/*v*). Lyophilization afforded **6k** as a colorless solid.
Yield: 6.0 mg (21 %).
^{1}H-NMR (400 MHz, D_2O) δ = 7.82 (d, *J* = 2.2 Hz, 1H), 7.71 (d, *J* = 7.4 Hz, 2H), 7.61 (dd, *J* = 8.6, 2.5 Hz, 1H), 7.58 – 7.49 (m, 3H), 7.49 – 7.43 (m, 1H) ppm.
^{31}P-NMR (162 MHz, D_2O) δ = -3.12 (s) ppm.
^{13}C-NMR (101 MHz, D_2O) δ = 147.8 (d, $^{2}J_{C\text{-}P}$ = 6.3 Hz), 139.0, 137.3 (d, $^{3}J_{C\text{-}P}$ = 4.1 Hz), 129.2 (d, $^{4}J_{C\text{-}P}$ = 2.0 Hz), 128.5 (d, $^{6}J_{C\text{-}P}$ = 0.9 Hz), 127.9 (d, $^{5}J_{C\text{-}P}$ = 1.7 Hz), 126.8 (d, $^{4}J_{C\text{-}P}$ = 2.0 Hz), 126.4, 125.8 (d, $^{3}J_{C\text{-}P}$ = 5.6 Hz), 121.9 ppm.
HRMS (ESI) *m*/*z* $[M-H]^-$ calculated for $C_{12}H_9ClO_4P^-$: 282.9932, found: 282.9926.
IR (KBr): $\tilde{\nu}$ 3124 (br), 3031 (w), 1636 (w), 1513 (w), 1439 (m), 1279 (m), 1097 (w), 1061 (m), 929 (w), 817 (w), 759 (m), 697 (m) cm^{-1}.
UV/Vis (H_2O): λ_{max} = 241; 272 nm.
R_f = 0.26 (acetonitrile/water 1:4, *v*/*v*).
m.p.: 180 – 182 °C.

3-Methoxy[1,1'-biphenyl]-4-ol (**4l**)

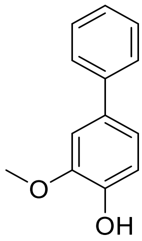

Compound **4l** was synthesized from 4-bromo-2-methoxyphenol (**3g**, 203 mg, 1.00 mmol, 1.0 eq.), phenylboronic acid (**2f**, 183 mg, 1.50 mmol, 1.5 eq.), K_3PO_4 (424 mg, 2.00 mmol, 2.0 eq.), $Pd(OAc)_2$ (6.7 mg, 0.030 mmol, 3.0 mol%), and SPhos (25 mg, 0.060 mmol, L:Pd=2:1) in 2 mL of dry and degassed toluene according to **Method 1A**. The crude product was purified by flash column chromatography (ethyl acetate/hexane 1:9, *v*/*v*) to give **4l** as a colorless solid.

Yield: 120 mg (60 %).

^{1}H-NMR (400 MHz, $CDCl_3$) δ = 7.58 – 7.52 (m, 2H), 7.46 – 7.38 (m, 2H), 7.35 – 7.28 (m, 1H), 7.14 – 7.07 (m, 2H), 6.99 (d, *J* = 8.1 Hz, 1H), 5.63 (s, 1H; O*H*), 3.96 (s, 3H; OCH_3) ppm.

^{13}C-NMR (76 MHz, $CDCl_3$) δ = 146.9, 145.4, 141.4, 133.9, 128.9, 127.0, 126.9, 120.3, 114.8, 109.9, 56.1 (O*C*H_3) ppm.

HRMS (ESI) *m*/*z* $[M-H]^-$ calculated for $C_{13}H_{11}O_2^-$: 199.0765, found: 199.0763.

IR (KBr): ṽ 3512 (br), 2940 (w), 2843 (w), 1604 (w), 1521 (m), 1488 (m), 1255 (m), 1208 (m), 760 (m), 699 (m) cm^{-1}.

UV/Vis ($CHCl_3$): λ_{max} = 265; 285 nm.

R_f = 0.24 (ethyl acetate/hexane 1:9, *v*/*v*).

m.p.: 73 – 76 °C (lit. 74 – 75 °C).[12]

Dibenzyl 3-methoxy[1,1'-biphenyl]-4-yl phosphate (**5l**)

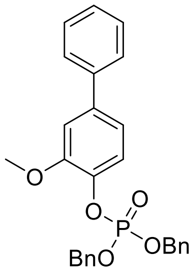

Compound **5l** was synthesized from compound **4l** (60 mg, 0.30 mmol, 1.0 eq.) according to **Method 2C**. The crude product was purified by flash column chromatography (ethyl acetate/hexane 1:4, *v*/*v*) to afford a colorless oil.

Yield: 97 mg (70 %).

^{1}H-NMR (400 MHz, $CDCl_3$) δ = 7.56 – 7.51 (m, 2H), 7.47 – 7.41 (m, 2H), 7.38 – 7.31 (m, 12H), 7.12 – 7.05 (m, 2H), 5.20 (d, *J* = 7.8 Hz, 4H; OCH_2Ph), 3.85 (s, 3H; OCH_3) ppm.

^{31}P-NMR (162 MHz, $CDCl_3$) δ = -5.77 (s) ppm.

^{13}C-NMR (101 MHz, $CDCl_3$) δ = 150.8 (d, $^3J_{C\text{-}P}$ = 5.9 Hz; *C*OCH$_3$), 140.7, 139.4, 139.2 (d, $^2J_{C\text{-}P}$ = 7.1 Hz; *C*OP), 135.8 (d, $^3J_{C\text{-}P}$ = 7.1 Hz), 128.8, 128.52, 128.47, 127.9, 127.4, 127.1, 121.7 (d, $^3J_{C\text{-}P}$ = 3.4 Hz), 119.5, 111.7, 69.9 (d, $^2J_{C\text{-}P}$ = 5.8 Hz; OC*H*$_2$Ph), 55.9 (O*C*H$_3$) ppm.
HRMS (ESI) *m*/*z* $[M+H]^+$ calculated for $C_{27}H_{26}O_5P^+$: 461.1512, found: 461.1508.
IR (film): ṽ 3011 (m), 2962 (w), 1600 (w), 1519 (m), 1489 (m), 1281 (m), 1238 (m), 1216 (s), 1019 (s), 955 (m), 754 (s), 697 (m) cm^{-1}.
UV/Vis ($CHCl_3$): λ_{max} = 255; 282 nm.
R$_f$ = 0.20 (ethyl acetate/hexane 1:4, *v*/*v*).

<u>3-Methoxy[1,1'-biphenyl]-4-yl dihydrogen phosphate (**6l**)</u>

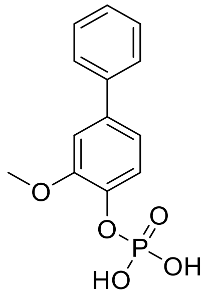

Compound **6l** was prepared from compound **5l** (49 mg, 0.11 mmol) according to **Method 3**. The crude product was purified by C_{18} reversed-phase column chromatography (acetonitrile/water 1:4, *v*/*v*). Lyophilization afforded **6l** as a colorless solid.
Yield: 25 mg (85 %).
^{1}H-NMR (400 MHz, D_2O) δ = 7.75 – 7.71 (m, 2H), 7.57 – 7.51 (m, 2H), 7.48 – 7.41 (m, 2H), 7.37 (d, *J* = 1.6 Hz, 1H), 7.29 (dd, *J* = 8.3, 2.2 Hz, 1H), 3.96 (s, 3H; OC*H*$_3$) ppm.
^{31}P-NMR (162 MHz, D_2O) δ = -2.14 (s) ppm.
^{13}C-NMR (101 MHz, D_2O) δ = 150.5 (d, $^3J_{C\text{-}P}$ = 5.7 Hz; *C*OCH$_3$), 141.7 (d, $^2J_{C\text{-}P}$ = 6.5 Hz; *C*OP), 140.2, 136.7, 129.1, 127.6, 126.9, 121.2 (d, $^3J_{C\text{-}P}$ = 2.3 Hz), 119.7, 111.9, 56.2 (O*C*H$_3$) ppm.
HRMS (ESI) *m*/*z* $[M-H]^-$ calculated for $C_{13}H_{13}O_5P^-$: 279.0428, found: 279.0434.
IR (KBr): ṽ 3446 (br), 2927 (w), 1636 (w), 1521 (m), 1490 (m), 1250 (w), 1211 (w), 1020 (w), 929 (w), 760 (m), 696 (m) cm^{-1}.
UV/Vis (H_2O): λ_{max} = 204; 256; 281 nm.
R$_f$ = 0.35 (acetonitrile/water 1/4, *v*/*v*).
m.p.: 172 – 174 °C.

2-Fluoro-4-(naphthalen-2-yl)phenol (**4m**)

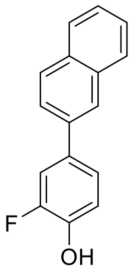

$Pd(PPh_3)_4$ (18 mg, 0.016 mmol, 3.0 mol%) was suspended in degassed 1,4-dioxane under an argon atmosphere. Next, 0.52 mL of a degassed aqueous 2 M Na_2CO_3 solution, 4-bromo-2-fluorophenol (**3c**, 0.0600 mL, 105 mg, 0.550 mmol, 1.0 eq.), and 2-naphthylboronic acid (**2e**, 100 mg, 0.580 mmol, 1.1 eq.) were added to the mixture, which was stirred at 90 °C for 24 h. The solvent was then evaporated, and the residue was taken up in water and extracted with ethyl acetate. The combined organic phases were dried over Na_2SO_4, before being filtered and evaporated, and the crude product was purified by flash column chromatography (ethyl acetate/hexane 1:3, *v/v*) to afford **4m** as a colorless solid.

Yield: 80 mg (64 %).

^{1}H-NMR (400 MHz, $CDCl_3$) δ = 7.97 (d, *J* = 1.8 Hz, 1H), 7.92 – 7.84 (m, 3H), 7.67 (dd, *J* = 8.5 Hz, 1.9, 1H), 7.54 – 7.38 (m, 4H), 7.12 (t, *J* = 8.7 Hz, 1H), 5.24 – 5.14 (m, 1H; O*H*) ppm.

^{19}F-NMR (377 MHz, D_2O) δ = -140.57 – -140.70 (m) ppm.

^{13}C-NMR (101 MHz, $CDCl_3$) δ = 151.3 (d, $^1J_{C\text{-}F}$ = 237.3 Hz; *C*F), 143.0 (d, $^2J_{C\text{-}F}$ = 14.4 Hz; *C*OH), 137.1 (d, $^4J_{C\text{-}F}$ = 2.0 Hz), 134.4 (d, $^3J_{C\text{-}F}$ = 6.2 Hz), 133.6, 132.5, 128.5, 128.1, 127.6, 126.4, 126.0, 125.3, 125.1, 123.7 (d, $^3J_{C\text{-}F}$ = 3.2 Hz), 117.6 (d, $^4J_{C\text{-}F}$ = 2.3 Hz), 114.4 (d, $^2J_{C\text{-}F}$ = 18.8 Hz) ppm.

HRMS (ESI) *m/z* $[M\text{-}H]^-$ calculated for $C_{16}H_{10}FO^-$: 237.0721, found: 237.0725.

IR (KBr): ṽ 3235 (br), 3055 (w), 1627 (m), 1601 (m), 1527 (s), 1504 (m), 1307 (s), 1117 (m), 857 (m), 810 (s), 742 (m) cm^{-1}.

UV/Vis (DCM): λ_{max} = 230; 258; 292 nm.

R_f = 0.45 (ethyl acetate/hexane 1:3, *v*/*v*).

m.p.: 122 °C (lit. 110 – 112 °C).[14]

Dibenzyl 2-fluoro-4-(naphthalen-2-yl)phenyl phosphate (**5m**)

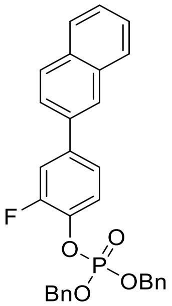

Compound **5m** was prepared from compound **4m** (70 mg, 0.29 mmol, 1.0 eq.) according to **Method 2B**. The crude product was purified by flash column chromatography (ethyl acetate/hexane 1:3, *v/v*) to give a colorless oil.

Yield: 107 mg (75 %).

^{1}H-NMR (400 MHz, $CDCl_3$) δ = 7.97 (d, *J* = 1.8 Hz, 1H), 7.94 – 7.85 (m, 3H), 7.65 (dd, *J* = 8.5, 1.9 Hz, 1H), 7.55 – 7.50 (m, 2H), 7.50 – 7.43 (m, 1H), 7.41 – 7.31 (m, 12H), 5.24 – 5.18 (m, 4H; OCH_2Ph) ppm.

^{19}F-NMR (377 MHz, $CDCl_3$) δ = -130.12 – -130.25 (m) ppm.

^{31}P-NMR (162 MHz, $CDCl_3$) δ = -6.03 (s) ppm.

^{13}C-NMR (101 MHz, $CDCl_3$) δ = 153.8 (dd, $^{1}J_{C\text{-}F}$ = 249.1 Hz, $^{3}J_{C\text{-}P}$ = 5.8 Hz; *C*F), 139.7 (d, $^{3}J_{C\text{-}F}$ = 6.6 Hz), 137.6 (dd, $^{2}J_{C\text{-}F}$ = 12.4 Hz, $^{2}J_{C\text{-}P}$ = 6.9 Hz; *C*OP), 136.7 – 136.6 (m), 135.5 (d, $^{3}J_{C\text{-}P}$ = 6.8 Hz), 133.7, 132.9, 128.84, 128.76, 128.4, 128.2, 127.8, 126.7, 126.5, 126.0, 125.2, 123.4 – 123.3 (m), 122.8 (d, $^{4}J_{C\text{-}F}$ = 3.0 Hz), 115.9 (d, $^{2}J_{C\text{-}F}$ = 19.1 Hz), 70.4 (d, $^{2}J_{C\text{-}P}$ = 5.9 Hz; OCH_2Ph) ppm.

HRMS (ESI) *m/z* [M+Na]$^{+}$ calculated for $C_{30}H_{24}FO_4PNa^{+}$: 521.1288, found: 521.1291.

IR (KBr): ṽ 3064 (w), 3035 (w), 2957 (w), 2898 (w), 1589 (m), 1522 (s), 1502 (s), 1274 (s), 1216 (m), 1055 (s), 1022 (s), 998 (s), 859 (s), 811 (s), 748 (s), 696 (s) cm^{-1}.

UV/Vis (DCM): λ_{max} = 228; 253; 290 nm.

R$_f$ = 0.36 (ethyl acetate/hexane 1:3, *v/v*).

2-Fluoro-4-(naphthalen-2-yl)phenyl dihydrogen phosphate (**6m**)

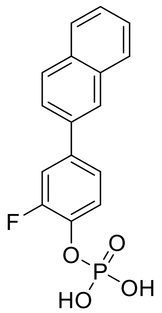

Compound **6m** was prepared as an off-white powder from compound **5m** (100 mg, 0.201 mmol) according to **Method 3**.

Yield: 52 mg (81 %).

^{1}H-NMR (400 MHz, DMSO-d$_6$) δ = 8.25 (d, *J* = 1.8, 1H), 8.04 – 7.91 (m, 3H), 7.86 (dd, *J* = 8.6, 1.9 Hz, 1H), 7.79 (dd, *J* = 12.2, 2.2 Hz, 1H), 7.67 – 7.62 (m, 1H), 7.58 – 7.48 (m, 3H) ppm.

^{19}F-NMR (377 MHz, DMSO-d$_6$) δ = -130.56 – -130.67 (m) ppm.

^{31}P-NMR (162 MHz, DMSO-d$_6$) δ = -5.88 (s) ppm.

^{13}C-NMR (101 MHz, DMSO-d$_6$) δ = 154.7 – 152.0 (m; *C*F), 141.2 – 140.8 (m; *C*OP), 136.2 – 136.1 (m), 134.6 (d, $^{3}J_{C\text{-}F}$ = 5.7 Hz), 133.3, 132.1, 128.4, 128.1, 127.5, 126.4, 126.1, 124.9, 124.8, 122.6 – 122.3 (m), 122.5 – 122.4 (m), 114.3 (d, $^{2}J_{C\text{-}F}$ = 19.6 Hz) ppm.

HRMS (ESI) *m*/*z* $[M-H]^-$ calculated for $C_{16}H_{11}FO_4P^-$: 317.0384, found: 317.0380.

IR (KBr): $\tilde{\nu}$ 3443 (br), 1619 (m), 1523 (m), 1230 (w), 1162 (m), 1013 (w), 990 (w), 814 (w), 768 (w) cm^{-1}.

UV/Vis (MeOH): λ_{max} = 215; 254; 292 nm.

m.p.: 179 °C.

Diethyl (4-bromo-2-fluorobenzyl)phosphonate (**7b**)

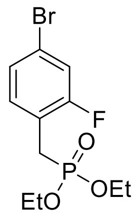

Under inert gas atmosphere and neat conditions, 4-bromo-1-(bromomethyl)-2-fluorobenzene (603 mg, 2.25 mmol, 1.0 eq.) was stirred with triethyl phosphite (779 µL, 748 mg, 4.40 mmol, 2.0 eq.) at 120 °C for 2 h. The reaction mixture was cooled and diluted with acetone, and the volatile components were eliminated *in vacuo*. The resulting residue was purified by flash column chromatography (hexane to ethyl acetate/hexane 7:3, *v*/*v*) to afford **7b** as a colorless oil.

Yield: 615 mg (84 %).

^{1}H-NMR (400 MHz, $(CD_3)_2CO$) δ = 7.42 – 7.34 (m, 3H), 4.03 (dq, *J* = 8.4, 7.1 Hz, 4H; CH_2CH_3), 3.18 (dd, *J* = 21.7, 1.2 Hz, 2H; CH_2P), 1.22 (td, *J* = 7.0, 0.4 Hz, 6H; CH_2CH_3) ppm.

^{19}F-NMR (377 MHz, $(CD_3)_2CO$) δ = -116.12 – -116.24 (m) ppm.

^{31}P-NMR (162 MHz, $(CD_3)_2CO$) δ = 22.66 (d, $^4J_{P\text{-}F}$ = 4.6 Hz) ppm.

^{13}C-NMR (101 MHz, $(CD_3)_2CO$) δ = 161.6 (dd, $^1J_{C\text{-}F}$ = 250.1 Hz, $^3J_{C\text{-}P}$ = 7.3 Hz; *C*F), 134.4 (dd, $^3J_{C\text{-}F}$ / $^3J_{C\text{-}P}$ = 5.3, 4.4 Hz), 128.3 – 128.2 (m), 121.1 (dd, $^3J_{C\text{-}F}$ = 9.6 Hz, $^5J_{C\text{-}P}$ = 4.5 Hz), 120.6 (dd, $^2J_{C\text{-}F}$= 15.8 Hz, $^2J_{C\text{-}P}$ = 9.3 Hz), 119.5 (dd, $^2J_{C\text{-}F}$ = 25.8 Hz, $^4J_{C\text{-}P}$ = 3.0 Hz), 62.6 (d, $^2J_{C\text{-}P}$ = 6.5 Hz; CH_2CH_3), 26.4 (dd, $^1J_{C\text{-}P}$ = 139.4 Hz, $^3J_{C\text{-}F}$ = 2.6 Hz; CH_2P), 16.7 (d, $^3J_{C\text{-}P}$ = 5.9 Hz; CH_2CH_3) ppm.

HRMS (ESI) *m*/*z* $[M+H]^+$ calculated for $C_{11}H_{16}BrFO_3P^+$: 324.9999, found: 324.9998.

IR (film): $\tilde{\nu}$ 2983 (w), 2928 (w), 2907 (w), 1606 (w), 1578 (w), 1488 (s), 1273 (m), 1255 (m), 1223 (m), 1053 (s), 1027 (s), 967 (m), 887 (m) cm^{-1}.

UV/Vis (DCM): λ_{max} = 236; 270; 276 nm.

R_f = 0.35 (ethyl acetate/hexane 7:3, *v*/*v*).

Diethyl [2-fluoro-4-(naphthalen-2-yl)benzyl]phosphonate (**7c**)

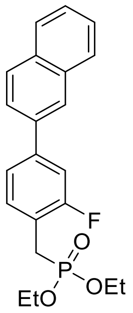

A *Schlenk* flask was charged with **7b** (152 mg, 0.468 mmol, 1.0 eq.), naphthalen-2-ylboronic acid (163 mg, 0.948 mmol, 2.0 eq.), Cs_2CO_3 (458 mg, 1.41 mmol, 3.0 eq.), SPhos (20 mg, 0.049 mmol, L:Pd=2:1), and 2 mL of dry toluene. The suspension was degassed three times by the freeze-pump-thaw method, before $Pd(PPh_3)_4$ (29 mg, 0.025 mmol, 5.4 mol%) was introduced, and the mixture was stirred at 80 °C for 20 h. The reaction was then cooled, diluted with acetone, and the catalyst was removed by filtration through a thin layer of silica gel. The solvents were evaporated under reduced pressure, and the crude product was purified by two successive flash column chromatographies (ethyl acetate/hexane 3:7 (*v*/*v*) to 100 % ethyl acetate, then dichloromethane to methanol/dichloromethane 1:99, *v*/*v*) to afford **7c** as a colorless solid.

Yield: 163 mg (94 %).

^{1}H-NMR (400 MHz, $CDCl_3$) δ = 8.04 – 8.01 (m, 1H), 7.94 – 7.84 (m, 3H), 7.71 (dd, *J* = 8.5, 1.9 Hz, 1H), 7.55 – 7.40 (m, 5H), 4.15 – 4.06 (m, 4H; CH_2CH_3), 3.26 (dd, *J* = 21.6, 1.0 Hz, 2H; CH_2P), 1.30 (td, *J* = 7.0, 0.5 Hz, 6H; CH_2CH_3) ppm.

^{19}F-NMR (376 MHz, $CDCl_3$) δ = -117.08 – -117.20 (m) ppm.

^{31}P-NMR (162 MHz, $CDCl_3$) δ = 25.11 (d, $^4J_{P\text{-}F}$ = 4.6 Hz) ppm.

^{13}C-NMR (75 MHz, $CDCl_3$) δ = 161.3 (dd, $^1J_{C\text{-}F}$ = 246.5 Hz, $^3J_{C\text{-}P}$ = 7.4 Hz; *C*F), 142.2 (dd, $^3J_{C\text{-}F}$ = 7.9 Hz, $^5J_{C\text{-}P}$ = 3.8 Hz), 137.1 – 136.9 (m), 133.7, 133.0, 132.3 (dd, $^3J_{C\text{-}F}$ / $^3J_{C\text{-}P}$ = 5.3, 4.2 Hz), 128.8, 128.4, 127.8, 126.6, 126.4, 126.0 (d, $^5J_{C\text{-}F}$ = 1.2 Hz), 125.2 (d, $^5J_{C\text{-}F}$ = 0.7 Hz), 123.2 – 123.1 (m), 118.2 (dd, $^2J_{C\text{-}F}$ = 16.0 Hz, $^2J_{C\text{-}P}$ = 9.5 Hz), 114.3 (dd, $^2J_{C\text{-}F}$ = 23.2 Hz, $^4J_{C\text{-}P}$ = 3.3 Hz), 62.4 (d, $^2J_{C\text{-}P}$ = 6.7 Hz; CH_2CH_3), 26.3 (dd, $^1J_{C\text{-}P}$ = 140.4 Hz, $^3J_{C\text{-}F}$ = 2.8 Hz; CH_2P), 16.5 (d, $^3J_{C\text{-}P}$ = 6.1 Hz; CH_2CH_3) ppm.

HRMS (ESI) *m*/*z* $[M+H]^+$ calculated for $C_{21}H_{23}FO_3P^+$: 373.1363, found: 373.1364.

IR (KBr): ṽ 3059 (w), 2983 (w), 2916 (w), 1626 (w), 1599 (w), 1565 (w), 1500 (w), 1279 (m), 1252 (m), 1227 (m), 1053 (s), 1021 (s), 967 (s), 818 (s), 749 (m) cm^{-1}.

UV/Vis (DCM): λ_{max} = 256; 292 nm.

R_f = 0.25 (ethyl acetate/hexane 7:3, *v*/*v*).

m.p.: 62 – 64 °C.

[2-Fluoro-4-(naphth-alen2-yl)benzyl]phosphonic acid (**7**)

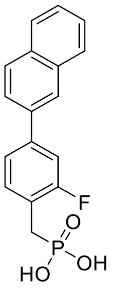

Compound **7** was synthesized from compound **7c** according to **Method 4.** Compound **7c** (110 mg, 0.295 mmol, 1.0 eq.) was stirred with TMS-Br (0.548 mL, 635 mg, 4.15 mmol, 14 eq.) in 4.6 mL of dry DCM for 2 h at 0 °C and then for 18°h at room temperature. The crude product was purified by C_{18} reversed-phase column chromatography (water to acetonitrile/water 1:1, *v*/*v*). Lyophilization afforded **7** as a colorless solid.

Yield: 75 mg (80 %).

^{1}H-NMR (400 MHz, DMSO-d_6) δ = 8.16 (d, *J* = 1.9 Hz, 1H), 8.00 – 7.88 (m, 3H), 7.79 (dd, *J* = 8.6, 1.9 Hz, 1H), 7.60 – 7.42 (m, 5H), 2.93 – 2.78 (m, 2H; CH_2P) ppm.

^{19}F-NMR (377 MHz, DMSO-d_6) δ = -117.49 – -117.63 (m) ppm.

^{31}P-NMR (162 MHz, DMSO-d_6) δ = 16.92 (bs) ppm.

^{13}C-NMR (75 MHz, DMSO-d_6) δ = 160.8 (dd, $^{1}J_{C-F}$ = 244.6 Hz, $^{3}J_{C-P}$ = 6.9 Hz; *C*F), 140.1 (dd, $^{3}J_{C-F}$ = 8.0 Hz, $^{5}J_{C-P}$ = 3.6 Hz), 136.1 – 136.0 (m), 133.3, 132.6 – 132.4 (m), 132.4, 128.5, 128.3, 127.5, 126.5, 126.3, 125.3, 124.8, 122.5 – 122.3 (m), 120.7 (dd, $^{2}J_{C-F}$ = 16.1 Hz, $^{2}J_{C-P}$ = 9.0 Hz), 113.3 (dd, $^{2}J_{C-F}$ = 23.9 Hz, $^{4}J_{C-P}$ = 2.1 Hz), 29.1 – 27.1 (m; *C*H_2P) ppm.

HRMS (ESI) *m*/*z* $[M-H]^-$ calculated for $C_{17}H_{13}FO_3P^-$: 315.0592, found: 315.0588:

IR (KBr): ṽ 3127 (br), 3059 (w), 3022 (w), 2923 (w), 2852 (w), 1632 (m), 1600 (m), 1565 (m), 1503 (m), 1402 (s), 1272 (m), 1033 (m), 917 (m), 816 (m), 743 (m) cm^{-1}.

UV/Vis (H_2O): λ_{max} = 291; 255; 215 nm.

R_f = 0.48 (acetonitrile/water 1:1, *v*/*v*).

m.p.: decomposition at 228 °C.

[(Diethoxyphosphoryl)(difluoro)methyl]zinc bromide (**11b**)

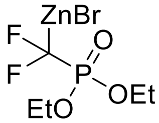

Zinc powder was stirred with an aqueous solution of 3 M HCl to activate it. The powder was then washed with water, methanol, and diethyl ether and dried under high vacuum overnight. Under inert gas atmosphere, the activated zinc dust (2.03 g, 30.8 mmol, 1.0 eq.) was suspended in 25 mL of dry THF and heated to 50 °C for 30 min. The zinc suspension was cooled to room temperature and sonicated for 20 min. Diethyl [bromo(difluoro)-

methyl]phosphonate (5.5 mL, 8.27 g, 31.0 mmol, 1.0 eq.) was then slowly added, during which the exothermic reaction turned clear. The mixture was stirred for a further 15 min at room temperature and then for 20 h at 50 °C. Once cool, it was used directly in the next step, without any further purification or characterization.

Diethyl [(4-bromo-2-fluorophenyl)(difluoro)methyl]phosphonate (**11c**)

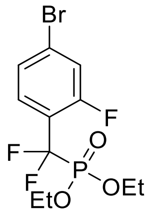

Copper(I) bromide (4.44 g, 31.0 mmol, 5.0 eq.) was stirred with the mixture containing the zinc reagent **11b** (maximum of 10.3 g, 31.0 mmol, 5.0 eq.) at room temperature for 30 min. 4-bromo-2-fluoro-1-iodobenzene (1.86 g, 6.18 mmol, 1.0 eq.) dissolved in 6.3 mL of dry THF was then transferred to the resulting suspension, which was stirred again at 45 °C for 20 h. Once cool, ethyl acetate and saturated NH_4Cl solution were introduced. The organic and aqueous phases were separated, and the aqueous phase was extracted three times with ethyl acetate. The combined organic phases were washed with brine, dried over Na_2SO_4, filtered, and evaporated *in vacuo*. The crude product was purified by flash column chromatography (hexane to acetone/hexane 1:9, *v*/*v*) to afford **11c** as a colorless oil.

Yield: 1.33 g (60 %).

^{1}H-NMR (400 MHz, $CDCl_3$) δ = 7.45 – 7.31 (m, 3H), 4.33 – 4.18 (m, 4H; CH_2CH_3), 1.34 (td, J = 7.1, 0.7 Hz, 6H; CH_2CH_3) ppm.

^{19}F-NMR (376 MHz, $CDCl_3$) δ = -107.66 (dd, $^2J_{F\text{-}P}$ = 112.8 Hz, $^4J_{F\text{-}F}$ = 19.3 Hz, 2F; CF_2), -109.43 – -109.64 (m, 1F; CF) ppm.

^{31}P-NMR (162 MHz, $CDCl_3$) δ = 4.83 (td, $^2J_{P\text{-}F}$ = 112.8 Hz, $^4J_{P\text{-}F}$ = 2.8 Hz) ppm.

^{13}C-NMR (101 MHz, $CDCl_3$) δ = 159.8 (dtd, $^1J_{C\text{-}F}$ = 260.3 Hz, $^3J_{C\text{-}F}$ = 3.5 Hz, $^3J_{C\text{-}P}$ = 2.4 Hz; *C*F), 129.8 (tdd, $^3J_{C\text{-}F}$ = 7.9 Hz, $^3J_{C\text{-}F}$ = 2.3 Hz, $^3J_{C\text{-}P}$ = 2.3 Hz), 127.7 (dd, $^4J_{C\text{-}F}$ = 3.9 Hz, $^4J_{C\text{-}P}$ = 1.3 Hz), 126.1 (dtd, $^3J_{C\text{-}F}$ = 9.1 Hz, $^5J_{C\text{-}F}$ = 2.2 Hz, $^5J_{C\text{-}P}$ = 2.0 Hz), 120.7 (dd, $^2J_{C\text{-}F}$ = 24.9 Hz, $^4J_{C\text{-}P}$ = 1.3 Hz), 119.7 (tdd, $^2J_{C\text{-}F}$ = 20.5 Hz, $^2J_{C\text{-}F}$ / $^2J_{C\text{-}P}$ = 13.5, 11.4 Hz), 116.8 (tdd, $^1J_{C\text{-}F}$ = 264.4 Hz, $^1J_{C\text{-}P}$ = 221.2 Hz, $^3J_{C\text{-}F}$ = 2.5 Hz; CF_2), 65.3 (d, $^2J_{C\text{-}P}$ = 6.8 Hz; CH_2CH_3), 16.5 (d, $^3J_{C\text{-}P}$ = 5.6 Hz; CH_2CH_3) ppm.

HRMS (ESI) *m*/*z* $[M+H]^+$ calculated for $C_{11}H_{14}BrF_3O_3P^+$: 360.9811, found: 360.9811.

IR (film): ṽ 2986 (m), 2936 (w), 2913 (w), 2870 (w), 2853 (w), 1606 (s), 1576 (m), 1487 (m), 1406 (m), 1273 (s), 1227 (m), 1061 (s), 1043 (s), 1021 (s), 875 (m) cm^{-1}.

UV/Vis ($CHCl_3$): λ_{max} = 276; 269; 241 nm.

R_f = 0.35 (acetone/hexane 1:4, *v*/*v*).

4,4,4',4',5,5,5',5'-Octaethyl-2,2'-bis(1,3,2-dioxaborolane) (**12b**)

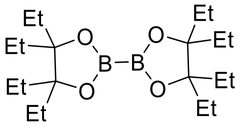

Synthesis of compound **12b** was essentially done as described in the literature.[15] Using a *Dean-Stark* apparatus, a mixture of tetrahydroxydiboron (139 mg, 1.55 mmol, 1.0 eq.), ethyl pinacol (540 mg, 3.10 mmol, 2.0 eq.), KOAc (401 mg, 4.09 mmol, 2.6 eq.), and 15 mL of dry toluene was refluxed for 17 h under inert gas atmosphere. Once at room temperature, the reaction mixture was evaporated *in vacuo*, and the crude product was purified by flash column chromatography (hexane to ethyl acetate/hexane 1:9, *v*/*v*) to afford **12b** as a colorless solid.

Yield: 452 mg (80 %).

^{1}H-NMR (300 MHz, $CDCl_3$) δ = 1.78 – 1.54 (m, 16H; C$H_2$$CH_3$), 0.90 (t, *J* = 7.5 Hz, 24H; CH_2CH_3) ppm.

^{13}C-NMR (75 MHz, $CDCl_3$) δ = 88.5 (O*C*), 26.6 (*C*$H_2$$CH_3$), 9.1 ($CH_2$*C*$H_3$) ppm.

HRMS (ESI) *m*/*z* $[M+H]^+$ calculated for $C_{20}H_{41}B_2O_4^+$: 367.3185, found: 367.3187.

IR (KBr): ṽ 2975 (s), 2948 (s), 2884 (s), 2858 (w), 2825 (w), 1456 (s), 1344 (s), 1274 (s), 1183 (s), 1173 (s), 1106 (s), 926 (s), 908 (s) cm^{-1}.

R_f = 0.60 (ethyl acetate/hexane 1:9, *v*/*v*).

m.p.: 65 °C (lit.: 62.7–64.5 °C).[15]

Diethyl {difluoro[2-fluoro-4-(4,4,5,5-tetramethyl-1,3,2-dioxaborolan-2-yl)phenyl]methyl} phosphonate (**13a**)

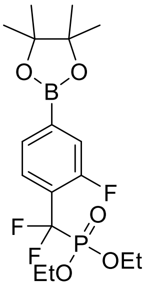

Phosphonate **11c** (880 mg, 2.44 mmol, 1.0 eq.) was dissolved in 8.1 mL of dry 1,4-dioxane degassed by the freeze-pump-thaw method. Under inert gas atmosphere, KOAc (718 mg, 7.32 mmol, 3.0 eq.), bis(pinacolato)diboron (743 mg, 2.93 mmol, 1.2 eq.), Pd(dppf)Cl_2 (54 mg, 0.074 mmol, 3.0 mol%), and dppf (41 mg, 0.074 mmol, 3.0 mol%) were added to the solution, which was stirred at 85 °C for 18 h. After being cooled, the reaction mixture was diluted with ethyl acetate and filtered through a short pad of silica gel. Water was added to the filtrate, and the phases were separated. The aqueous phase was extracted three times with ethyl acetate, and the combined organic phases were washed with water and brine and dried over Na_2SO_4,

after which they were filtered and evaporated. The crude product **13a** was obtained as a brown oil and used directly for subsequent reactions without further purification.

HRMS (ESI) *m*/*z* $[M+H]^+$ calculated for $C_{17}H_{26}BF_3O_5P$: 409.1558, found: 409.1558.

$\mathbf{R_f}$ = 0.33 (acetone/hexane 1:4, *v*/*v*).

Diethyl {difluoro[2-fluoro-4-(4,4,5,5-tetraethyl-1,3,2-dioxaborolan-2-yl)phenyl]methyl} phosphonate (**13b**)

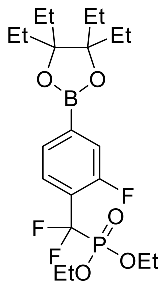

A *Schlenk* flask was charged with **12b** (593 mg, 1.62 mmol, 1.5 eq.), KOAc (325 mg, 3.31 mmol, 3.0 eq.), and a solution of phosphonate **11c** (393 mg, 1.09 mmol, 1.0 eq.) in 4 mL of dry 1,4-dioxane. The mixture was degassed three times by the freeze-pump-thaw method, before Pd(dppf)Cl_2 (40 mg, 0.055 mmol, 5.0 mol%) and dppf (30 mg, 0.054 mmol, 5.0 mol%) were introduced. After being stirred at 85 °C for 16 h, the reaction mixture was cooled to room temperature, filtered through a short pad of celite and washed with acetone. The filtrate was evaporated under reduced pressure, and the resulting residue was purified by flash column chromatography (hexane to ethyl acetate/hexane 1:4, *v*/*v*) to afford **13b** as a colorless oil.

Yield: 376 mg (74 %).

^{1}H-NMR (400 MHz, $(CD_3)_2CO$) δ = 7.71 – 7.67 (m, 1H), 7.64 – 7.59 (m, 1H), 7.56 – 7.51 (m, 1H), 4.30 – 4.15 (m, 4H; OCH_2CH_3), 1.88 – 1.72 (m, 8H; $C(CH_2CH_3)_2$), 1.31 (td, *J* = 7.0, 0.6 Hz, 6H; OCH_2CH_3), 0.98 (t, *J* = 7.5 Hz, 12H; $C(CH_2CH_3)_2$) ppm.

^{19}F-NMR (377 MHz, $(CD_3)_2CO$) δ = -109.00 (dd, $^2J_{F\text{-}P}$ = 110.8 Hz, $^4J_{F\text{-}F}$ = 20.0 Hz, 2F; CF_2), -115.53 – -115.77 (m, 1F; CF) ppm.

^{31}P-NMR (162 MHz, $(CD_3)_2CO$) δ = 3.78 (td, $^2J_{P\text{-}F}$ = 110.7 Hz, $^4J_{P\text{-}F}$ = 2.7 Hz) ppm.

^{13}C-NMR (76 MHz, $(CD_3)_2CO$) δ = 162.1 – 158.5 (m; *C*F), 131.0 (dd, $^4J_{C\text{-}F}$ = 3.4 Hz, $^4J_{C\text{-}P}$ = 1.1 Hz), 129.3 – 129.0 (m), 123.7 (tdd, $^2J_{C\text{-}F}$ = 22.4 Hz, $^2J_{C\text{-}F}$ / $^2J_{C\text{-}P}$ = 12.8, 11.1 Hz), 122.8 (dd, $^2J_{C\text{-}F}$ = 20.2 Hz, $^4J_{C\text{-}P}$ = 1.1 Hz), 117.6 (tdd, $^1J_{C\text{-}F}$ = 263.7 Hz, $^1J_{C\text{-}P}$ = 218.8 Hz, $^3J_{C\text{-}F}$ = 2.3 Hz; CF_2), 90.4 ($C(CH_2CH_3)_2$), 65.5 (d, $^2J_{C\text{-}P}$ = 6.7 Hz; OCH_2CH_3), 27.2 ($C(CH_2CH_3)_2$), 16.6 (d, $^3J_{C\text{-}P}$ = 5.3 Hz; OCH_2CH_3), 9.1 ($C(CH_2CH_3)_2$) ppm.

HRMS (ESI) *m*/*z* $[M+H]^+$ calculated for $C_{21}H_{34}BF_3O_5P^+$: 465.2184, found: 465.2185.

IR (film): ṽ 2981 (s), 2946 (m), 2885 (m), 1564 (m), 1509 (m), 1460 (m), 1411 (s), 1366 (s), 1352 (s), 1276 (s), 1060 (s), 1044 (s), 1021 (s), 927 (m), 897 (m) cm^{-1}.

UV/Vis (DCM): λ_{max} = 235; 275; 282 nm.

R_f = 0.41 (ethyl acetate/hexane 3:7, *v*/*v*).

Diethyl {difluoro[2-fluoro-4-(naphthalen-2-yl)phenyl]methyl}phosphonate (**15a**)

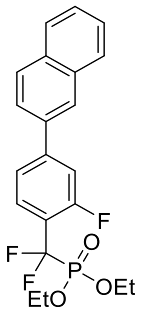

Compound **15a** was synthesized from 2-bromonaphthalene (**14a**, 64.8 mg, 0.313 mmol, 1.0 eq.), compound **13a** (178 mg crude, maximum 0.436 mmol, 1.4 eq.), K_3PO_4 (199 mg, 0.937 mmol, 3.0 eq.), $Pd_2(dba)_3$ (1.5 mg, 0.0016 mmol, 0.51 mol%), and SPhos (2.7 mg, 0.0066 mmol, L:Pd=4:1) in 1.5 mL of dry and degassed toluene according to **Method 1B**. The crude product was purified by flash column chromatography (hexane to ethyl acetate/hexane 3:7, *v/v*) to afford **15a** as a colorless solid.

Yield: 104 mg (81 %).

^{1}H-NMR (400 MHz, $CDCl_3$) δ = 8.05 (d, *J* = 1.9 Hz, 1H), 7.98 – 7.83 (m, 3H), 7.71 (dd, *J* = 8.5, 1.9 Hz, 1H), 7.68 – 7.47 (m, 5H), 4.37 – 4.22 (m, 4H; CH_2CH_3), 1.38 (td, *J* = 7.1, 0.7 Hz, 6H; CH_2CH_3) ppm.

^{19}F-NMR (377 MHz, $CDCl_3$) δ = -107.09 (dd, $^2J_{F\text{-}P}$ = 114.5 Hz, $^4J_{F\text{-}F}$ = 18.7 Hz, 2F; CF_2), -112.03 – -112.22 (m, 1F; CF) ppm.

^{31}P-NMR (162 MHz, $CDCl_3$) δ = 5.43 (td, $^2J_{P\text{-}F}$ = 114.5 Hz, $^4J_{P\text{-}F}$ = 2.6 Hz) ppm.

^{13}C-NMR (101 MHz, $CDCl_3$) δ = 160.5 (dtd, $^1J_{C\text{-}F}$ = 255.2 Hz, $^3J_{C\text{-}F}$ = 4.1 Hz, $^3J_{C\text{-}P}$ = 2.4 Hz; *C*F), 146.4 (dtd, $^3J_{C\text{-}F}$ = 8.2 Hz, $^5J_{C\text{-}F}$ = 1.8 Hz, $^5J_{C\text{-}P}$ = 1.5 Hz), 136.2 – 136.1 (m), 133.6, 133.3, 129.1 (tdd, $^3J_{C\text{-}F}$ = 8.0 Hz, $^3J_{C\text{-}F}$ / $^3J_{C\text{-}P}$ = 2.4, 2.2 Hz), 129.0, 128.5, 127.9, 126.82, 126.81, 126.5, 125.0, 123.0 (dd, $^4J_{C\text{-}F}$ = 3.3 Hz, $^4J_{C\text{-}P}$ = 1.3 Hz), 119.1 – 118.6 (m), 118.2 (tdd, $^1J_{C\text{-}F}$ = 264.6 Hz, $^1J_{C\text{-}P}$ = 221.5 Hz, $^3J_{C\text{-}F}$ = 2.6 Hz; CF_2), 115.6 (dd, $^2J_{C\text{-}F}$ = 22.6 Hz, $^4J_{C\text{-}P}$ = 1.3 Hz), 65.2 (d, $^2J_{C\text{-}P}$ = 6.8 Hz; CH_2CH_3), 16.5 (d, $^3J_{C\text{-}P}$ = 5.6 Hz; CH_2CH_3) ppm.

HRMS (ESI) *m*/*z* $[M+H]^+$ calculated for $C_{21}H_{21}F_3O_3P^+$: 409.1175, found: 409.1175.

IR (KBr): ṽ 3057 (w), 2989 (w), 2933 (w), 2915 (w), 1625 (s), 1600 (w), 1567 (w), 1503 (w), 1412 (m), 1273 (s), 1237 (m), 1061 (s), 1039 (s), 1021 (s), 822 (s), 752 (m) cm^{-1}.

UV/Vis ($CHCl_3$): λ_{max} = 259; 294 nm.

R_f = 0.30 (ethyl acetate/hexane 3:7, *v*/*v*).

m.p.: 80 – 82 °C.

{Difluoro[2-fluoro-4-(naphthalen-2-yl)phenyl]methyl}phosphonic acid (**8a**)

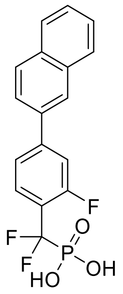

Compound **8a** was prepared from compound **15a** according to **Method 4**. Compound **15a** (52.2 mg, 0.128 mmol, 1.0 eq.) was stirred with TMS-Br (0.110 mL, 128 mg, 0.836 mmol, 6.5 eq.) in 2 mL of dry DCM for 2 h at 0 °C and then for 18 h at room temperature. The crude product was purified by C_{18} reversed-phase column chromatography (water to acetonitrile/water 2:3, *v*/*v*). Lyophilization afforded **8a** as a colorless solid.

Yield: 45 mg (quant.)

^{1}H-NMR (300 MHz, D_2O+CD_3CN, *v*/*v* = 1:1) δ = 8.77 (d, *J* = 1.9 Hz, 1H), 8.58 – 8.43 (m, 3H), 8.39 (dd, *J* = 8.6, 1.9 Hz, 1H), 8.29 – 8.05 (m, 5H) ppm.

^{19}F-NMR (376 MHz, D_2O+CD_3CN, *v*/*v* = 1:1) δ = -105.50 (dd, $^2J_{F\text{-}P}$ = 94.9 Hz, $^4J_{F\text{-}F}$ = 19.6 Hz, 2F; CF_2), -112.30 – -112.55 (m, 1F; CF) ppm.

^{31}P-NMR (162 MHz, D_2O+CD_3CN, *v*/*v* = 1:1) δ = 7.27 – 4.60 (m) ppm.

^{13}C-NMR (101 MHz, D_2O+CD_3CN, *v*/*v* = 1:1) δ = 164.2 – 161.5 (m, *C*F), 147.1 – 146.9 (m), 139.0 – 138.8 (m), 136.3, 135.8, 132.5 – 132.2 (m), 131.6, 131.2, 130.5, 129.6, 129.5, 128.9, 127.8, 125.3 – 125.2 (m), 125.2 – 124.6 (m), 117.9 – 117.5 (m) ppm.

HRMS (ESI) *m*/*z* $[M\text{-}H]^-$ calculated for $C_{17}H_{11}F_3O_3P^+$: 351.0403, found: 351.0405.

IR (KBr): ṽ 3137 (br), 3060 (m), 1627 (s), 1602 (m), 1566 (m), 1503 (w), 1409 (s), 1236 (m), 1129 (m), 813 (s), 747 (m) cm^{-1}.

UV/Vis ($CHCl_3$): λ_{max} = 213; 256; 292 nm.

R_f = 0.46 (acetonitrile/water 2:3, *v*/*v*).

m.p.: decomposition at 214 °C.

Diethyl {difluoro[2-fluoro-4-(furan-3-yl)phenyl]methyl}phosphonate (**15b**)

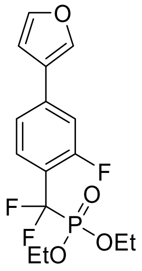

A *Schlenk* flask was charged with 3-bromofuran (**14b**, 26.4 µL, 43.2 mg, 0.294 mmol, 1.0 eq.) and K_3PO_4 (126 mg, 0.594 mmol, 2.0 eq.) under inert gas atmosphere. Compound **13a**

(132 mg crude, maximum 0.323 mmol, 1.1 eq.) dissolved in 1.1 mL of dry 1,4-dioxane was added to the mixture, which was then degassed three times by the freeze-pump-thaw method. $Pd(PPh_3)_4$ (18 mg, 0.016 mmol, 5.4 mol%) was introduced, and the suspension was stirred at 90 °C for 16 h. Once cool, the reaction mixture was diluted with acetone and filtered through a thin layer of silica gel to remove the catalyst. The volatiles were evaporated *in vacuo*, and the crude product was purified by two successive flash column chromatographies (hexane to ethyl acetate/hexane 3:7, *v*/*v*, then hexane to acetone/hexane 3:17, *v*/*v*) to give **15b** as a light-yellow oil.

Yield: 39 mg (38 %).

^{1}H-NMR (400 MHz, $(CD_3)_2CO$) δ = 8.23 – 8.22 (m, 1H), 7.70 (t, *J* = 1.8 Hz, 1H), 7.62 – 7.52 (m, 3H), 7.00 (dd, *J* = 1.9, 0.9 Hz, 1H), 4.30 – 4.15 (m, 4H; CH_2CH$_3$), 1.31 (td, *J* = 7.1, 0.6 Hz, 6H; CH$_2$CH_3) ppm.

^{19}F-NMR (377 MHz, $(CD_3)_2CO$) δ = -108.47 (dd, $^{2}J_{F\text{-}P}$ = 112.7 Hz, $^{4}J_{F\text{-}F}$ = 19.8 Hz, 2F; CF_2), -114.35 – -114.60 (m, 1F; CF) ppm.

^{31}P-NMR (162 MHz, $(CD_3)_2CO$) δ = 4.00 (td, $^{2}J_{P\text{-}F}$ = 112.4 Hz, $^{4}J_{P\text{-}F}$ = 2.3 Hz) ppm.

^{13}C-NMR (76 MHz, $(CD_3)_2CO$) δ = 161.1 (dtd, $^{1}J_{C\text{-}F}$ = 253.3 Hz, $^{3}J_{C\text{-}F}$ = 3.9 Hz, $^{3}J_{C\text{-}P}$ = 2.2 Hz; *C*F), 145.5, 141.7, 138.9 (dtd, $^{3}J_{C\text{-}F}$ = 9.0 Hz, $^{5}J_{C\text{-}F}$ = 1.9 Hz, $^{5}J_{C\text{-}P}$ = 1.8 Hz), 130.0 (tdd, $^{3}J_{C\text{-}F}$ = 7.9 Hz, $^{3}J_{C\text{-}F}$ = 2.3 Hz, $^{3}J_{C\text{-}P}$ = 2.3 Hz), 125.5 (dd, $^{4}J_{C\text{-}F}$ = 1.4 Hz, $^{6}J_{C\text{-}P}$ = 0.8 Hz), 122.2 (dd, $^{4}J_{C\text{-}F}$ = 3.3 Hz, $^{4}J_{C\text{-}P}$ = 1.1 Hz), 119.1 (tdd, $^{2}J_{C\text{-}F}$ = 22.6 Hz, $^{2}J_{C\text{-}F}$ / $^{2}J_{C\text{-}P}$ = 13.3, 11.2 Hz), 118.5 (tdd, $^{1}J_{C\text{-}F}$ = 263.8 Hz, $^{1}J_{C\text{-}P}$ = 220.7 Hz, $^{3}J_{C\text{-}F}$ = 2.5 Hz; *C*F$_2$), 114.5 (dd, $^{2}J_{C\text{-}F}$ = 23.2 Hz, $^{4}J_{C\text{-}P}$ = 1.1 Hz), 109.4, 65.5 (d, $^{2}J_{C\text{-}P}$ = 6.7 Hz; *C*H$_2$CH$_3$), 16.7 (d, $^{3}J_{C\text{-}P}$ = 5.3 Hz; CH$_2$*C*H$_3$) ppm.

HRMS (ESI) *m*/*z* $[M+H]^+$ calculated for $C_{15}H_{17}F_3O_4P^+$: 349.0811, found: 349.0812.

IR (film): ṽ 3128 (w), 2987 (m), 2916 (m), 2856 (w), 1626 (s), 1593 (m), 1569 (m), 1520 (m), 1364 (m), 1274 (s), 1236 (s), 1165 (s), 1146 (s), 1061 (s), 1015 (s), 794 (s), 755 (s), 598 (s), 558 (s) cm^{-1}.

UV/Vis (DCM): λ_{max} = 266; 229 nm.

R$_f$ = 0.35 (acetone/hexane 3:7, *v*/*v*).

{Difluoro[2-fluoro-4-(furan-3-yl)phenyl]methyl}phosphonic acid (**8b**)

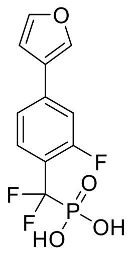

Compound **8b** was synthesized from compound **15b** according to **Method 4**. Compound **15b** (11.0 mg, 0.0316 mmol, 1.0 eq.) was stirred with TMS-Br (85.0 µL, 98.6 mg, 0.644 mmol, 20 eq.) in 1.0 mL of dry DCM for 2 h at 0 °C and then for 32 h at room temperature. The crude

product was purified by C_{18} reversed-phase column chromatography using pure water as the eluent. Compound **8b** was obtained as an off-white solid after lyophilization.

Yield: 9.2 mg (quant.)

^{1}H-NMR (400 MHz, D_2O) δ = 8.08 – 8.06 (m, 1H), 7.67 (t, *J* = 1.8 Hz, 1H), 7.65 – 7.59 (m, 1H), 7.56 – 7.45 (m, 2H), 6.95 (dd, *J* = 1.9, 0.9 Hz, 1H) ppm.

^{19}F-NMR (376 MHz, D_2O) δ = -106.84 (dd, $^{2}J_{F\text{-}P}$ = 100.8 Hz, $^{4}J_{F\text{-}F}$ =18.5 Hz, 2F; CF_2), -113.25 – -113.48 (m, 1F; CF) ppm.

^{31}P-NMR (162 MHz, D_2O) δ = 4.12 (t, $^{2}J_{P\text{-}F}$ = 97.6 Hz) ppm.

^{13}C-NMR (101 MHz, D_2O) δ = 161.3 – 158.5 (m; *C*F), 144.8, 140.4, 136.8 – 136.5 (m), 129.0 – 128.7 (m), 124.7 – 124.4 (m), 121.5 – 121.2 (m), 120.4 – 119.4 (m), 113.9 – 113.5 (m), 108.5 ppm.

HRMS (ESI) *m*/*z* $[M\text{-}H]^-$ calculated for $C_{11}H_7F_3O_4P^-$: 291.0040, found: 291.0032.

IR (KBr): $\tilde{\nu}$ 3126 (br), 3029 (m), 2852 (w), 2794 (w), 1627 (m), 1400 (s), 1235 (w), 792 (w), 596 (w), 559 (w) cm^{-1}.

UV/Vis (H_2O): λ_{max} = 200; 259 nm.

R_f = 0.37 (acetonitrile/water 1:4, *v*/*v*).

m.p.: decomposition at 206 °C.

Diethyl {difluoro[2-fluoro-4-(1-methyl-1*H*-pyrazol-4-yl)phenyl]methyl}phosphonate (**15c**)

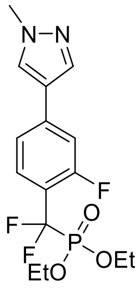

A *Schlenk* flask was charged with 4-bromo-1-methyl-1*H*-pyrazole (**14c**, 27.5 µL, 42.8 mg, 0.266 mmol, 1.0 eq.), K_3PO_4 (170 mg, 0.801 mmol, 3.0 eq.), $Pd_2(dba)_3$ (1.3 mg, 0.0014 mmol, 0.53 mol%), and SPhos (2.3 mg, 0.0056 mmol, L:Pd=4:1) under inert gas atmosphere. Compound **13a** (152 mg crude, 0.372 mmol maximum, 1.4 eq.) dissolved in 1.2 mL of dry toluene degassed by sonication was added to the mixture, which was stirred at 100 °C for 18 h. The reaction mixture was cooled, diluted with acetone, and filtered through a thin layer of silica gel to remove the catalyst. The volatiles were removed under reduced pressure, and the resulting residue was purified by two successive flash column chromatographies (hexane to acetone/hexane 3:2, *v*/*v*, then using a gradient from hexane to pure ethyl acetate) to give **15c** as a light-yellow oil.

Yield: 35 mg (36 %).

^{1}H-NMR (400 MHz, $(CD_3)_2CO$) δ = 8.15 (s, 1H), 7.93 (s, 1H), 7.56 – 7.45 (m, 3H), 4.30 – 4.14 (m, 4H; C*H*$_2$CH$_3$), 3.93 (s, 3H; C*H*$_3$), 1.30 (td, *J* = 7.1, 0.5 Hz, 6H; CH$_2$C*H*$_3$) ppm.
^{19}F-NMR (377 MHz, $(CD_3)_2CO$) δ = -108.23 (dd, $^2J_{F\text{-}P}$ = 113.4 Hz, $^4J_{F\text{-}F}$ = 19.6 Hz, 2F; CF$_2$), -114.55 – -114.78 (m, 1F; CF) ppm.
^{31}P-NMR (162 MHz, $(CD_3)_2CO$) δ = 4.10 (td, $^2J_{P\text{-}F}$ = 113.4 Hz, $^4J_{P\text{-}F}$ = 2.6 Hz) ppm.
^{13}C-NMR (101 MHz, $(CD_3)_2CO$) δ = 161.2 (dtd, $^1J_{C\text{-}F}$ = 253.3 Hz, $^3J_{C\text{-}F}$ = 4.1 Hz, $^3J_{C\text{-}P}$ = 2.5 Hz; *C*F), 139.7 (dtd, $^3J_{C\text{-}F}$ = 9.2 Hz, $^5J_{C\text{-}F}$ = 1.6 Hz, $^5J_{C\text{-}P}$ = 1.6 Hz), 137.5, 129.9 (tdd, $^3J_{C\text{-}F}$ = 7.8 Hz, $^3J_{C\text{-}F}$ = 2.3 Hz, $^3J_{C\text{-}P}$ = 2.3 Hz), 129.3, 121.5 (dd, $^4J_{C\text{-}F}$ = 3.1 Hz, $^4J_{C\text{-}P}$ = 1.1 Hz), 121.5 – 121.4 (m), 118.6 (tdd, $^1J_{C\text{-}F}$ = 263.8 Hz, $^1J_{C\text{-}P}$ = 220.8 Hz, $^3J_{C\text{-}F}$ = 2.5 Hz; *C*F$_2$), 118.0 (tdd, $^2J_{C\text{-}F}$ = 22.7 Hz, $^2J_{C\text{-}F}$ / $^2J_{C\text{-}P}$ = 13.2, 11.7 Hz), 113.6 (dd, $^2J_{C\text{-}F}$ = 23.1 Hz, $^4J_{C\text{-}P}$ = 1.1 Hz), 65.4 (d, $^2J_{C\text{-}P}$ = 6.7 Hz; *C*H$_2$CH$_3$), 39.3 (*C*H$_3$), 16.7 (d, $^3J_{C\text{-}P}$ = 5.3 Hz; CH$_2$*C*H$_3$) ppm.
HRMS (ESI) *m/z* $[M+H]^+$ calculated for $C_{15}H_{19}F_3N_2O_3P^+$: 363.1080, found: 363.1081.
IR (film): ṽ 3107 (w), 2986 (m), 2933 (m), 2853 (w), 2812 (w), 1627 (s), 1574 (s), 1444 (m), 1274 (s), 1238 (s), 1182 (s), 1145 (m), 1057 (s), 1016 (s), 865 (s), 754 (m), 606 (m), 559 (s) cm^{-1}.
UV/Vis (DCM): λ_{max} = 226; 270 nm.
R$_f$ = 0.38 (acetone/hexane 2:3, *v*/*v*).

{Difluoro[2-fluoro-4-(1-methyl-1*H*-pyrazol-4-yl)phenyl]methyl}phosphonic acid (**8c**)

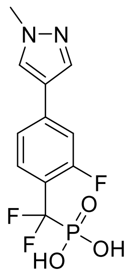

Compound **8c** was prepared from compound **15c** according to **Method 4**. Compound **15c** (11.6 mg, 0.0320 mmol, 1.0 eq.) was stirred with TMS-Br (85.0 µL, 98.6 mg, 0.644 mmol, 20 eq.) in 0.5 mL of dry DCM for 2 h at 0 °C and then for 46 h at room temperature. The crude product was purified by C$_{18}$ reversed-phase column chromatography using pure water as the eluent. Compound **8c** was obtained as a colorless solid after lyophilization.
Yield: 9.8 mg (quant.)
^{1}H-NMR (400 MHz, D_2O) δ = 7.97 (s, 1H), 7.89 (d, *J* = 0.8 Hz, 1H), 7.60 – 7.54 (m, 1H), 7.43 – 7.30 (m, 2H), 3.91 (s, 3H; C*H*$_3$) ppm.
^{19}F-NMR (377 MHz, D_2O) δ = -105.62 (dd, $^2J_{F\text{-}P}$ = 95.4 Hz, $^4J_{F\text{-}F}$ = 18.8 Hz, 2F; CF$_2$), -113.21 – -113.45 (m, 1F; CF) ppm.
^{31}P-NMR (162 MHz, D_2O) δ = 4.42 (t, $^2J_{P\text{-}F}$ = 95.3 Hz) ppm.

^{13}C-NMR (101 MHz, D_2O) δ = 161.3 – 158.4 (m; *C*F), 136.8, 136.1 – 136.0 (m), 129.5, 129.2 – 129.0 (m), 121.0 – 120.9 (m), 120.6 – 120.5 (m), 120.8 – 120.3 (m), 113.1 – 112.7 (m), 38.3 (*C*H$_3$) ppm.

^{13}C-NMR DEPT ^{19}F coupled (101 MHz, D_2O) δ 161.3 – 158.4 (m; *C*F), 124.6 – 116.2 (m; *C*F$_2$) ppm.

HRMS (ESI) *m*/*z* $[M+H]^+$ calculated for $C_{11}H_{11}F_3N_2O_3P^+$: 307.0454, found: 307.0456.

IR (KBr): ṽ 3126 (br), 3033 (m), 1631 (m), 1574 (m), 1400 (br), 1237 (m), 1179 (m), 605 (m), 565 (m) cm^{-1}.

UV/Vis (H_2O): λ_{max} = 201; 262 nm.

R_f = 0.54 (acetonitrile/water 1:4, *v*/*v*).

m.p.: decomposition at 205 °C.

Diethyl {difluoro[2-fluoro-4-(thiophen-2-yl)phenyl]methyl}phosphonate (**15d**)

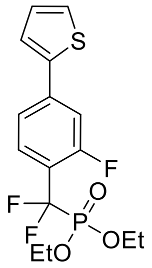

Compound **15d** was synthesized from 2-bromothiophene (**14d**, 22.2 µL, 37.4 mg, 0.229 mmol, 1.0 eq.), compound **13a** (131 mg crude, maximum 0.321 mmol, 1.4 eq.), K_3PO_4 (147 mg, 0.693 mmol, 3.0 eq.), $Pd_2(dba)_3$ (2.0 mg, 0.0022 mmol, 1.0 mol%), and SPhos (3.7 mg, 0.0090 mmol, L:Pd=4:1) in 1.1 mL of dry and degassed toluene according to **Method 1B**. The crude product was purified by flash column chromatography (hexane to ethyl acetate/hexane 3:7, *v*/*v*) to give **15d** as a light-yellow oil.

Yield: 59 mg (71 %).

^{1}H-NMR (400 MHz, $CDCl_3$) δ = 7.60 – 7.43 (m, 2H), 7.40 – 7.34 (m, 3H), 7.11 (dd, J = 5.1, 3.7 Hz, 1H), 4.35 – 4.20 (m, 4H; C*H*$_2$CH$_3$), 1.36 (td, J = 7.1, 0.7 Hz, 6H; CH$_2$C*H*$_3$) ppm.

^{19}F-NMR (377 MHz, $CDCl_3$) δ = -107.20 (dd, $^2J_{F\text{-}P}$ = 114.6 Hz, $^3J_{F\text{-}F}$ = 18.8 Hz; 2F; CF_2), -111.92 – -112.13 (m; 1F; CF) ppm.

^{31}P-NMR (162 MHz, $CDCl_3$) δ = 5.35 (td, $^2J_{P\text{-}F}$ = 114.6 Hz, $^4J_{P\text{-}F}$ = 2.7 Hz) ppm.

^{13}C-NMR (75 MHz, $CDCl_3$) δ = 160.1 (dtd, $^1J_{C\text{-}F}$ = 255.4 Hz, $^3J_{C\text{-}F}$ = 4.0 Hz, $^3J_{C\text{-}P}$ = 2.4 Hz; *C*F), 141.8 – 141.7 (m), 139.4 (dtd, $^3J_{C\text{-}F}$ = 8.4 Hz, $^5J_{C\text{-}F}$ = 1.8 Hz, $^5J_{C\text{-}P}$ = 1.8 Hz), 129.2 (tdd, $^3J_{C\text{-}F}$ = 7.9 Hz, $^3J_{C\text{-}F}$ / $^3J_{C\text{-}P}$ = 2.5, 2.3 Hz), 128.5, 126.8, 125.0, 121.4 (dd, $^4J_{C\text{-}F}$ = 3.4 Hz, $^4J_{C\text{-}P}$ = 1.2 Hz), 119.2 – 118.3 (m), 117.4 (tdd, $^1J_{C\text{-}F}$ = 264.6 Hz, $^1J_{C\text{-}P}$ = 221.6 Hz, $^3J_{C\text{-}F}$ = 2.5 Hz; *C*F$_2$), 113.9 (dd, $^2J_{C\text{-}F}$ = 23.4 Hz, $^4J_{C\text{-}P}$ = 1.2 Hz), 65.2 (d, $^2J_{C\text{-}P}$ = 6.8 Hz; *C*H$_2$CH$_3$), 16.5 (d, $^3J_{C\text{-}P}$ = 5.6 Hz; CH$_2$*C*H$_3$) ppm.

HRMS (ESI) *m/z* [M+H]$^+$ calculated for $C_{15}H_{17}F_3O_3PS^+$: 365.0583, found: 365.0584.

IR (film): ṽ 3088 (w), 2986 (m), 2931 (w), 2915 (w), 1624 (s), 1570 (m), 1537 (w), 1497 (m), 1420 (m), 1272 (s), 1237 (m), 1145 (m), 1042 (s), 1021 (s), 573 (m) cm^{-1}.

UV/Vis ($CHCl_3$): λ_{max} = 297 nm.

R$_f$ = 0.25 (ethyl acetate/hexane 3:7, *v*/*v*).

{Difluoro[2-fluoro-4-(thiophen-2-yl)phenyl]methyl}phosphonic acid (**8d**)

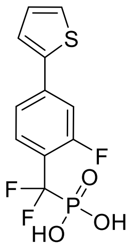

Compound **8d** was prepared from compound **15d** according to **Method 4**. Compound **15d** (48.7 mg, 0.134 mmol, 1.0 eq.) was stirred with TMS-Br (0.110 mL, 128 mg, 0.836 mmol, 6.2 eq.) in 2.1 mL of dry DCM for 2 h at 0 °C and then for 18 h at room temperature. The work-up yielded 43 mg of incompletely deprotected product, as confirmed by ^{1}H NMR spectroscopy. The product was later dissolved in 1.8 mL of dry DCM and stirred again with additional TMS-Br (0.050 mL, 58 mg, 0.38 mmol, 3.2 eq.) for 2 h at 0 °C and then for 16 h at room temperature. The crude product was purified by two successive C_{18} reversed-phase column chromatographies (water to acetonitrile/water 2:3, *v*/*v*) to afford **8d** as a beige solid after lyophilization.

Yield: 41 mg (99 %).

^{1}H-NMR (400 MHz, D_2O) δ = 7.65 – 7.50 (m, 5H), 7.23 – 7.19 (m, 1H) ppm.

^{19}F-NMR (376 MHz, D_2O) δ = -106.14 (dd, $^2J_{F\text{-}P}$ = 96.4 Hz, $^3J_{F\text{-}F}$ = 18.8 Hz, 2F; CF_2), -112.91 – -113.10 (m, 1F; CF) ppm.

^{31}P-NMR (162 MHz, D_2O) δ = 4.17 (t, $^2J_{P\text{-}F}$ = 95.5 Hz) ppm.

^{13}C-NMR (101 MHz, D_2O) δ = 161.2 – 158.4 (m; *C*F), 141.8 – 141.7, 138.0 – 137.8 (m), 129.4 – 129.1 (m), 128.7, 126.9, 125.1, 121.6 – 120.9 (m), 121.2 – 121.0 (m), 121.9 – 114.4 (m; *C*F_2), 113.6 – 113.2 (m) ppm.

HRMS (ESI) *m/z* [M-H]$^-$ calculated for $C_{11}H_7F_3O_3PS^-$: 306.9811, found: 306.9812.

IR (KBr): ṽ 3138 (br), 3030 (w), 2921 (w), 1626 (m), 1568 (w), 1496 (w), 1402 (s), 1140 (m), 585 (m) cm^{-1}.

UV/Vis (H_2O): λ_{max} = 202; 293 nm.

R$_f$ = 0.35 (acetonitrile/water 1:4, *v*/*v*).

m.p.: decomposition at 206 °C.

Diethyl {difluoro[2-fluoro-4-(thiophen-3-yl)phenyl]methyl}phosphonate (**15e**)

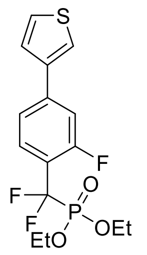

Compound **15e** was synthesized from 3-bromothiophene (**14e**, 18.3 µL, 31.8 mg, 0.195 mmol, 1.0 eq.), compound **13a** (111 mg crude, maximum 0.272 mmol, 1.4 eq.), K_3PO_4 (125 mg, 0.589 mmol, 3.0 eq.), $Pd_2(dba)_3$ (1.3 mg, 0.0014 mmol, 0.72 mol%), and SPhos (2.3 mg, 0.0056 mmol, L:Pd=4:1) in 1.0 mL of dry and degassed toluene according to **Method 1B**. The crude product was chromatographed on silica gel (hexane to ethyl acetate/hexane 3:7, *v*/*v*) to afford **15e** as a light-yellow oil.

Yield: 50 mg (70 %).

^{1}H-NMR (400 MHz, $CDCl_3$) δ = 7.59 – 7.53 (m, 2H), 7.47 – 7.41 (m, 2H), 7.39 – 7.33 (m, 2H), 4.35 – 4.20 (m, 4H; CH_2CH_3), 1.36 (td, J = 7.1, 0.7 Hz, 6H; CH_2CH_3) ppm.

^{19}F-NMR (377 MHz, $CDCl_3$) δ = -107.12 (dd, $^2J_{F\text{-}P}$ = 114.7 Hz, $^3J_{F\text{-}F}$ = 18.8 Hz, 2F; CF_2), -112.11 – -112.35 (m, 1F; CF) ppm.

^{31}P-NMR (162 MHz, $CDCl_3$) δ = 5.41 (td, $^2J_{P\text{-}F}$ = 114.7 Hz, $^4J_{P\text{-}F}$ = 2.7 Hz) ppm.

^{13}C-NMR (101 MHz, $CDCl_3$) δ = 161.7 – 159.0 (m; *C*F), 140.9 – 140.8 (m), 140.2 – 140.1 (m), 129.1 (tdd, $^3J_{C\text{-}F}$ = 7.9 Hz, $^3J_{C\text{-}F}$ / $^3J_{C\text{-}P}$ = 2.5, 2.3 Hz), 127.1, 126.1, 122.4, 122.1 (dd, $^4J_{C\text{-}F}$ = 3.3 Hz, $^4J_{C\text{-}P}$ = 1.1 Hz), 118.6 (tdd, $^2J_{C\text{-}F}$ = 22.9 Hz, $^2J_{C\text{-}F}$ / $^2J_{C\text{-}P}$ = 13.1, 11.3 Hz), 117.5 (tdd, $^1J_{C\text{-}F}$ = 264.3 Hz, $^1J_{C\text{-}P}$ = 221.3 Hz, $^3J_{C\text{-}F}$ = 2.4 Hz; *C*F_2), 114.6 (dd, $^2J_{C\text{-}F}$ = 22.9 Hz, $^4J_{C\text{-}P}$ = 1.2 Hz), 65.2 (d, $^2J_{C\text{-}P}$ = 6.7 Hz; CH_2CH_3), 16.49 (d, $^3J_{C\text{-}P}$ = 5.6 Hz; CH_2CH_3) ppm.

HRMS (ESI) *m/z* $[M+H]^+$ calculated for $C_{15}H_{17}F_3O_3PS^+$: 365.0583, found: 365.0583.

IR (film): ṽ 3095 (w), 2986 (m), 2914 (w), 2851 (w), 1622 (s), 1572 (m), 1539 (w), 1503 (m), 1437 (m), 1274 (s), 1236 (m), 1146 (m), 1041 (s), 1020 (s), 786 (m), 595 (m) cm^{-1}.

UV/Vis ($CHCl_3$): λ_{max} = 269 nm.

R_f = 0.24 (ethyl acetate/hexane 3:7, *v*/*v*).

{Difluoro[2-fluoro-4-(thiophen-3-yl)phenyl]methyl}phosphonic acid (**8e**)

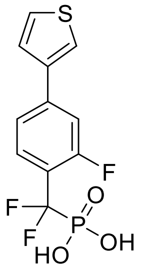

Compound **8e** was prepared from compound **15e** according to **Method 4**. Compound **15e** (40.9 mg, 0.112 mmol, 1.0 eq.) was stirred with TMS-Br (0.0900 mL, 104 mg, 0.679 mmol, 6.1 eq.) in 1.7 mL of dry DCM for 2 h at 0 °C and then for 18 h at room temperature. The work-up yielded 36 mg of incompletely deprotected product, as confirmed by 1H NMR spectroscopy. The product was then dissolved in 1.6 mL of dry DCM and stirred again with additional TMS-Br (0.040 mL, 46 mg, 0.30 mmol, 3.0 eq.) for 2 h at 0 °C and then for 16 h at room temperature. The crude product was purified by two successive C_{18} reversed-phase column chromatographies (water to acetonitrile/water 2:3, *v*/*v*), affording **8e** as an off-white solid after lyophilization.

Yield: 34 mg (98 %).

^{1}H-NMR (400 MHz, D_2O) δ = 7.84 – 7.81 (m, 1H), 7.68 – 7.50 (m, 5H) ppm.

^{19}F-NMR (376 MHz, D_2O) δ = -106.03 (dd, $^{2}J_{F\text{-}P}$ = 96.5 Hz, $^{3}J_{F\text{-}F}$ = 18.7 Hz, 2F; CF_2), -113.04 – -113.23 (m, 1F; CF) ppm.

^{31}P-NMR (162 MHz, D_2O) δ = 4.21 (t, $^{2}J_{P\text{-}F}$ = 96.4 Hz) ppm.

^{13}C-NMR (101 MHz, D_2O) δ = 161.2 – 158.5 (m; *C*F), 139.9 – 139.8 (m), 139.4 – 139.2 (m), 129.2 – 128.9 (m), 127.5, 126.1, 122.6, 121.8 – 121.6 (m), 121.3 – 120.6 (m), 122.0 – 114.4 (m; *C*F_2), 114.3 – 113.9 (m) ppm.

HRMS (ESI) *m*/*z* $[M-H]^-$ calculated for $C_{11}H_7F_3O_3PS^-$: 306.9811, found: 306.9811.

IR (KBr): ṽ 3153 (br), 3029 (w), 2786 (w), 1626 (br), 1573 (w), 1503 (w), 1400 (m), 1143 (m), 780 (m), 597 (w) cm^{-1}.

UV/Vis (H_2O): λ_{max} = 202; 267 nm.

R_f = 0.35 (acetonitrile/water 1:4, *v*/*v*).

m.p.: decomposition at 208 °C.

Methyl 4-{4-[(diethylphosphono)(difluoro)methyl]-3-fluorophenyl}thiophene-2-carboxylate (**15f**)

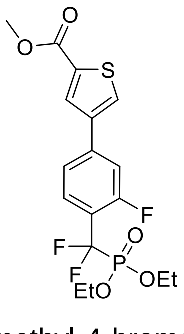

Compound **15f** was synthesized from methyl 4-bromothiophene-2-carboxylate (**14f**, 15.9 µL, 27.0 mg, 0.122 mmol, 1.0 eq.), compound **13b** (79.4 mg, 0.171 mmol, 1.4 eq.), K_3PO_4 (78.3 mg, 0.368 mmol, 3.0 eq.), $Pd_2(dba)_3$ (1.1 mg, 0.0012 mmol, 1.0 mol%), and SPhos (2.0 mg, 0.0049 mmol, L:Pd=4:1) in 0.85 mL of dry toluene according to **Method 1C**. The crude

product was purified by two successive flash column chromatographies (hexane to ethyl acetate/hexane 3:7, *v*/*v*) to give **15f** as a colorless solid.

Yield: 45 mg (87 %).

^{1}H-NMR (400 MHz, $CDCl_3$) δ = 8.05 (d, *J* = 1.7 Hz, 1H), 7.73 (d, *J* = 1.6 Hz, 1H), 7.61 – 7.55 (m, 1H), 7.46 – 7.42 (m, 1H), 7.38 – 7.32 (m, 1H), 4.35 – 4.20 (m, 4H; CH_2CH_3), 3.92 (s, 3H; OCH_3), 1.36 (td, *J* = 7.1, 0.7 Hz, 6H; CH_2CH_3) ppm.

^{19}F-NMR (377 MHz, $CDCl_3$) δ = -107.30 (dd, $^2J_{F-P}$ = 113.8 Hz, $^4J_{F-F}$ = 18.6 Hz, 2F; CF_2), -111.50 – -111.71 (m, 1F; CF) ppm.

^{31}P-NMR (162 MHz, $CDCl_3$) δ = 5.26 (td, $^2J_{P-F}$ = 113.9 Hz, $^4J_{P-F}$ = 2.9 Hz) ppm.

^{13}C-NMR (75 MHz, $CDCl_3$) δ = 162.4 (*C*O), 160.4 (dtd, $^1J_{C-F}$ = 255.7 Hz, $^3J_{C-F}$ = 3.8 Hz, $^3J_{C-P}$ = 2.4 Hz; *C*F), 140.6 – 140.5 (m), 139.7 (dtd, $^3J_{C-F}$ = 8.4 Hz, $^5J_{C-F}$ = 1.6 Hz, $^5J_{C-P}$ = 1.6 Hz), 135.1, 131.8, 129.4 (tdd, $^3J_{C-F}$ = 7.9 Hz, $^3J_{C-F}$ / $^3J_{C-P}$ = 2.5, 2.3 Hz), 128.6, 122.0 (dd, $^4J_{C-F}$ = 3.4 Hz, $^4J_{C-P}$ = 1.4 Hz), 119.9 – 118.9 (m), 117.2 (tdd, $^4J_{C-P}$ = 264.7 Hz, $^4J_{C-P}$ = 221.3 Hz, $^4J_{C-P}$ = 2.4 Hz; CF_2), 114.6 (dd, $^2J_{C-F}$ = 23.1 Hz, $^4J_{C-P}$ = 1.4 Hz), 65.2 (d, $^2J_{C-P}$ = 7.3 Hz; CH_2CH_3), 52.6 (OCH_3), 16.5 (d, $^3J_{C-P}$ = 5.6 Hz; CH_2CH_3)

HRMS (ESI) *m*/*z* $[M+H]^+$ calculated for $C_{17}H_{19}F_3O_5PS^+$: 423.0637, found: 423.0636.

IR (KBr): ṽ 3102 (m), 2993 (w), 2951 (w), 2918 (w), 1707 (s), 1625 (m), 1575 (w), 1550 (m), 1511 (w), 1446 (s), 1281 (s), 1267 (s), 1192 (m), 1041 (s), 1030 (s), 589 (m) cm^{-1}.

UV/Vis (DCM): λ_{max} = 251 nm.

R_f = 0.20 (ethyl acetate/hexane 3:7, *v*/*v*).

m.p.: 138 – 140 °C.

<u>(Difluoro{2-fluoro-4-[5-(methoxycarbonyl)thiophen-3-yl]phenyl}methyl)phosphonic acid (**8f**)</u>

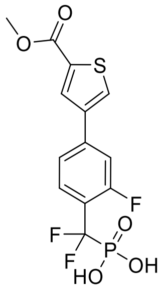

Compound **8f** was prepared from compound **15f** according to **Method 4**. Compound **15f** (21.6 mg, 0.0511 mmol, 1.0 eq.) was stirred with TMS-Br (0.135 mL, 157 mg, 1.03 mmol, 20 eq.) in 0.8 mL of dry DCM for 2 h at 0 °C and then for 23 h at room temperature. The crude product was purified by C_{18} reversed-phase column chromatography (water to acetonitrile/water 1:4, *v*/*v*) to give **8f** as a colorless solid after lyophilization.

Yield: 19 mg (quant.)

^{1}H-NMR (400 MHz, D_2O+CD_3CN, *v*/*v* = 1:1) δ = 8.65 (d, *J* = 1.6 Hz, 1H), 8.54 (d, *J* = 1.6 Hz, 1H), 8.12 – 8.06 (m, 1H), 8.03 – 7.99 (m, 1H), 7.97 – 7.91 (m, 1H), 4.35 (s, 3H; OCH_3) ppm.

^{19}F-NMR (377 MHz, D_2O+CD_3CN, *v*/*v* = 1:1) δ = -105.44 (dd, $^{2}J_{F\text{-}P}$ = 92.8 Hz, $^{4}J_{F\text{-}F}$ =19.6 Hz, 2F; CF_2), -112.18 – -112.38 (m; 1F; CF) ppm.

^{31}P-NMR (162 MHz, D_2O+CD_3CN, *v*/*v* = 1:1) δ = 3.99 (t, $^{2}J_{P\text{-}F}$ = 92.9 Hz) ppm.

^{13}C-NMR (75 MHz, D_2O+CD_3CN, *v*/*v* = 1:1) δ = 163.8 (*C*O), 162.0 – 158.5 (m; *C*F), 141.1 – 141.0 (m), 138.1 – 137.9 (m), 134.3, 132.6, 129.9, 130.1 – 129.8 (m), 123.4 – 122.4 (m), 121.9 (dd, $^{4}J_{C\text{-}F}$ = 3.3 Hz, $^{4}J_{C\text{-}P}$ = 1.1 Hz), 114.6 – 114.2 (m), 52.9 (O*C*H_3).

HRMS (ESI) *m*/*z* [M-H]$^-$ calculated for $C_{13}H_9F_3O_5PS^-$: 364.9866, found: 364.9862.

IR (KBr): ṽ 3132 (br), 2956 (w), 2848 (w), 2800 (w), 1713 (s), 1627 (m), 1574 (w), 1546 (m), 1510 (w), 1446 (m), 1401 (m), 1264 (s), 1179 (m), 597 (m) cm^{-1}.

UV/Vis (H_2O): λ_{max} = 202; 251 nm.

R$_f$ = 0.48 (acetonitrile/water 3:7, *v*/*v*).

m.p.: decomposition at 220 °C.

Diethyl {[4-(5-carbamoylthiophen-3-yl)-2-fluorophenyl](difluoro)methyl}phosphonate (**15g**)

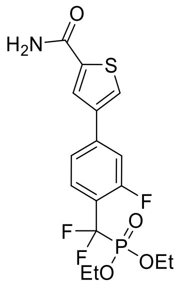

Compound **15g** was synthesized from 4-bromothiophene-2-carboxamide (**14g**, 25.5 mg, 0.124 mmol, 1.0 eq.), compound **13b** (80.4 mg, 0.173 mmol, 1.4 eq.), K_3PO_4 (79.9 mg, 0.376 mmol, 3.0 eq.), $Pd_2(dba)_3$ (1.2 mg, 0.0013 mmol, 1.0 mol%), and SPhos (2.1 mg, 0.0051 mmol, L:Pd=4:1) in 0.85 mL of dry toluene according to **Method 1C**. The crude product was chromatographed on silica gel (ethyl acetate/hexane 2:3 to 4:1, *v*/*v*), affording **15g** as an off-white solid.

Yield: 37 mg (73 %).

^{1}H-NMR (400 MHz, CD_3OD) δ = 8.13 (d, *J* = 1.5 Hz, 1H), 8.08 (d, *J* = 1.5 Hz, 1H), 7.66 – 7.54 (m, 3H), 4.34 – 4.19 (m, 4H; C*H*$_2$CH$_3$), 1.34 (td, *J* = 7.1, 0.7 Hz, 6H; CH$_2$C*H*$_3$) ppm.

^{19}F-NMR (377 MHz, CD_3OD) δ = -109.25 (dd, $^{2}J_{F\text{-}P}$ = 116.2 Hz, $^{4}J_{F\text{-}F}$ = 19.0 Hz, 2F; CF_2), -113.82 – -114.05 (m, 1F; CF) ppm.

^{31}P-NMR (162 MHz, CD_3OD) δ = 4.44 (td, $^{2}J_{P\text{-}F}$ = 116.2 Hz, $^{4}J_{P\text{-}F}$ = 2.8 Hz) ppm.

^{13}C-NMR (75 MHz, CD_3OD) δ = 166.1 (*C*O), 161.5 (dtd, $^{1}J_{C\text{-}F}$ = 254.4 Hz, $^{3}J_{C\text{-}F}$ = 4.0 Hz, $^{3}J_{C\text{-}P}$ = 2.3 Hz; *C*F), 141.8 (dtd, $^{3}J_{C\text{-}F}$ = 8.8 Hz, $^{5}J_{C\text{-}F}$ = 1.8 Hz, $^{5}J_{C\text{-}P}$ = 1.8 Hz), 141.5, 141.4 (dd, $^{4}J_{C\text{-}F}$ = 2.0 Hz, $^{6}J_{C\text{-}P}$ = 0.9 Hz), 130.2 (tdd, $^{3}J_{C\text{-}F}$ = 8.0 Hz, $^{3}J_{C\text{-}F}$ = 2.4 Hz, $^{3}J_{C\text{-}P}$ = 2.4 Hz), 129.2, 128.8, 123.0 (dd, $^{4}J_{C\text{-}F}$ = 3.3 Hz, $^{4}J_{C\text{-}P}$ = 1.2 Hz), 119.6 (tdd, $^{2}J_{C\text{-}F}$ = 22.6 Hz, $^{2}J_{C\text{-}F}$ / $^{2}J_{C\text{-}P}$ = 13.6, 11.8 Hz),

118.7 (tdd, $^{1}J_{C\text{-}F}$ = 263.6 Hz, $^{1}J_{C\text{-}P}$ = 224.4 Hz, $^{3}J_{C\text{-}F}$ = 2.6 Hz), 115.3 (dd, $^{2}J_{C\text{-}F}$ = 23.2 Hz, $^{4}J_{C\text{-}P}$ = 1.3 Hz), 66.8 (d, $^{2}J_{C\text{-}P}$ = 7.0 Hz; CH_2CH_3), 16.7 (d, $^{3}J_{C\text{-}P}$ = 5.4 Hz; CH_2CH_3) ppm.
HRMS (ESI) *m*/*z* $[M+H]^+$ calculated for $C_{16}H_{18}F_3NO_4PS^+$: 408.0641, found: 408.0642.
IR (KBr): ṽ 3426 (m), 3371 (m), 3174 (w), 3105 (w), 2986 (w), 2929 (w), 1666 (s), 1647 (s), 1623 (s), 1575 (m), 1547 (m), 1511 (m), 1266 (s), 1149 (m), 1115 (m), 1059 (s), 1024 (s) cm^{-1}.
UV/Vis (DCM): λ_{max} = 250 nm.
R_f = 0.19 (ethyl acetate/hexane 4:1, *v*/*v*).
m.p.: decomposition at 184 °C.

{[4-(5-Carbamoylthiophen-3-yl)-2-fluorophenyl](difluoro)methyl}phosphonic acid (**8g**)

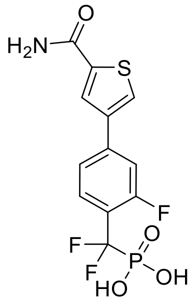

Compound **8g** was prepared from compound **15g** according to **Method 4**. Compound **15g** (21.9 mg, 0.0538 mmol, 1.0 eq.) was stirred with TMS-Br (0.140 mL, 162 mg, 1.06 mmol, 20 eq.) in 1.6 mL of dry DCM for 2 h at 0 °C and then for 46 h at room temperature. The crude product was purified by C_{18} reversed-phase column chromatography (water to acetonitrile/water 1:9, *v*/*v*) to afford **8g** as an off-white solid after lyophilization.
Yield: 19 mg (quant.)
^{1}H-NMR (400 MHz, D_2O) δ = 7.86 (d, *J* = 1.5 Hz, 1H), 7.83 (d, *J* = 1.5 Hz, 1H), 7.61 – 7.54 (m, 1H), 7.44 – 7.39 (m, 1H), 7.36 – 7.31 (m, 1H) ppm.
^{19}F-NMR (377 MHz, D_2O) δ = -106.23 (dd, $^{2}J_{F\text{-}P}$ = 95.8 Hz, $^{4}J_{F\text{-}F}$ = 18.8 Hz, 2F; CF_2), -112.82 – -113.04 (m, 1F; CF) ppm.
^{31}P-NMR (162 MHz, D_2O) δ = 5.45 – 3.09 (m) ppm.
^{13}C-NMR (75 MHz, D_2O) δ = 166.0 (*C*O), 161.4 – 157.8 (m; *C*F), 140.1 – 140.0 (m), 138.0 – 137.7 (m), 137.3, 129.2 – 128.7 (m), 128.4, 127.9, 121.3 – 121.1 (m), 113.9 – 113.4 (m) ppm.
HRMS (ESI) *m*/*z* $[M-H]^-$ calculated for $C_{12}H_8F_3NO_4PS^-$: 349.9869, found: 349.9870.
IR (KBr): ṽ 3443 (br), 3147 (m), 3031 (w), 2925 (w), 2799 (w), 1649 (m), 1627 (m), 1605 (m), 1575 (w), 1545 (w), 1511 (w), 1400 (br), 1251 (w), 1143 (m), 1114 (m) cm^{-1}.
UV/Vis (H_2O): λ_{max} = 202; 251 nm.
R_f = 0.49 (acetonitrile/water 1:4, *v*/*v*).
m.p.: decomposition at 179 °C.

Diethyl {[4-(5-acetylthiophen-3-yl)-2-fluorophenyl](difluoro)methyl}phosphonate (**15h**)

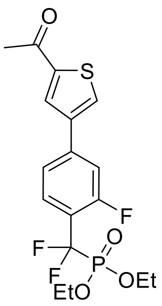

Compound **15h** was synthesized from 1-(4-bromothiophen-2-yl)ethan-1-one (**14h**, 28.4 µL, 45.4 mg, 0.221 mmol, 1.0 eq.), compound **13a** (127 mg crude, maximum 0.311 mmol, 1.4 eq.), K_3PO_4 (142 mg, 0.669 mmol, 3.0 eq.), $Pd_2(dba)_3$ (1.1 mg, 0.0012 mmol, 0.54 mol%), and SPhos (2.0 mg, 0.0049 mmol, L:Pd=4:1) in 1.5 mL of dry and degassed toluene according to **Method 1B**. The crude product was purified by two successive flash column chromatographies (hexane to acetone/hexane 1:4, *v*/*v*, then hexane to ethyl acetate/ hexane 1:1, *v/v*) to give **15h** as a light-yellow solid.

Yield: 46 mg (51 %).

^{1}H-NMR (400 MHz, $CDCl_3$) δ = 7.92 (d, *J* = 1.5 Hz, 1H), 7.81 (d, *J* = 1.5 Hz, 1H), 7.62 – 7.56 (m, 1H), 7.46 – 7.42 (m, 1H), 7.38 – 7.32 (m, 1H), 4.35 – 4.20 (m, 4H; CH_2CH_3), 2.61 (s, 3H; CH_3), 1.36 (td, *J* = 7.1, 0.6 Hz, 6H; CH_2CH_3) ppm.

^{19}F-NMR (377 MHz, $CDCl_3$) δ = -107.33 (dd, $^2J_{F\text{-}P}$ = 113.8 Hz, $^4J_{F\text{-}F}$ = 18.8 Hz, 2F; CF_2), -111.40 – -111.61 (m, 1F; CF) ppm.

^{31}P-NMR (162 MHz, $CDCl_3$) δ = 5.21 (td, $^2J_{P\text{-}F}$ = 113.8 Hz, $^4J_{P\text{-}F}$ = 2.9 Hz) ppm.

^{13}C-NMR (101 MHz, $CDCl_3$) δ = 190.6 (*C*O), 161.7 – 159.0 (m; *C*F), 145.8, 141.0 – 140.9 (m), 139.8 – 139.6 (m), 130.6, 129.9, 129.4 (tdd, $^3J_{C\text{-}F}$ = 7.9 Hz, $^3J_{C\text{-}F}$ / $^3J_{C\text{-}P}$ = 2.5, 2.3 Hz), 122.0 (dd, $^4J_{C\text{-}F}$ = 3.4 Hz, $^4J_{C\text{-}P}$ = 1.2 Hz), 119.5 (tdd, $^2J_{C\text{-}F}$ = 22.6 Hz, $^2J_{C\text{-}F}$ / $^2J_{C\text{-}P}$ = 13.5, 11.7 Hz), 117.7 (tdd, $^1J_{C\text{-}F}$ = 264.6 Hz, $^1J_{C\text{-}P}$ = 221.3 Hz, $^3J_{C\text{-}F}$ = 2.5 Hz; CF_2), 114.6 (dd, $^2J_{C\text{-}F}$ = 23.1 Hz, $^4J_{C\text{-}P}$ = 1.2 Hz), 65.2 (d, $^2J_{C\text{-}P}$ = 6.8 Hz; CH_2CH_3), 27.1 (CH_3), 16.5 (d, $^3J_{C\text{-}P}$ = 5.6 Hz; CH_2CH_3) ppm.

HRMS (ESI) *m*/*z* $[M+H]^+$ calculated for $C_{17}H_{19}F_3O_4PS^+$: 407.0688, found: 407.0688.

IR (KBr): ṽ 3094 (m), 2996 (m), 2916 (w), 1660 (s), 1622 (s), 1575 (m), 1543 (m), 1509 (m), 1280 (s), 1265 (s), 1185 (m), 1056 (s), 1046 (s), 800 (m) cm^{-1}.

UV/Vis ($CHCl_3$): λ_{max} = 291; 309 nm.

R_f = 0.19 (ethyl acetate/hexane 2:3, *v*/*v*).

m.p.: 84 – 86 °C.

{[4-(5-Acetylthiophen-3-yl)-2-fluorophenyl](difluoro)methyl}phosphonic acid (**8h**)

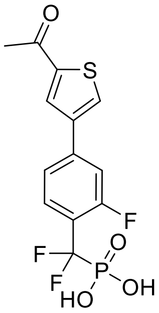

Compound **8h** was prepared from compound **15h** according to **Method 4**. Compound **15h** (26.0 mg, 0.0640 mmol, 1.0 eq.) was stirred with TMS-Br (0.0680 mL, 78.9 mg, 0.515 mmol, 8.0 eq.) in 1.0 mL of dry DCM for 2 h at 0 °C and then for 16 h at room temperature. The crude product was purified by C_{18} reversed-phase column chromatography (water to acetonitrile/water 3:7, *v*/*v*) to afford **8h** as a colorless solid after lyophilization.

Yield: 19 mg (84 %).

^{1}H-NMR (400 MHz, D_2O) δ = 8.10 (d, *J* = 1.5 Hz, 1H), 8.06 (d, *J* = 1.5 Hz, 1H), 7.60 – 7.54 (m, 1H), 7.48 – 7.43 (m, 1H), 7.41 – 7.35 (m, 1H), 2.58 (s, 3H; C*H*$_3$) ppm.

^{19}F-NMR (377 MHz, D_2O) δ = -106.43 (dd, $^2J_{F\text{-}P}$ = 96.5 Hz, $^4J_{F\text{-}F}$ = 18.8 Hz, 2F; CF_2), -112.74 – -112.97 (m, 1F; CF) ppm.

^{31}P-NMR (162 MHz, D_2O) δ = 4.09 (t, $^2J_{P\text{-}F}$ = 96.4 Hz) ppm.

^{13}C-NMR (76 MHz, D_2O) δ = 195.8 (*C*O), 161.5 – 157.9 (m; *C*F), 143.7, 140.9 – 140.6 (m), 137.9 – 137.6 (m), 133.2, 131.8, 129.3 – 128.9 (m), 121.9 – 121.0 (m), 121.5 – 121.3 (m), 114.1 – 113.6 (m), 26.1 (*C*H$_3$) ppm.

HRMS (ESI) *m*/*z* $[M\text{-}H]^-$ calculated for $C_{13}H_9F_3O_4PS^-$: 348.9917, found: 348.9919.

IR (KBr): ṽ 3686 (br), 2926 (w), 2854 (w), 1648 (m), 1630 (m), 1574 (w), 1545 (w), 1509 (w), 1400 (m), 1182 (w), 1113 (m), 801 (w) cm^{-1}.

UV/Vis (H_2O): λ_{max} = 258; 319 nm.

R_f = 0.41 (acetonitrile/water 1:4, *v*/*v*).

m.p.: decomposition at 204 °C.

Diethyl {[4-(5-chlorothiophen-3-yl)-2-fluorophenyl](difluoro)methyl}phosphonate (**15i**)

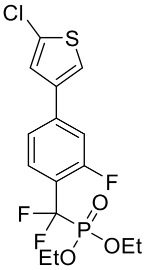

A *Schlenk* flask was charged with 4-bromo-2-chlorothiophene (**14i**, 24.3 µL, 44.8 mg, 0.227 mmol, 1.0 eq.) and 0.340 mL of an aqueous 2 M Na_2CO_3 solution under inert gas

atmosphere. Compound **13b** (148 mg, 0.319 mmol, 1.4 eq.) dissolved in 1.2 mL of dry dimethoxyethane was added to the mixture, which was then degassed three times by the freeze-pump-thaw method. $Pd(PPh_3)_4$ (16.5 mg, 0.0143 mmol, 6.3 mol%) was introduced, and the suspension was stirred at 80 °C for 16 h. Once cool, it was diluted with water and extracted three times with ethyl acetate. The combined organic layers were washed twice with water, before being dried over Na_2SO_4, filtered, and evaporated under reduced pressure. The resulting residue was purified by two successive flash column chromatographies (hexane to ethyl acetate/hexane 3:17, *v/v*, then hexane to acetone/hexane 1:19, *v/v*) to afford **15i** as a light-yellow oil.

Yield: 25 mg (28 %).

^{1}H-NMR (400 MHz, $(CD_3)_2CO$) δ = 7.88 (d, *J* = 1.8 Hz, 1H), 7.71 – 7.58 (m, 4H), 4.31 – 4.16 (m, 4H, C*H*$_2$CH$_3$), 1.31 (td, *J* = 7.0, 0.6 Hz, 6H, CH$_2$C*H*$_3$) ppm.

^{19}F-NMR (377 MHz, $(CD_3)_2CO$) δ = -108.65 (dd, $^2J_{F\text{-}P}$ = 111.8 Hz, $^4J_{F\text{-}F}$ = 19.8 Hz, 2F; CF_2), -113.87 – -114.07 (m, 1F; CF) ppm.

^{31}P-NMR (162 MHz, $(CD_3)_2CO$) δ = 3.88 (td, $^2J_{P\text{-}F}$ = 112.0 Hz, $^4J_{P\text{-}F}$ = 2.3 Hz) ppm.

^{13}C-NMR (101 MHz, $(CD_3)_2CO$) δ = 161.1 (dtd, $^1J_{C\text{-}F}$ = 254.1 Hz, $^3J_{C\text{-}F}$ = 3.8 Hz, $^3J_{C\text{-}P}$ = 2.3 Hz; *C*F), 140.8 (dtd, $^3J_{C\text{-}F}$ = 8.8 Hz, $^5J_{C\text{-}F}$ = 1.7 Hz, $^5J_{C\text{-}P}$ = 1.7 Hz), 140.0 – 139.9 (m), 131.5, 130.1 (tdd, $^3J_{C\text{-}F}$ = 8.0 Hz, $^3J_{C\text{-}F}$ / $^3J_{C\text{-}P}$ = 2.4, 2.3 Hz), 126.5, 122.9, 122.6 (dd, $^4J_{C\text{-}F}$ = 3.3 Hz, $^4J_{C\text{-}P}$ = 1.3 Hz), 119.9 (tdd, $^2J_{C\text{-}F}$ = 22.7 Hz, $^2J_{C\text{-}F}$ / $^2J_{C\text{-}P}$ = 13.3, 11.7 Hz), 118.4 (tdd, $^1J_{C\text{-}F}$ = 263.5 Hz, $^1J_{C\text{-}P}$ = 219.9 Hz, $^3J_{C\text{-}F}$ = 2.5 Hz; *C*F$_2$), 115.0 (dd, $^2J_{C\text{-}F}$ = 23.4 Hz, $^4J_{C\text{-}P}$ = 1.2 Hz), 65.5 (d, $^2J_{C\text{-}P}$ = 6.8 Hz; *C*H$_2$CH$_3$), 16.66 (d, $^3J_{C\text{-}P}$ = 5.3 Hz; CH$_2$*C*H$_3$) ppm.

HRMS (ESI) *m/z* $[M+H]^+$ calculated for $C_{15}H_{16}ClF_3O_3PS^+$: 399.0193, found: 399.0193.

IR (film): ṽ 3088 (w), 2984 (m), 2927 (m), 2853 (w), 1624 (m), 1575 (m), 1541 (w), 1505 (m), 1438 (m), 1274 (s), 1236 (m), 1181 (m), 1147 (m), 1042 (s), 1020 (s), 823 (m), 795 (m), 749 (m) cm^{-1}.

UV/Vis (DCM): λ_{max} = 242; 270 nm.

R$_f$ = 0.13 (ethyl acetate/hexane 1:4, *v/v*).

{[4-(5-Chlorothiophen-3-yl)-2-fluorophenyl](difluoro)methyl}phosphonic acid (**8i**)

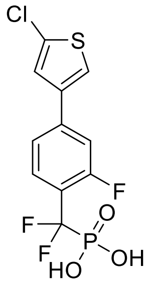

Compound **8i** was prepared from compound **15i** according to **Method 4**. Compound **15i** (10.9 mg, 0.0273 mmol, 1.0 eq.) was stirred with TMS-Br (0.0720 mL, 83.5 mg, 0.545 mmol,

20 eq.) in 0.8 mL of dry DCM for 2 h at 0 °C and then for 46 h at room temperature. The crude product was purified by C_{18} reversed-phase column chromatography using pure water as the eluent. Compound **8i** was obtained as an off-white solid after lyophilization.

Yield: 9.3 mg (99 %).

^{1}H-NMR (400 MHz, D_2O+CD_3CN, *v*/*v* = 1:1) δ = 8.26 – 8.19 (m, 2H), 8.14 – 8.00 (m, 3H) ppm.

^{19}F-NMR (282 MHz, D_2O+CD_3CN, *v*/*v* = 1:1) δ = -105.76 (dd, $^2J_{F\text{-}P}$ = 95.4 Hz, $^4J_{F\text{-}F}$ = 19.8 Hz, 2F; CF_2), -112.16 – -112.44 (m, 1F; CF) ppm.

^{31}P-NMR (162 MHz, D_2O+CD_3CN, *v*/*v* = 1:1) δ = 3.74 – 1.84 (m) ppm.

^{13}C-NMR (101 MHz, D_2O+CD_3CN, *v*/*v* = 1:1) δ = 161.1 – 158.4 (m; *C*F), 139.2 – 139.1 (m), 138.0 – 137.8 (m), 130.3, 129.5 – 129.2 (m), 125.4, 122.3 – 121.6 (m), 121.1, 113.8 – 113.4 (m) ppm.

HRMS (ESI) *m*/*z* $[M\text{-}H]^-$ calculated for $C_{11}H_6ClF_3O_3PS^-$: 340.9421, found: 340.9419.

IR (KBr): ṽ 3125 (br), 3020 (w), 2926 (w), 2851 (w), 1626 (m), 1574 (w), 1441 (w), 1505 (w), 1435 (m), 1401 (s), 1236 (w), 1176 (m), 1144 (m), 821 (w), 795 (w), 747 (w) cm^{-1}.

UV/Vis (H_2O): λ_{max} = 201; 239; 268 nm.

R_f = 0.46 (acetonitrile/water 3:7, *v*/*v*).

m.p.: decomposition at 186 °C.

Diethyl {[4-(2-chlorothiophen-3-yl)-2-fluorophenyl](difluoro)methyl}phosphonate (**15j**)

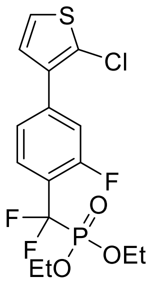

$Pd(PPh_3)_4$ (13.5 mg, 0.0117 mmol, 6.2 mol%) and 0.3 mL of a degassed aqueous 2 M Na_2CO_3 solution were added to a mixture of 3-bromo-2-chlorothiophene (**14i**, 20.7 µL, 37.4 mg, 0.189 mmol, 1.0 eq.) and compound **13a** (109 mg crude, maximum 0.267 mmol, 1.4 eq.) dissolved in 0.9 mL of dry and degassed dimethoxyethane. The suspension was stirred at 80 °C for 16 h under inert gas atmosphere, before being cooled to room temperature, diluted with water, and extracted three times with ethyl acetate. The combined organic phases were washed twice with water and dried over Na_2SO_4, after which they were evaporated *in vacuo*. The crude product was purified by two successive flash column chromatographies (hexane to acetone/hexane 3:17, *v*/*v*, then and hexane to ethyl acetate/hexane 3:7, *v*/*v*) to afford **15j** as a light-yellow oil.

Yield: 18 mg (24 %).

^{1}H-NMR (400 MHz, $CDCl_3$) δ = 7.63 – 7.57 (m, 1H), 7.46 – 7.37 (m, 2H), 7.19 (d, *J* = 5.8 Hz, 1H), 7.06 (d, *J* = 5.8 Hz, 1H), 4.36 – 4.20 (m, 4H), 1.36 (td, *J* = 7.1, 0.7 Hz, 6H) ppm.

^{19}F-NMR (377 MHz, $CDCl_3$) δ = -107.19 (dd, *J* = 113.9, 18.7 Hz; 2F, CF_2), -111.92 – -112.12 (m; 1F, CF) ppm.

^{31}P-NMR (162 MHz, $CDCl_3$) δ = 5.26 (td, *J* = 113.7, 2.5 Hz) ppm.

^{13}C-NMR (75 MHz, $CDCl_3$) δ = 159.9 (dtd, $^1J_{C\text{-}F}$ = 255.5 Hz, $^3J_{C\text{-}F}$ = 3.9 Hz, $^3J_{C\text{-}P}$ = 2.3 Hz; *C*F), 139.3 – 139.1 (m), 136.0 – 135.8 (m), 128.8 (tdd, $^3J_{C\text{-}F}$ = 7.9 Hz, $^3J_{C\text{-}F}$ / $^3J_{C\text{-}P}$ = 2.5, 2.3 Hz), 128.1, 126.7, 124.2 (dd, $^4J_{C\text{-}F}$ = 3.5 Hz, $^4J_{C\text{-}P}$ = 1.3 Hz), 123.5, 119.7 – 118.7 (m), 117.4 (tdd, $^1J_{C\text{-}F}$ = 264.7 Hz, $^1J_{C\text{-}P}$ = 220.9 Hz, $^3J_{C\text{-}F}$ = 2.5 Hz; *C*F_2), 116.8 (dd, $^2J_{C\text{-}F}$ = 23.1 Hz, $^4J_{C\text{-}P}$ = 1.2 Hz), 65.2 (d, $^2J_{C\text{-}P}$ = 6.8 Hz; *C*H_2CH_3), 16.5 (d, $^3J_{C\text{-}P}$ = 5.6 Hz; CH_2*C*H_3) ppm.

HRMS (ESI) *m/z* $[M+H]^+$ calculated for $C_{15}H_{16}ClF_3O_3PS^+$: 399.0193, found: 399.0193.

IR (film): ṽ 3113 (w), 2986 (m), 2917 (w), 2851 (w), 1624 (m), 1572 (m), 1546 (w), 1503 (w), 1428 (m), 1275 (s), 1235 (m), 1164 (m), 1147 (w), 1123 (m), 1042 (s), 1024 (s) cm^{-1}.

UV/Vis (DCM): λ_{max} = 238; 263 nm.

R_f = 0.34 (ethyl acetate/hexane 3:7, *v*/*v*).

{[4-(2-Chlorothiophen-3-yl)-2-fluorophenyl](difluoro)methyl}phosphonic acid (**8j**)

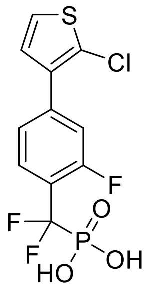

Compound **8j** was prepared from compound **15j** according to **Method 4**. Compound **15j** (12.5 mg, 0.0313 mmol, 1.0 eq.) was stirred with TMS-Br (0.0830 mL, 96.3 mg, 0.629 mmol, 20 eq.) in 1.0 mL of dry DCM for 2 h at 0 °C and then for 46 h at room temperature. The crude product was purified by two successive C_{18} reversed-phase column chromatographies (water to acetonitrile/water 2:3, *v*/*v*) to give **8j** as an off-white solid after lyophilization.

Yield: 10 mg (94 %).

^{1}H-NMR (400 MHz, D_2O) δ = 7.74 – 7.64 (m, 1H), 7.59 – 7.48 (m, 2H), 7.41 (d, *J* = 5.8 Hz, 1H), 7.25 (d, *J* = 5.9 Hz, 1H) ppm.

^{19}F-NMR (377 MHz, D_2O) δ = -106.23 (dd, *J* = 95.3, 18.8 Hz), -112.81 – -113.01 (m) ppm.

^{31}P-NMR (162 MHz, D_2O) δ = 5.20 – 3.47 (m) ppm.

^{13}C-NMR (101 MHz, D_2O) δ = 160.7 – 157.8 (m; *C*F), 137.8 – 137.5 (m), 136.0 – 135.8 (m), 128.6, 128.2, 125.4, 124.0, 123.9 – 123.8 (m), 121.9 – 121.1 (m), 116.5 – 116.0 (m) ppm.

HRMS (ESI) *m/z* $[M-H]^-$ calculated for $C_{11}H_6ClF_3O_3PS^-$: 340.9421, found: 340.9411.

IR (KBr): ṽ 3158 (br), 3012 (m), 2926 (w), 2850 (w), 2834 (w), 1628 (br), 1569 (w), 1542 (w), 1505 (w), 1400 (s), 1192 (m), 1141 (m), 1123 (m) cm^{-1}.
UV/Vis (H_2O): λ_{max} = 202; 235; 261 nm.
$\mathbf{R_f}$ = 0.46 (acetonitrile/water 3:7, *v*/*v*).
m.p.: decomposition at 183 °C.

<u>Diethyl {difluoro[2-fluoro-4-(5-methylthiophen-3-yl)phenyl]methyl}phosphonate (**15k**)</u>

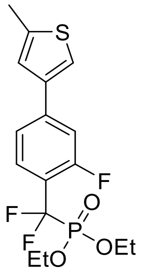

Compound **15k** was synthesized from 4-bromo-2-methylthiophene (**14k**, 72.9 µL, 115 mg, 0.650 mmol, 1.0 eq.), compound **13b** (363 mg, 0.782 mmol, 1.2 eq.), K_3PO_4 (416 mg, 1.96 mmol, 3.0 eq.), $Pd_2(dba)_3$ (3.0 mg, 0.0033 mmol, 0.51 mol%), and SPhos (5.4 mg, 0.013 mmol, L:Pd=4:1) in 3.0 mL of dry toluene according to **Method 1C**. The crude product was purified by flash column chromatography (hexane to ethyl acetate/hexane 3:7, *v*/*v*) to afford **15k** as a light-yellow oil.
Yield: 196 mg (80 %).
^{1}H-NMR (400 MHz, $CDCl_3$) δ = 7.56 – 7.50 (m, 1H), 7.42 – 7.37 (m, 1H), 7.33 – 7.28 (m, 2H), 7.05 – 7.02 (m, 1H), 4.34 – 4.19 (m, 4H; CH_2CH_3), 2.53 (d, J = 1.1 Hz, 3H; CH_3), 1.35 (td, J = 7.1, 0.7 Hz, 6H; CH_2CH_3) ppm.
^{19}F-NMR (376 MHz, $CDCl_3$) δ = -107.04 (dd, $^2J_{F\text{-}P}$ = 114.9 Hz, $^4J_{F\text{-}F}$ = 18.7 Hz, 2F; CF_2), -112.30 – -112.52 (m, 1F; CF) ppm.
^{31}P-NMR (162 MHz, $CDCl_3$) δ = 5.44 (td, $^2J_{P\text{-}F}$ = 114.9 Hz, $^4J_{P\text{-}F}$ = 2.8 Hz) ppm.
^{13}C-NMR (101 MHz, $CDCl_3$) δ = 160.3 (dtd, $^1J_{C\text{-}F}$ = 254.7 Hz, $^3J_{C\text{-}F}$ = 3.9 Hz, $^3J_{C\text{-}P}$ = 2.5 Hz; *C*F), 141.5, 141.1 (dtd, $^3J_{C\text{-}F}$ = 8.6 Hz, $^5J_{C\text{-}F}$ = 1.8 Hz, $^5J_{C\text{-}P}$ = 1.7 Hz), 139.8 – 139.7 (m), 129.0 (tdd, $^3J_{C\text{-}F}$ = 7.9 Hz, $^3J_{C\text{-}F}$ / $^3J_{C\text{-}P}$ = 2.5, 2.3 Hz), 124.3, 121.8 (dd, $^4J_{C\text{-}F}$ = 3.3 Hz, $^4J_{C\text{-}P}$ = 1.3 Hz), 120.2, 118.3 (tdd, $^2J_{C\text{-}F}$ = 22.9 Hz, $^2J_{C\text{-}F}$ / $^2J_{C\text{-}P}$ = 13.5, 11.7 Hz), 117.5 (tdd, $^1J_{C\text{-}F}$ = 264.7 Hz, $^1J_{C\text{-}P}$ = 221.6 Hz, $^3J_{C\text{-}F}$ = 2.5 Hz; CF_2), 114.3 (dd, $^2J_{C\text{-}F}$ = 22.7 Hz, $^4J_{C\text{-}P}$ = 1.4 Hz), 65.1 (d, $^2J_{C\text{-}P}$ = 6.8 Hz; CH_2CH_3), 16.5 (d, $^3J_{C\text{-}P}$ = 5.6 Hz; CH_2CH_3), 15.5 (CH_3) ppm.
HRMS (ESI) *m*/*z* $[M+H]^+$ calculated for $C_{16}H_{19}F_3O_3PS^+$: 379.0739, found: 379.0740.
IR (film): ṽ 3092 (w), 2985 (m), 2924 (m), 2868 (w), 1624 (s), 1574 (m), 1552 (w), 1513 (m), 1436 (m), 1276 (s), 1237 (m), 1142 (s), 1042 (s), 1021 (s), 824 (m), 801 (m), 748 (m) cm^{-1}.
UV/Vis (DCM): λ_{max} = 241; 270 nm.
$\mathbf{R_f}$ = 0.31 (ethyl acetate/hexane 3:7, *v*/*v*).

{Difluoro[2-fluoro-4-(5-methylthiophen-3-yl)phenyl]methyl}phosphonic acid (**8k**)

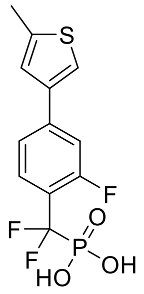

Compound **8k** was prepared from compound **15k** according to **Method 4**. Compound **15k** (64.2 mg, 0.170 mmol, 1.0 eq.) was stirred with TMS-Br (0.450 mL, 522 mg, 3.41 mmol, 20 eq.) in 2.6 mL of dry DCM for 2 h at 0 °C and then for 46 h at room temperature. The crude product was purified by C_{18} reversed-phase column chromatography (water to acetonitrile/water 1:9, *v*/*v*) to afford **8k** as a colorless solid after lyophilization.

Yield: 55 mg (quant.)

^{1}H-NMR (400 MHz, D_2O+CD_3CN, *v*/*v* = 1:1) δ = 8.18 – 8.12 (m, 1H), 8.11 – 8.06 (m, 2H), 8.03 – 7.98 (m, 1H), 7.80 – 7.76 (m, 1H), 3.08 (d, *J* = 1.1 Hz, 3H; C*H*$_3$) ppm.

^{19}F-NMR (377 MHz, D_2O+CD_3CN, *v*/*v* = 1:1) δ = -106.45 (dd, $^{2}J_{F-P}$ = 96.2 Hz, $^{4}J_{F-F}$ = 19.4 Hz, 2F; CF_2), -113.43 – -113.65 (m; 1F; CF) ppm.

^{31}P-NMR (162 MHz, D_2O+CD_3CN, *v*/*v* = 1:1) δ = 2.80 (t, $^{2}J_{P-F}$ = 96.1 Hz) ppm.

^{13}C-NMR (101 MHz, D_2O+CD_3CN, *v*/*v* = 1:1) δ = 162.3 – 159.5 (m; *C*F), 142.4, 140.7 – 140.5 (m), 140.1 – 139.8 (m), 130.5 – 130.2 (m), 125.2, 123.0 – 122.3 (m), 122.2 – 122.0 (m), 121.1, 124.9 – 117.2 (m; *C*F$_2$), 114.7 – 114.3 (m), 15.4 (*C*H$_3$) ppm.

HRMS (ESI) *m*/*z* $[M-H]^-$ calculated for $C_{12}H_9F_3O_3PS^-$: 320.9968, found: 320.9962.

IR (KBr): ṽ 3185 (br), 3013 (w), 2920 (w), 2854 (w), 1622 (m), 1573 (m), 1552 (w), 1513 (w), 1431 (m), 1400 (s), 1233 (m), 1147 (m), 823 (m), 800 (m), 749 (m) cm^{-1}.

UV/Vis (H_2O): λ_{max} = 204; 237; 268 nm.

R_f = 0.29 (acetonitrile/water 1:4, *v*/*v*).

m.p.: decomposition at 210 °C.

Diethyl {difluoro[2-fluoro-4-(2-methylthiophen-3-yl)phenyl]methyl}phosphonate (**15l**)

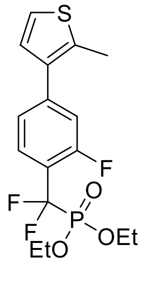

Compound **15l** was synthesized from 3-bromo-2-methylthiophene (**14l**, 16.2 µL, 25.7 mg, 0.145 mmol, 1.0 eq.), compound **13b** (94.4 mg, 0.203 mmol, 1.4 eq.), K_3PO_4 (93.4 mg, 0.440 mmol, 3.0 eq.), $Pd_2(dba)_3$ (1.3 mg, 0.0014 mmol, 1.0 mol%), and SPhos (2.4 mg, 0.0058 mmol, L:Pd=4:1) in 0.90 mL of dry toluene according to **Method 1C**. The crude product was purified by two successive flash column chromatographies (hexane to ethyl acetate/hexane 1:4, *v*/*v*, then hexane to acetone/hexane 1:19, *v*/*v*) to give **15l** as a light-yellow oil.

Yield: 43 mg (78 %).

^{1}H-NMR (400 MHz, $(CD_3)_2CO$)) δ = 7.67 – 7.61 (m, 1H), 7.46 – 7.41 (m, 1H), 7.39 – 7.34 (m, 1H), 7.32 (d, J = 5.3 Hz, 1H), 7.18 (d, J = 5.3 Hz, 1H), 4.32 – 4.17 (m, 4H; CH_2CH_3), 2.56 (s, 3H; CH_3), 1.32 (td, J = 7.1, 0.6 Hz, 6H; CH_2CH_3) ppm.

^{19}F-NMR (377 MHz, $(CD_3)_2CO$) δ = -108.37 (dd, $^2J_{F\text{-}P}$ = 111.9 Hz, $^4J_{F\text{-}F}$ = 19.7 Hz, 2F; CF_2), -114.23 – -114.47 (m, 1F; CF) ppm.

^{31}P-NMR (162 MHz, $(CD_3)_2CO$) δ = 3.94 (td, $^2J_{P\text{-}F}$ = 112.1 Hz, $^4J_{P\text{-}F}$ = 2.6 Hz) ppm.

^{13}C-NMR (76 MHz, $(CD_3)_2CO$) δ = 160.5 (dtd, $^1J_{C\text{-}F}$ = 254.2 Hz, $^3J_{C\text{-}F}$ = 3.8 Hz, $^3J_{C\text{-}P}$ = 2.5 Hz; *C*F), 142.9 (dtd, $^3J_{C\text{-}F}$ = 8.7 Hz, $^5J_{C\text{-}F}$ = 1.7 Hz, $^5J_{C\text{-}P}$ = 1.7 Hz), 137.0 – 136.9 (m), 136.8, 129.6, 129.6 (tdd, $^3J_{C\text{-}F}$ = 7.9 Hz, $^3J_{C\text{-}F}$ / $^3J_{C\text{-}P}$ = 2.5, 2.3 Hz), 125.2 (dd, $^4J_{C\text{-}F}$ = 3.4 Hz, $^4J_{C\text{-}P}$ = 1.2 Hz), 123.4, 119.2 (tdd, $^2J_{C\text{-}F}$ = 22.7 Hz, $^2J_{C\text{-}F}$ / $^2J_{C\text{-}P}$ = 13.7, 11.5 Hz), 118.7 (tdd, $^1J_{C\text{-}F}$ = 263.4 Hz, $^1J_{C\text{-}P}$ = 220.1 Hz, $^3J_{C\text{-}F}$ = 2.5 Hz; CF_2), 117.3 (dd, $^2J_{C\text{-}F}$ = 22.5 Hz, $^4J_{C\text{-}P}$ = 1.1 Hz), 65.5 (d, $^2J_{C\text{-}P}$ = 6.7 Hz; CH_2CH_3), 16.7 (d, $^3J_{C\text{-}P}$ = 5.5 Hz; CH_2CH_3), 14.3 (CH_3) ppm.

HRMS (ESI) *m*/*z* $[M+H]^+$ calculated for $C_{16}H_{19}F_3O_3PS^+$: 379.0739, found: 379.0741.

IR (film): ṽ 3104 (w), 2985 (w), 2919 (w), 2867 (w), 1624 (m), 1571 (m), 1547 (w), 1505 (w), 1274 (s), 1235 (m) 1043 (s), 1020 (s) cm^{-1}.

UV/Vis (DCM): λ_{max} = 236; 263 nm.

R_f = 0.31 (ethyl acetate/hexane 3:7, *v*/*v*).

{Difluoro[2-fluoro-4-(2-methylthiophen-3-yl)phenyl]methyl}phosphonic acid (**8l**)

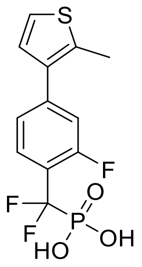

Compound **8l** was prepared from compound **15l** according to **Method 4**. Compound **15l** (22.1 mg, 0.0584 mmol, 1.0 eq.) was stirred with TMS-Br (0.154 mL, 179 mg, 1.17 mmol, 20 eq.) in 1.1 mL of dry DCM for 2 h at 0 °C and then for 46 h at room temperature. The crude product was purified by C_{18} reversed-phase column chromatography (water to acetonitrile/water 1:9, *v*/*v*) to afford **8l** as an off-white solid after lyophilization.

Yield: 19 mg (quant.)

^{1}H-NMR (400 MHz, D_2O+CD_3CN, *v*/*v* = 1:1) δ = 8.09 – 8.02 (m, 1H), 7.75 – 7.70 (m, 1H), 7.69 – 7.62 (m, 2H), 7.57 (d, *J* = 5.3 Hz, 1H), 2.94 (s, 3H; C*H*$_3$) ppm.

^{19}F-NMR (377 MHz, D_2O+CD_3CN, *v*/*v* = 1:1) δ = -104.99 (dd, $^2J_{F\text{-}P}$ = 91.7 Hz, $^4J_{F\text{-}F}$ = 19.6 Hz, 2F; CF_2), -112.74 – -112.95 (m, 1F; CF) ppm.

^{31}P-NMR (162 MHz, D_2O+CD_3CN, *v*/*v* = 1:1) δ = 4.33 (t, $^2J_{P\text{-}F}$ = 91.7 Hz) ppm.

^{13}C-NMR (101 MHz, D_2O+CD_3CN, *v*/*v* = 1:1) δ = 161.3 – 158.6 (m; *C*F), 140.4 – 140.2 (m), 136.9 – 136.8 (m), 136.2, 129.6 – 129.3 (m), 129.4, 124.3 – 124.1 (m), 122.9, 122.7 – 121.9 (m), 124.8 – 117.6 (m; *C*F$_2$), 116.7 – 116.3 (m), 14.0 (*C*H$_3$) ppm.

HRMS (ESI) *m*/*z* [M-H]$^-$ calculated for $C_{12}H_9F_3O_3PS^-$: 320.9968, found: 320.9968.

IR (KBr): ṽ 3125 (br), 2924 (w), 2857 (w), 2799 (w), 1626 (m), 1569 (w), 1551 (w), 1504 (w), 1402 (s), 1231 (w), 1126 (m) cm^{-1}.

UV/Vis (H_2O): λ_{max} = 202; 232; 258 nm.

R$_f$ = 0.29 (acetonitrile/water 1:4, *v*/*v*).

m.p.: decomposition at 205 °C.

<u>Diethyl {difluoro[2-fluoro-4-(4-methylthiophen-3-yl)phenyl]methyl}phosphonate (**15m**)</u>

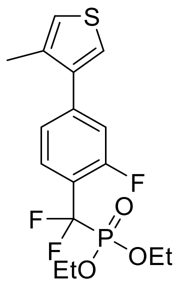

Compound **15m** was synthesized from 3-bromo-4-methylthiophene (**14m**, 16.5 µL, 26.1 mg, 0.147 mmol, 1.0 eq.), compound **13b** (95.8 mg, 0.206 mmol, 1.4 eq.), K_3PO_4 (94.6 mg, 0.446 mmol, 3.0 eq.), $Pd_2(dba)_3$ (1.3 mg, 0.0014 mmol, 1.0 mol%), and SPhos (2.4 mg, 0.0058 mmol, L:Pd=4:1) in 0.90 mL of dry toluene according to **Method 1C**. The crude product was purified by two successive flash column chromatographies (hexane to ethyl acetate/hexane 1:4, *v*/*v*, then hexane to acetone/hexane 1:19, *v*/*v*) to give **15m** as a light-yellow oil.

Yield: 44 mg (79 %).

^{1}H-NMR (400 MHz, $(CD_3)_2CO$) δ = 7.66 – 7.60 (m, 1H), 7.58 (d, *J* = 3.3 Hz, 1H), 7.46 – 7.42 (m, 1H), 7.39 – 7.34 (m, 1H), 7.27 – 7.24 (m, 1H), 4.32 – 4.17 (m, 4H; C*H*$_2$CH$_3$), 2.31 (d, *J* = 1.1 Hz, 3H; C*H*$_3$), 1.32 (td, *J* = 7.0, 0.6 Hz, 6H; CH$_2$C*H*$_3$) ppm.

^{19}F-NMR (377 MHz, $(CD_3)_2CO$) δ = -108.40 (dd, $^2J_{F\text{-}P}$ = 111.9 Hz, $^4J_{F\text{-}F}$ = 19.8 Hz, 2F; CF_2), -114.33 – -114.57 (m, 1F; CF) ppm.

^{31}P-NMR (162 MHz, $(CD_3)_2CO$) δ = 3.93 (td, $^2J_{P\text{-}F}$ = 111.9 Hz, $^4J_{P\text{-}F}$ = 2.5 Hz) ppm.

^{13}C-NMR (101 MHz, $(CD_3)_2CO$) δ = 160.6 (dtd, $^1J_{C-F}$ = 254.2 Hz, $^3J_{C-F}$ = 3.8 Hz, $^3J_{C-P}$ = 2.5 Hz; *C*F), 143.3 (dtd, $^3J_{C-F}$ = 8.7 Hz, $^5J_{C-F}$ = 1.8 Hz, $^5J_{C-P}$ = 1.8 Hz), 141.3 – 141.2 (m), 136.5, 129.5 (tdd, $^3J_{C-F}$ = 7.9 Hz, $^3J_{C-F}$ / $^3J_{C-P}$ = 2.5, 2.3 Hz), 125.8, 125.2 (dd, $^4J_{C-F}$ = 3.4 Hz, $^4J_{C-P}$ = 1.2 Hz), 124.0, 119.5 (tdd, $^2J_{C-F}$ = 22.7 Hz, $^2J_{C-F}$ / $^2J_{C-P}$ = 13.4, 11.3 Hz), 119.0 (tdd, $^1J_{C-F}$ = 263.6 Hz, $^1J_{C-P}$ = 220.2 Hz, $^3J_{C-F}$ = 2.5 Hz; $\mathit{C}F_2$), 117.3 (dd, $^2J_{C-F}$ = 22.5 Hz, $^4J_{C-P}$ = 1.1 Hz), 65.5 (d, $^2J_{C-P}$ = 6.7 Hz; $\mathit{C}H_2CH_3$), 16.7 (d, $^3J_{C-P}$ = 5.3 Hz; $CH_2\mathit{C}H_3$), 15.6 ($\mathit{C}H_3$) ppm.

HRMS (ESI) *m/z* $[M+H]^+$ calculated for $C_{16}H_{19}F_3O_3PS^+$: 379.0739, found: 379.0740.

IR (film): ṽ 3095 (w), 2985 (m), 2928 (w), 1625 (m), 1571 (m), 1539 (w), 1508 (w), 1276 (s), 1234 (m), 1139 (m), 1044 (s), 1020 (s), 797 (m) cm^{-1}.

UV/Vis (DCM): λ_{max} = 234; 262 nm.

R_f = 0.30 (ethyl acetate/hexane 3:7, *v*/*v*).

{Difluoro[2-fluoro-4-(4-methylthiophen-3-yl)phenyl]methyl}phosphonic acid (**8m**)

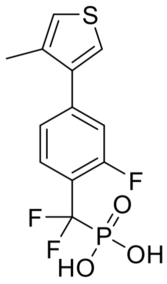

Compound **8m** was prepared from compound **15m** according to **Method 4**. Compound **15m** (19.7 mg, 0.0521 mmol, 1.0 eq.) was stirred with TMS-Br (0.138 mL, 160 mg, 1.05 mmol, 20 eq.) in 1.1 mL of dry DCM for 2 h at 0 °C and then for 46 h at room temperature. The crude product was purified by C_{18} reversed-phase column chromatography (water to acetonitrile/water 1:9, *v*/*v*) to afford **8m** as a colorless solid after lyophilization.

Yield: 17 mg (quant.)

^{1}H-NMR (400 MHz, D_2O+CD_3CN, *v*/*v* = 1:1) δ = 8.08 – 8.02 (m, 1H), 7.88 (d, *J* = 3.3 Hz, 1H), 7.75 – 7.70 (m, 1H), 7.68 – 7.62 (m, 1H), 7.61 – 7.58 (m, 1H), 2.70 (d, *J* = 1.0 Hz, 3H; $C\mathit{H}_3$) ppm.

^{19}F-NMR (377 MHz, D_2O+CD_3CN, *v*/*v* = 1:1) δ = -104.94 (dd, $^2J_{F-P}$ = 91.4 Hz, $^4J_{F-F}$ = 19.7 Hz, 2F; CF_2), -112.80 – -112.99 (m, 1F; CF) ppm.

^{31}P-NMR (162 MHz, D_2O+CD_3CN, *v*/*v* = 1:1) δ = 4.35 (t, $^2J_{P-F}$ = 91.2 Hz) ppm.

^{13}C-NMR (101 MHz, D_2O+CD_3CN, *v*/*v* = 1:1) δ = 161.2 – 158.5 (m; *C*F), 141.3 – 141.2 (m), 140.7 – 140.5 (m), 136.5, 129.6 – 129.3 (m), 124.9, 124.2 – 124.1 (m), 123.5, 123.1 – 122.3 (m), 124.8 – 117.7 (m; $\mathit{C}F_2$), 116.7 – 116.3 (m), 15.2 ($\mathit{C}H_3$) ppm.

HRMS (ESI) *m/z* $[M-H]^-$ calculated for $C_{12}H_9F_3O_3PS^-$: 320.9968, found: 320.9970.

IR (KBr): ṽ 3124 (br), 3017 (w), 2926 (w), 2852 (w), 1627 (m), 1568 (w), 1541 (w), 1598 (w), 1401 (m), 1231 (w), 1133 (m), 1111 (w), 793 (w) cm^{-1}.

UV/Vis (H_2O): λ_{max} = 203; 249; 281 nm.

R$_f$ = 0.29 (acetonitrile/water 1:4, *v*/*v*).
m.p.: decomposition at 211 °C

Iodomethyl pivalate[16]

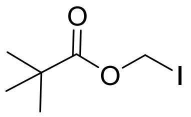

Synthesis of iodomethyl pivalate was carried our essentially as described in the literature.[16] Sodium iodide (576 mg, 3.84 mmol, 1.8 eq.) was added to a solution of chloromethyl pivalate (306 µL, 320 mg, 2.12 mmol, 1.0 eq.) dissolved in 0.90 mL of dry acetonitrile. The suspension was then heated to 55 °C for 5 h under inert gas atmosphere. Once cool, the reaction mixture was transferred to 100 mL of H_2O/DCM (1:1, *v*/*v*) and stirred for 10 min. The organic and aqueous phases were separated, and the organic phase was washed twice with 50 mL of an aqueous 2 % $Na_2S_2O_3$ solution, before being dried over Na_2SO_4, filtered, and evaporated under reduced pressure. Iodomethyl pivalate was obtained as a yellow oil.
Yield: 486 mg (94 %).
^{1}H-NMR (300 MHz, $CDCl_3$) δ = 5.93 (s, 2H; OC*H*$_2$I), 1.18 (s, 9H; C(C*H*$_3$)$_3$) ppm.
^{13}C-NMR (101 MHz, $CDCl_3$) δ = 176.5 (*C*O), 39.0 (*C*(CH$_3$)$_3$), 31.5 (O*C*H$_2$I), 26.7 (C(*C*H$_3$)$_3$) ppm.
R$_f$ = 0.66 (ethyl acetate/hexane 1:9, *v*/*v*).

Prodrug **9**

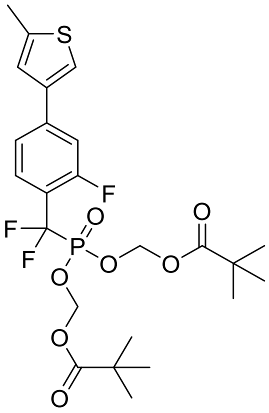

Synthesis of **9** was carried out analogous to the published procedure.[17] Phosphonate **8k** (48.4 mg, 0.150 mmol, 1.0 eq.) was suspended in 0.350 mL of water, to which NaOH (12.0 mg, 0.300 mmol, 2.0 eq.) dissolved in 0.150 mL of water was added dropwise, turning the suspension clear. After being stirred for 1 h, $AgNO_3$ (56.3 mg, 0.331 mmol, 2.2 eq.) was introduced, and the mixture was stirred again for 2 h at 0 °C. The light-gray precipitate was filtered and then dried under high vacuum overnight, yielding 67.5 mg of silver salt. A *Schlenk* flask was charged with the silver salt (maximum of 63.4 mg, 0.118 mmol, 1.0 eq.) and 1.8 mL of dry toluene under inert gas atmosphere, and the suspension was sonicated for 1 min.

Iodomethyl pivalate (88.7 µL, 143 mg, 0.591 mmol, 5.0 eq.) was then added, and the reaction mixture was stirred at room temperature for 20 h, before being diluted with acetone, filtered, and evaporated under reduced pressure. To eliminate the excess iodomethyl pivalate, the concentrate was dried under high vacuum. The resulting residue was purified by two successive flash column chromatography (ethyl acetate/hexane 1:4, *v/v*) to give **9** as an off-white solid.

Yield: 43 mg (52 % over two steps).

^{1}H-NMR (400 MHz, $(CD_3)_2CO$) δ = 7.73 (d, *J* = 1.4 Hz, 1H), 7.68 – 7.63 (m, 1H), 7.61 – 7.54 (m, 2H), 7.32 – 7.30 (m, 1H), 5.84 – 5.74 (m, 4H; OCH_2O), 2.53 (d, *J* = 1.1 Hz, 3H; CH_3), 1.21 (s, 18H; (CH_3)$_3$) ppm.

^{19}F-NMR (377 MHz, $(CD_3)_2CO$) δ =-108.85 (dd, $^2J_{F\text{-}P}$ = 121.4 Hz, $^4J_{F\text{-}F}$ = 19.8 Hz, 2F; CF_2), -113.78 – -114.03 (m, 1F; CF) ppm.

^{31}P-NMR (162 MHz, $(CD_3)_2CO$) δ = 2.70 (td, $^2J_{P\text{-}F}$ = 121.5 Hz, $^4J_{P\text{-}F}$ = 2.7 Hz) ppm.

^{13}C-NMR (76 MHz, $(CD_3)_2CO$) δ = 176.8 (*C*O), 162.9 – 159.3 (m), 142.5 – 142.3 (m), 142.1, 140.1 – 140.0 (m), 129.9 (tdd, $^3J_{C\text{-}F}$ = 7.9 Hz, $^3J_{C\text{-}F}$ = 2.4 Hz, $^3J_{C\text{-}P}$ = 2.4 Hz), 125.2, 122.7 (dd, $^4J_{C\text{-}F}$ = 3.2 Hz, $^4J_{C\text{-}P}$ = 1.2 Hz), 122.0, 117.9 (tdd, $^2J_{C\text{-}F}$ = 22.3 Hz, $^2J_{C\text{-}F}$ / $^2J_{C\text{-}P}$ = 14.3, 11.9 Hz), 114.9 (dd, $^2J_{C\text{-}F}$ = 22.8 Hz, $^4J_{C\text{-}P}$ = 1.0 Hz), 83.5 (d, $^2J_{C\text{-}P}$ = 6.5 Hz; OCH_2O), 39.3 (*C*(CH_3)$_3$), 27.0 (C(CH_3)$_3$), 15.3 (CH_3) ppm.

HRMS (ESI) *m/z* $[M+Na]^+$ calculated for $C_{24}H_{30}F_3O_7PSNa^+$: 573.1294, found: 573.1296.

IR (KBr): ṽ 2984 (m), 2975 (m), 2923 (w), 2874 (w), 1748 (s), 1625 (m), 1574 (w), 1546 (w), 1515 (w), 1303 (m), 1285 (s), 1240 (m), 1156 (s), 1142 (s), 999 (s), 977 (s) cm^{-1}.

UV/Vis (DCM): λ_{max} = 240; 270 nm.

R$_f$ = 0.39 (ethyl acetate/hexane 1:4, *v/v*).

m.p.: 70 – 72 °C.

Prodrug **10**

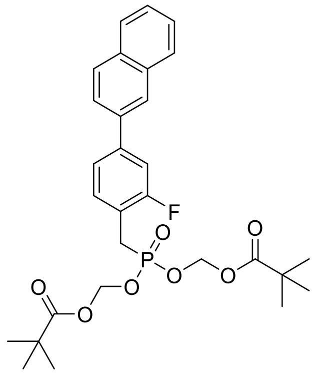

A solution of compound **7** (51.2 mg, 0.162 mmol, 1.0 eq.) was prepared in 3.2 mL of dry DMF, to which DIPEA (111 µL, 83.7 mg, 0.648 mmol, 4.0 eq.) was added. After being stirred for 20 min, iodomethyl pivalate (107 µL, 173 mg, 0.715 mmol, 4.4 eq.) was introduced, and the

mixture was stirred under an inert gas atmosphere for 16 h at room temperature. The volatiles were then evaporated *in vacuo*, and the resulting residue was chromatographed twice on silica gel (ethyl acetate in hexane 1:4, *v*/*v*) to afford **10** as an off-white solid.

Yield: 18 mg (20 %).

^{1}H-NMR (400 MHz, $(CD_3)_2CO$) δ = 8.24 (d, *J* = 1.9 Hz, 1H), 8.03 – 7.92 (m, 3H), 7.85 (dd, *J* = 8.6, 1.9 Hz, 1H), 7.66 – 7.50 (m, 5H), 5.74 – 5.65 (m, 4H; OCH_2O), 3.42 (d, *J* = 22.3 Hz, 2H; CH_2P), 1.20 (s, 18H; $(CH_3)_3$) ppm.

^{19}F-NMR (377 MHz, $(CD_3)_2CO$) δ = -118.42 – -118.57 (m) ppm.

^{31}P-NMR (162 MHz, $(CD_3)_2CO$) δ = 23.99 (d, $^{4}J_{P\text{-}F}$ = 4.8 Hz) ppm.

^{13}C-NMR (101 MHz, $(CD_3)_2CO$) δ = 177.1 (*C*O), 162.2 (dd, $^{1}J_{C\text{-}F}$ = 245.9 Hz, $^{3}J_{C\text{-}P}$ = 7.9 Hz; *C*F), 143.0 (dd, $^{3}J_{C\text{-}F}$ = 7.9 Hz, $^{5}J_{C\text{-}P}$ = 4.0 Hz), 137.44 – 137.38 (m), 134.7, 134.0, 133.4 (dd, $^{3}J_{C\text{-}F}$ / $^{3}J_{C\text{-}P}$ = 5.5, 4.2 Hz), 129.5, 129.2, 128.5, 127.4, 127.2, 126.6 (d, $^{5}J_{C\text{-}F}$ = 1.0 Hz), 125.8 (d, $^{5}J_{C\text{-}F}$ = 0.8 Hz), 123.8 – 123.7 (m), 118.4 (dd, $^{2}J_{C\text{-}F}$ = 16.1 Hz, $^{2}J_{C\text{-}P}$ = 9.9 Hz), 114.7 (dd, $^{2}J_{C\text{-}F}$ = 23.3 Hz, $^{4}J_{C\text{-}P}$ = 3.2 Hz), 82.5 (d, $^{2}J_{C\text{-}P}$ = 6.3 Hz; OCH_2O), 39.3 (*C*$(CH_3)_3$), 27.3 (dd, $^{1}J_{C\text{-}P}$ = 140.6 Hz, $^{3}J_{C\text{-}F}$ = 2.8 Hz; *C*H_2P), 27.1 (C(CH_3)$_3$) ppm.

HRMS (ESI) *m*/*z* $[M+Na]^+$ calculated for $C_{29}H_{34}FO_7PNa^+$: 567.1918, found: 567.1918.

IR (KBr): ṽ 3055 (w), 2978 (m), 2929 (m), 2873 (w), 2852 (w), 1758 (s), 1626 (w), 1598 (w), 1567 (w), 1282 (m), 1260 (m), 1143 (s), 1001 (m), 970 (s) cm^{-1}.

UV/Vis (DCM): λ_{max} = 228; 256; 292 nm.

R_f = 0.27 (ethyl acetate/hexane 3:7, *v*/*v*).

m.p.: 62 – 63 °C.

NMR spectra

^{1}H NMR of compound **1**

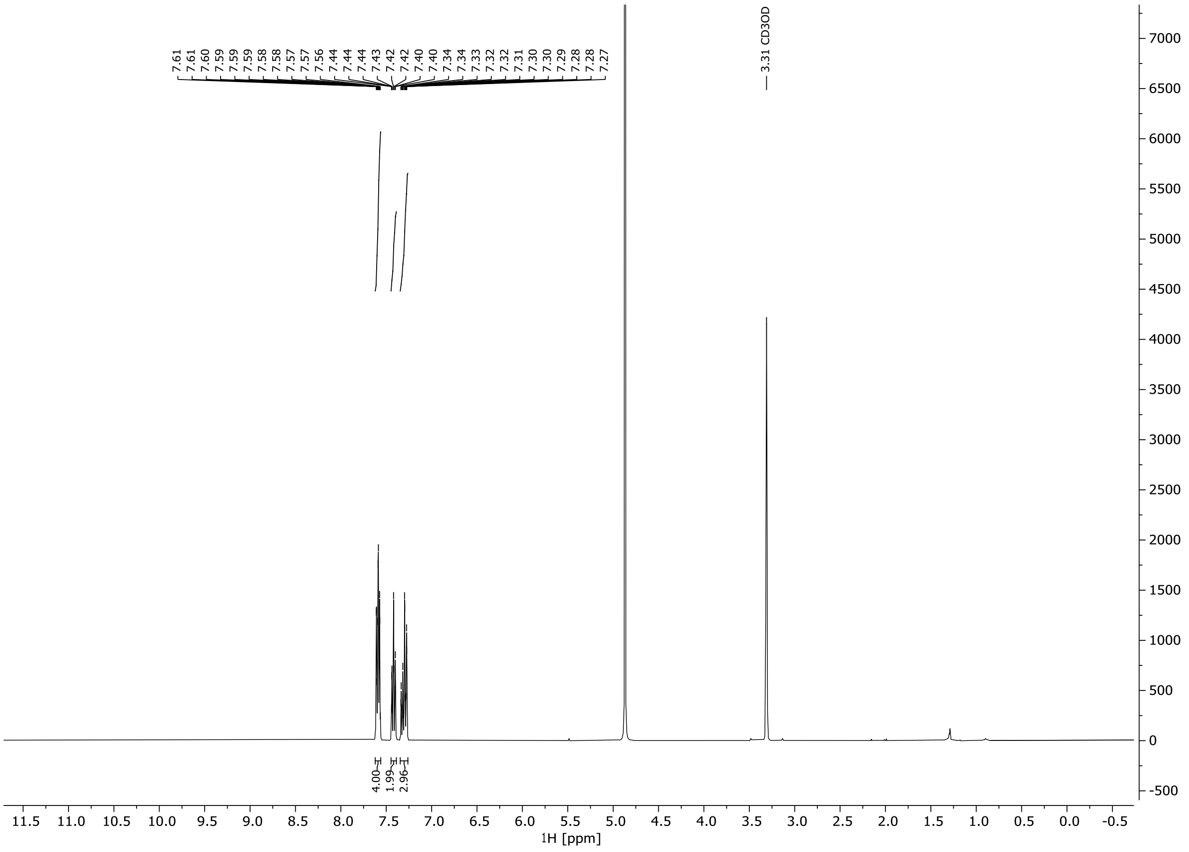

^{13}P NMR of compound **1**

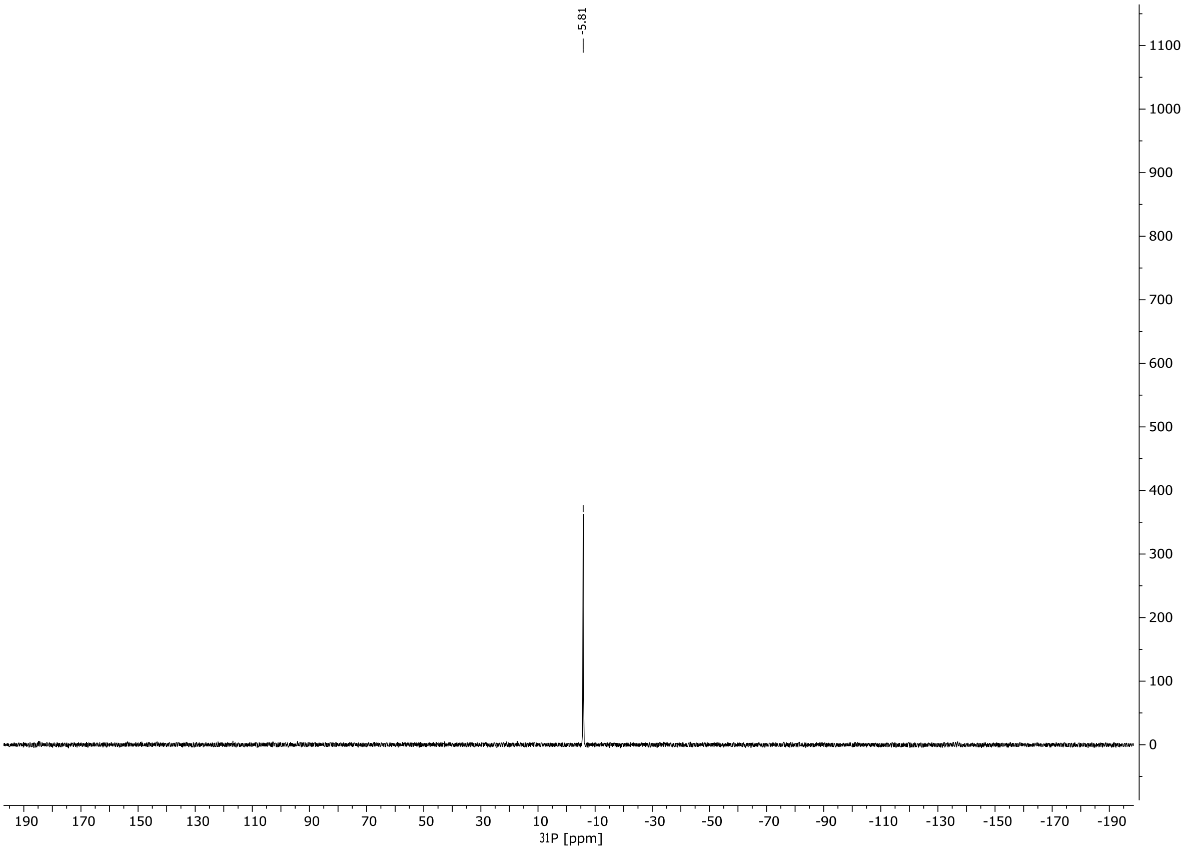

^{13}C NMR of compound **1**

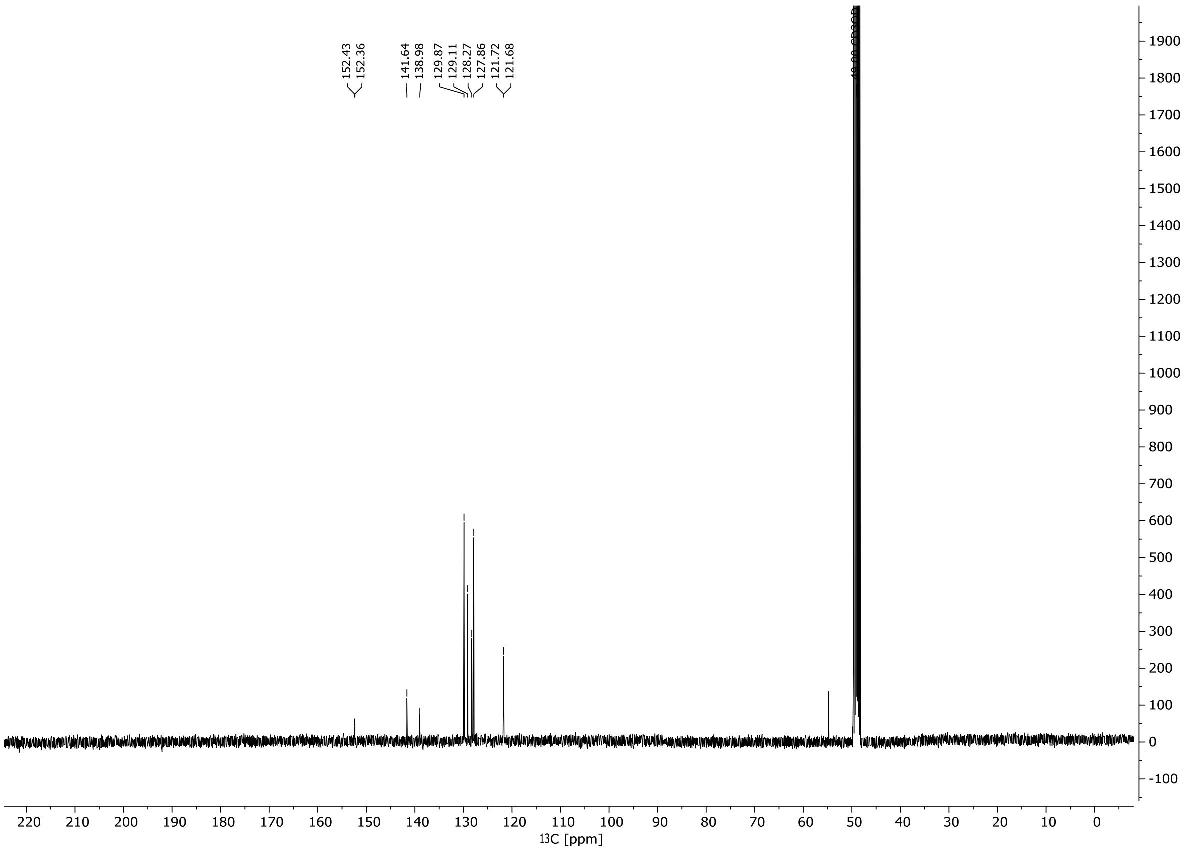

^{1}H NMR of compound **6a**

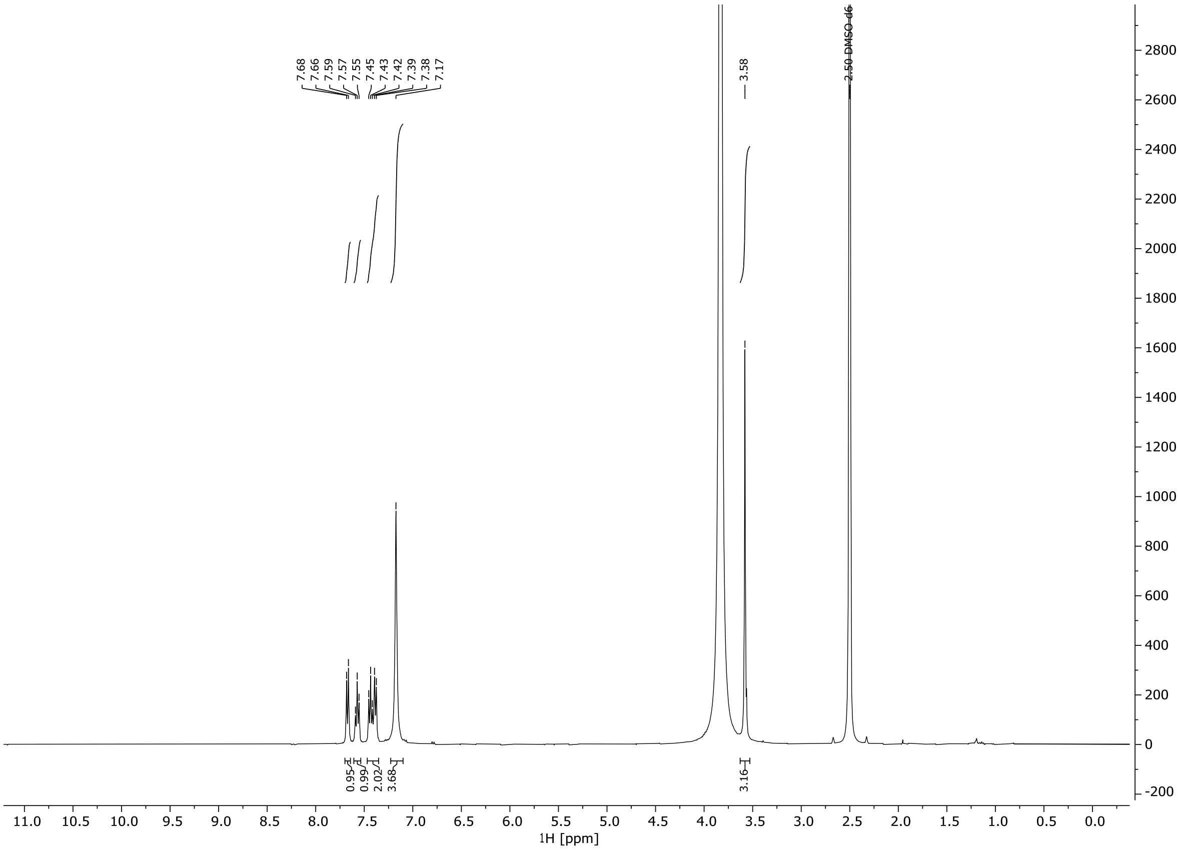

^{31}P NMR of compound **6a**

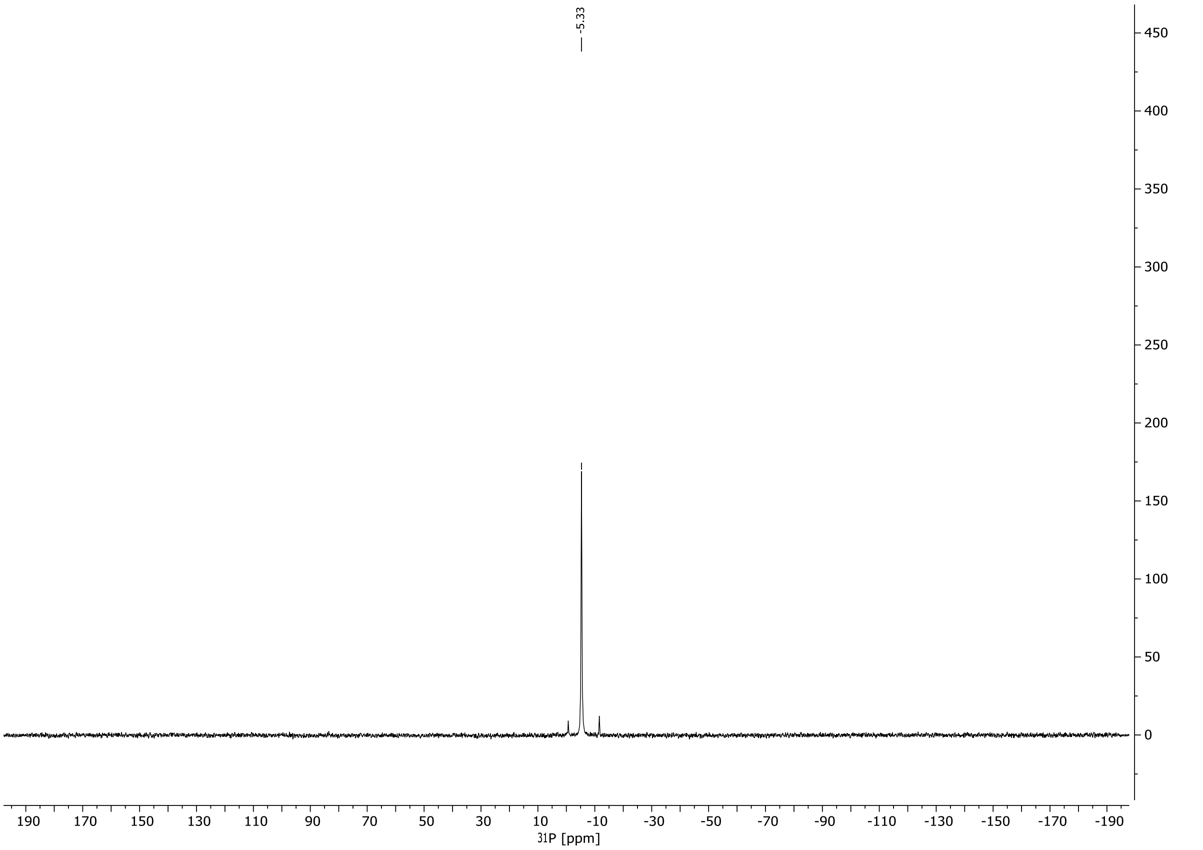

^{13}C NMR of compound **6a**

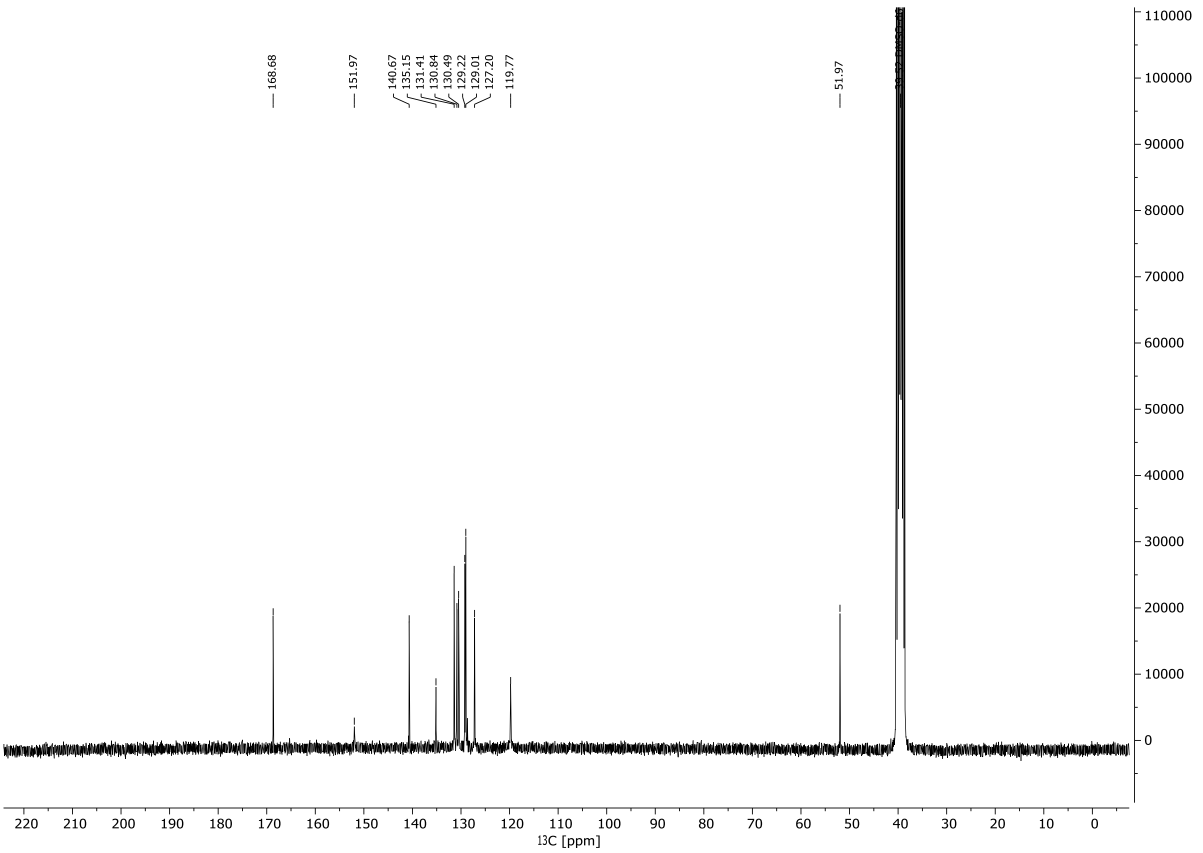

^{1}H NMR of compound **6b**

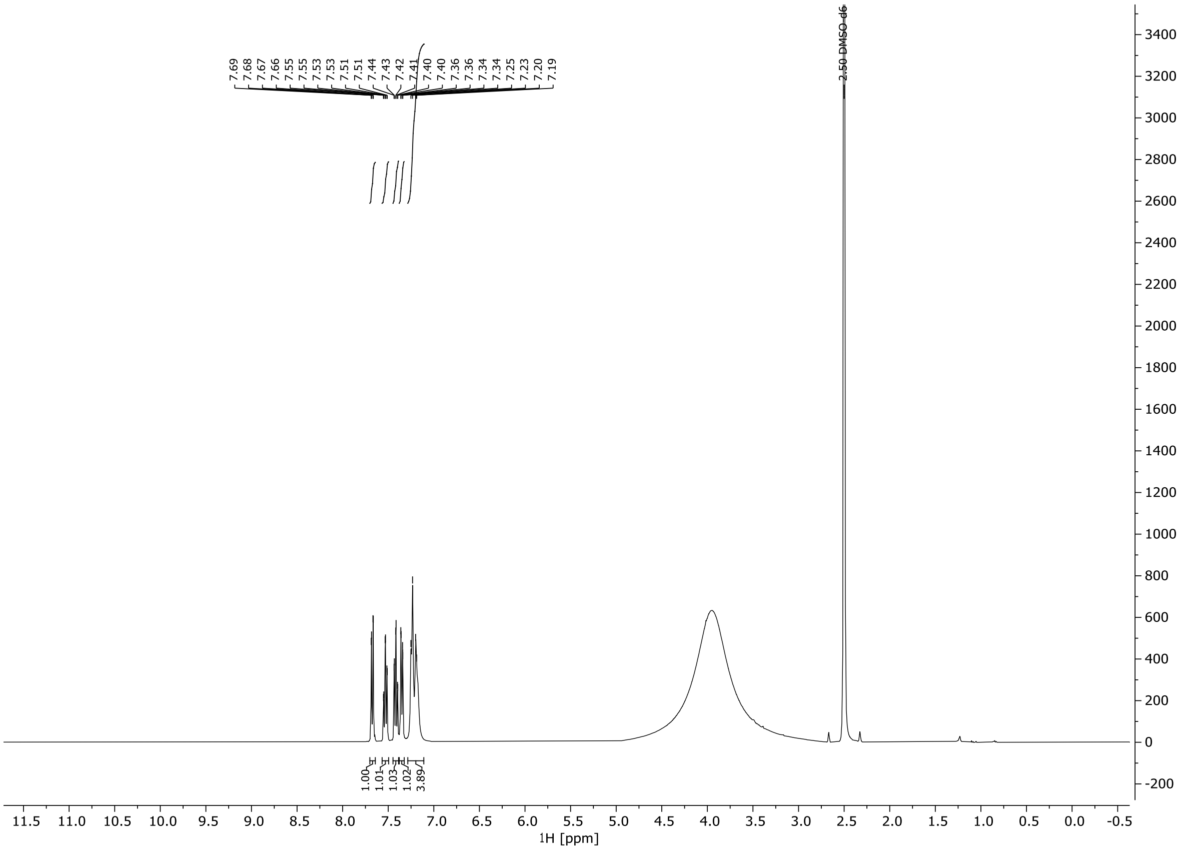

^{31}P NMR of compound **6b**

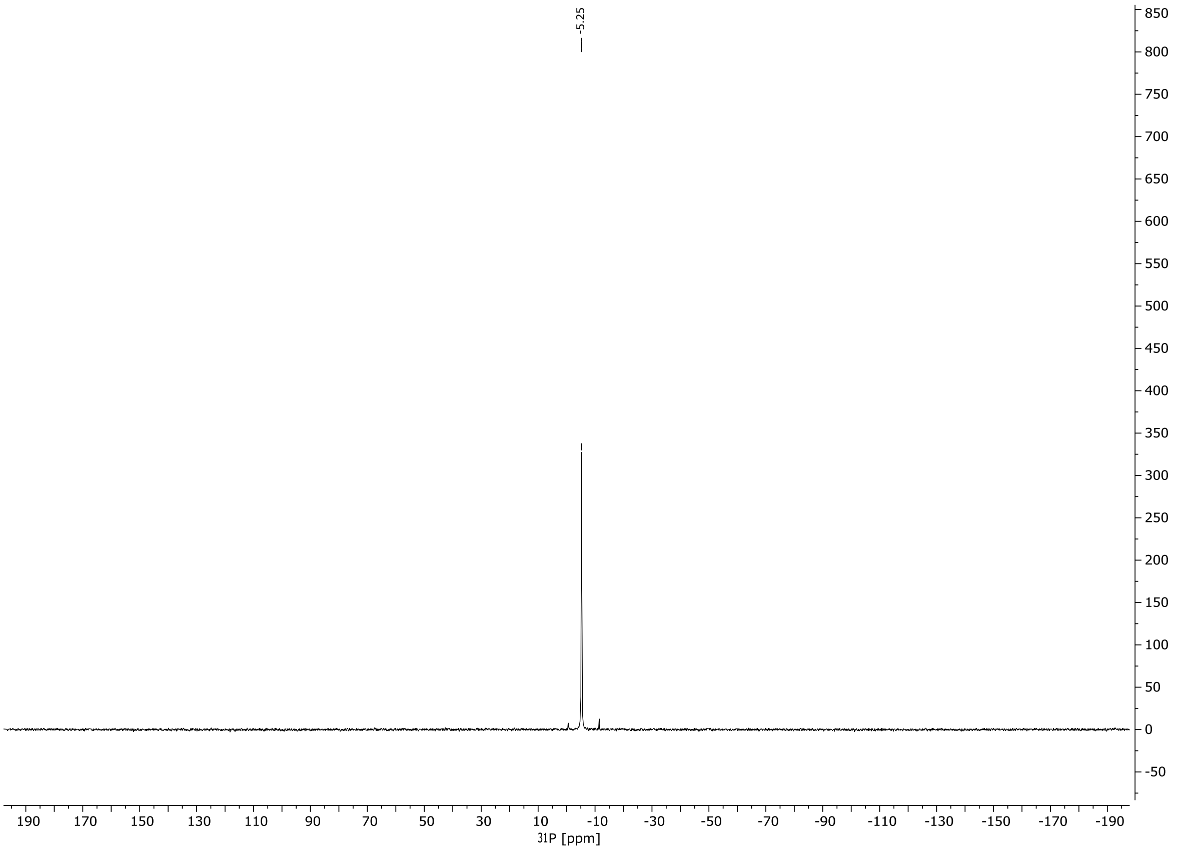

^{13}C NMR of compound **6b**

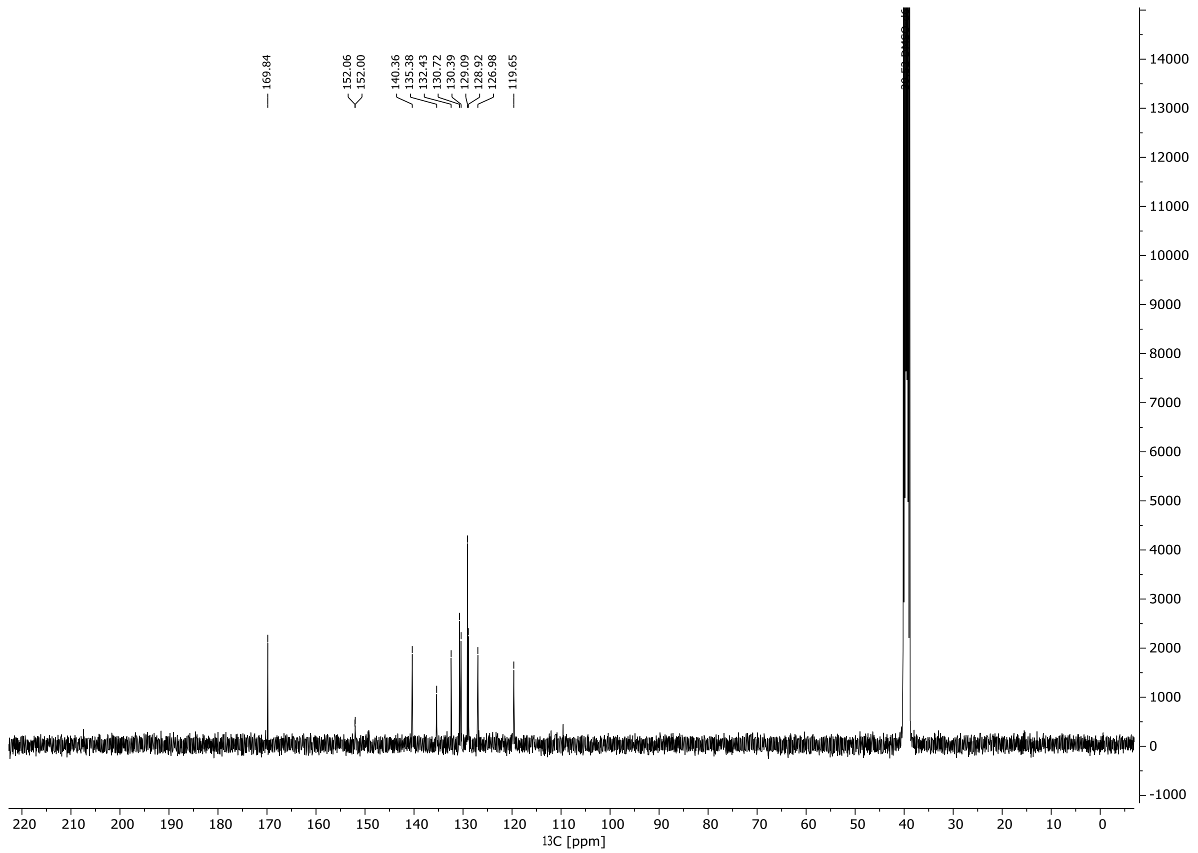

^{1}H NMR of compound **6c**

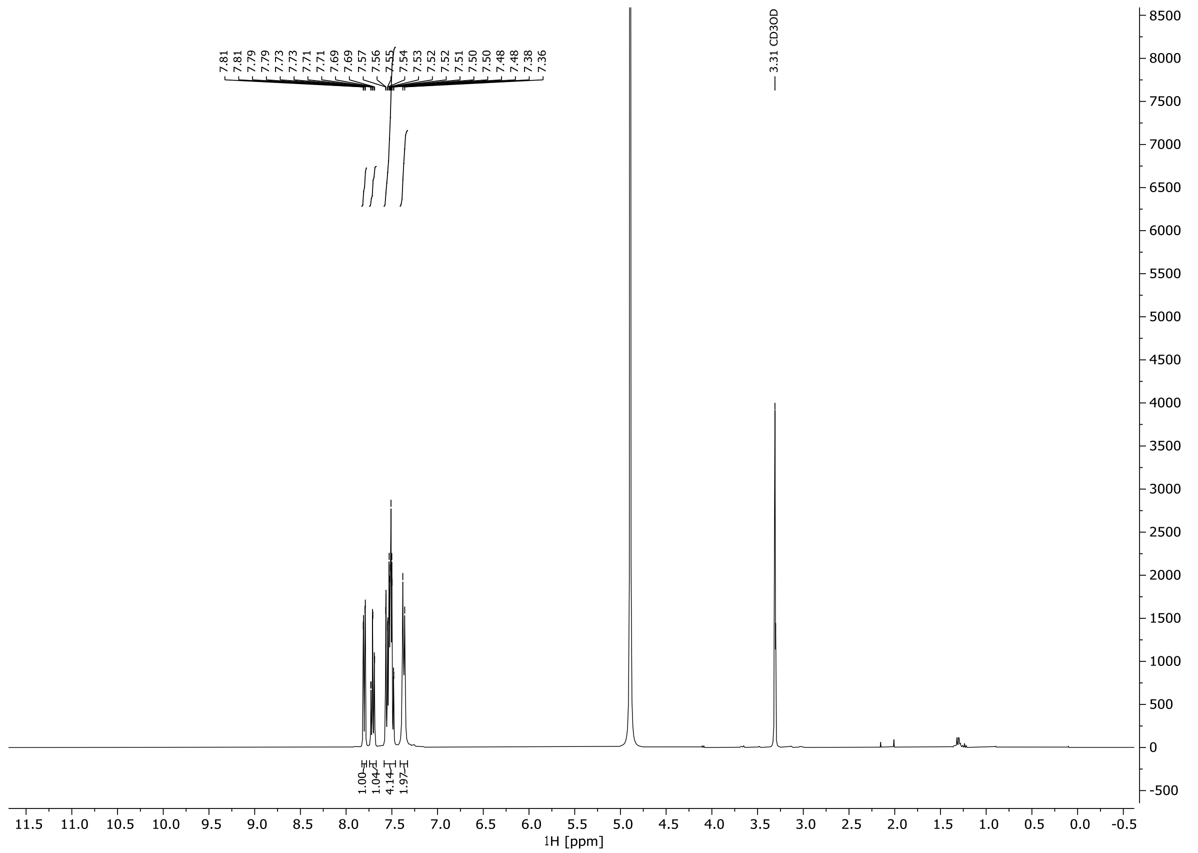

^{31}P NMR of compound **6c**

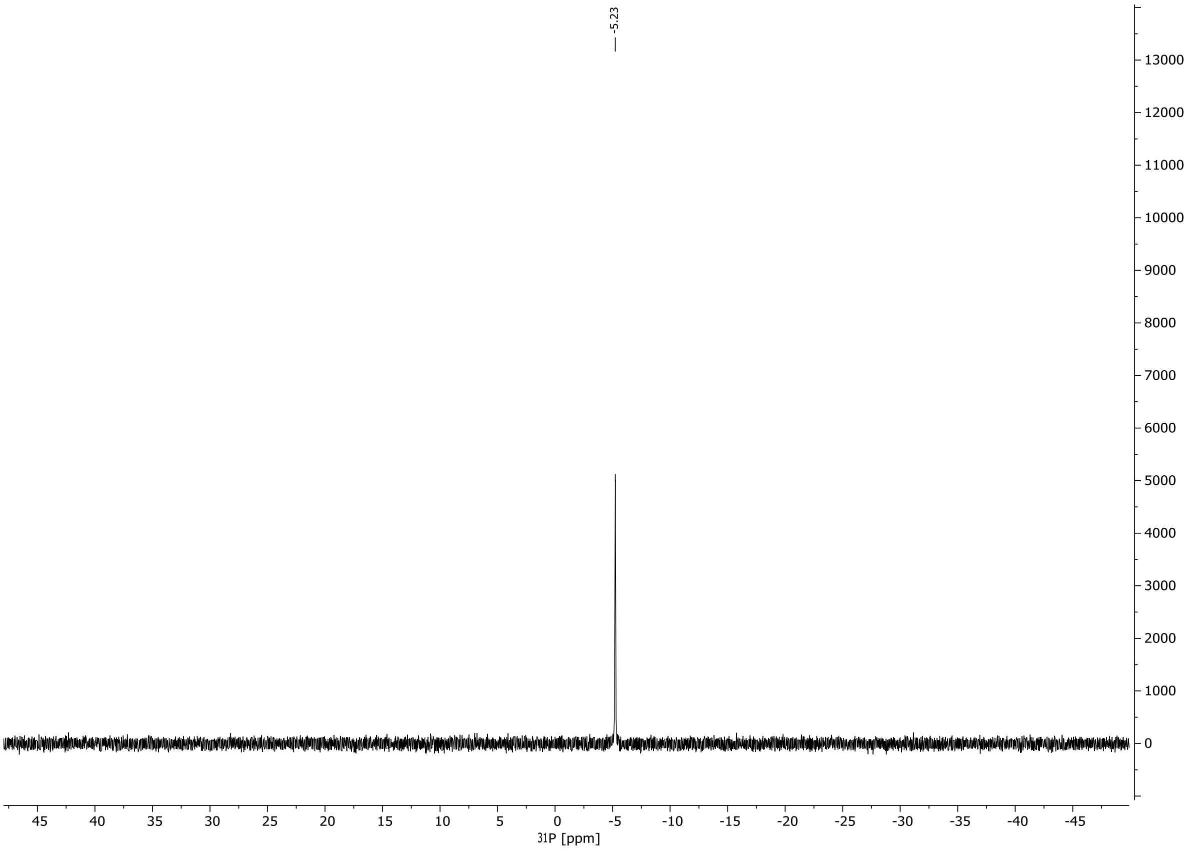

^{13}C NMR of compound **6c**

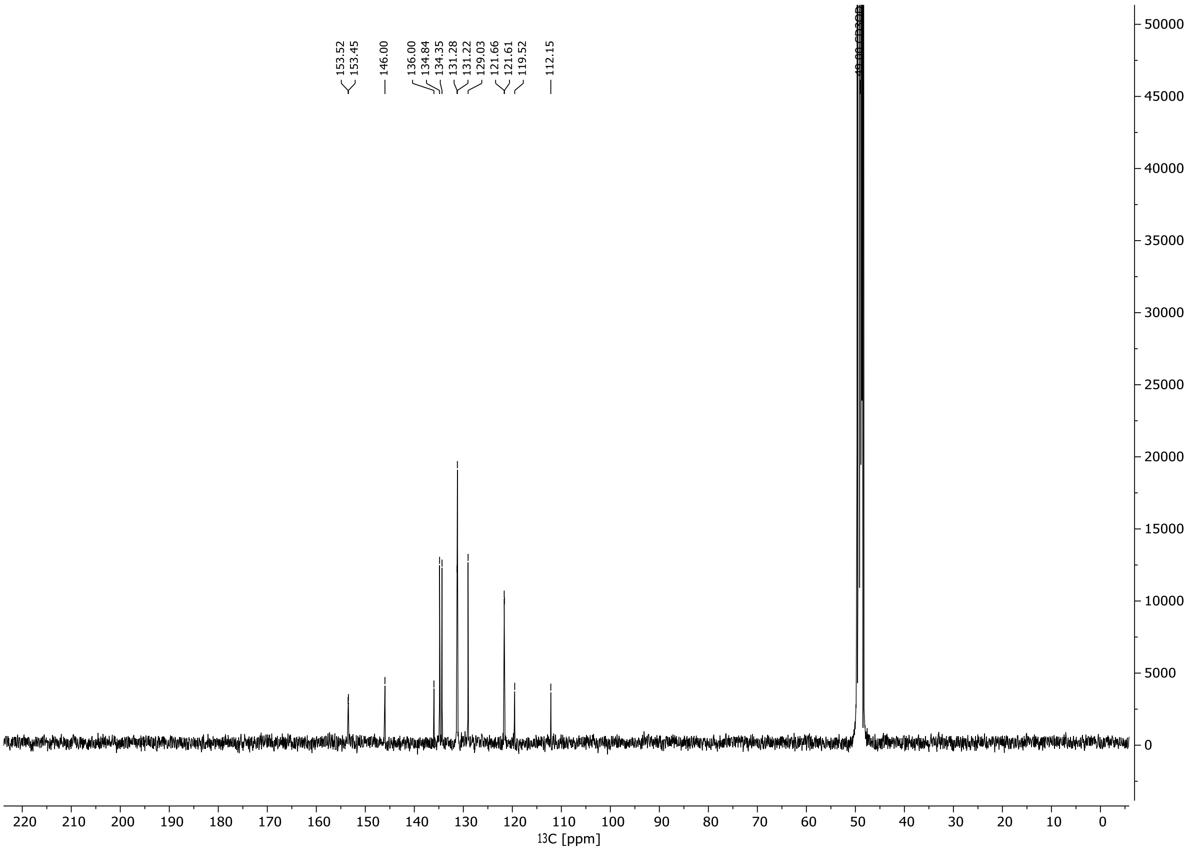

^{1}H NMR of compound **6d**

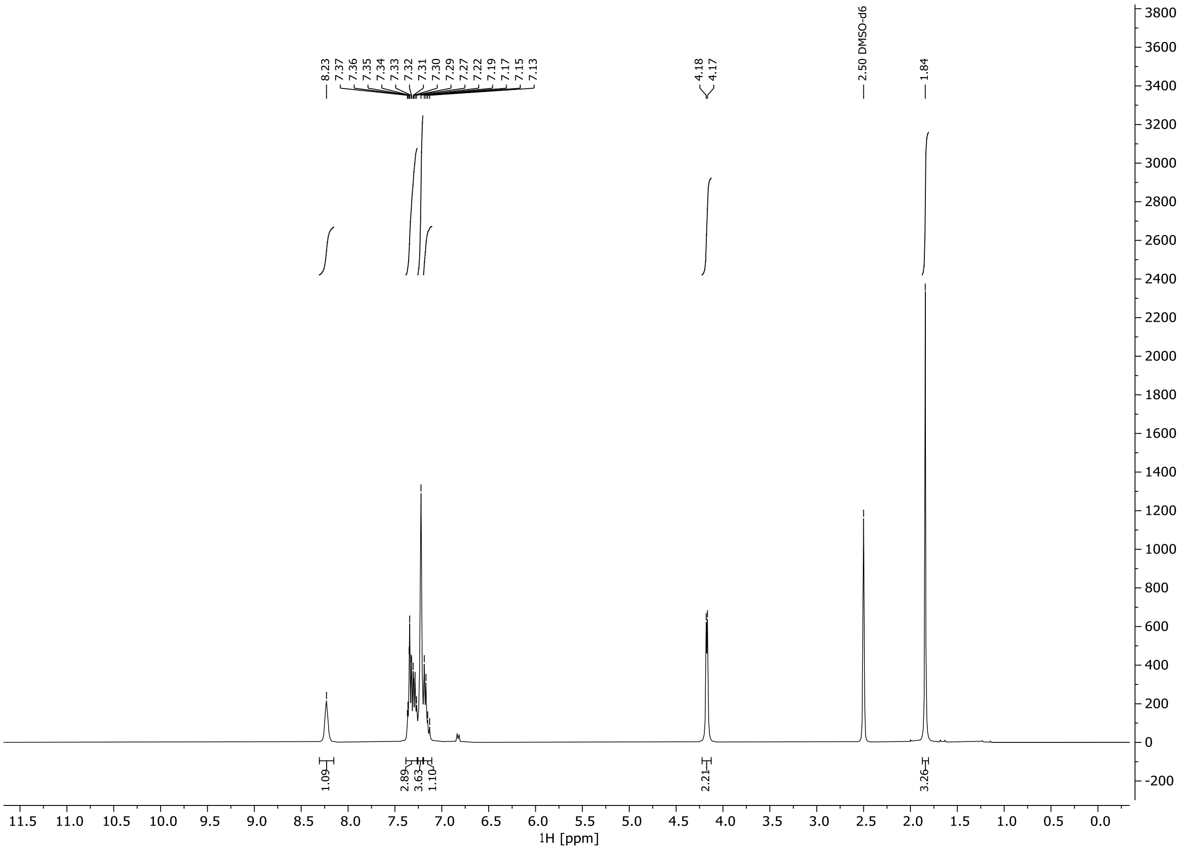

^{31}P NMR of compound **6d**

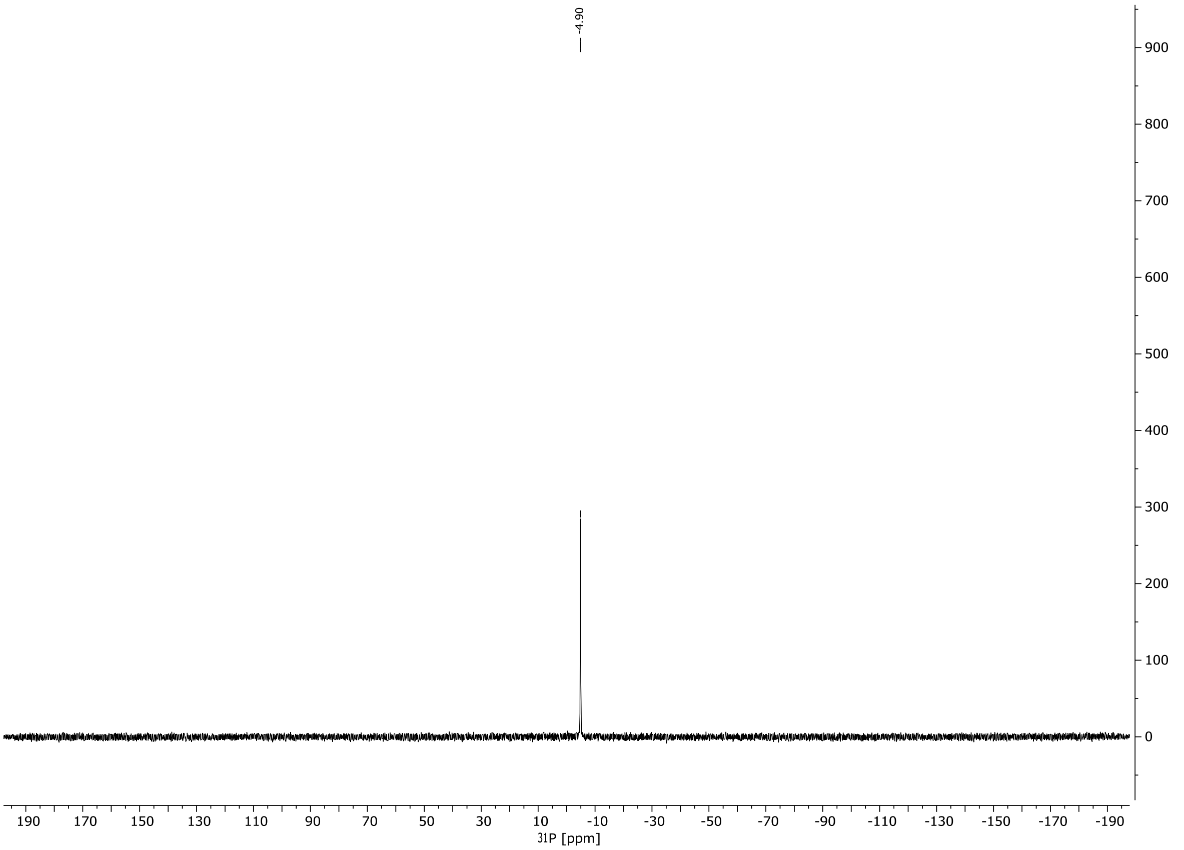

^{13}C NMR of compound **6d**

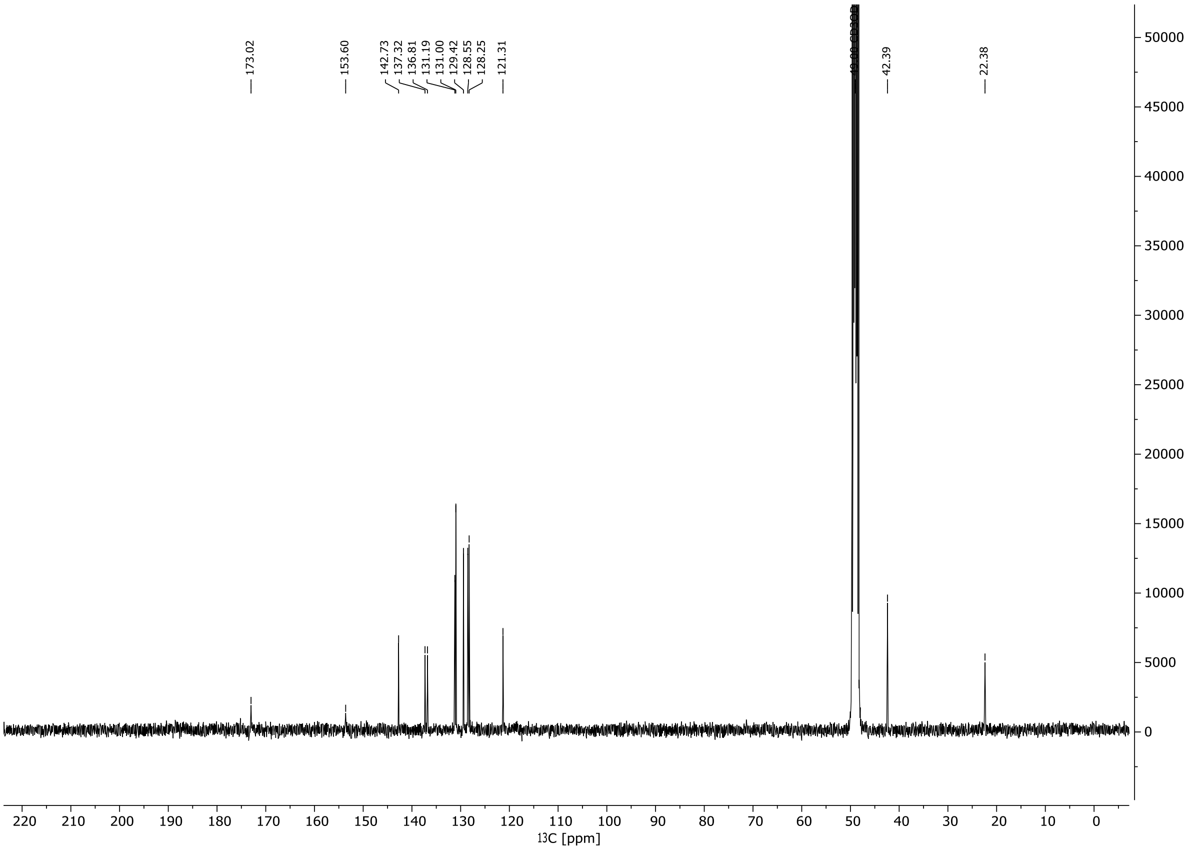

^{1}H NMR of compound **6e**

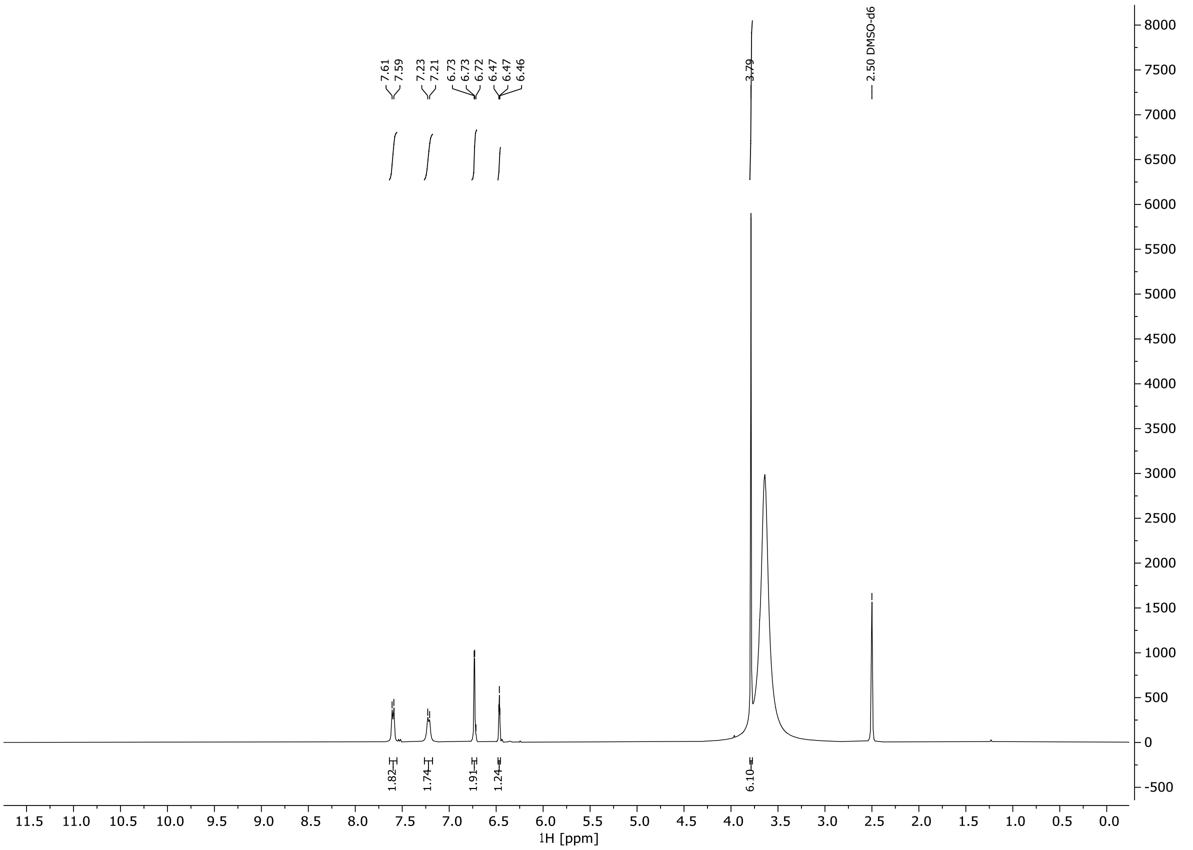

^{31}P NMR of compound **6e**

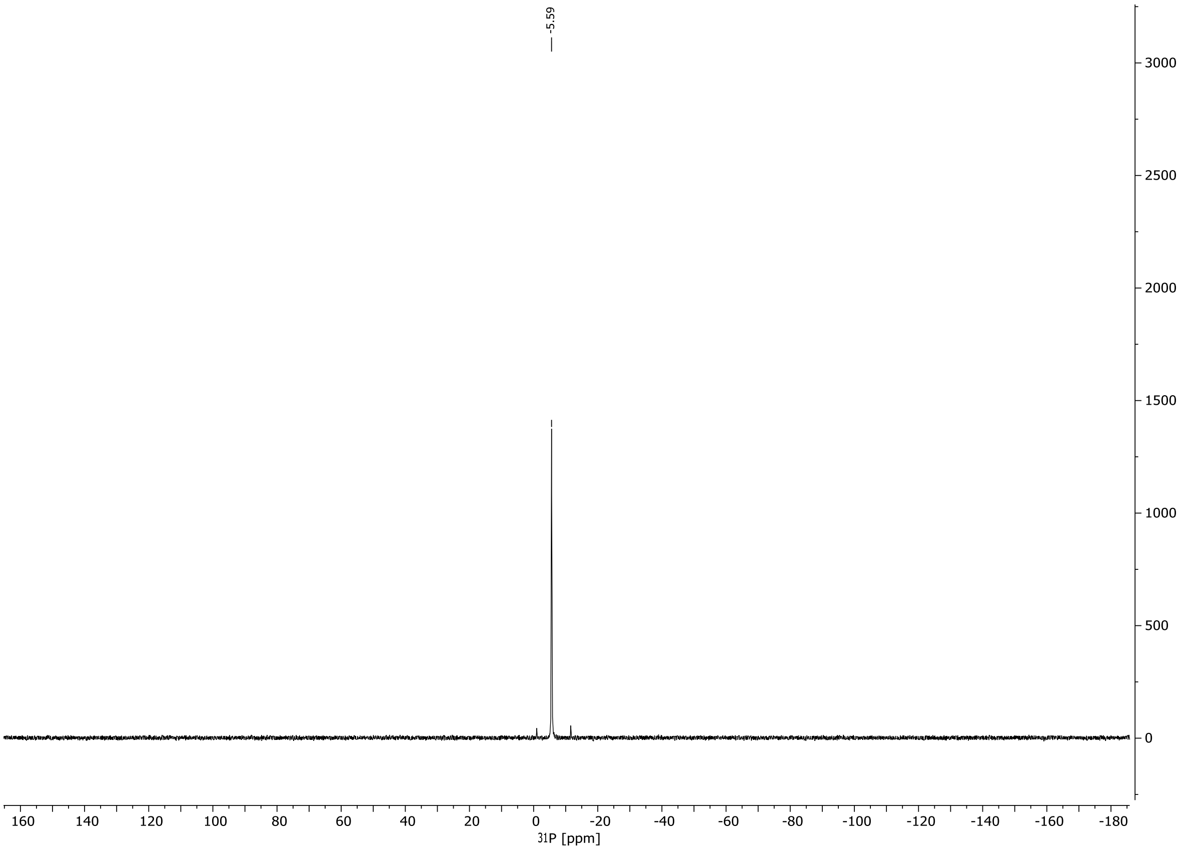

^{13}C NMR of compound **6e**

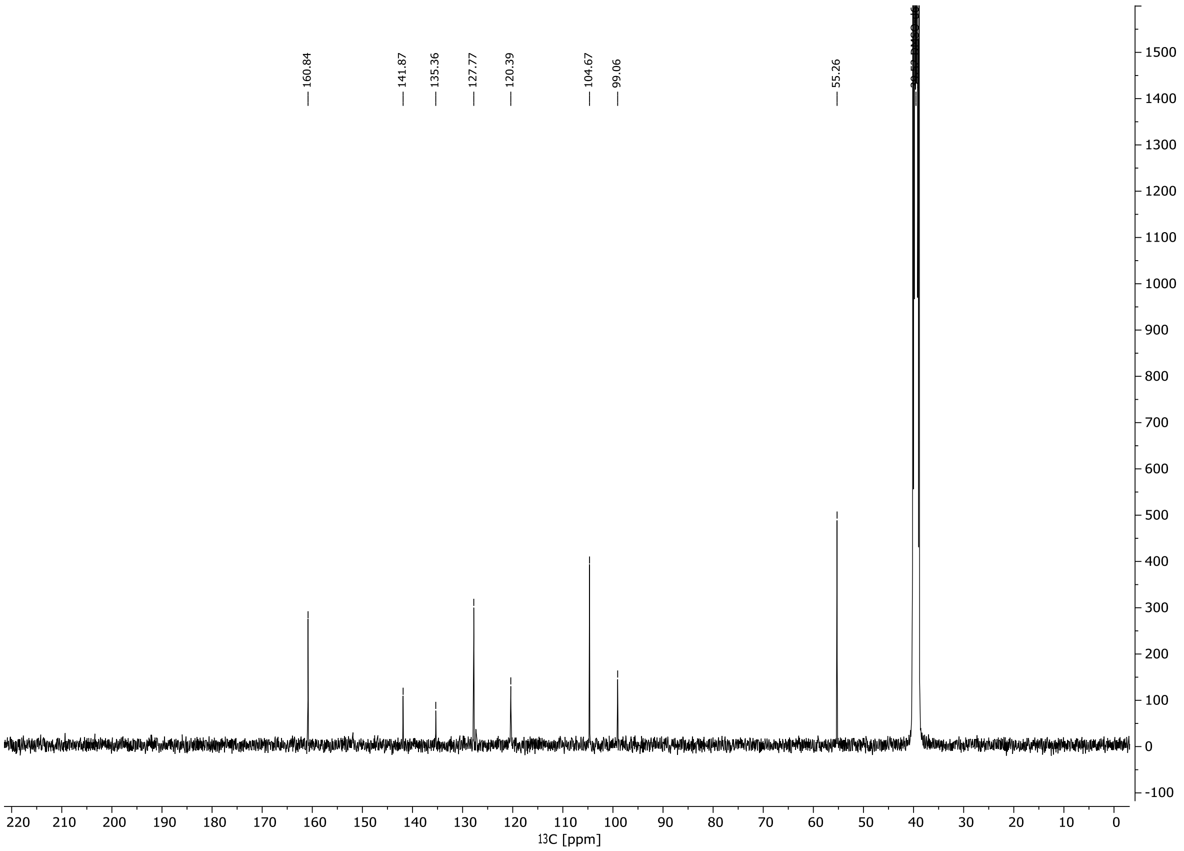

^{1}H NMR of compound **6f**

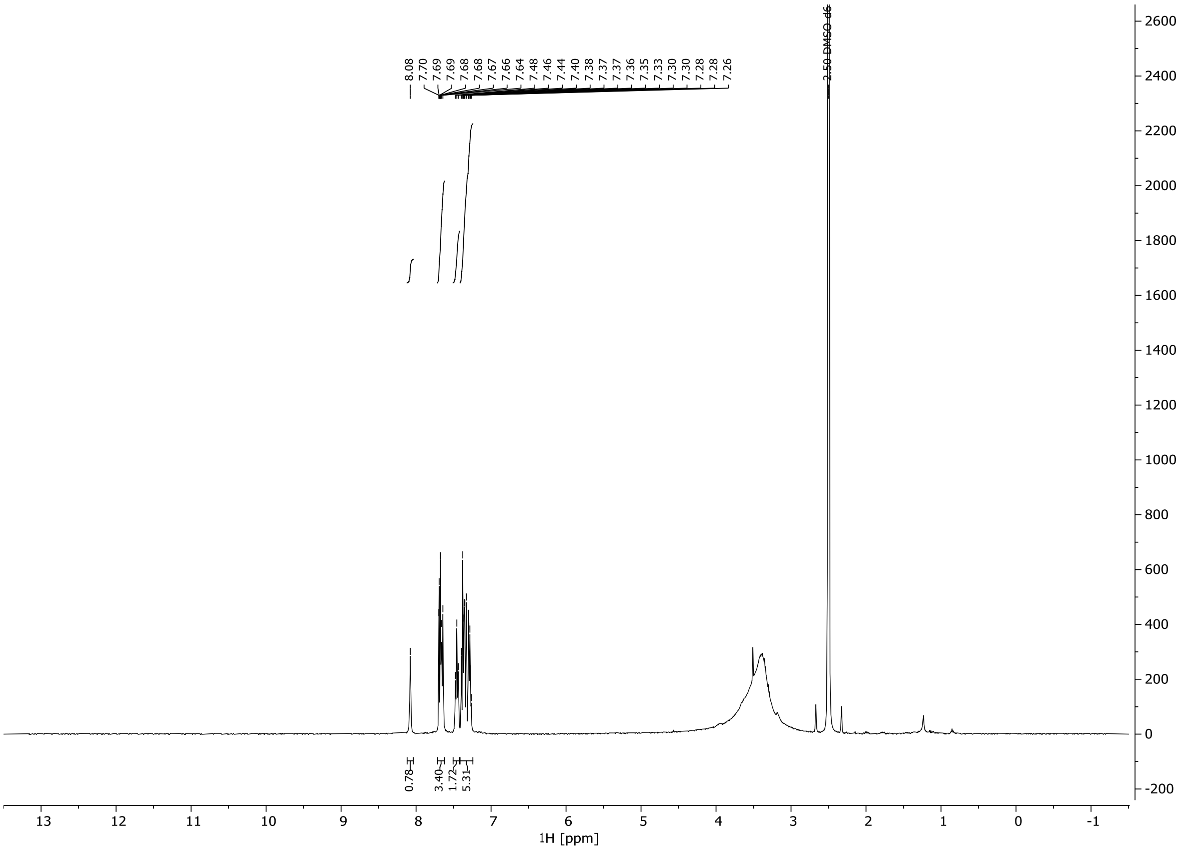

^{31}P NMR of compound **6f**

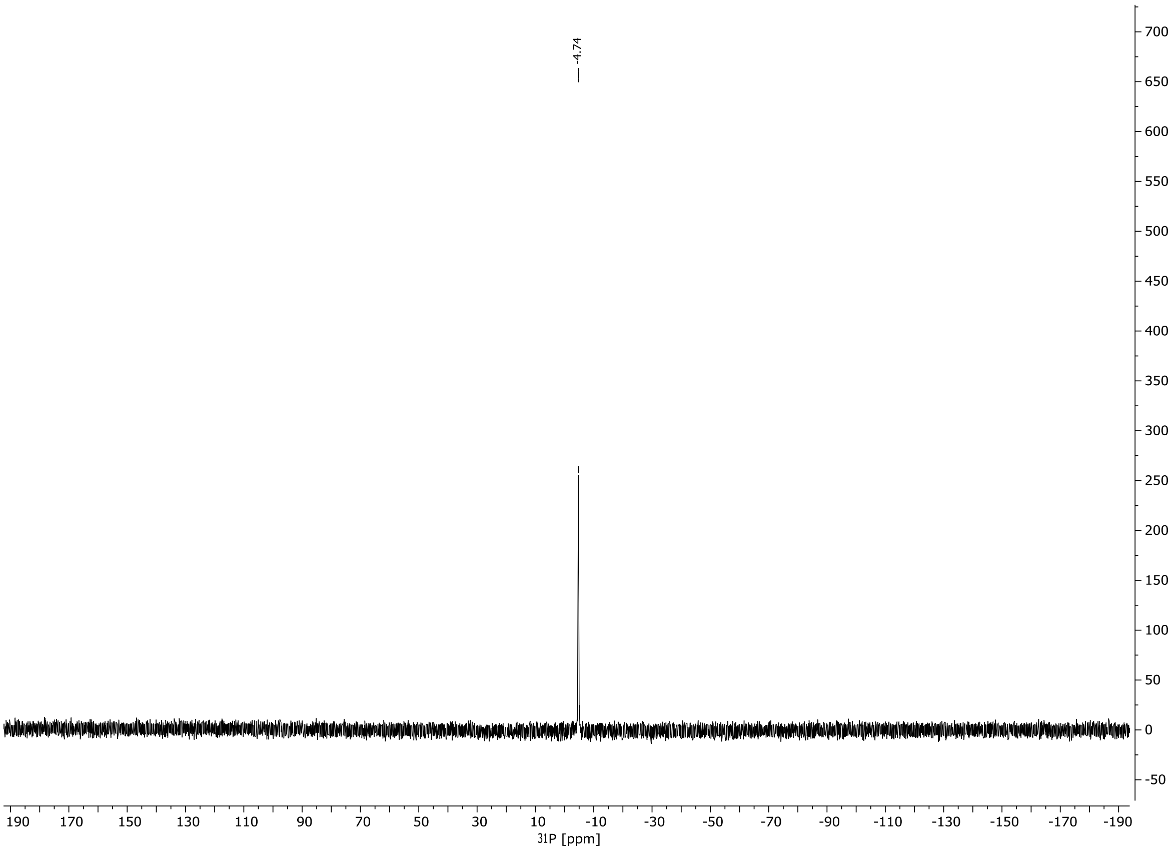

^{13}C NMR of compound **6f**

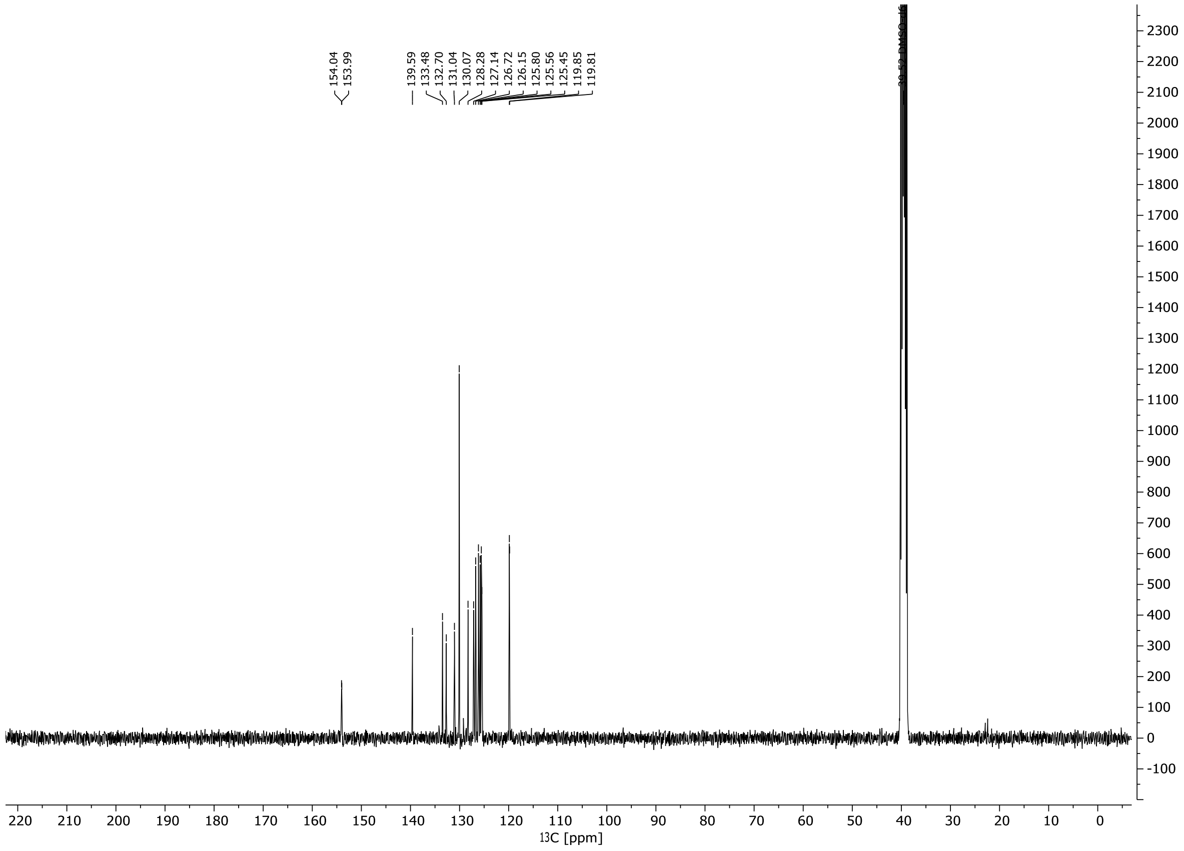

^{1}H NMR of compound **6g**

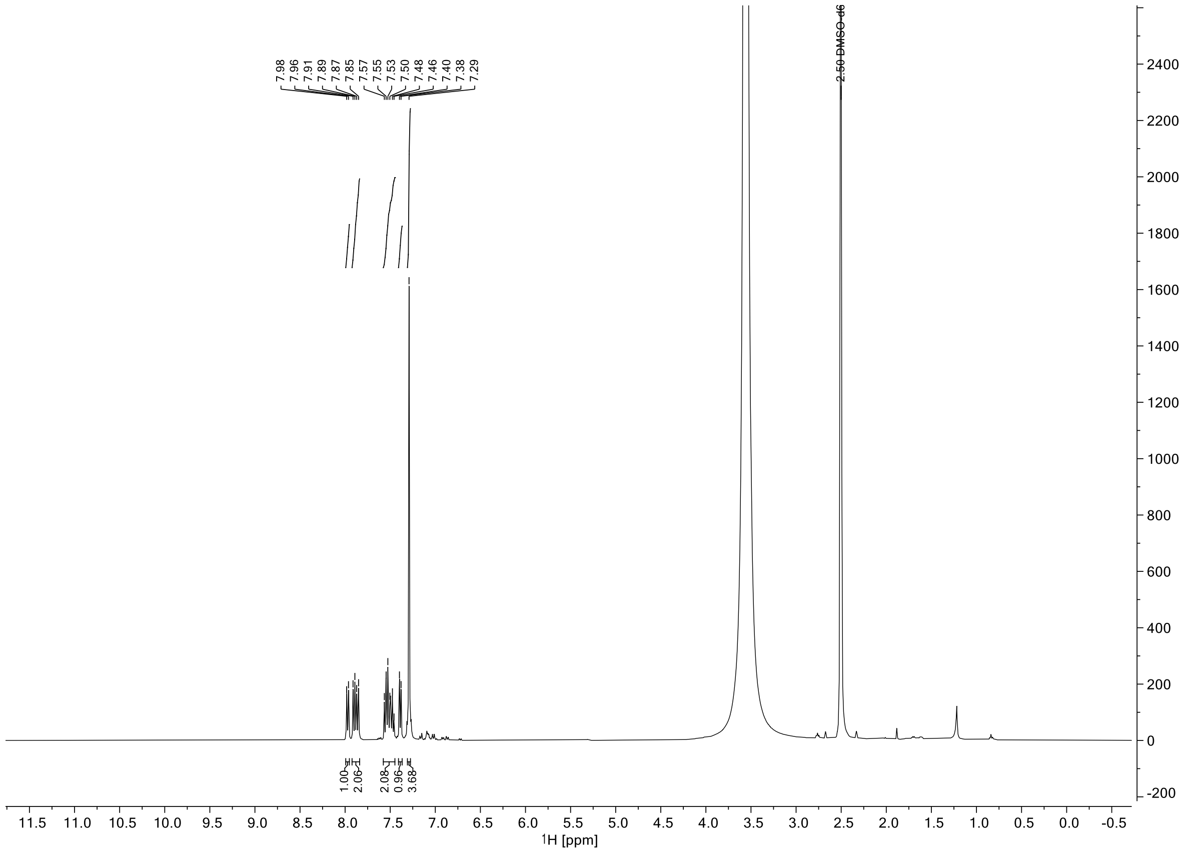

^{31}P NMR of compound **6g**

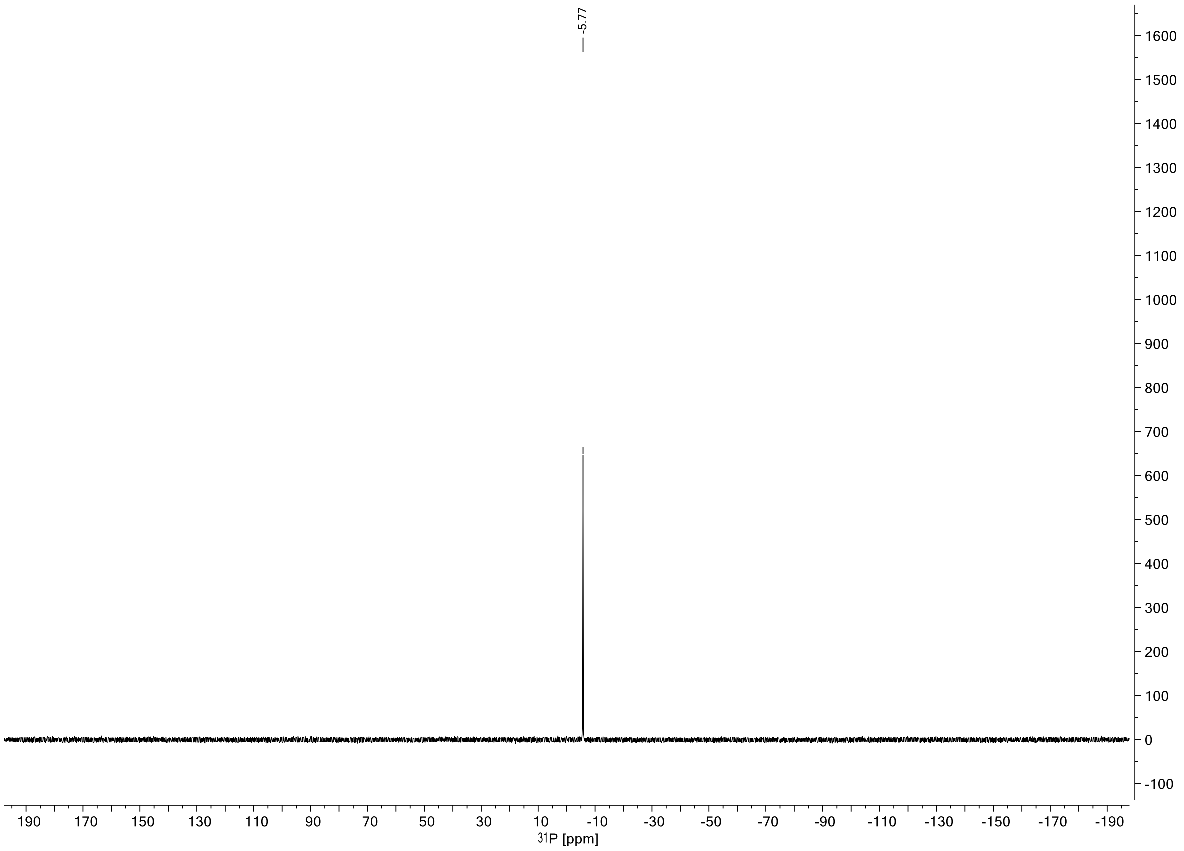

^{13}C NMR of compound **6g**

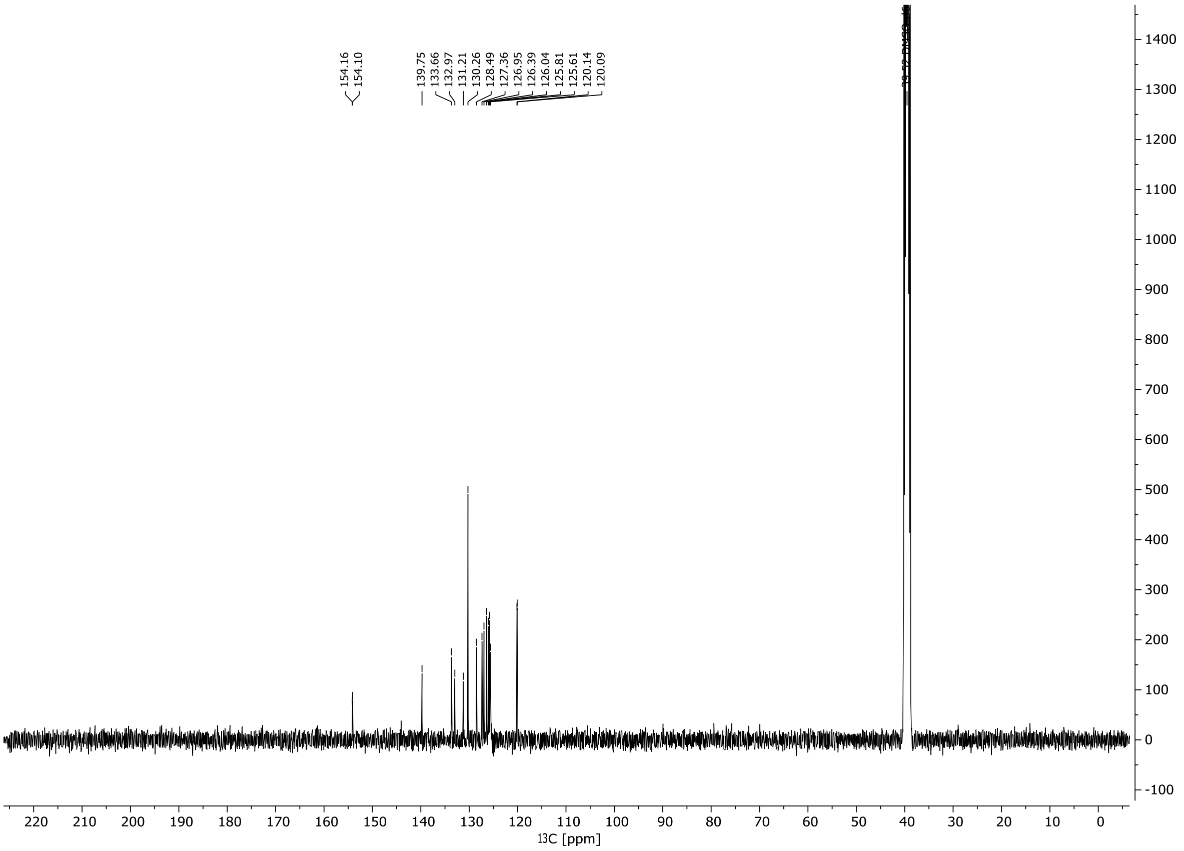

^1H NMR of compound **6h**

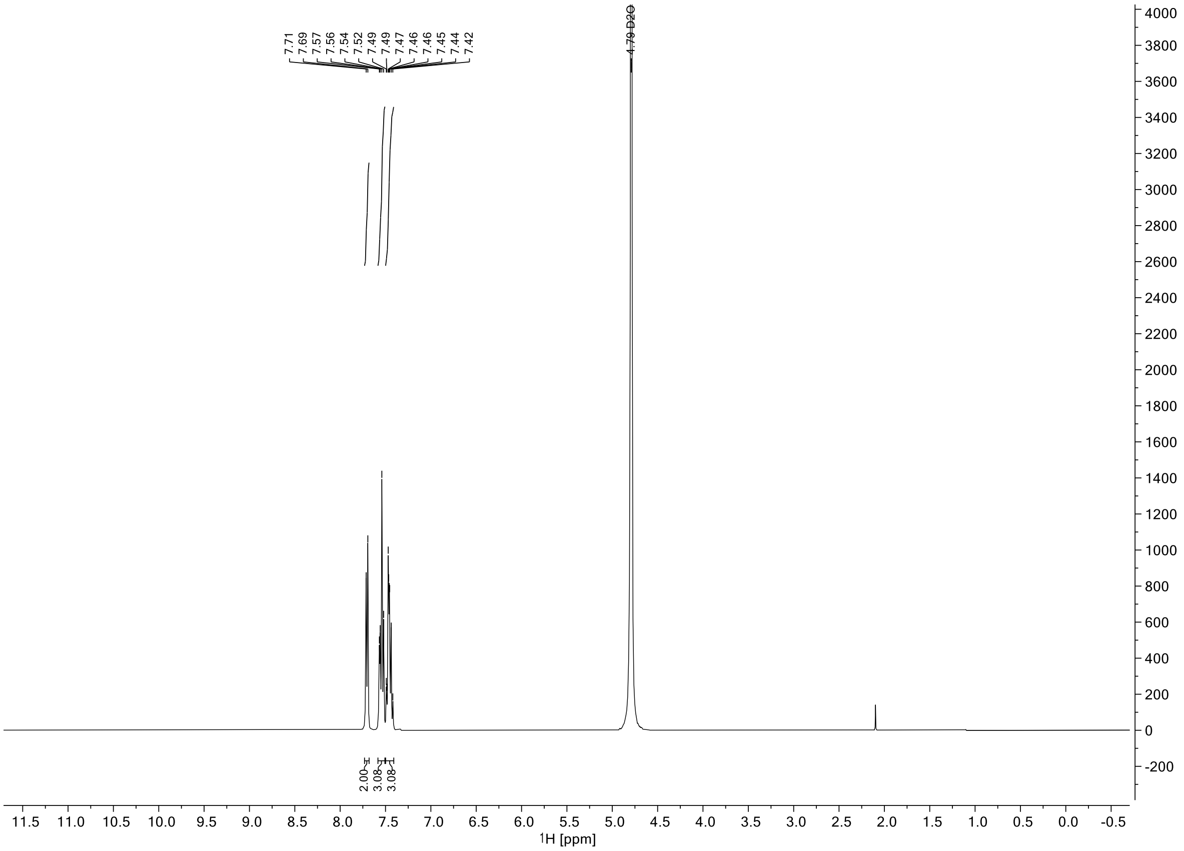

^{19}F NMR of compound **6h**

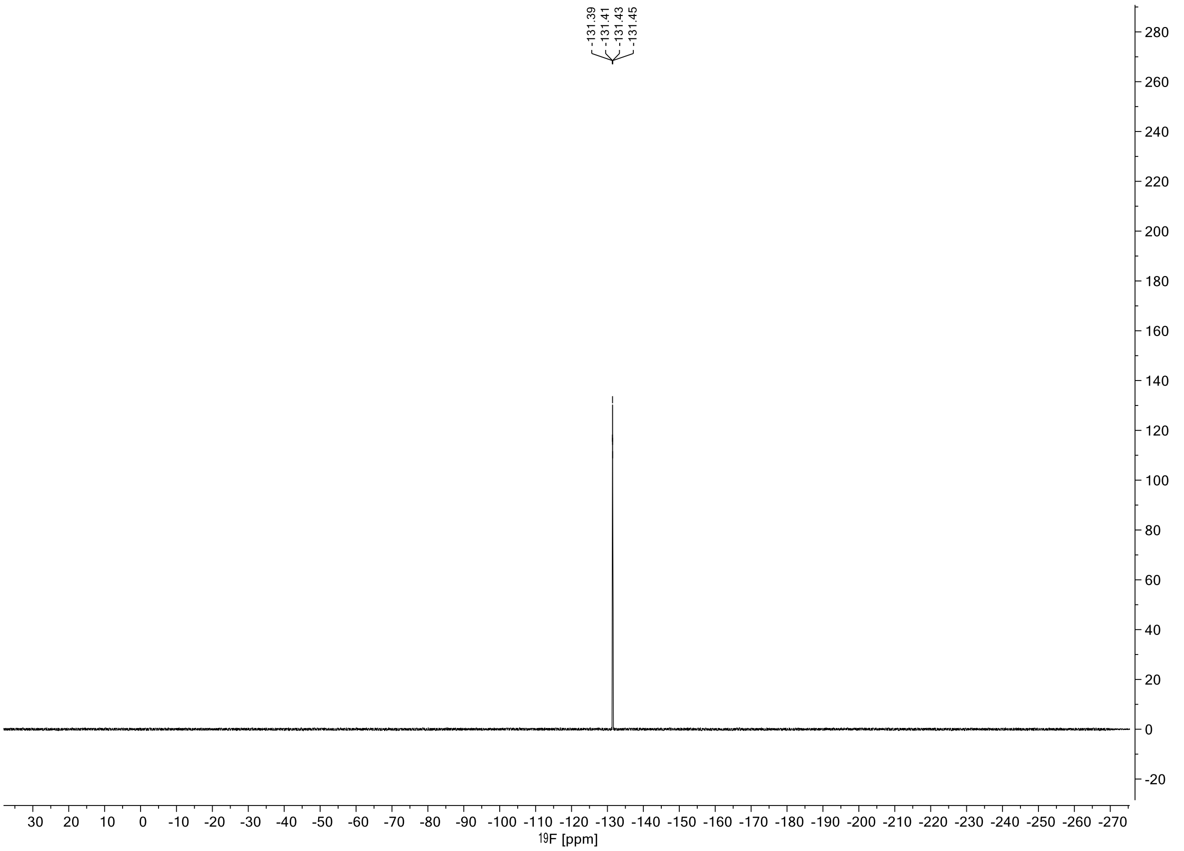

^{31}P NMR of compound **6h**

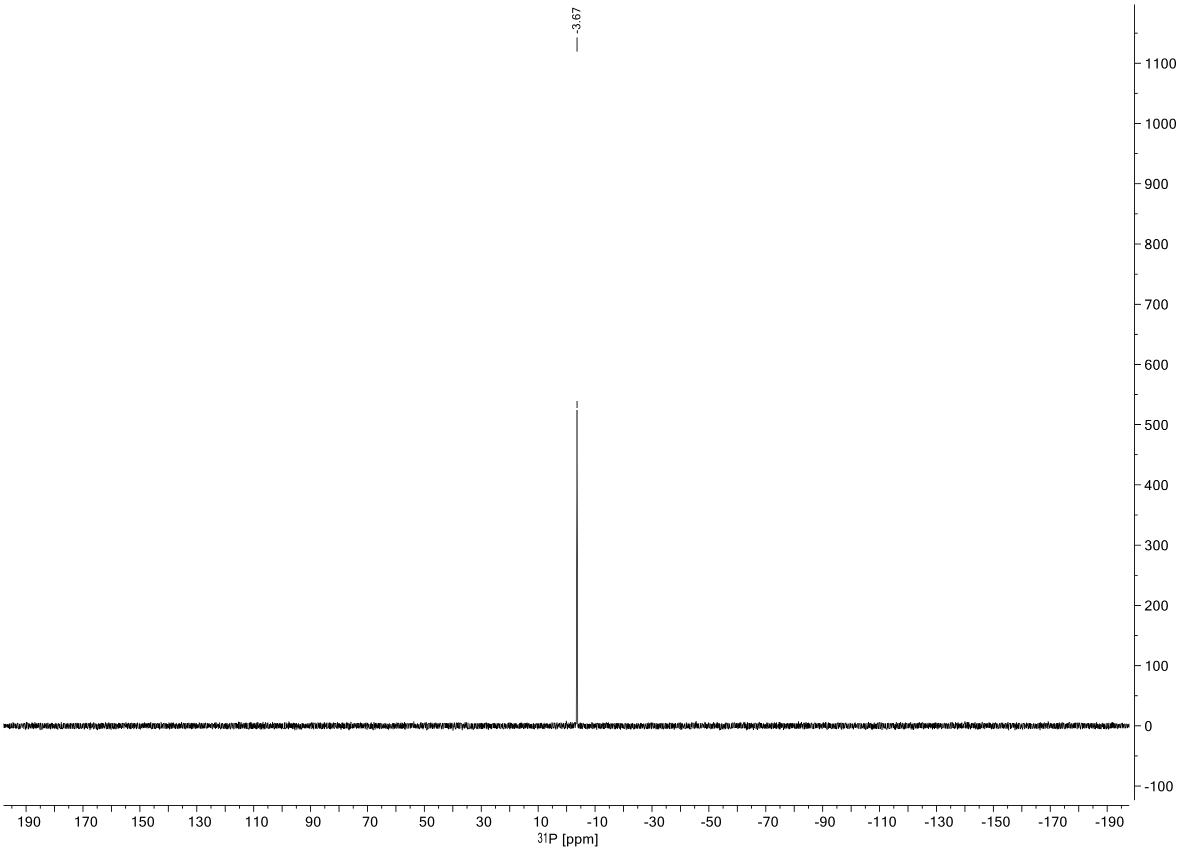

^{13}C NMR of compound **6h**

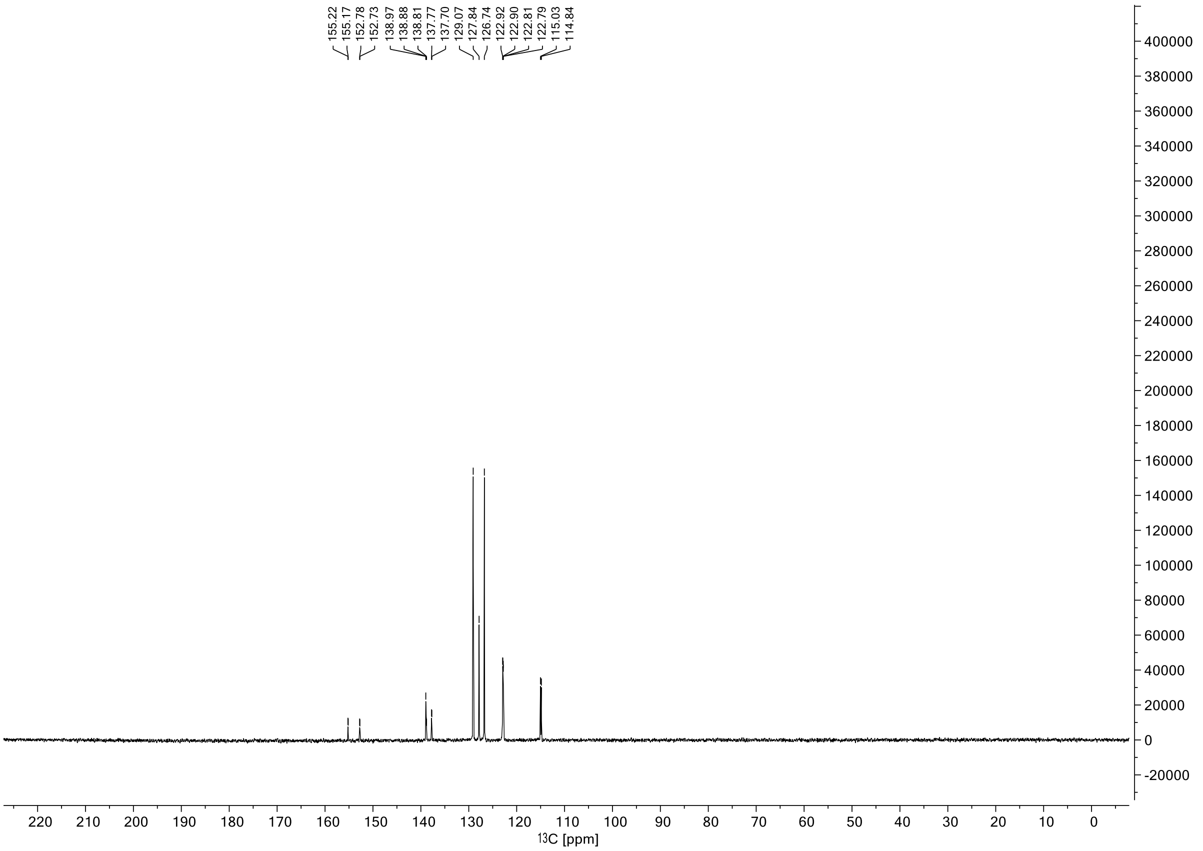

^{1}H NMR of compound **6i**

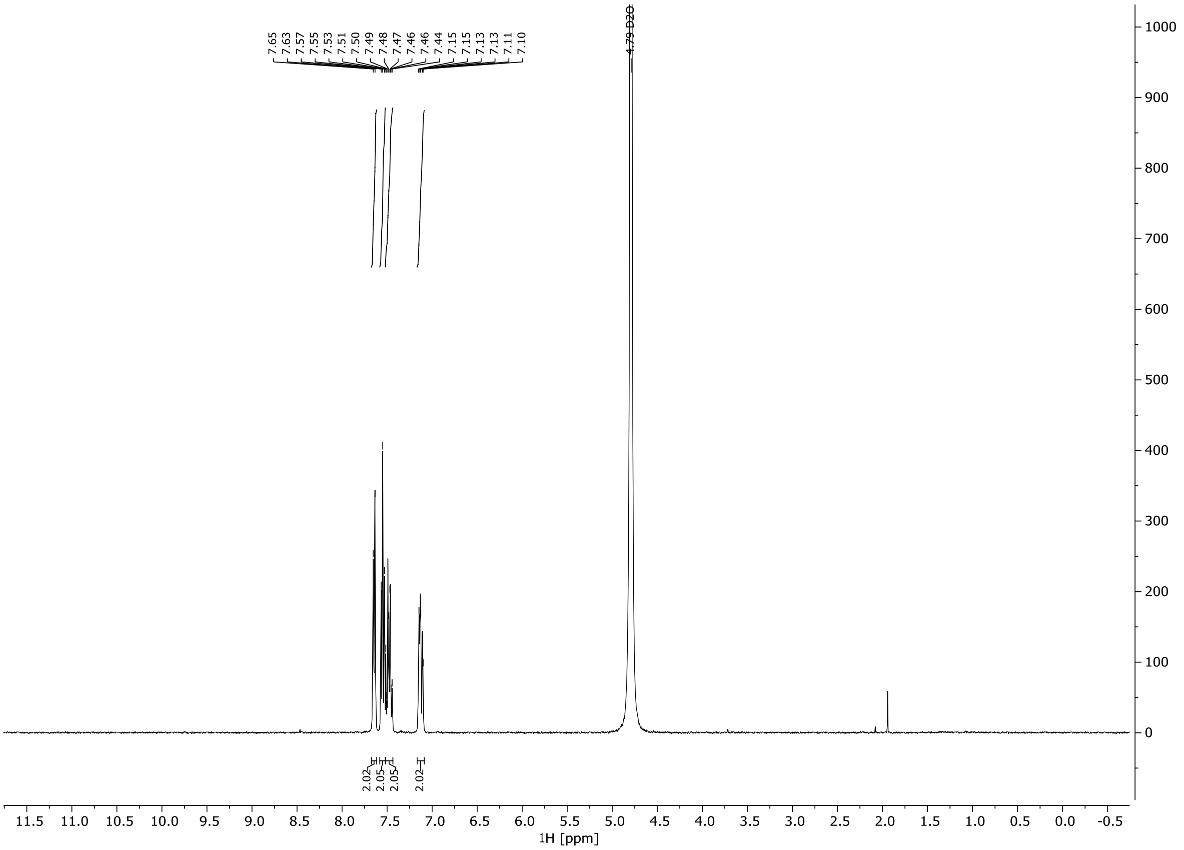

^{19}F NMR of compound **6i**

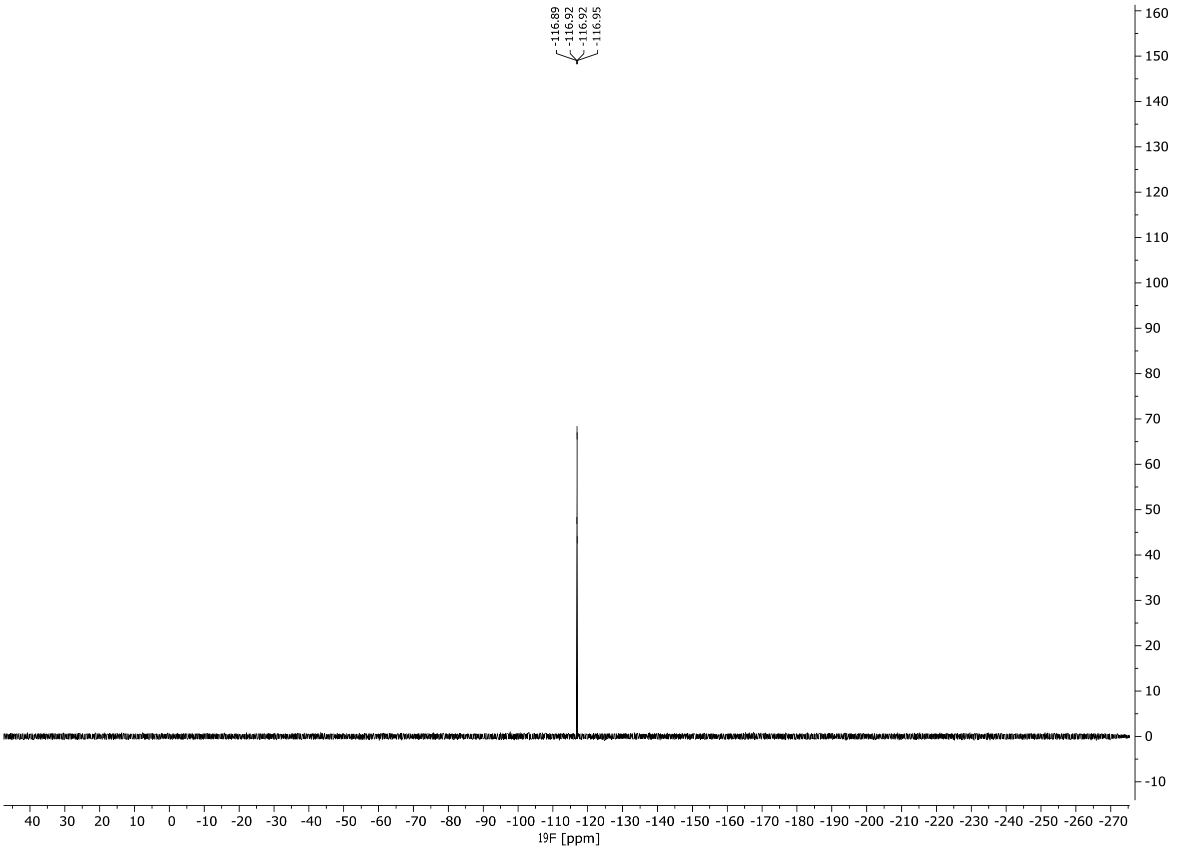

^{31}P NMR of compound **6i**

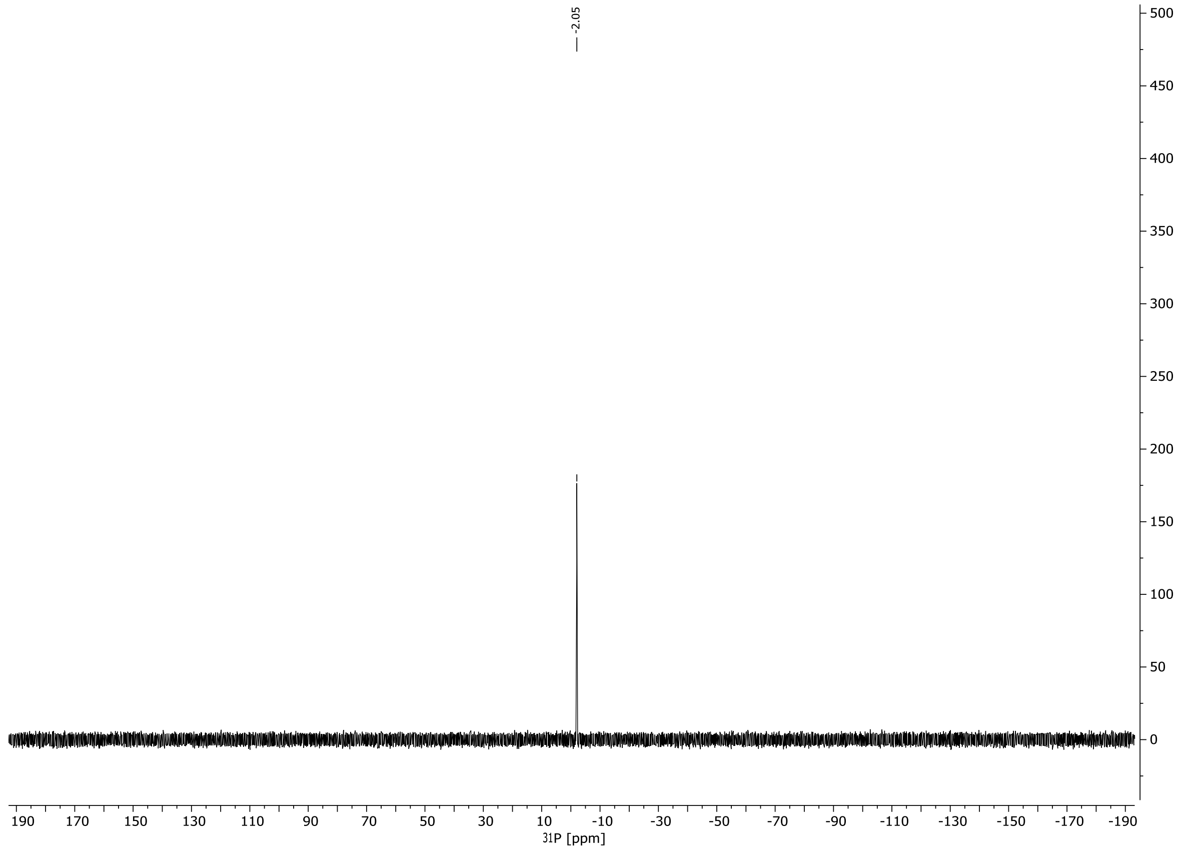

^{13}C NMR of compound **6i**

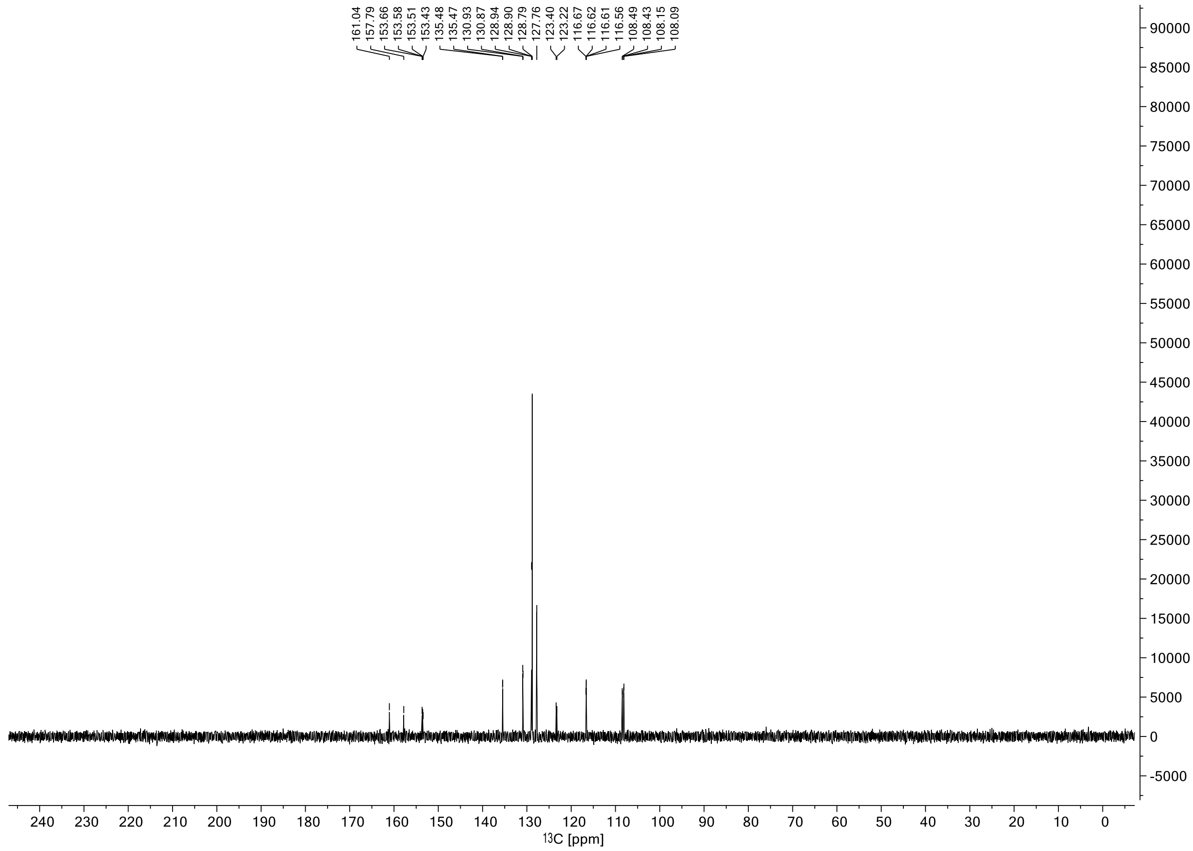

^{1}H NMR of compound **6j**

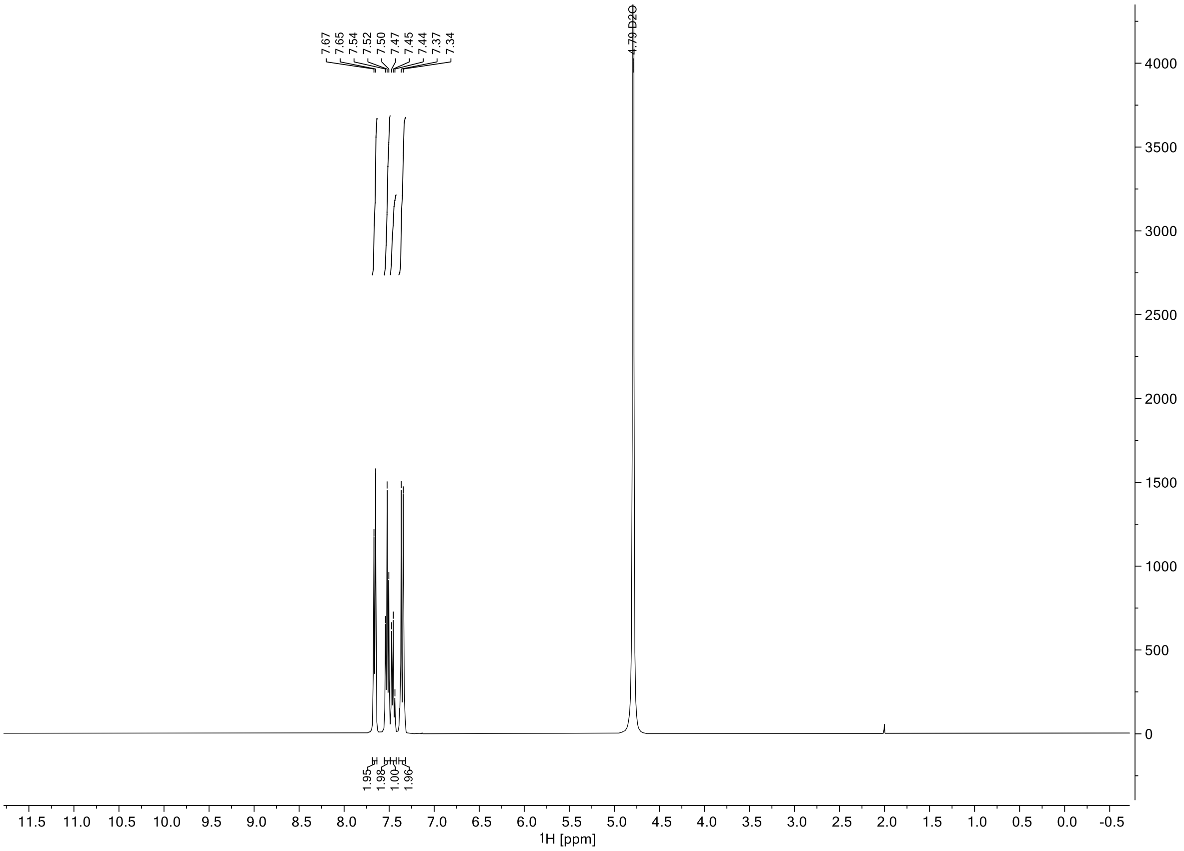

^{19}F NMR of compound **6j**

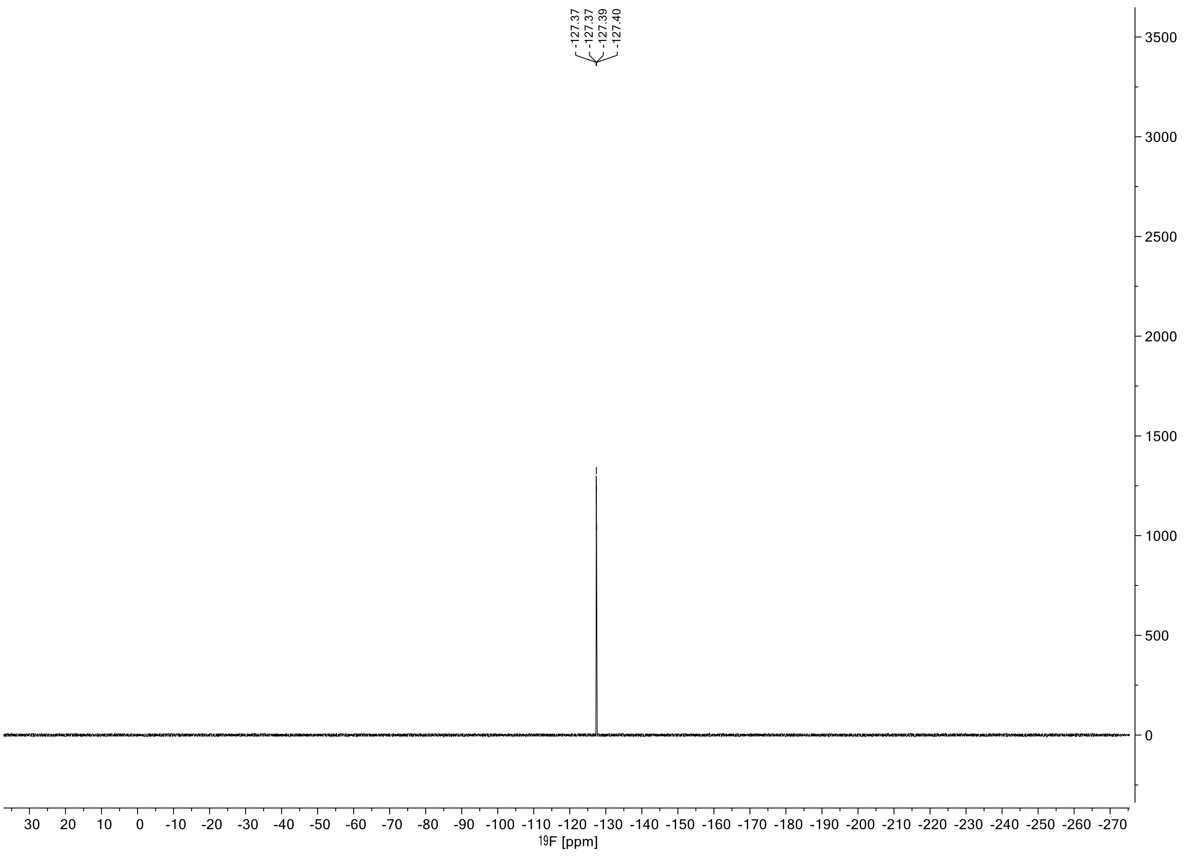

^{31}P NMR of compound **6j**

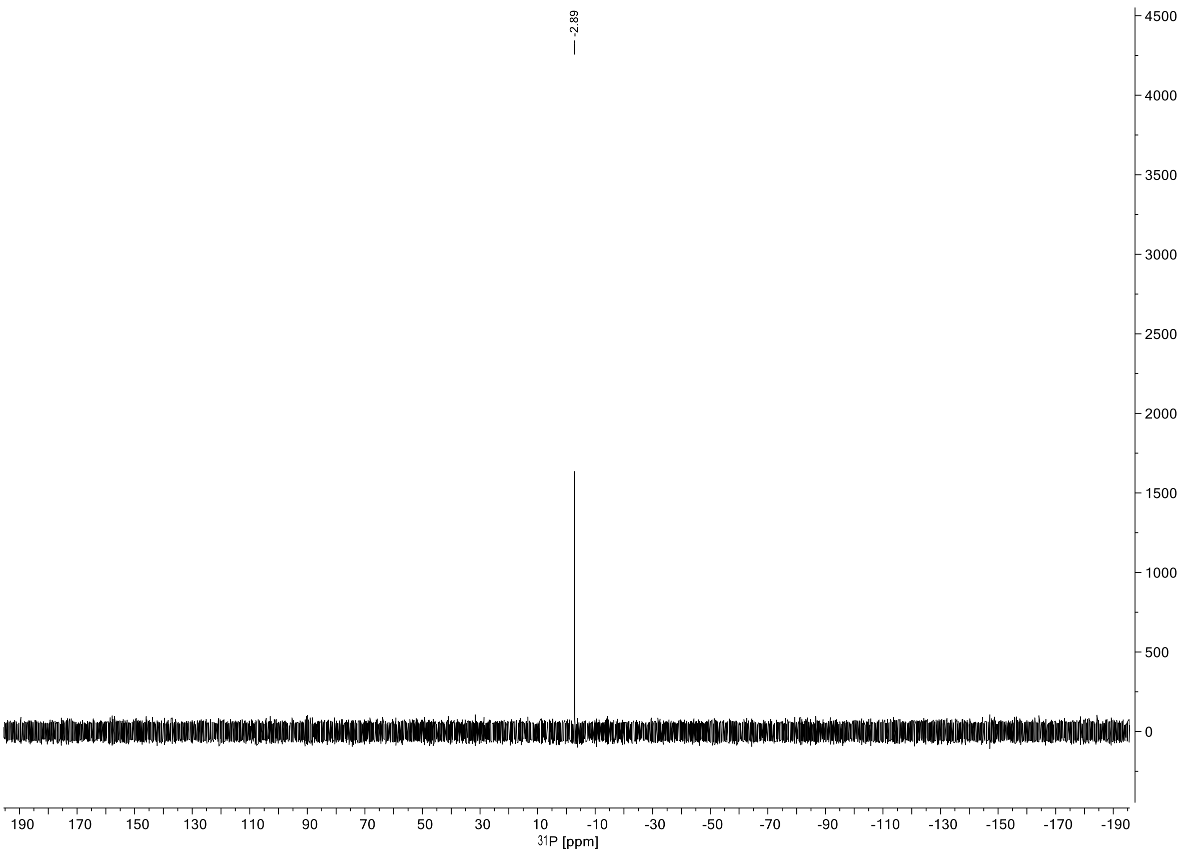

^{13}C NMR of compound **6j**

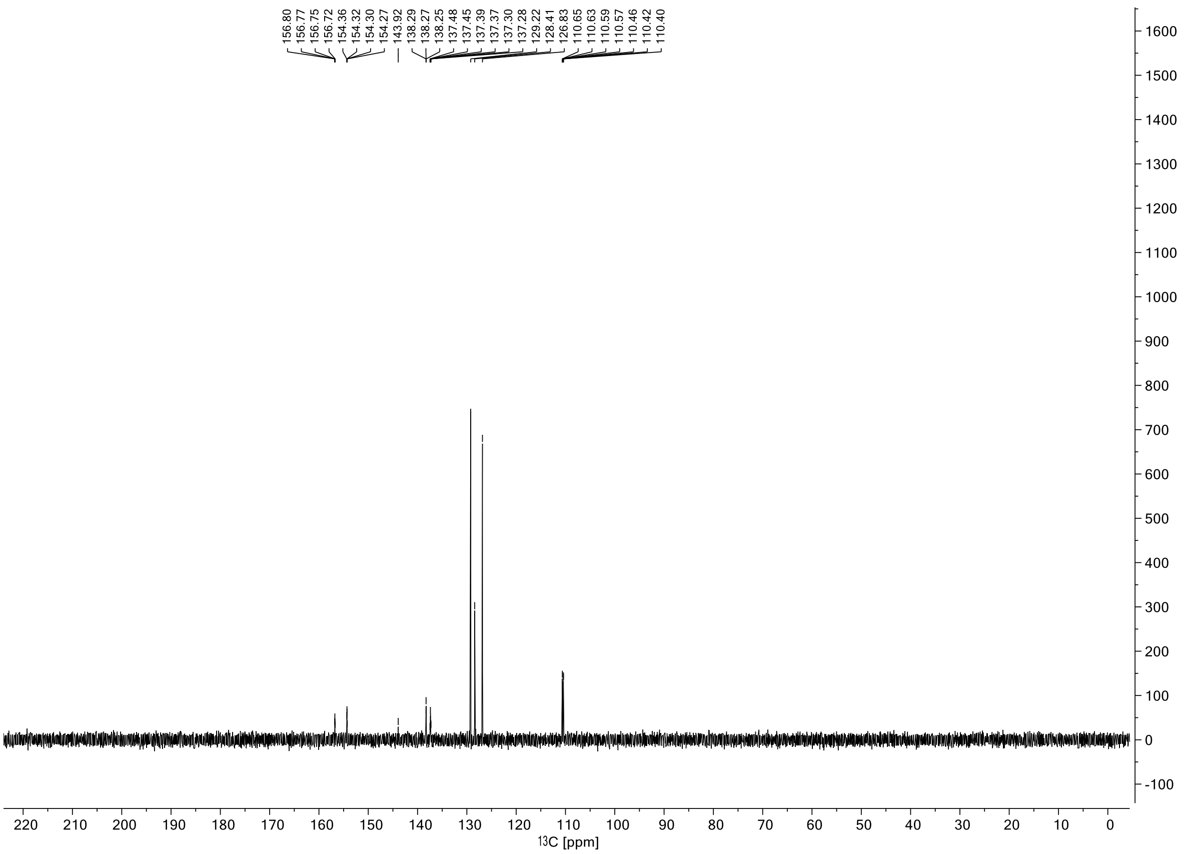

^{1}H NMR of compound **6k**

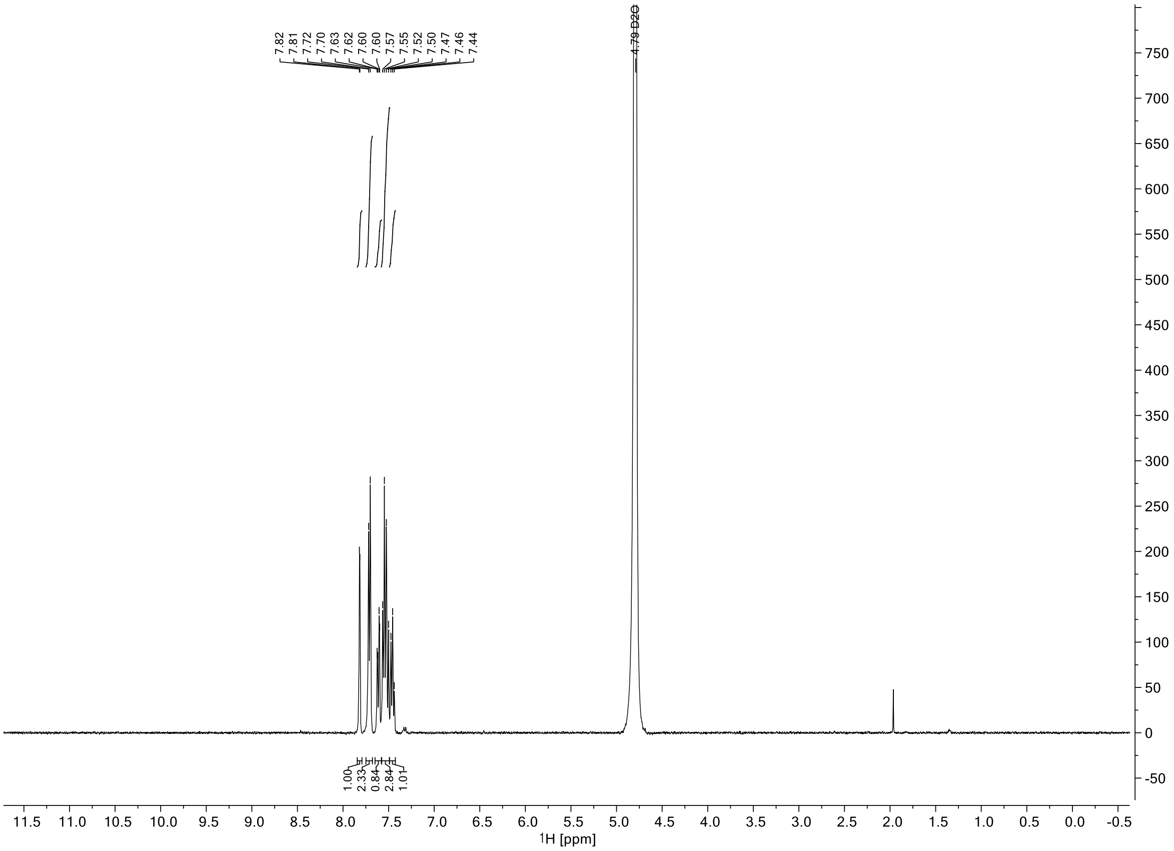

^{31}P NMR of compound **6k**

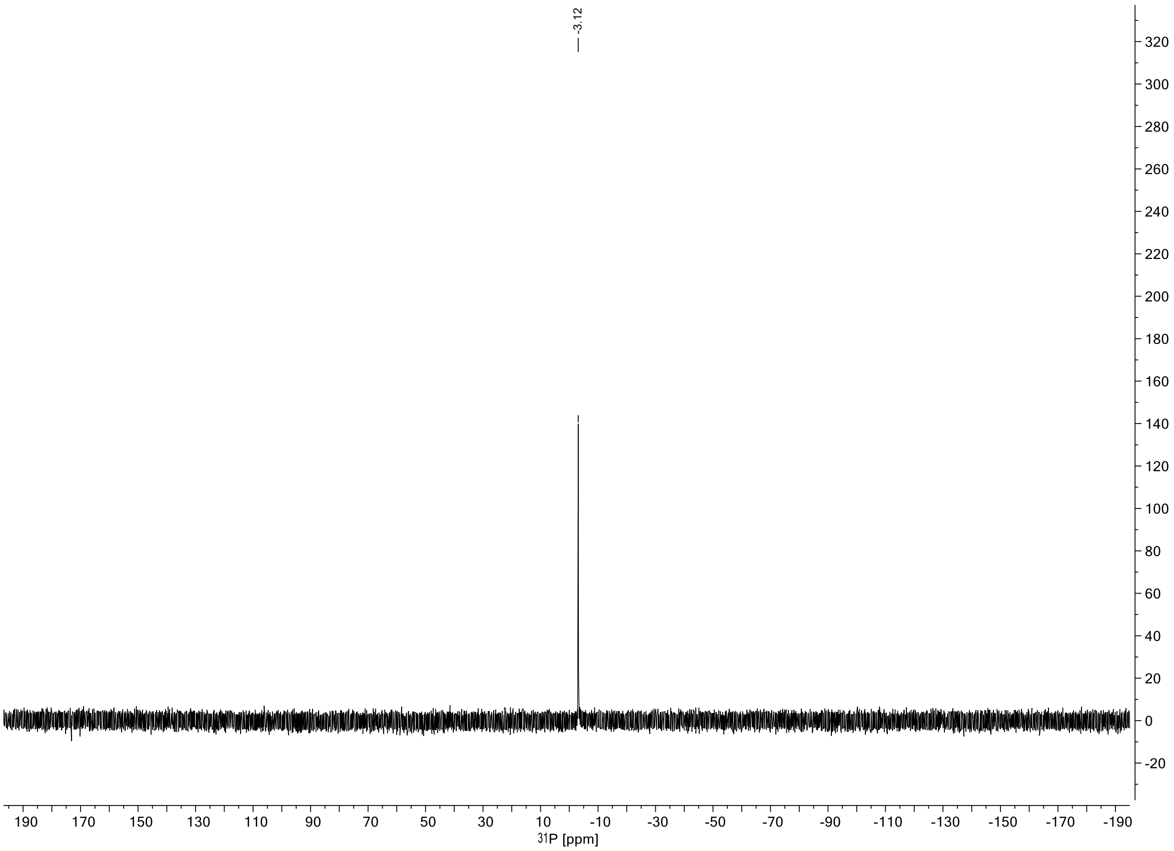

31C NMR of compound **6k**

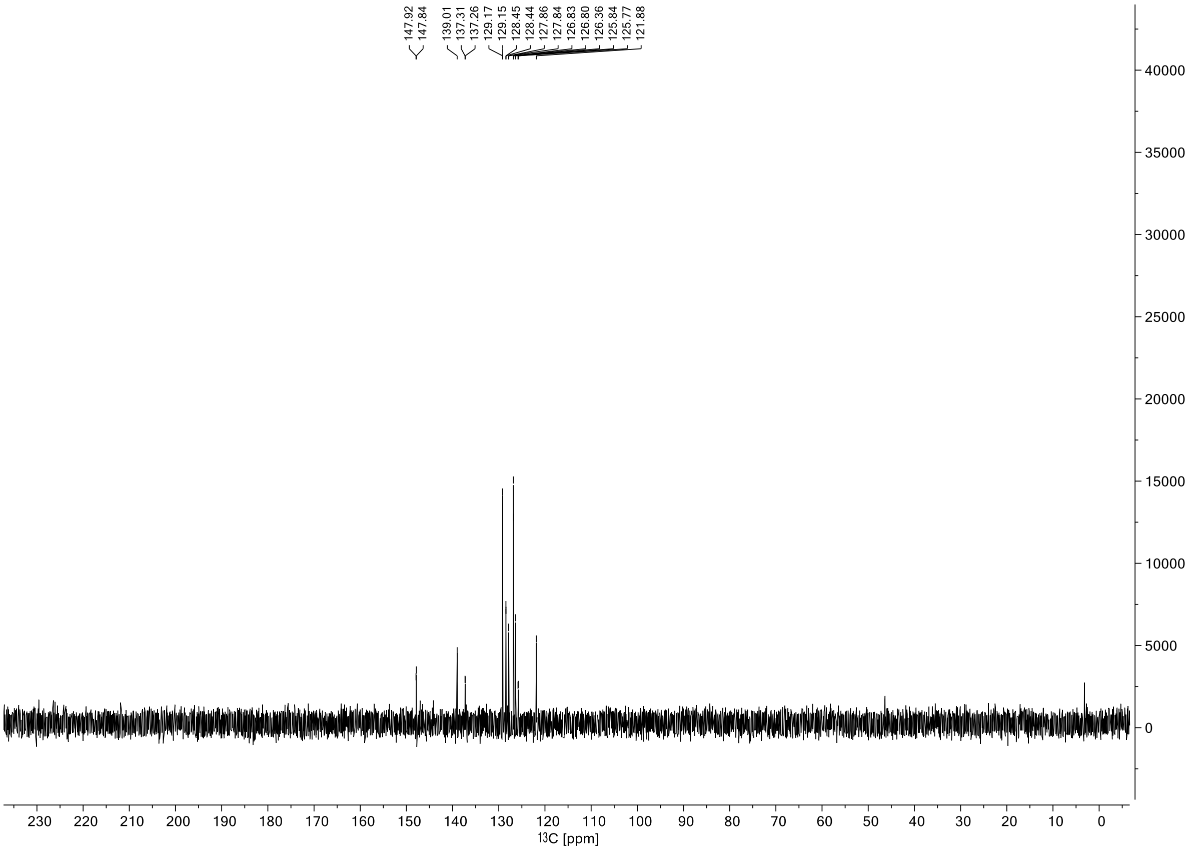

1H NMR of compound **6l**

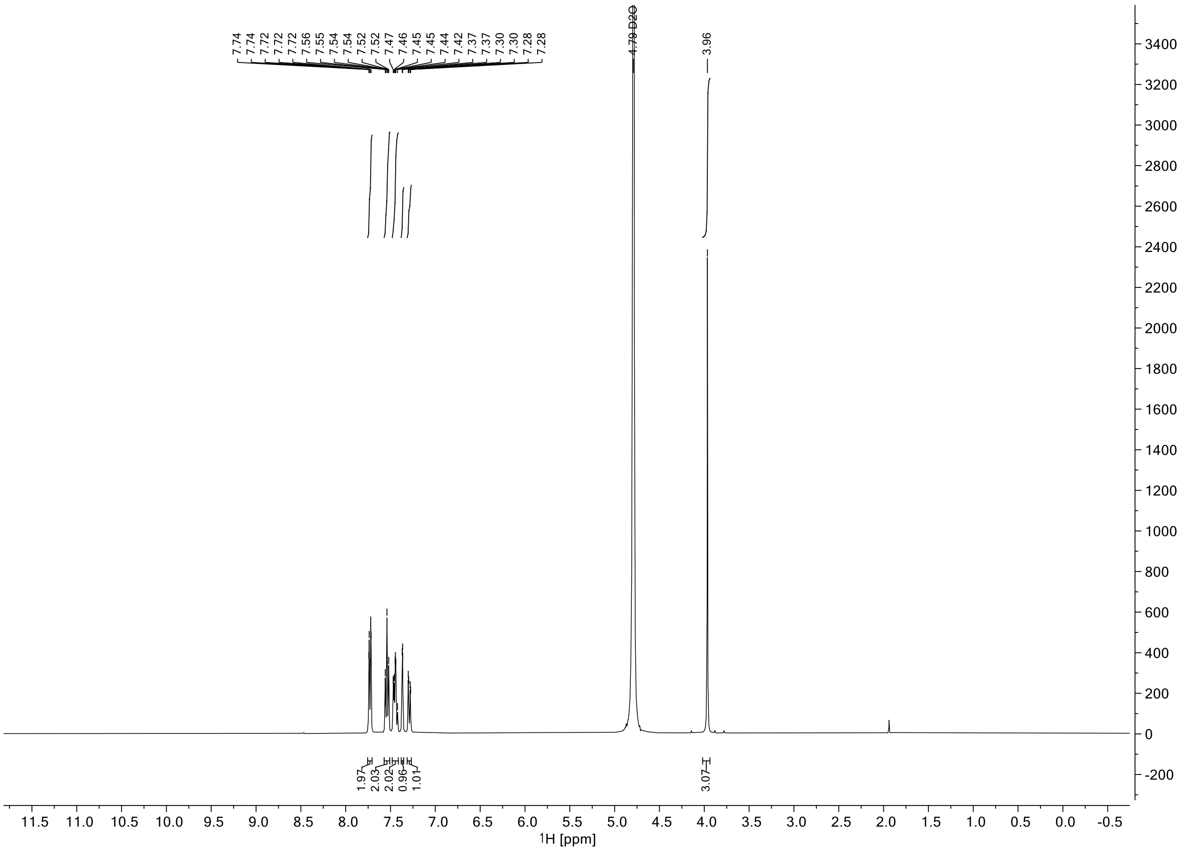

[13]P NMR of compound **6l**

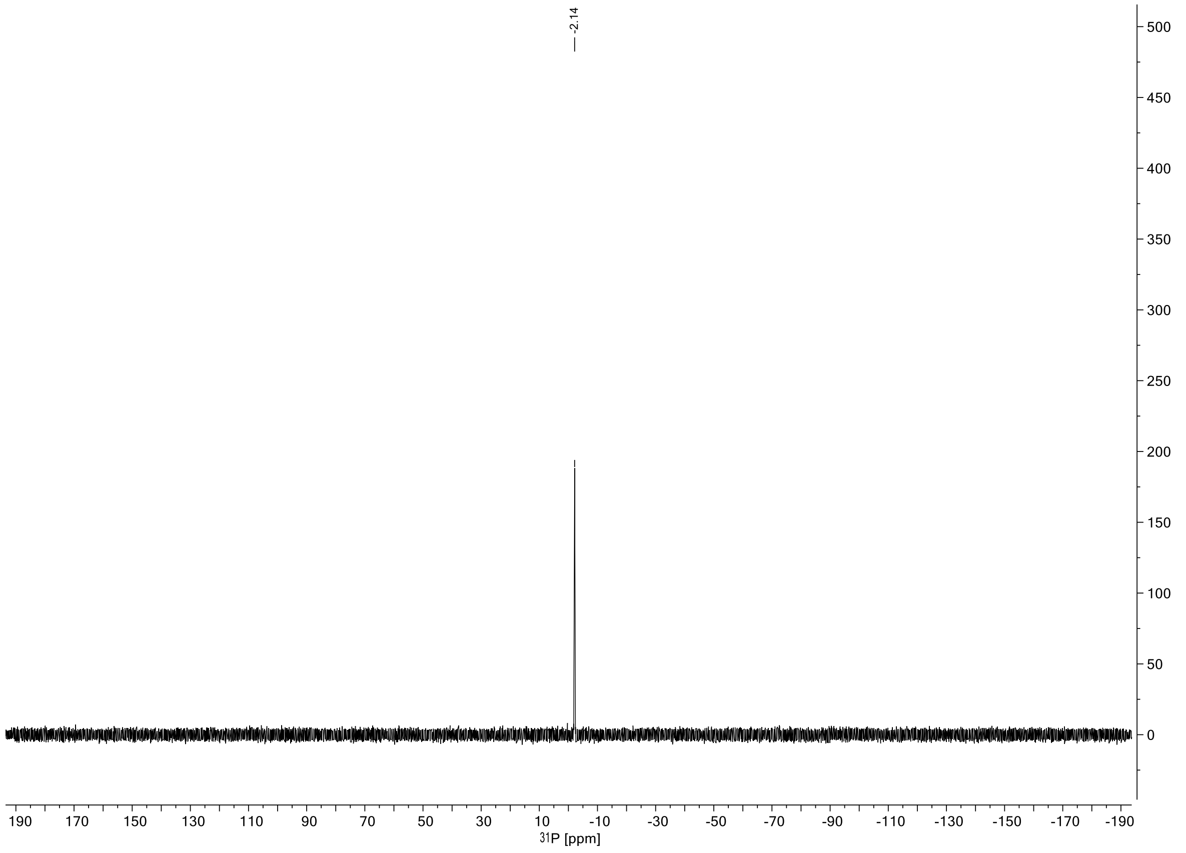

^{13}C NMR of compound **6l**

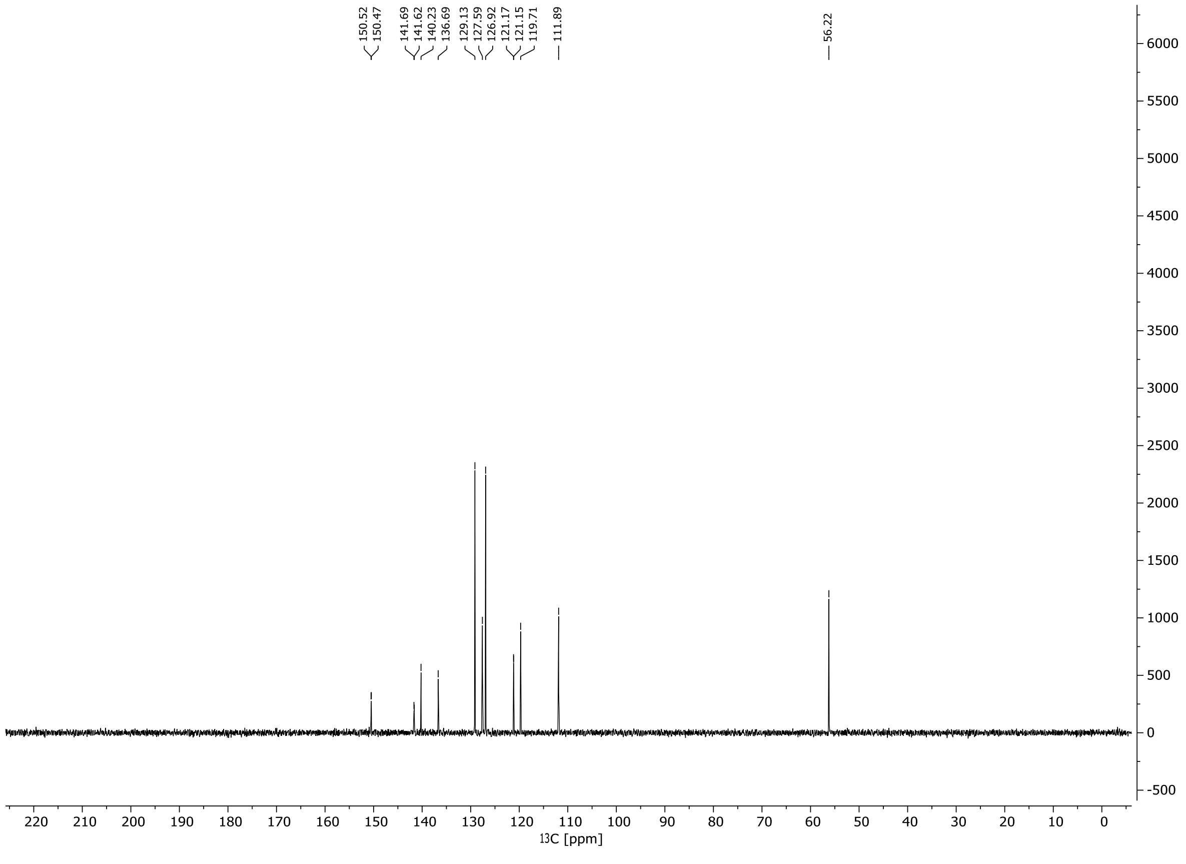

^{1}H NMR of compound **6m**

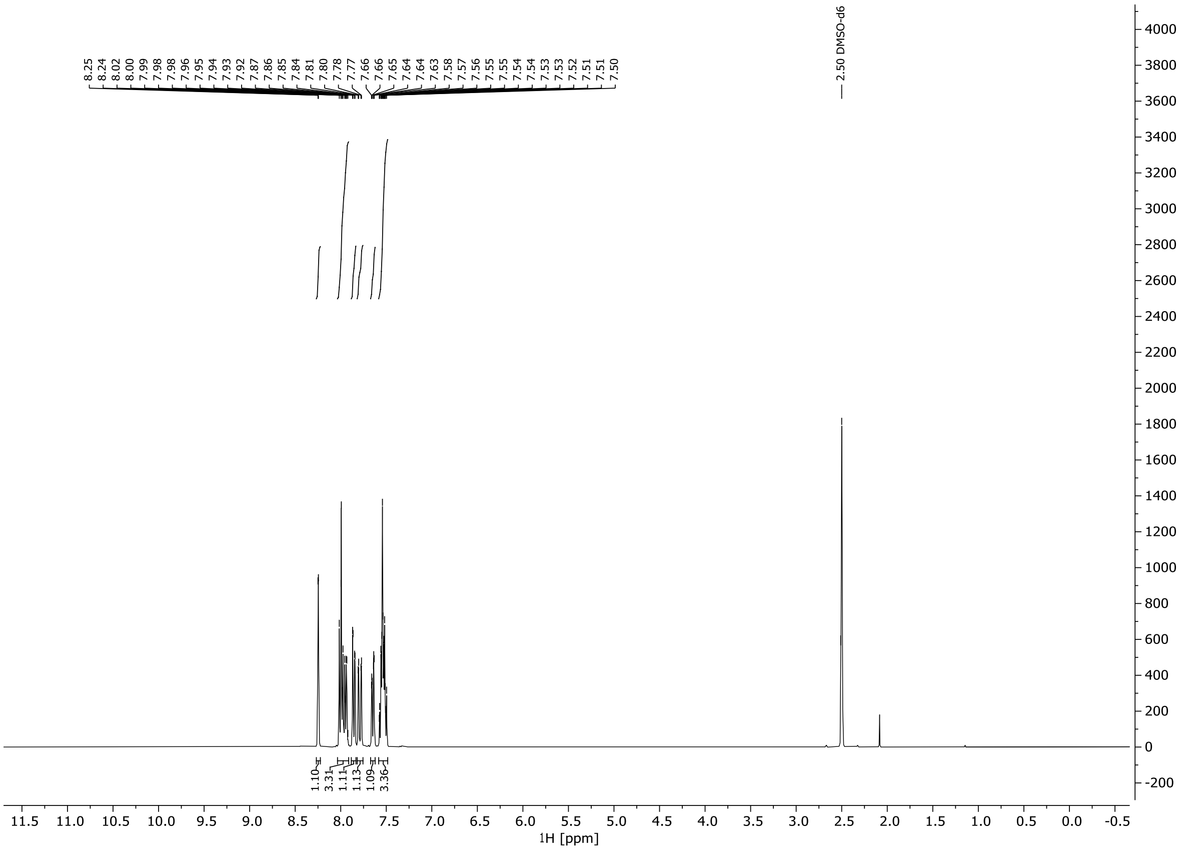

^{19}F NMR of compound **6m**

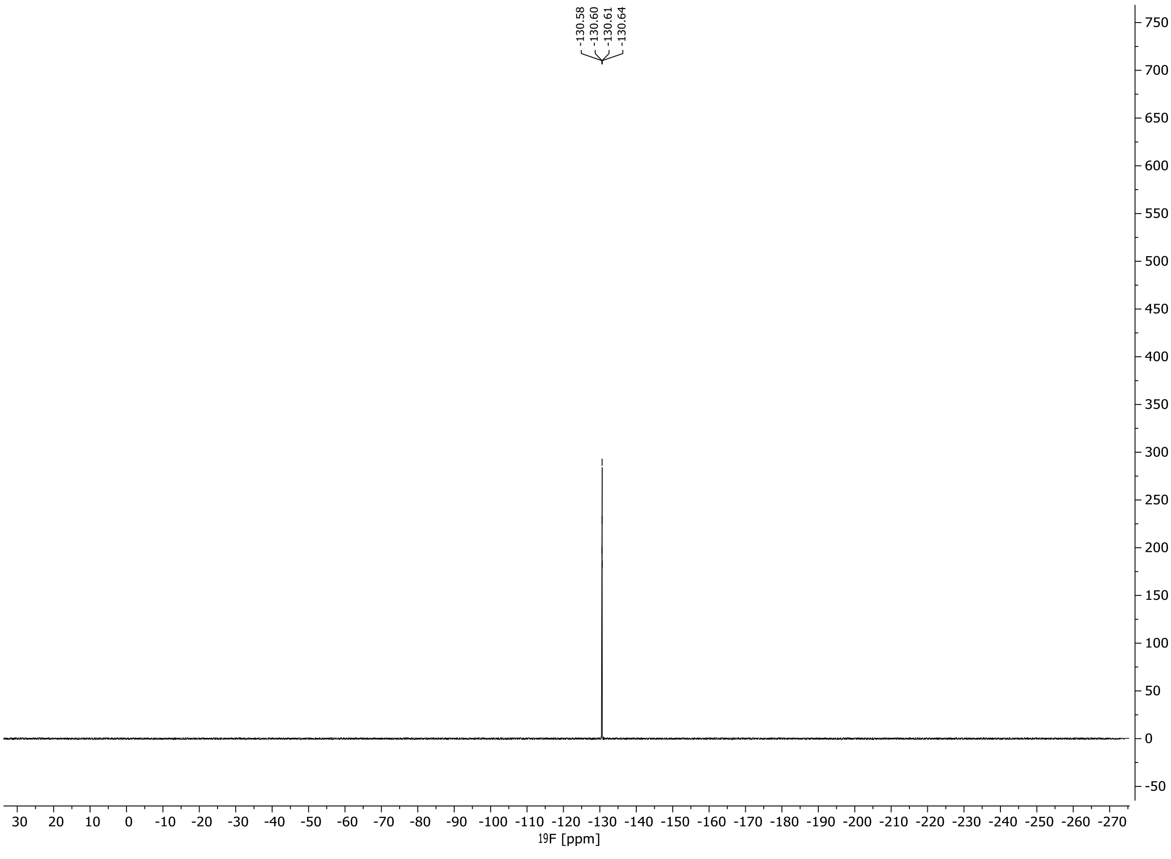

^{31}P NMR of compound **6m**

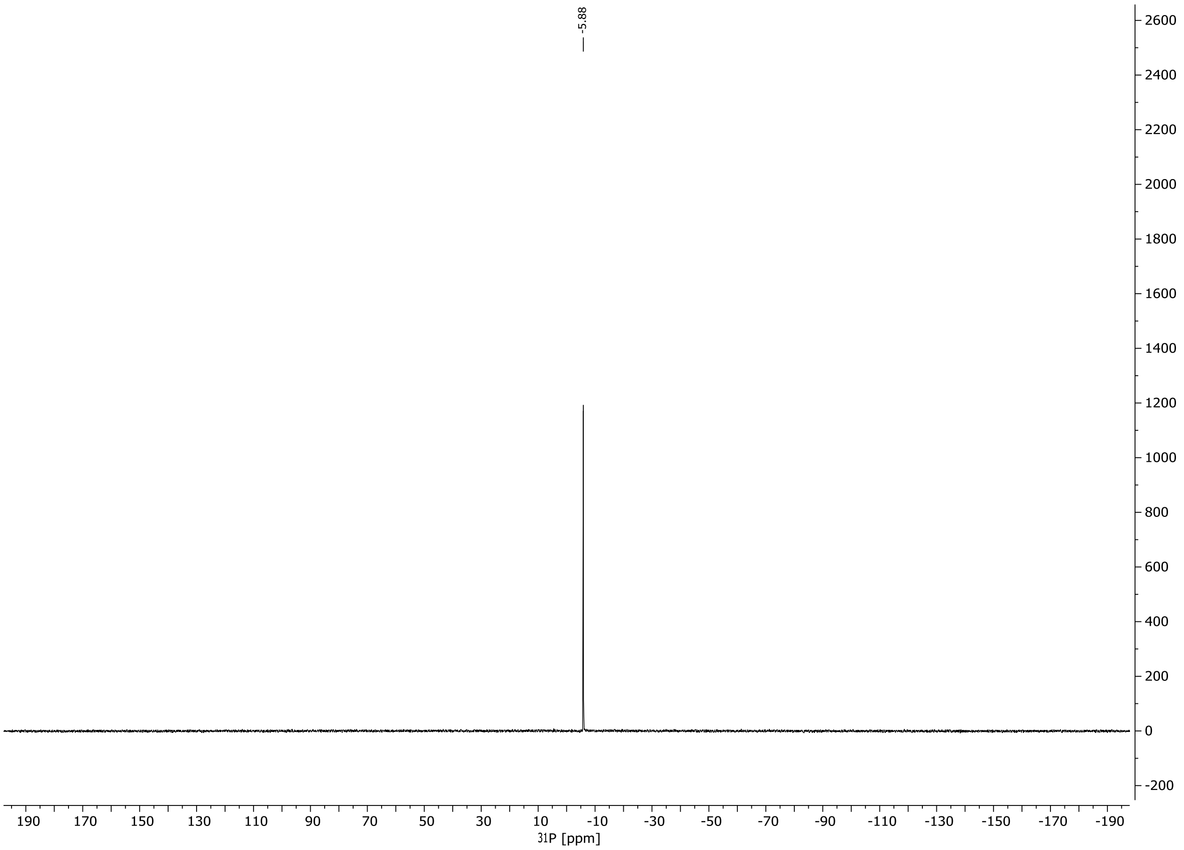

^{13}C NMR of compound **6m**

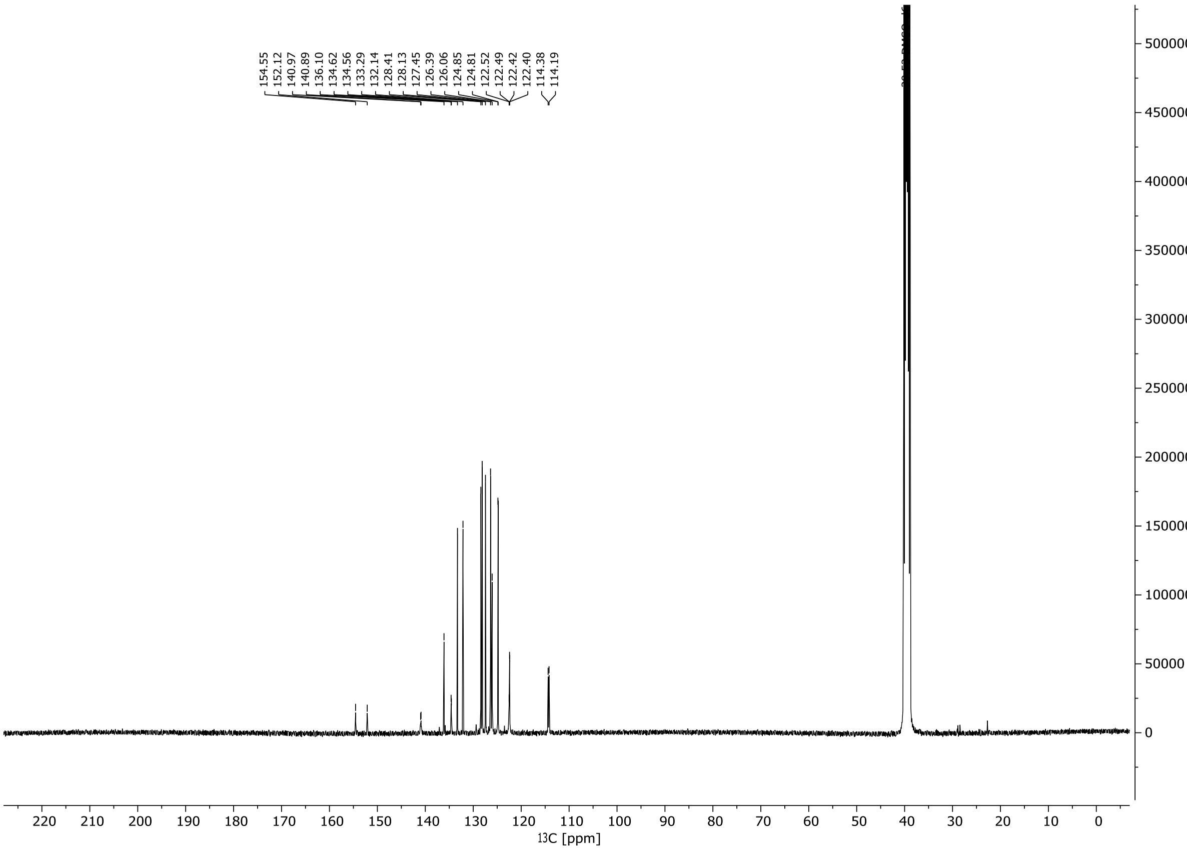

^{1}H NMR of compound **7**

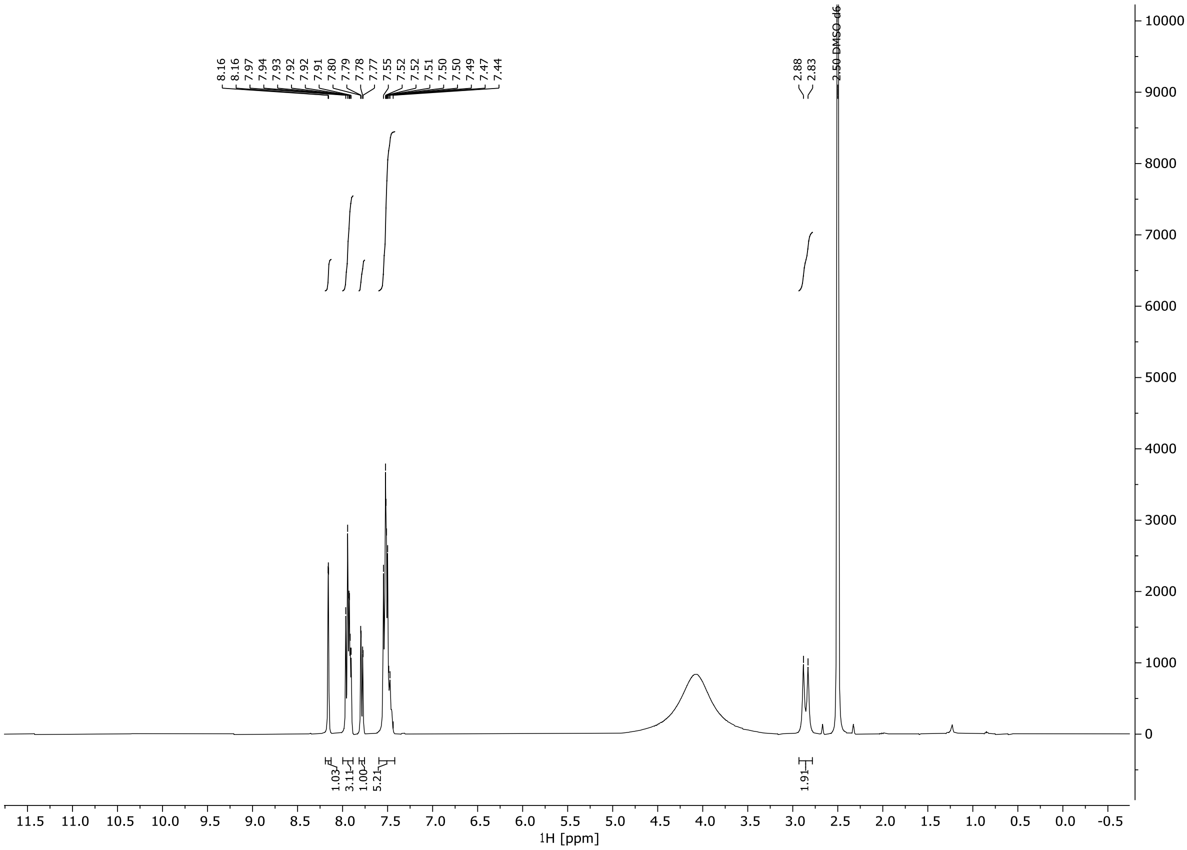

^{19}F NMR of compound **7**

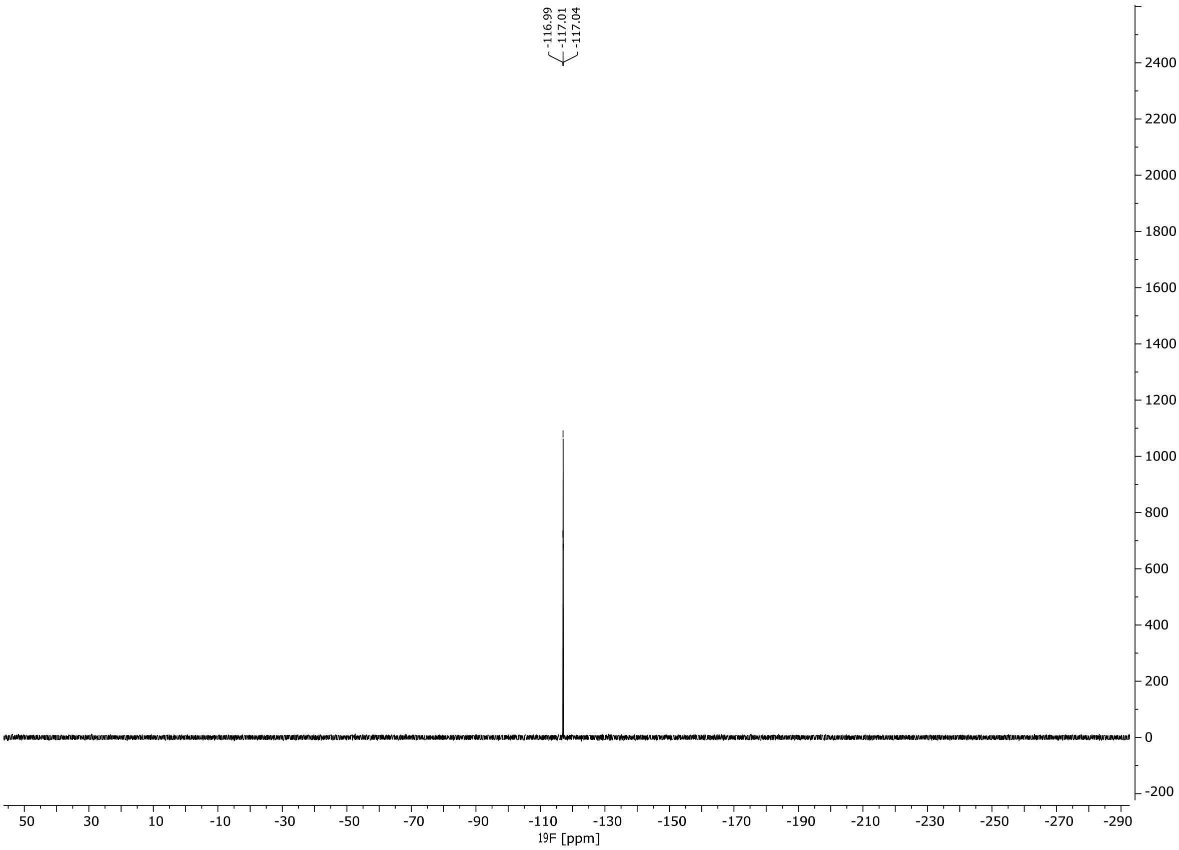

^{31}P NMR of compound **7**

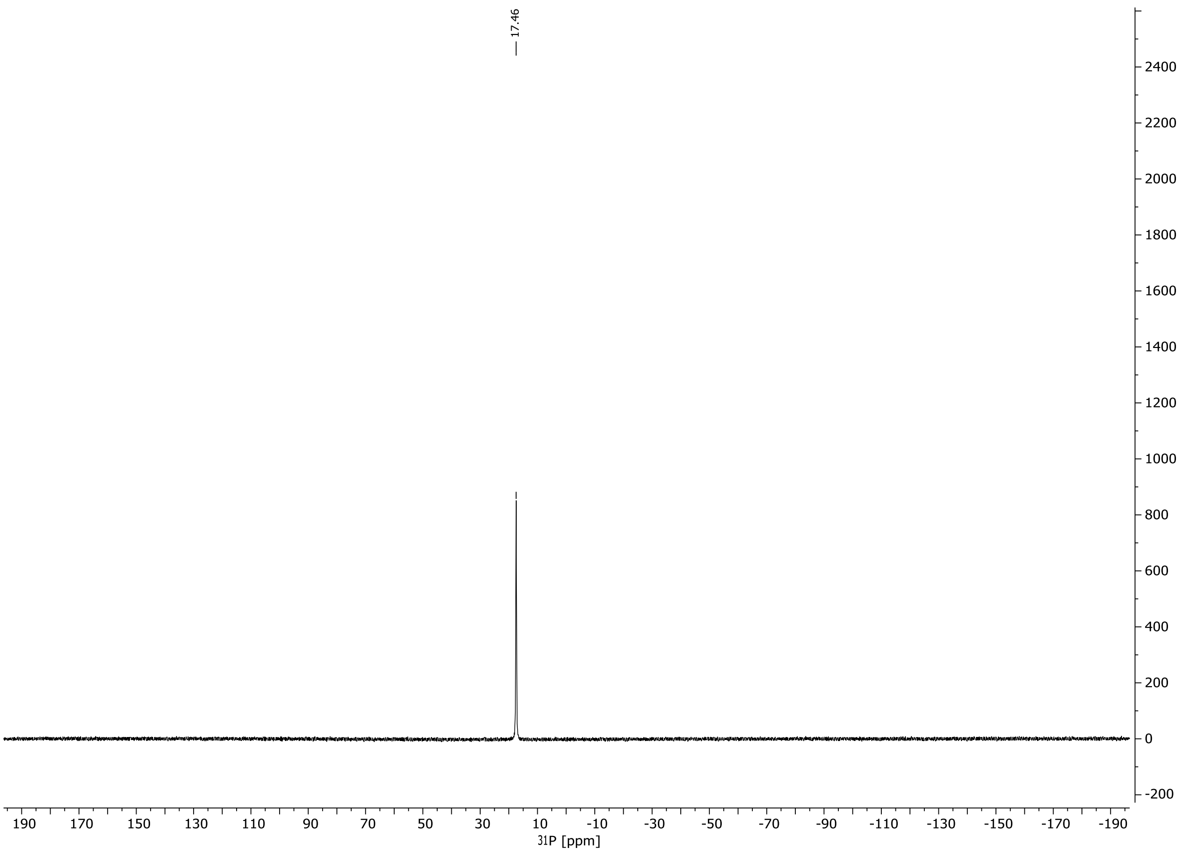

^{13}C NMR of compound **7**

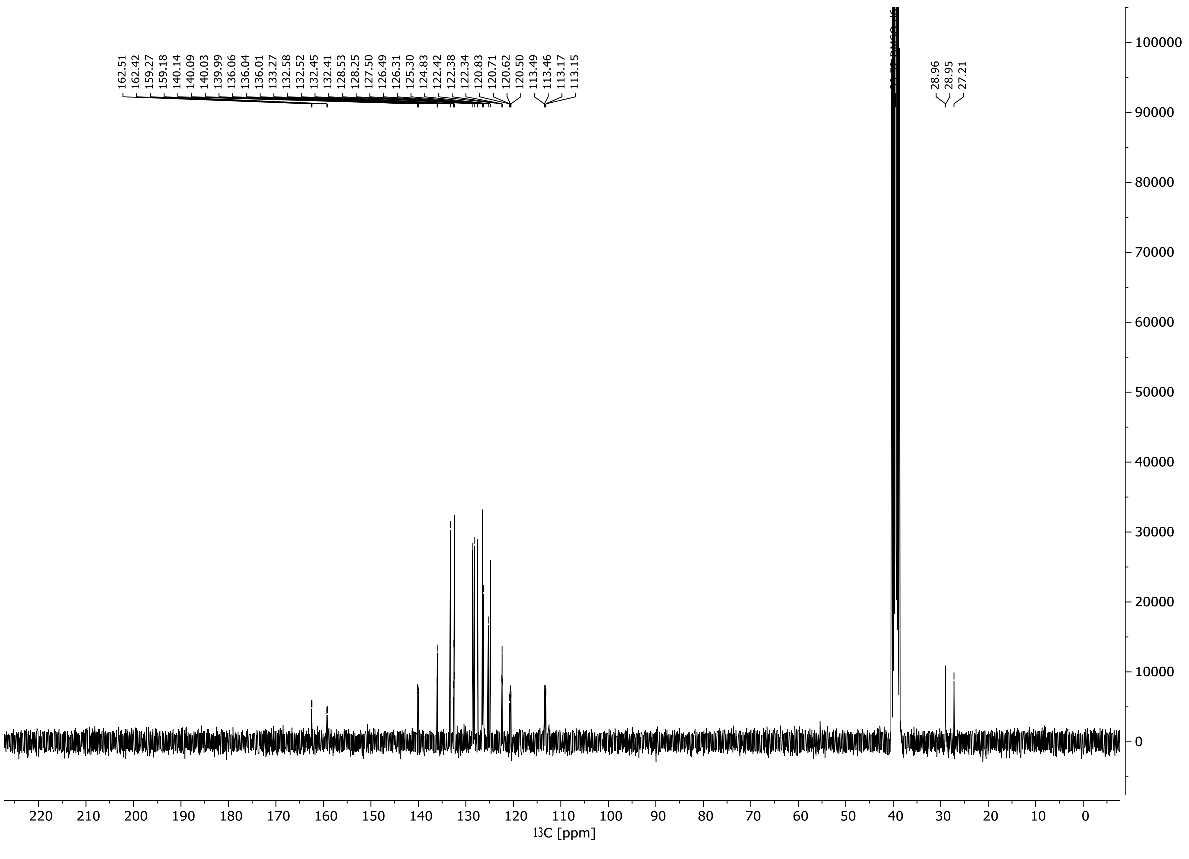

^{1}H NMR of compound **8a**

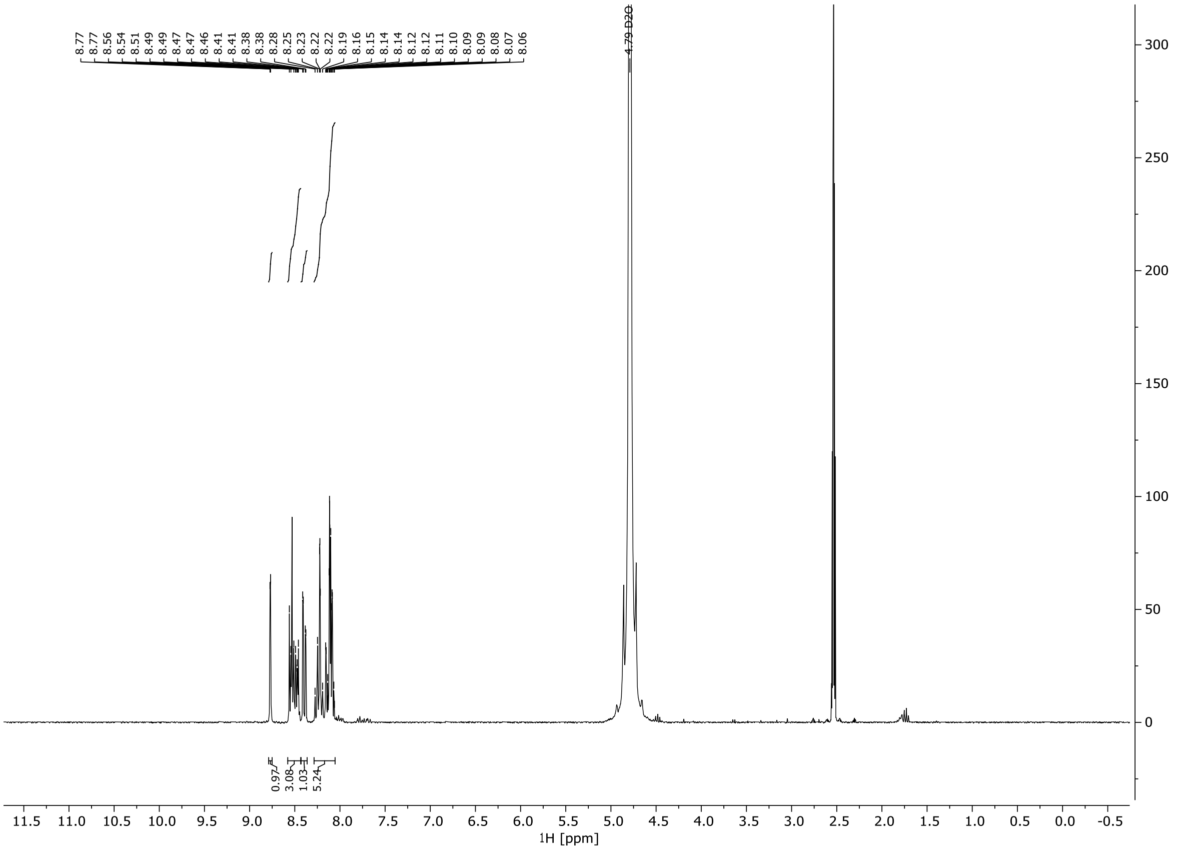

^{19}F NMR of compound **8a**

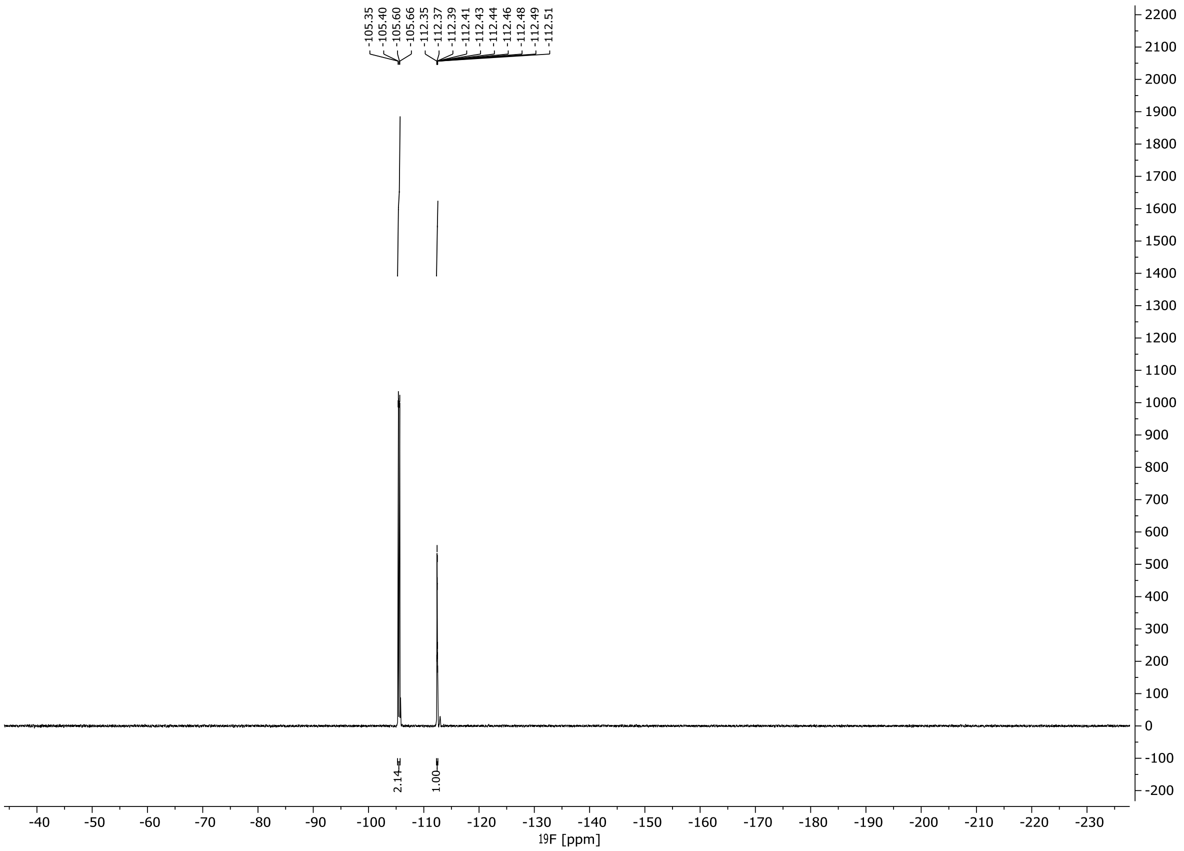

^{31}P NMR of compound **8a**

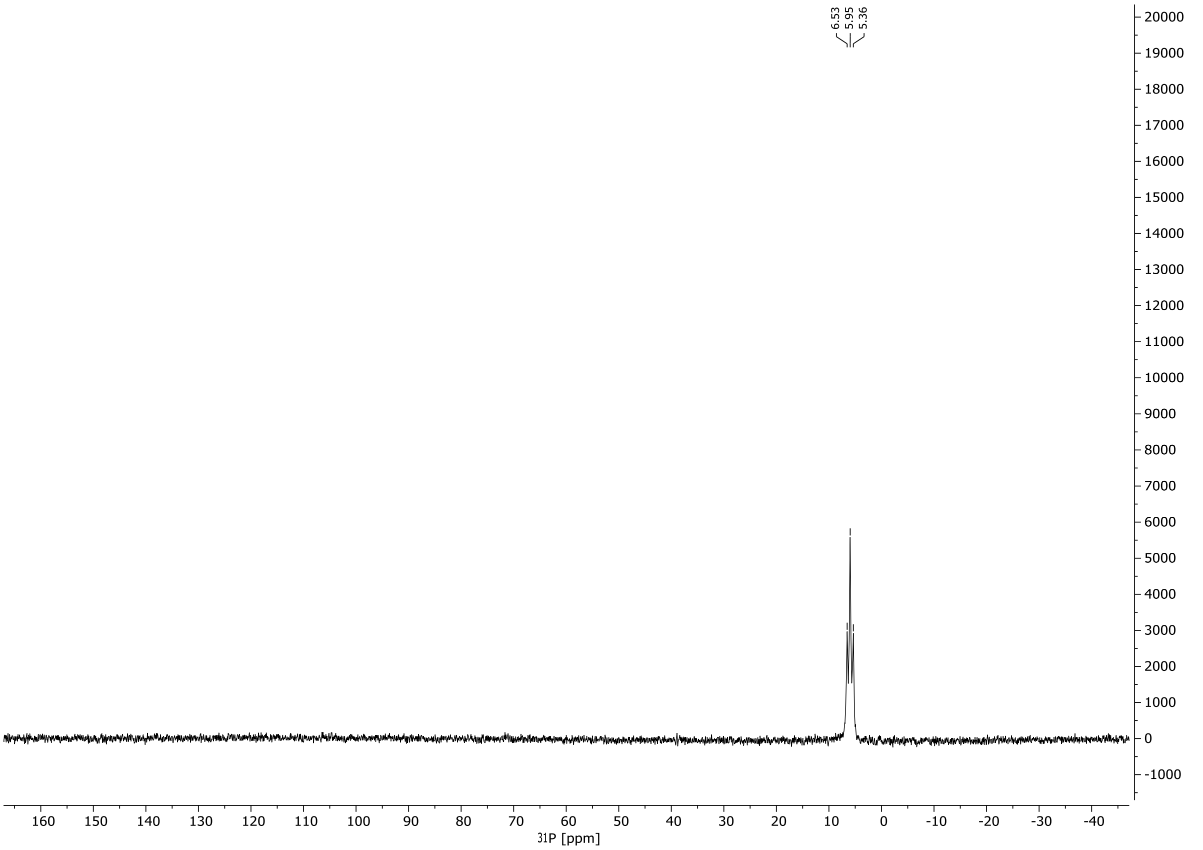

^{13}C NMR of compound **8a**

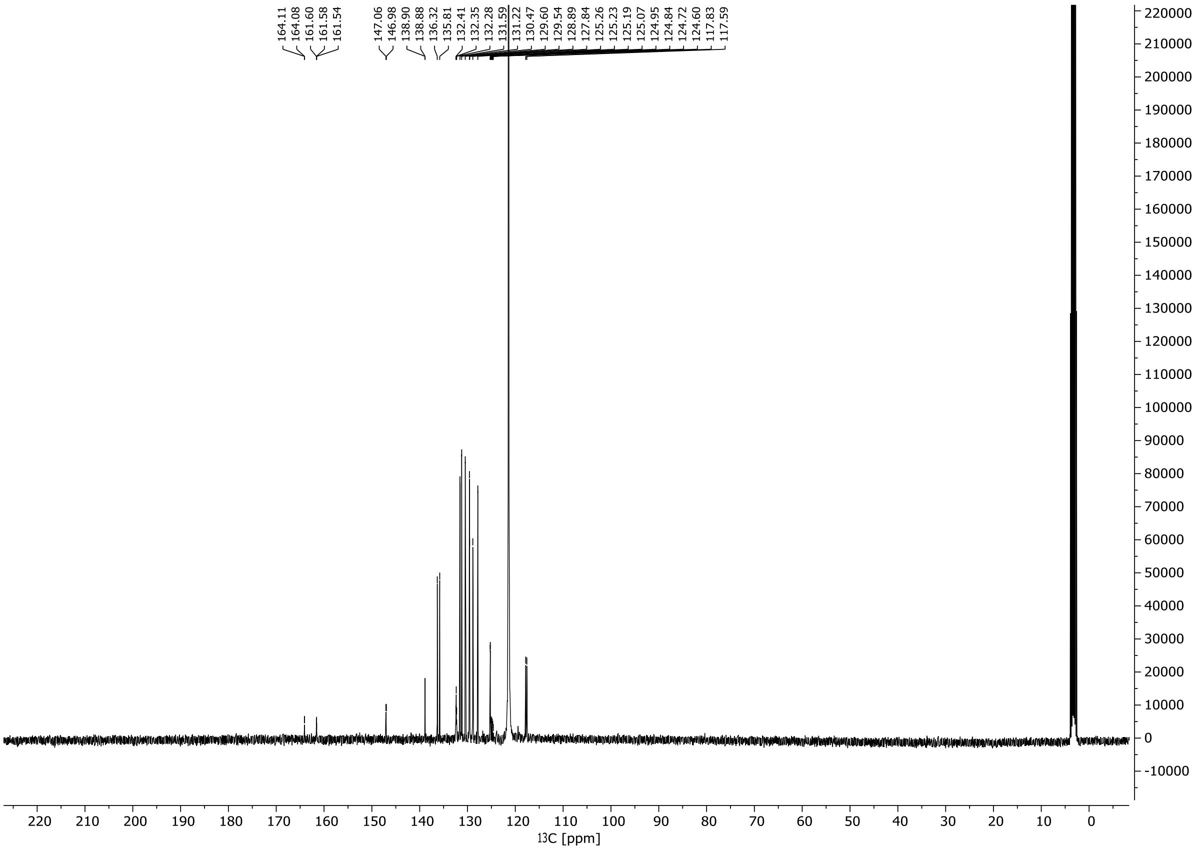

^{1}H NMR of compound **8b**

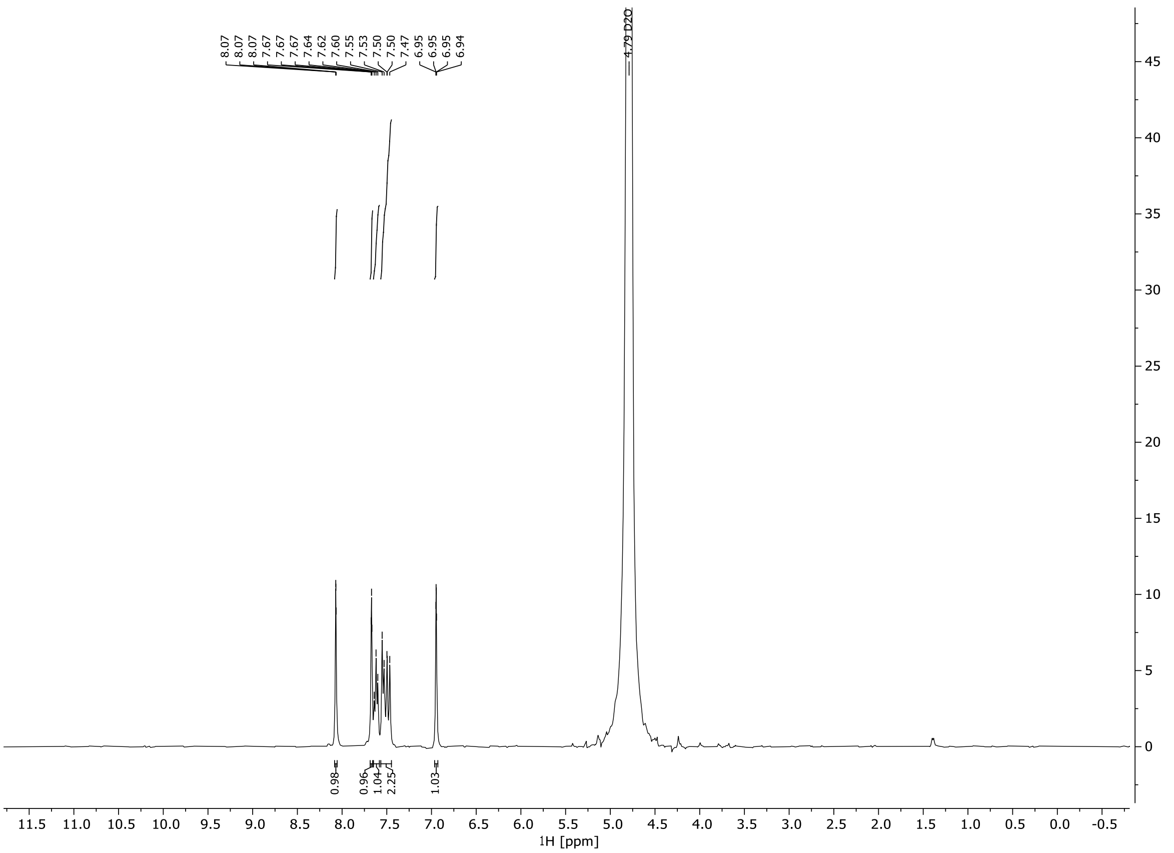

^{19}F NMR of compound **8b**

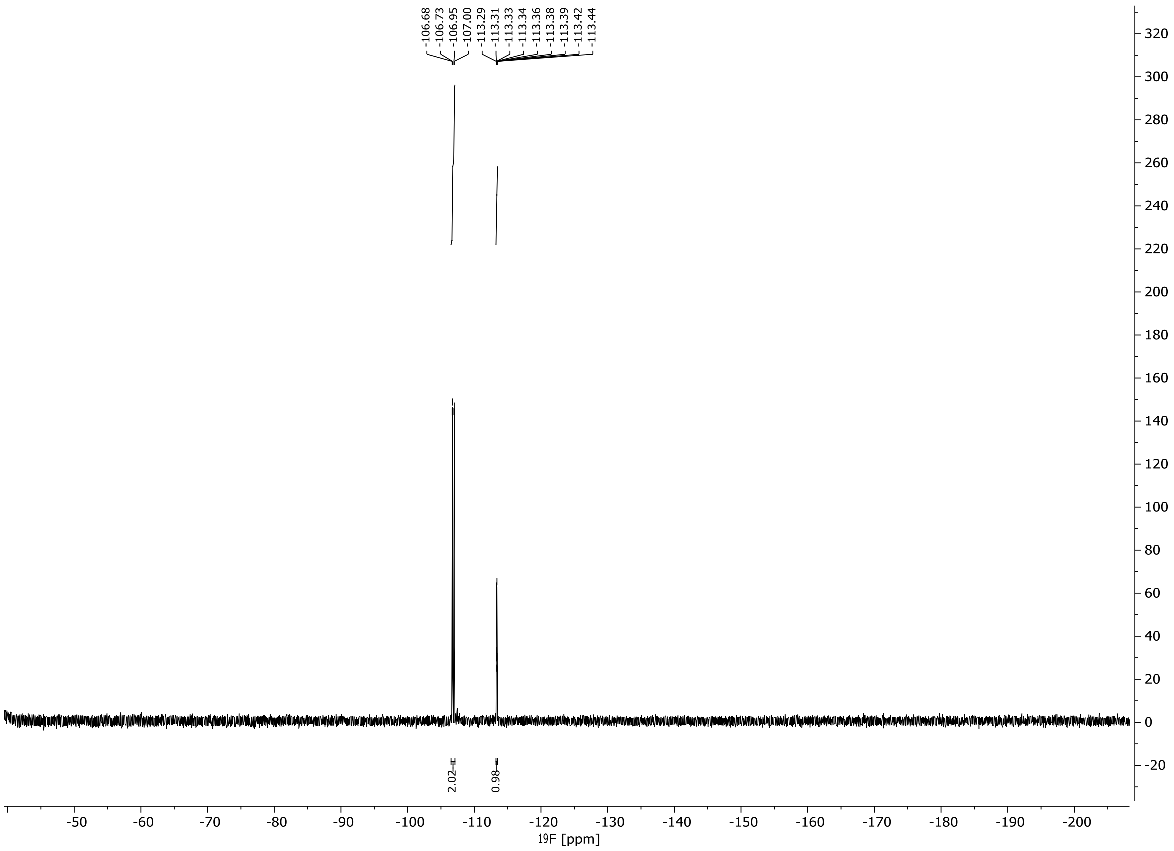

^{31}P NMR of compound **8b**

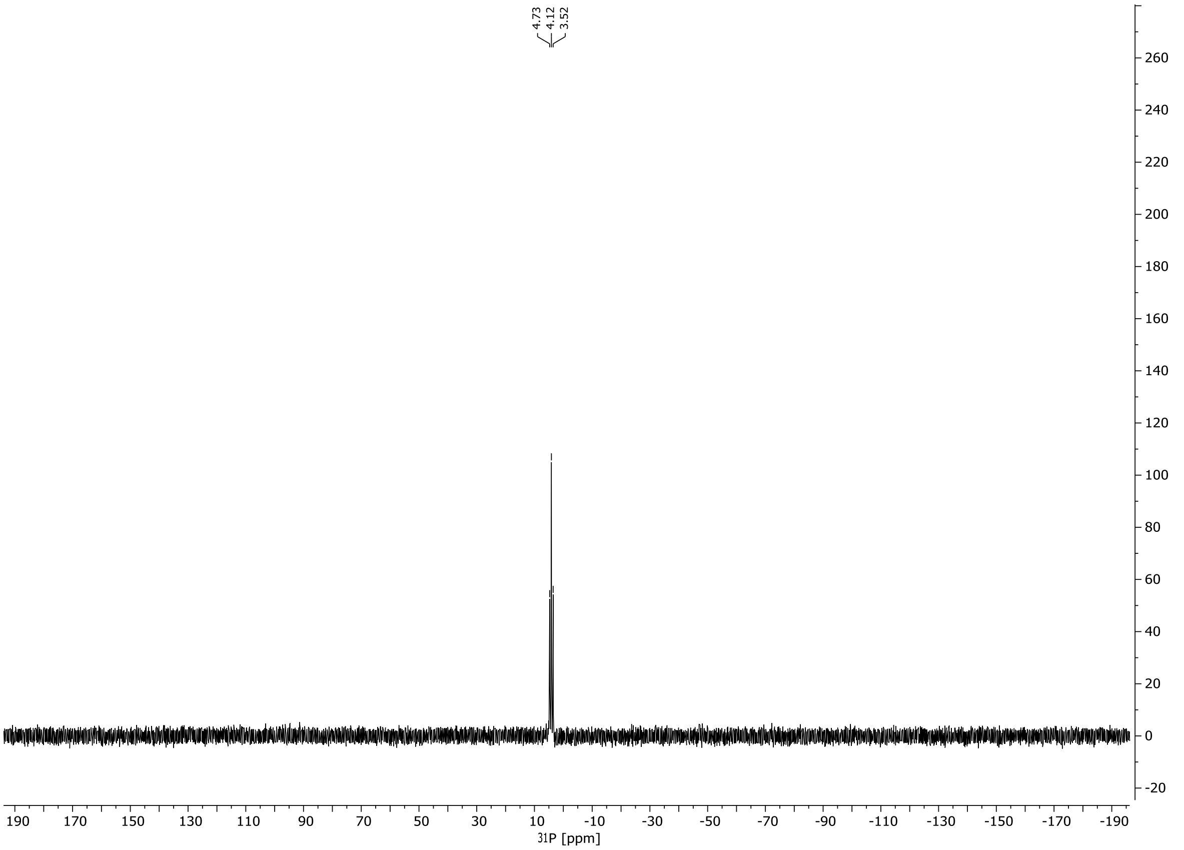

^{13}C NMR of compound **8b**

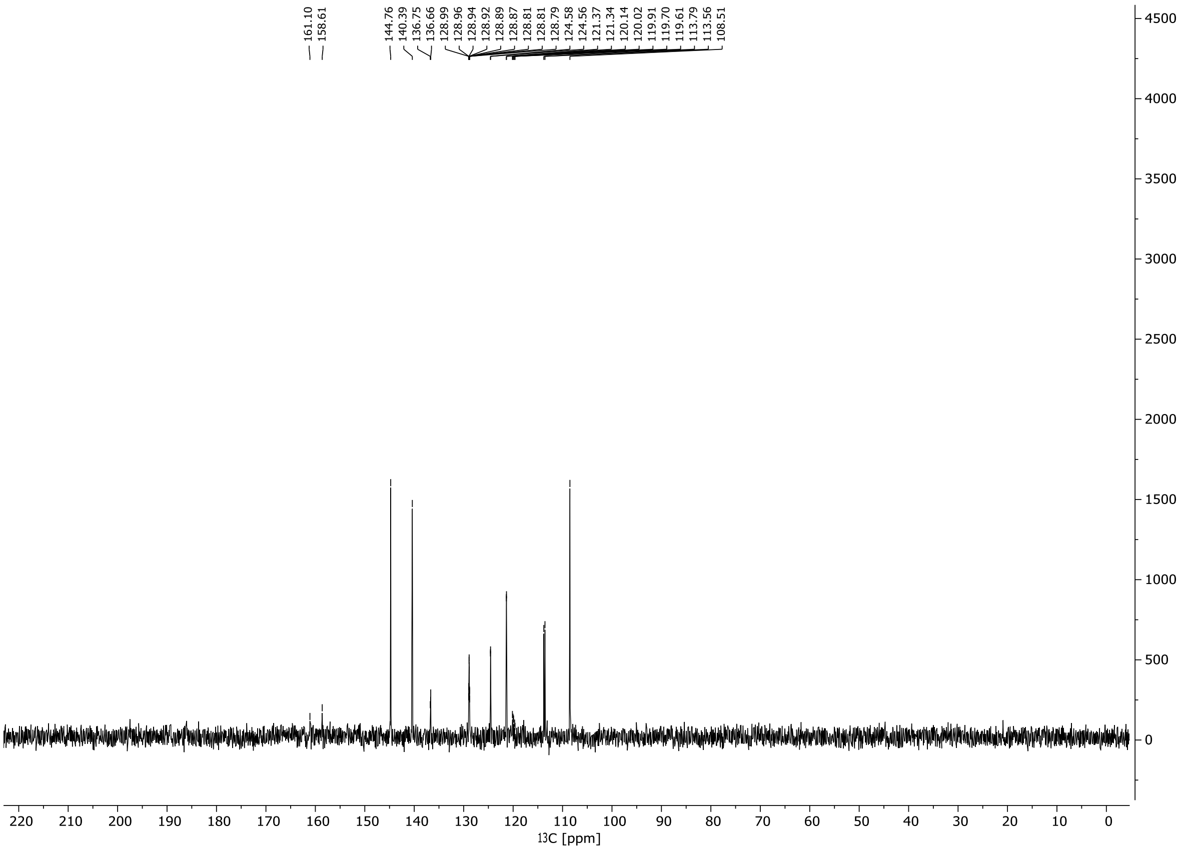

^{1}H NMR of compound **8c**

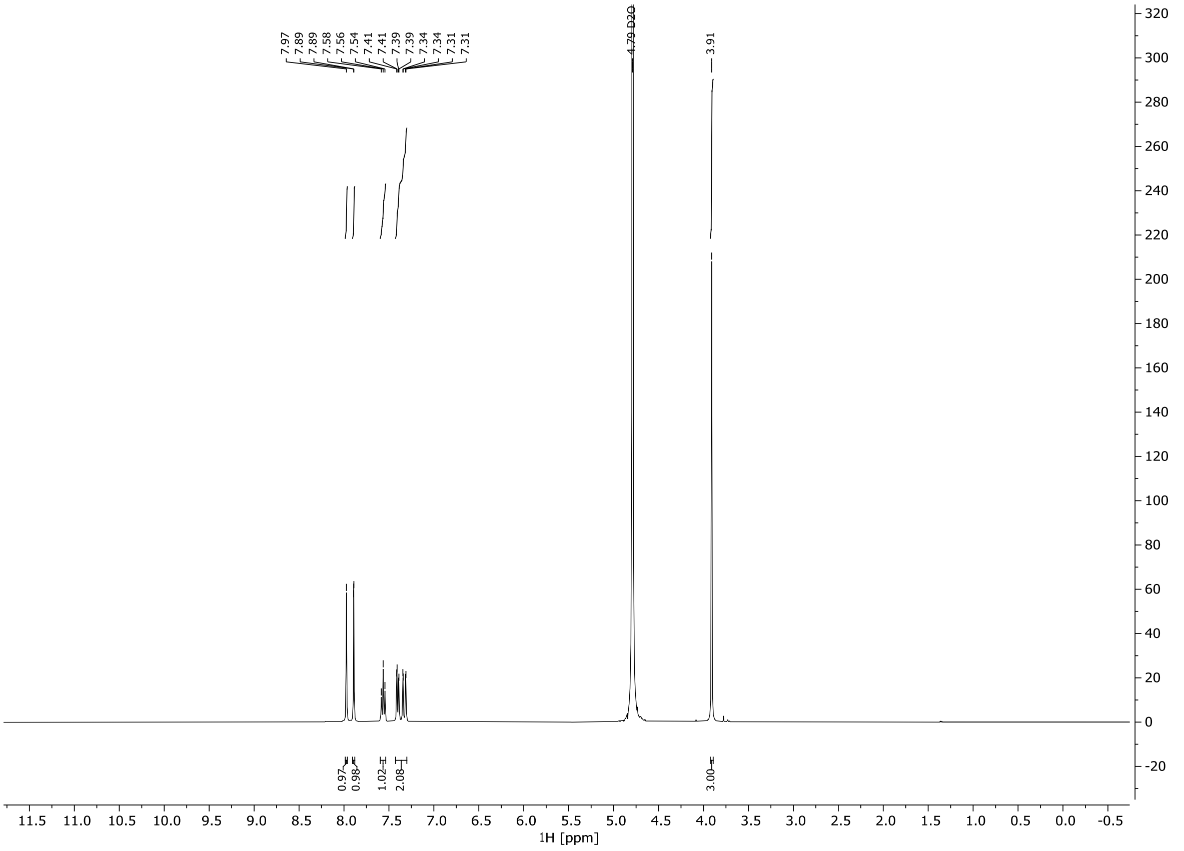

^{19}F NMR of compound **8c**

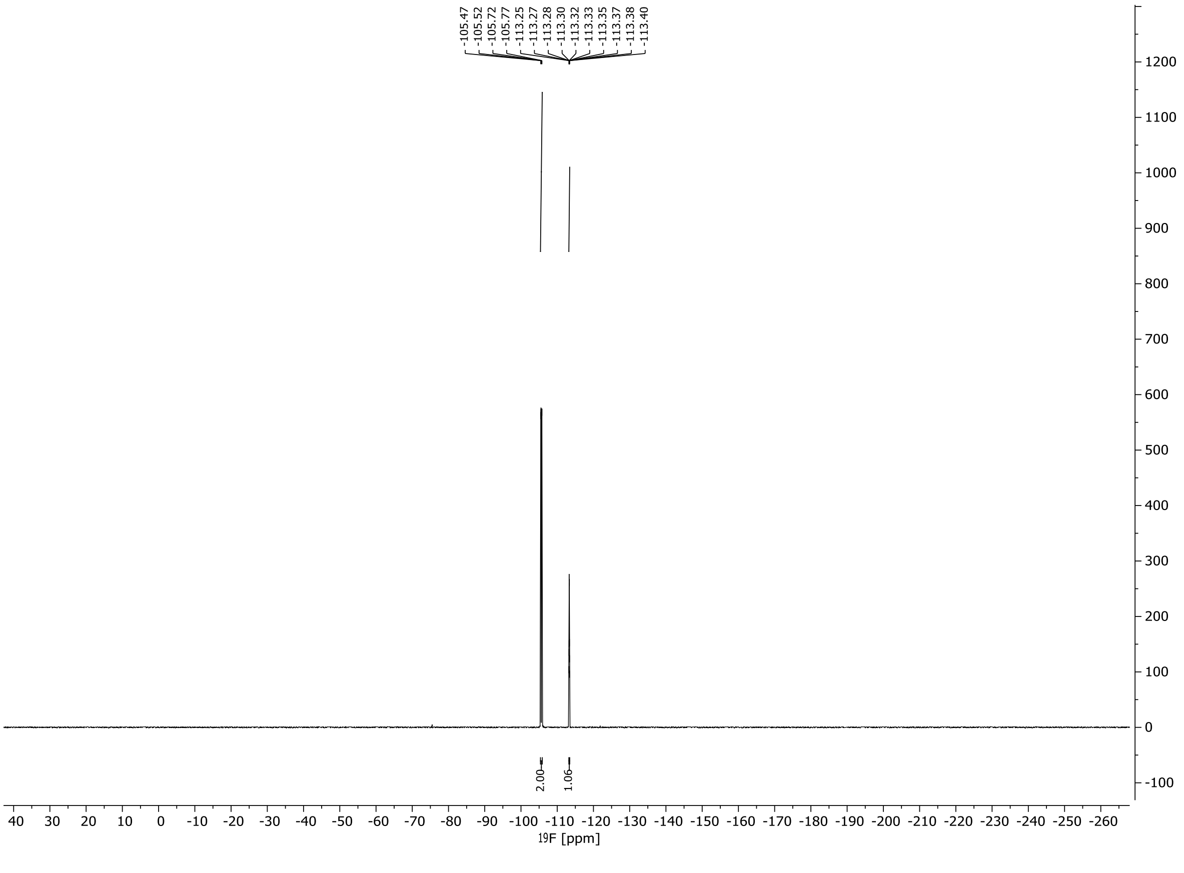

^{31}P NMR of compound **8c**

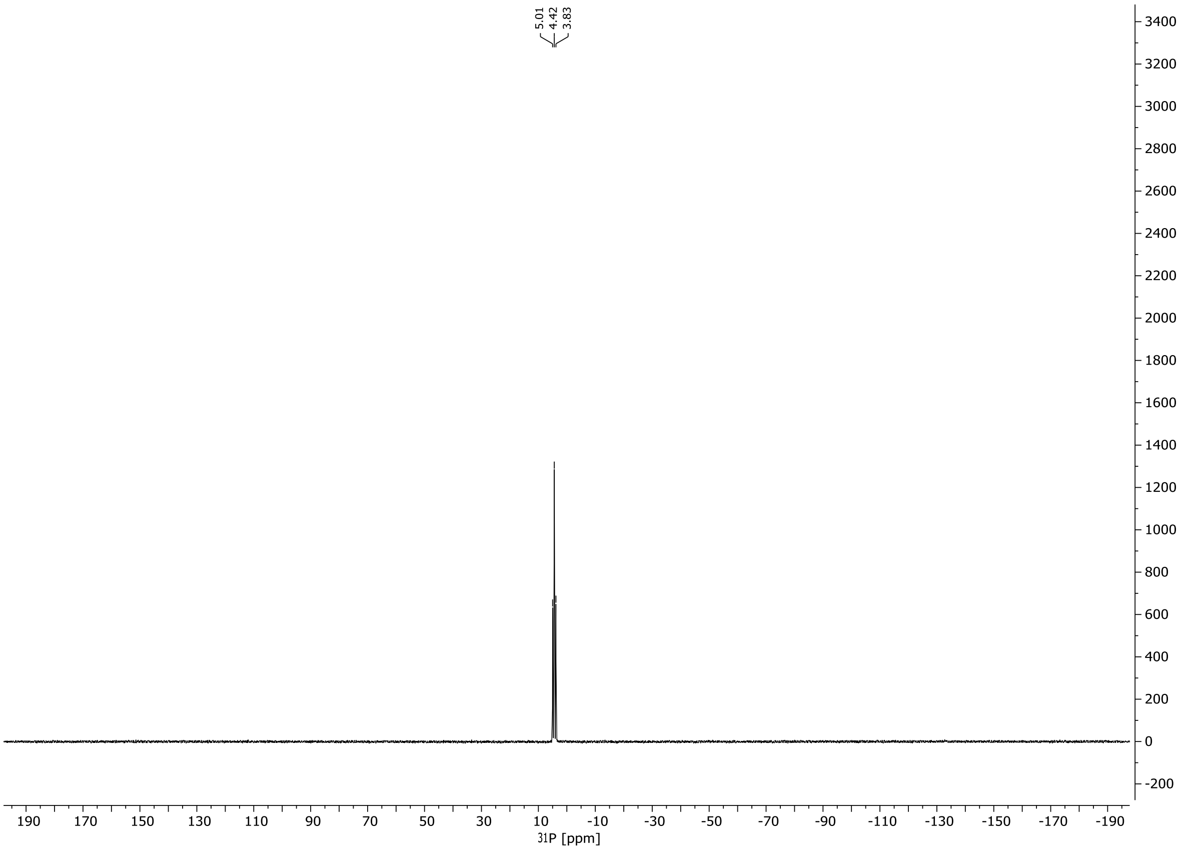

^{13}C NMR of compound **8c**

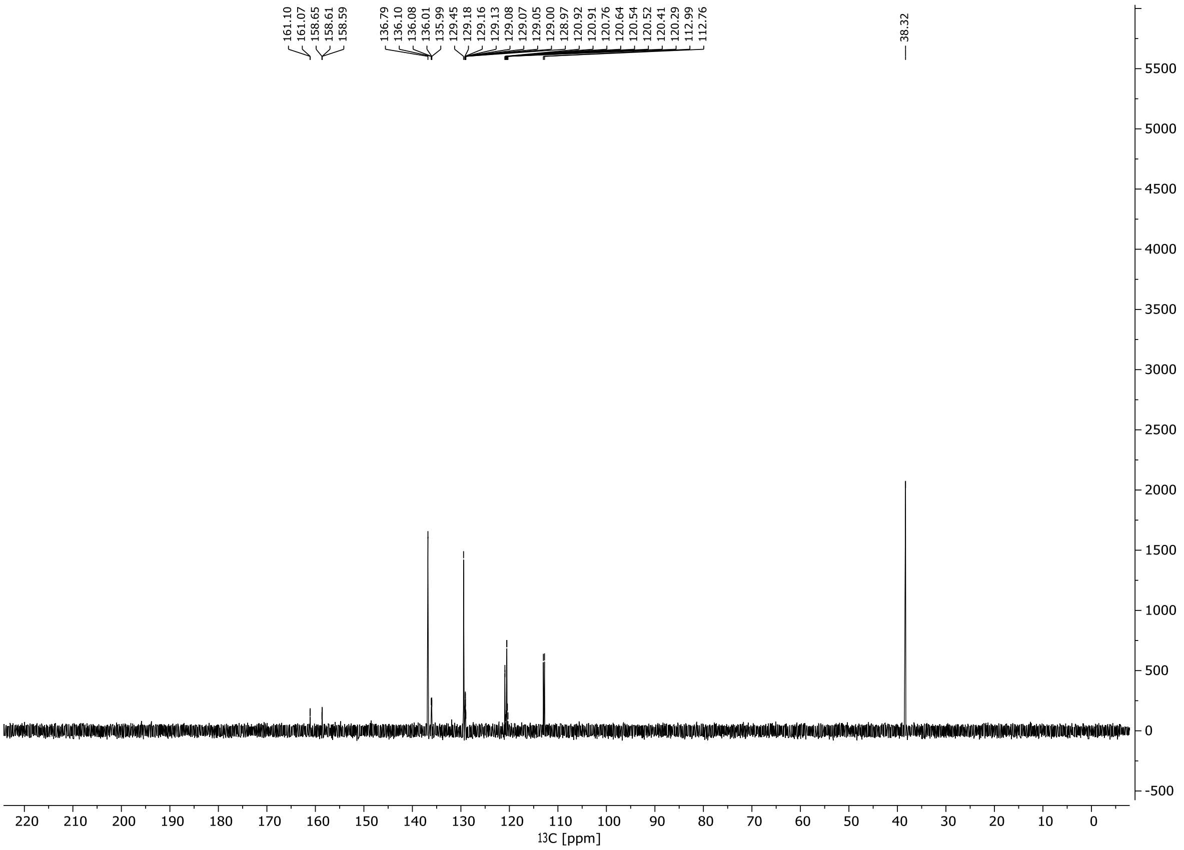

^{1}H NMR of compound **8d**

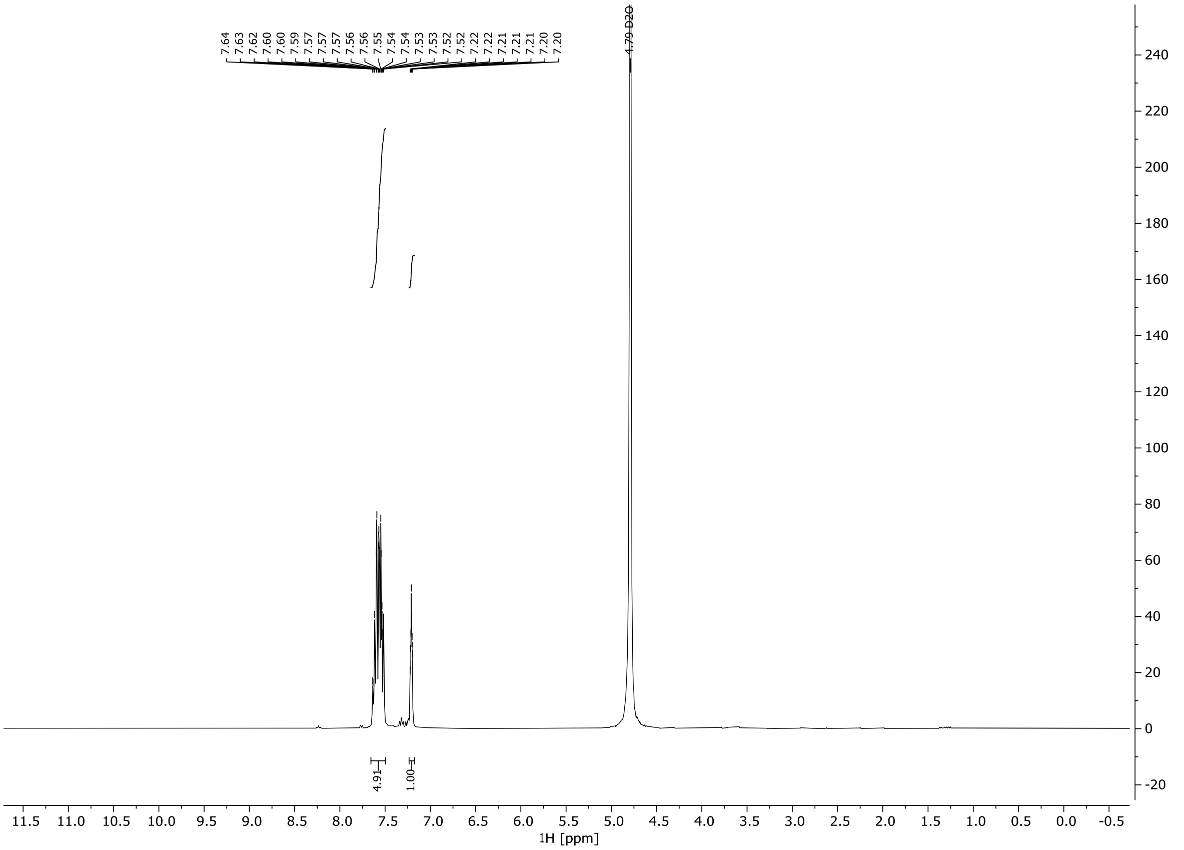

^{19}F NMR of compound **8d**

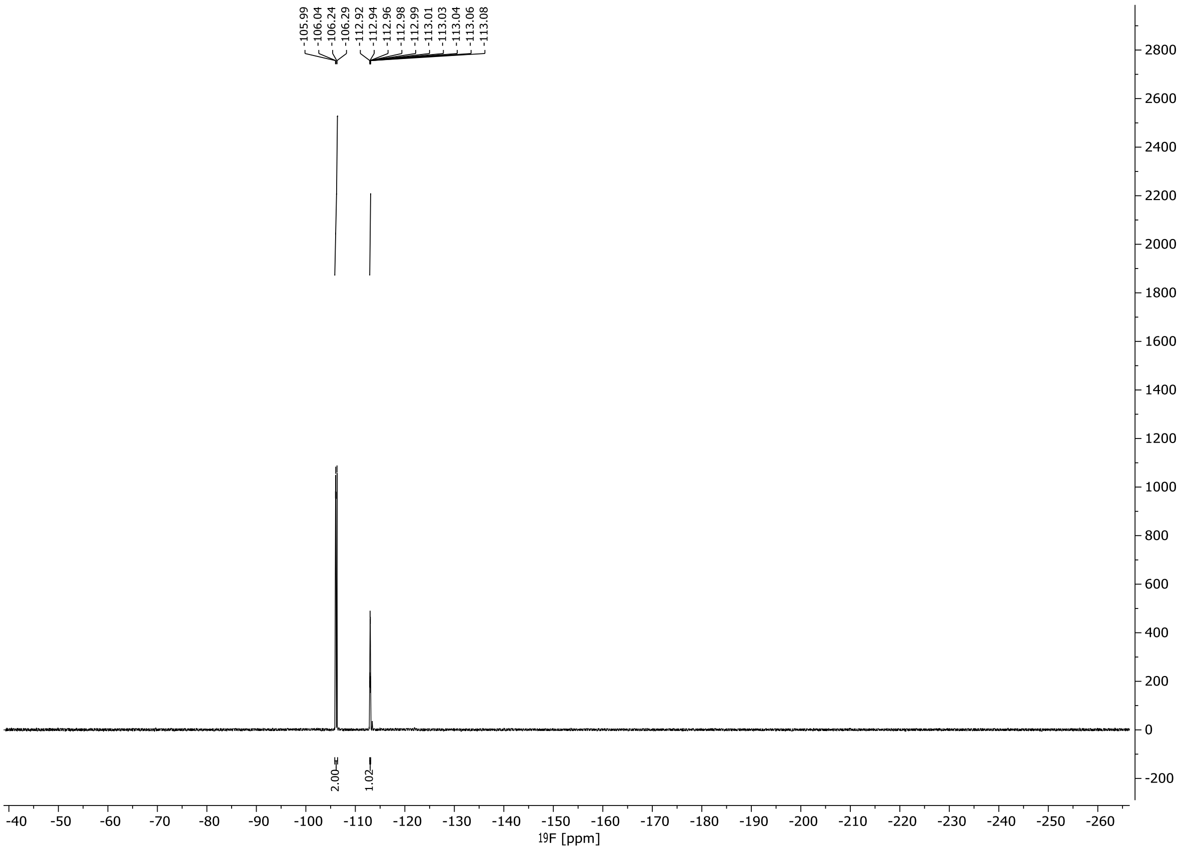

^{31}P NMR of compound **8d**

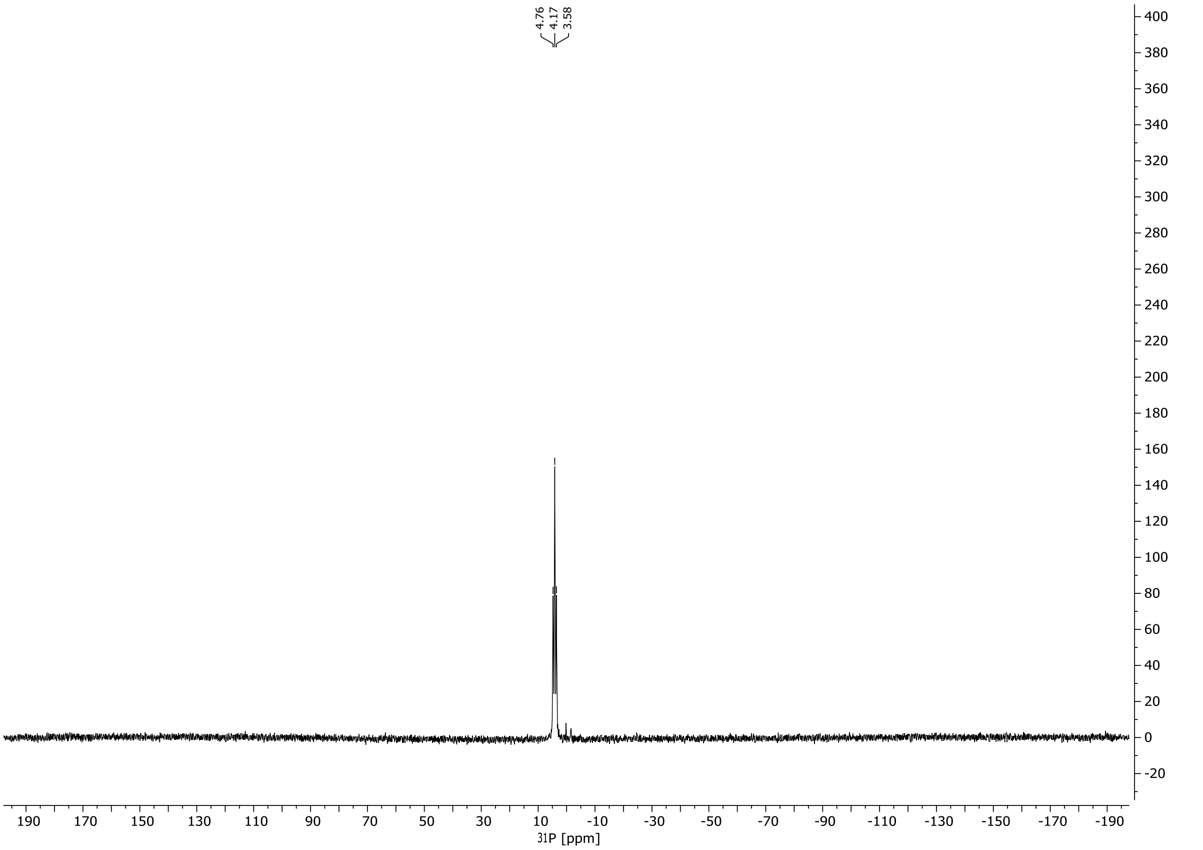

^{13}C NMR of compound **8d**

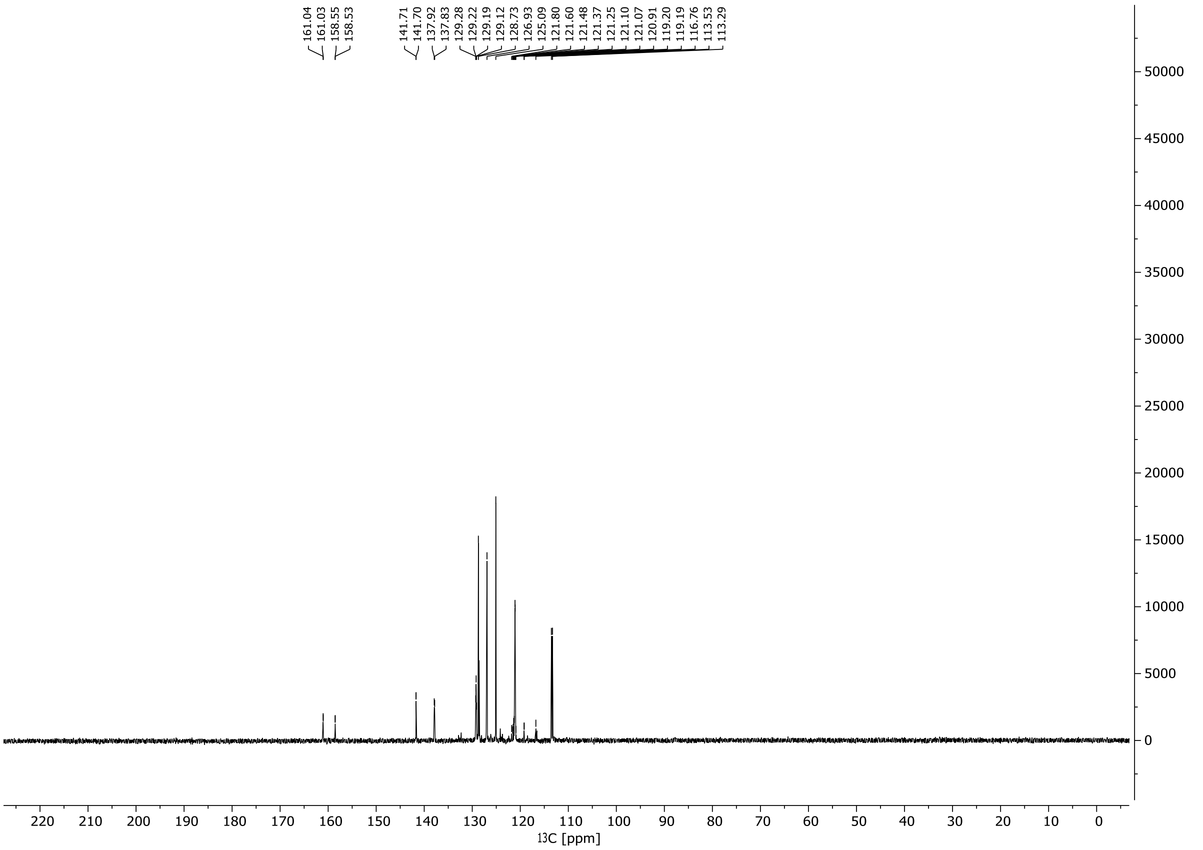

^{1}H NMR of compound **8e**

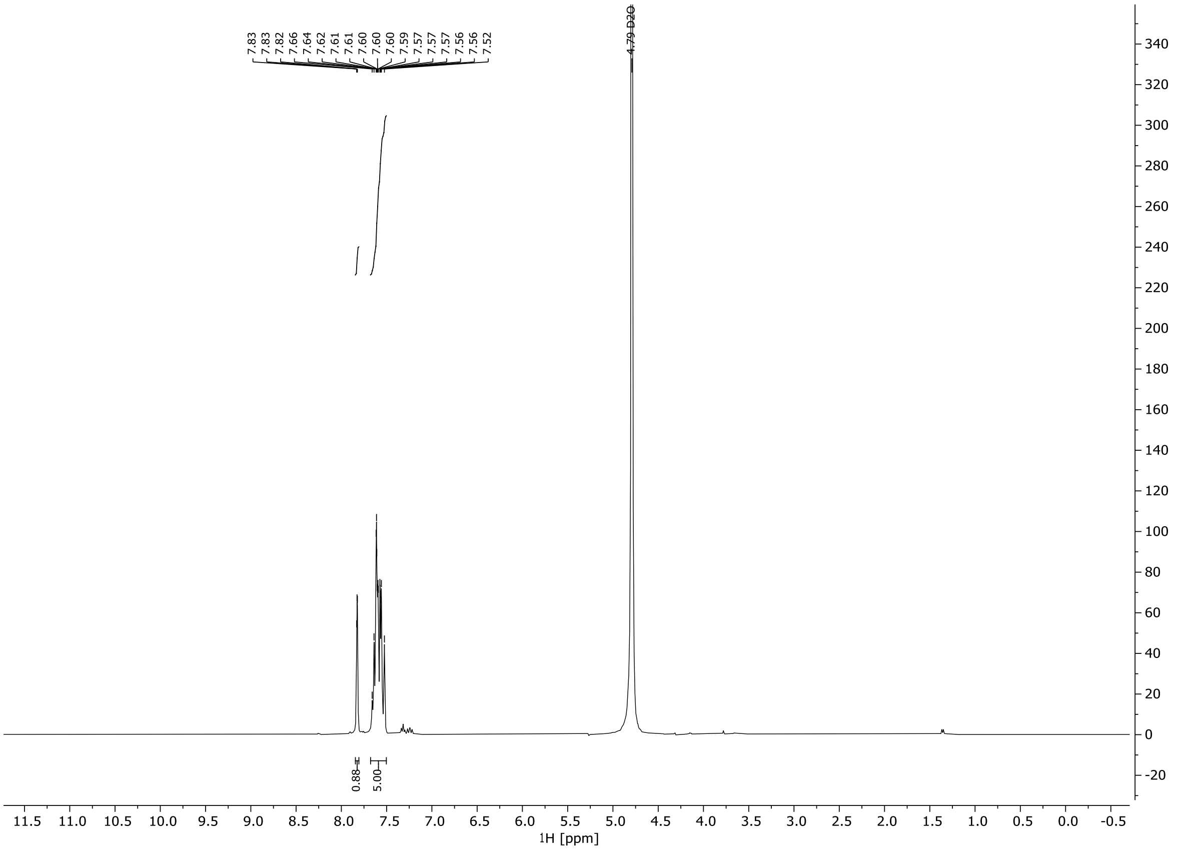

^{19}F NMR of compound **8e**

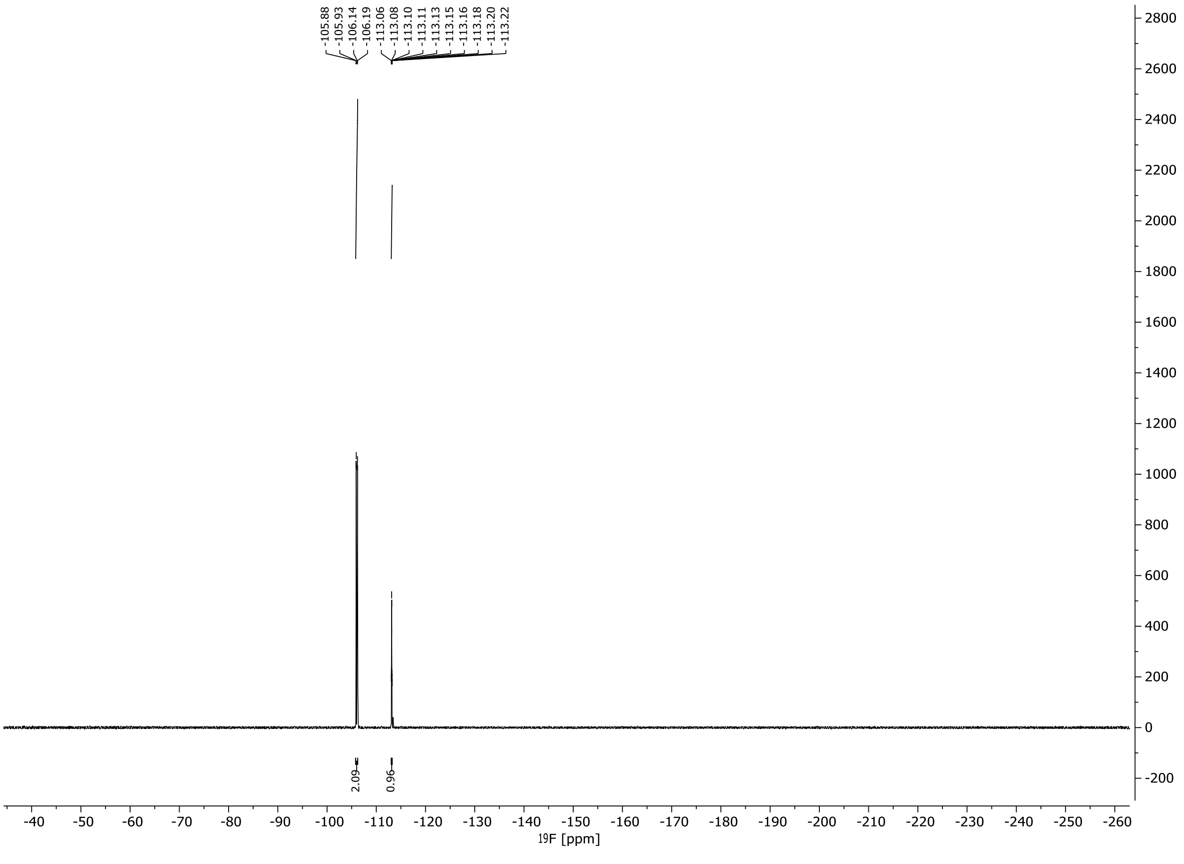

^{31}P NMR of compound **8e**

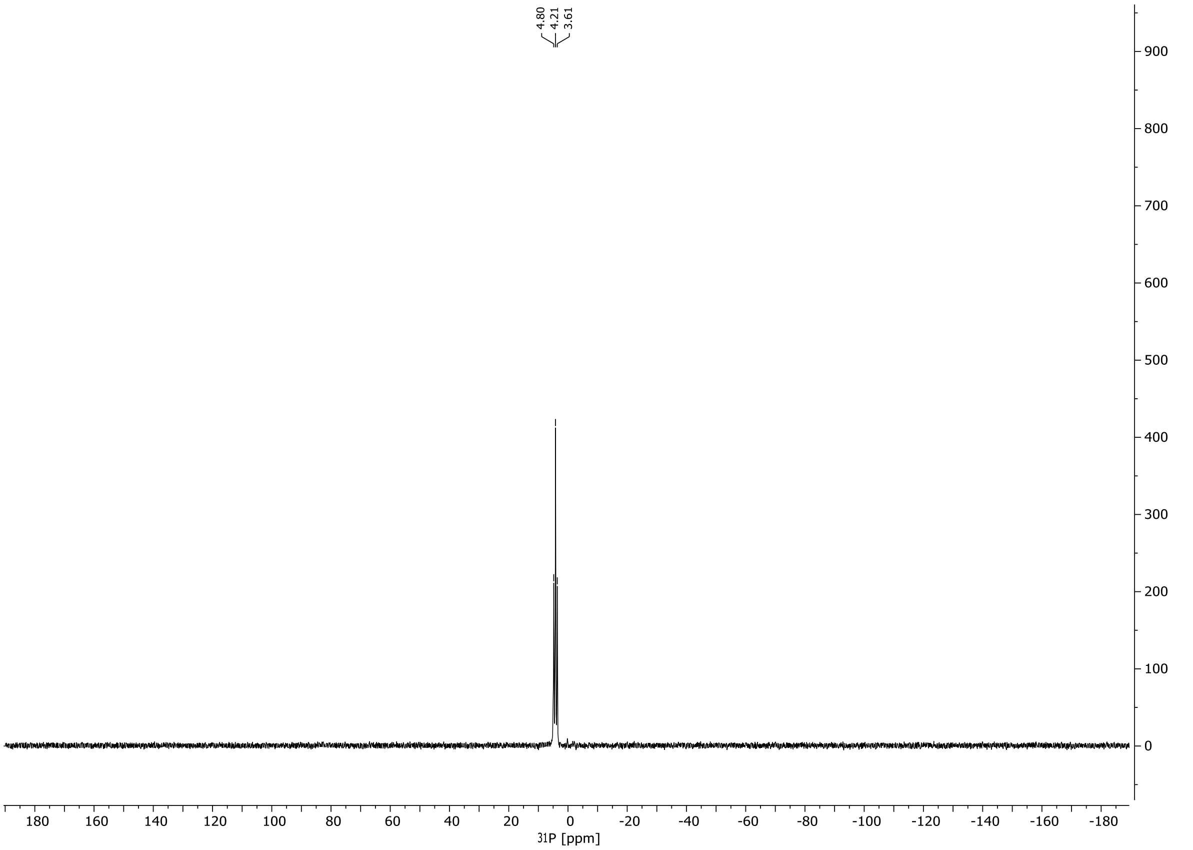

^{13}C NMR of compound **8e**

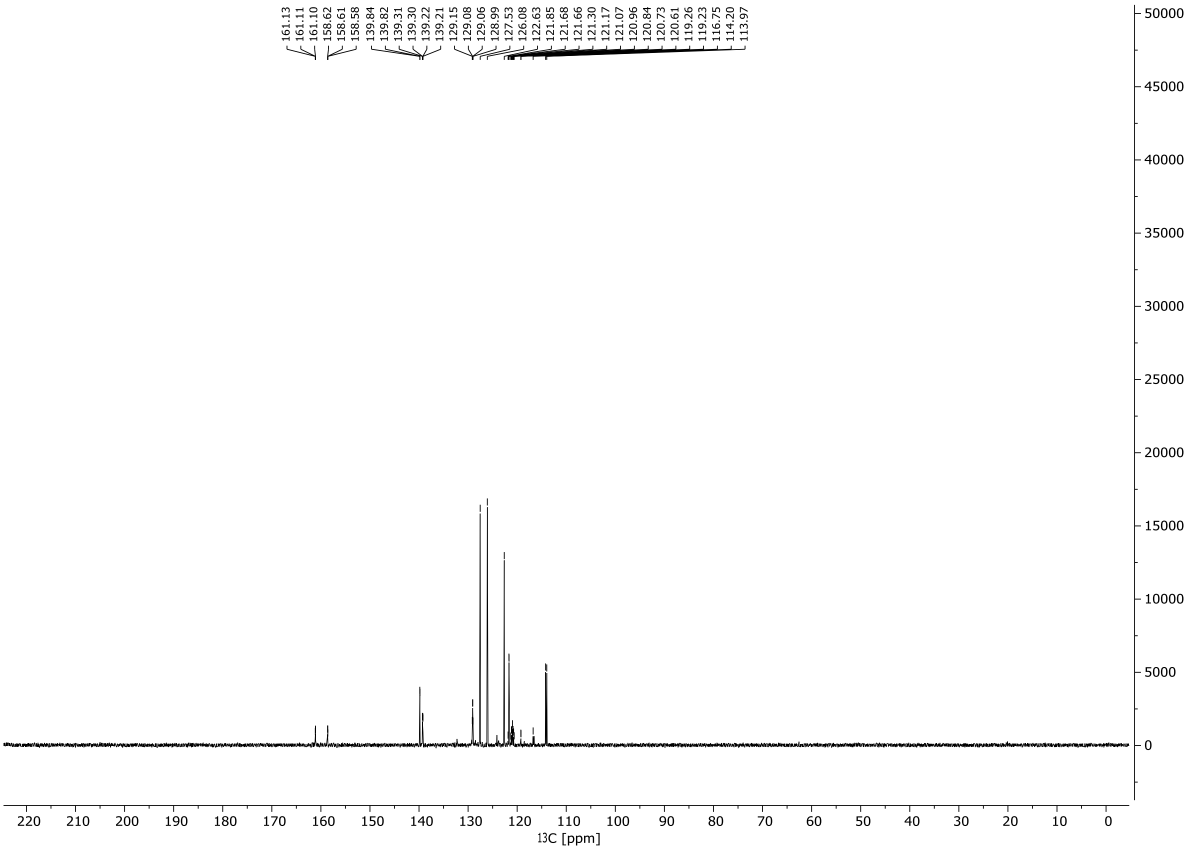

^{1}H NMR of compound **8f**

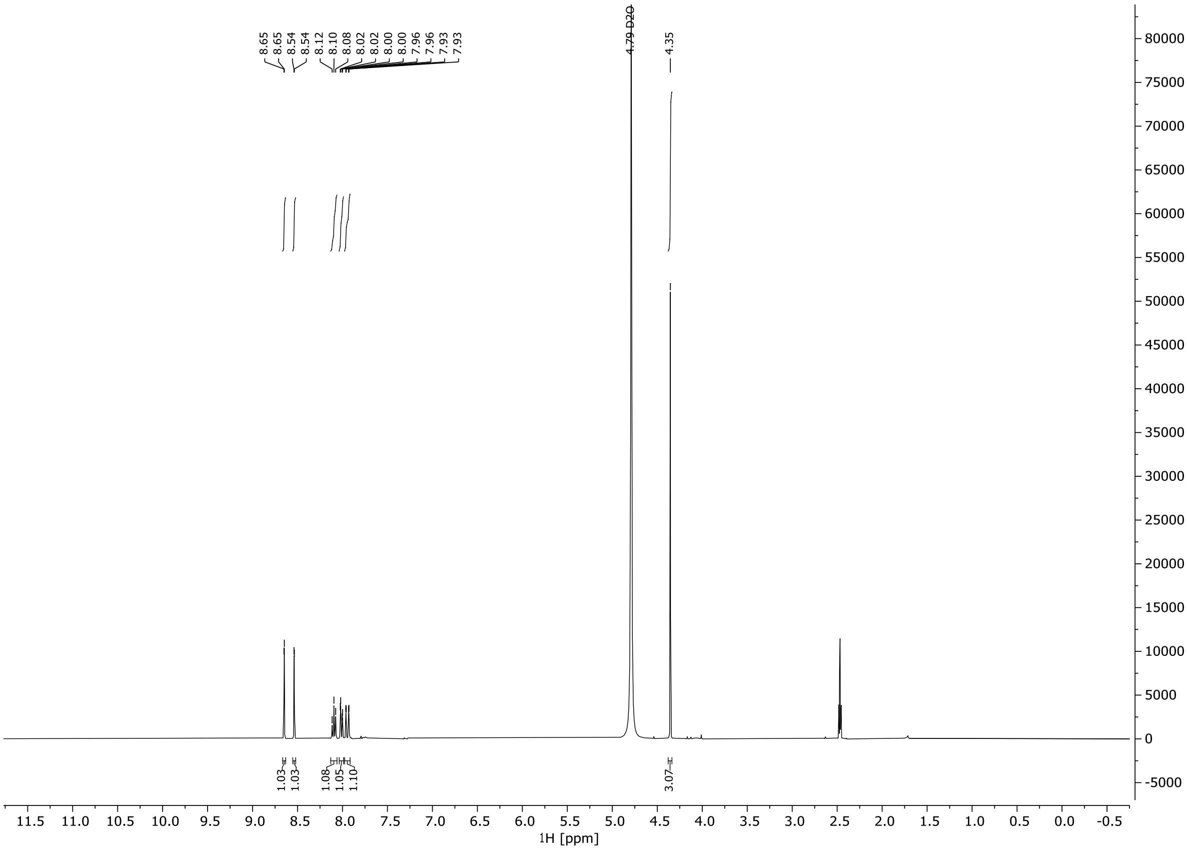

^{19}F NMR of compound **8f**

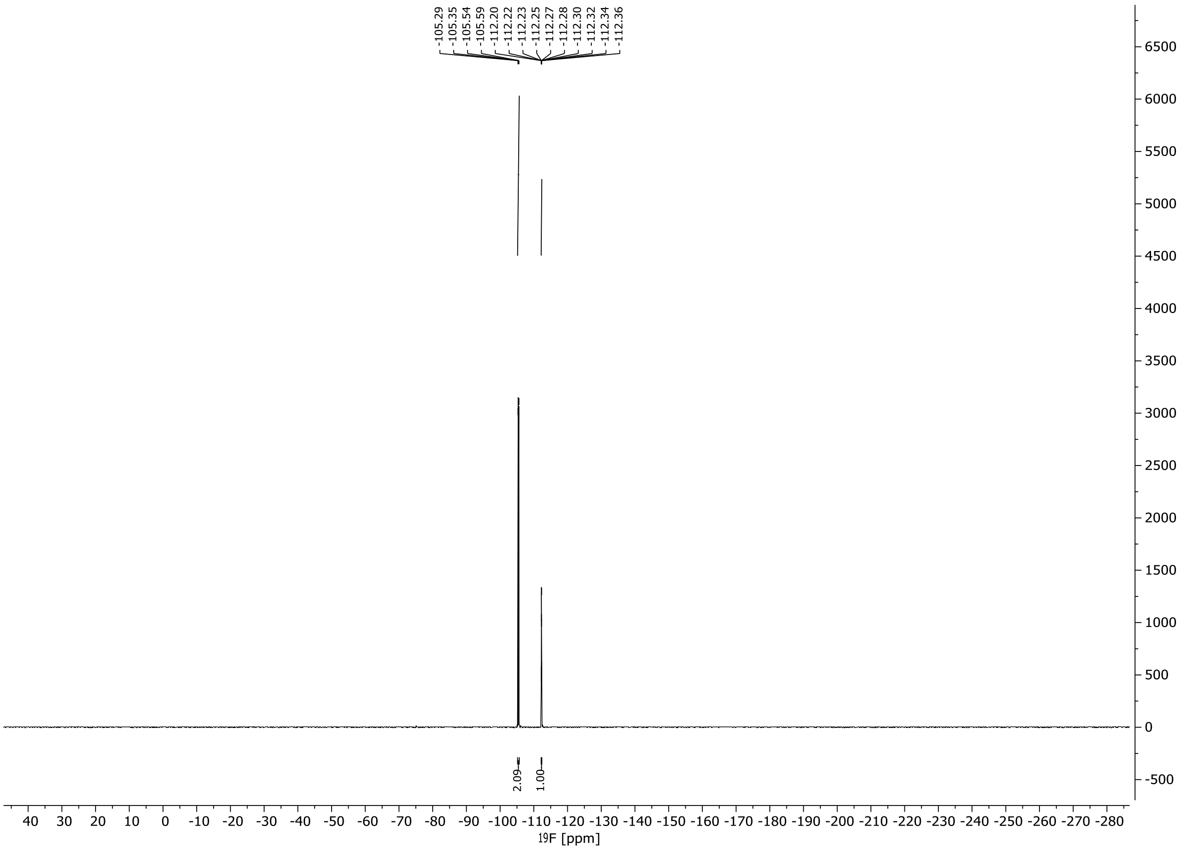

^{31}P NMR of compound **8f**

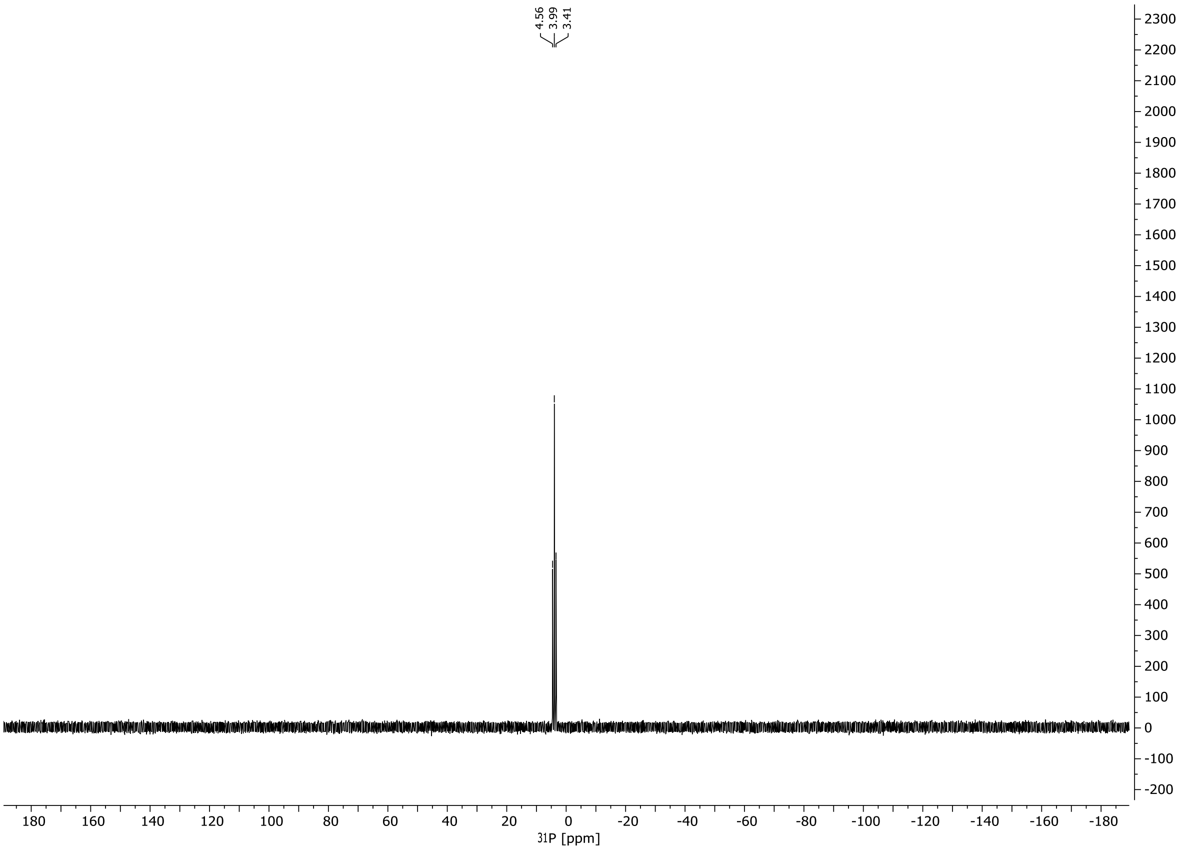

^{13}C NMR of compound **8f**

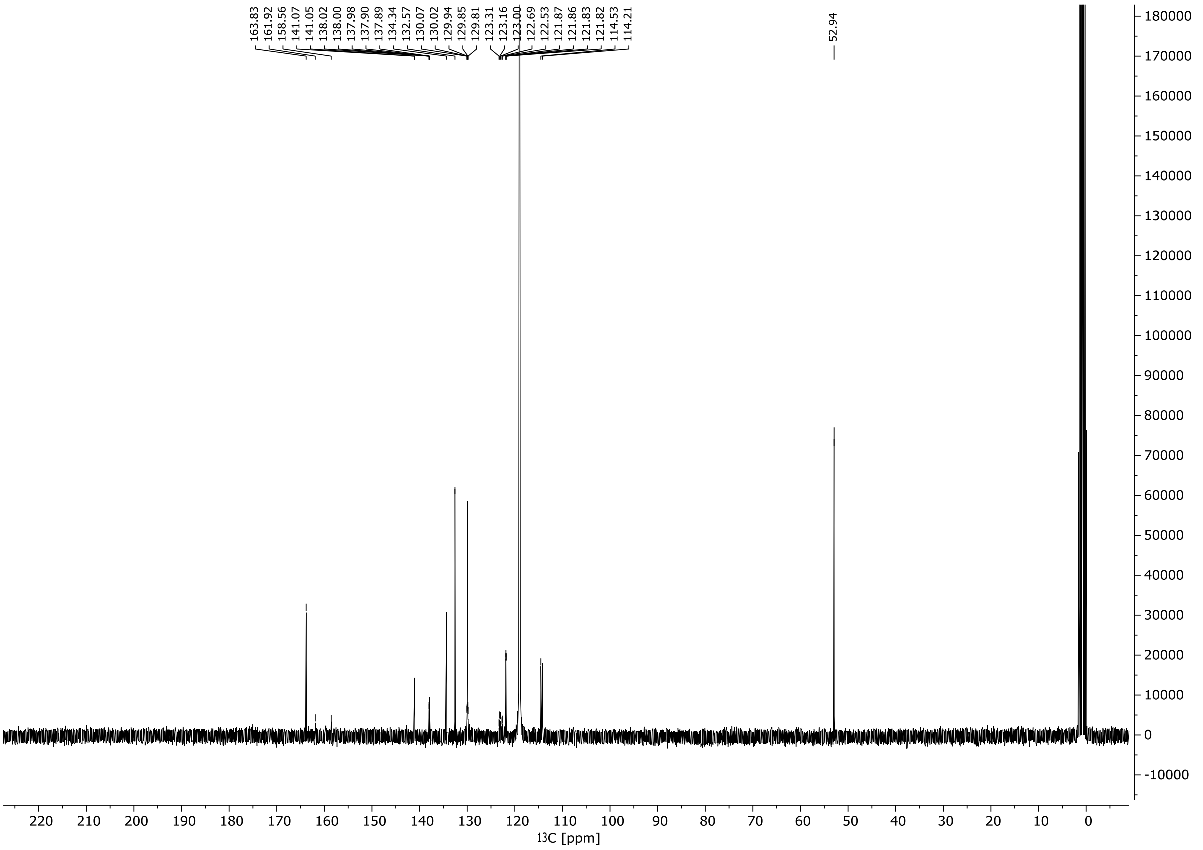

^{1}H NMR of compound **8g**

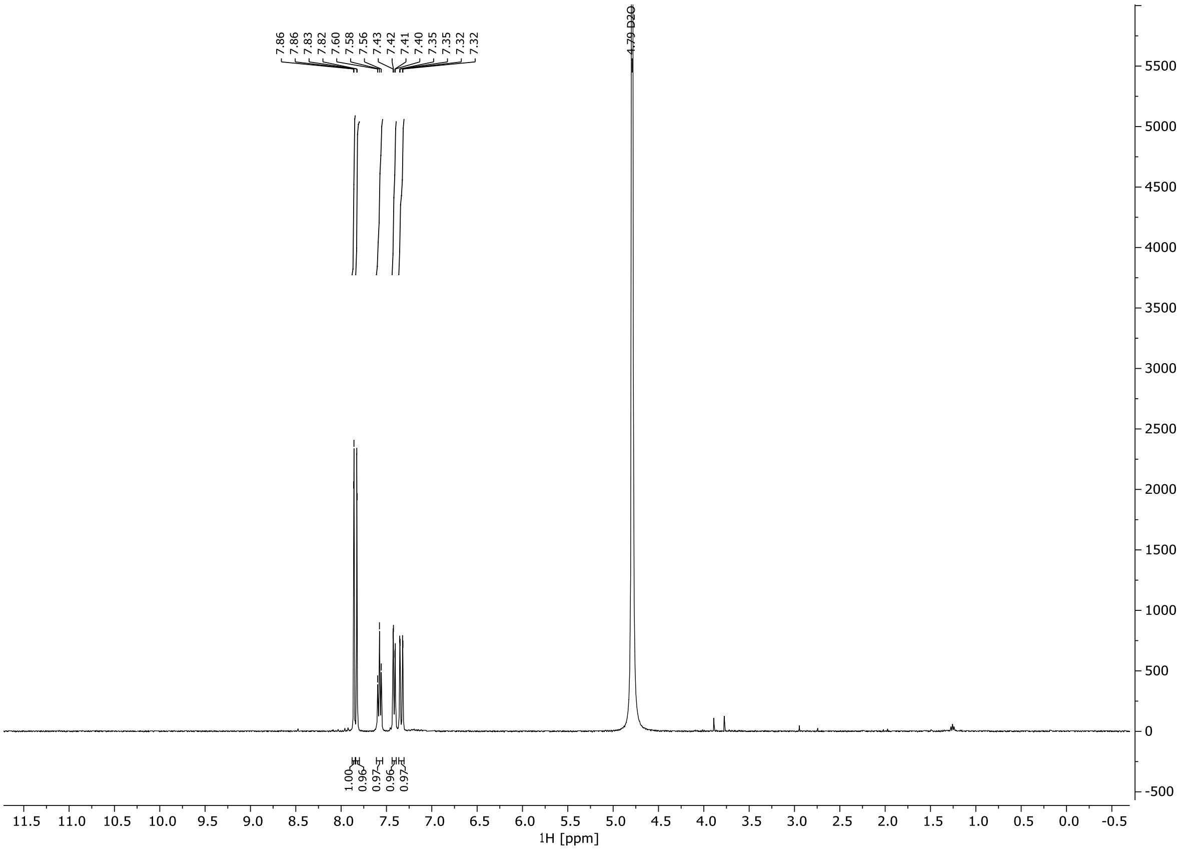

^{19}F NMR of compound **8g**

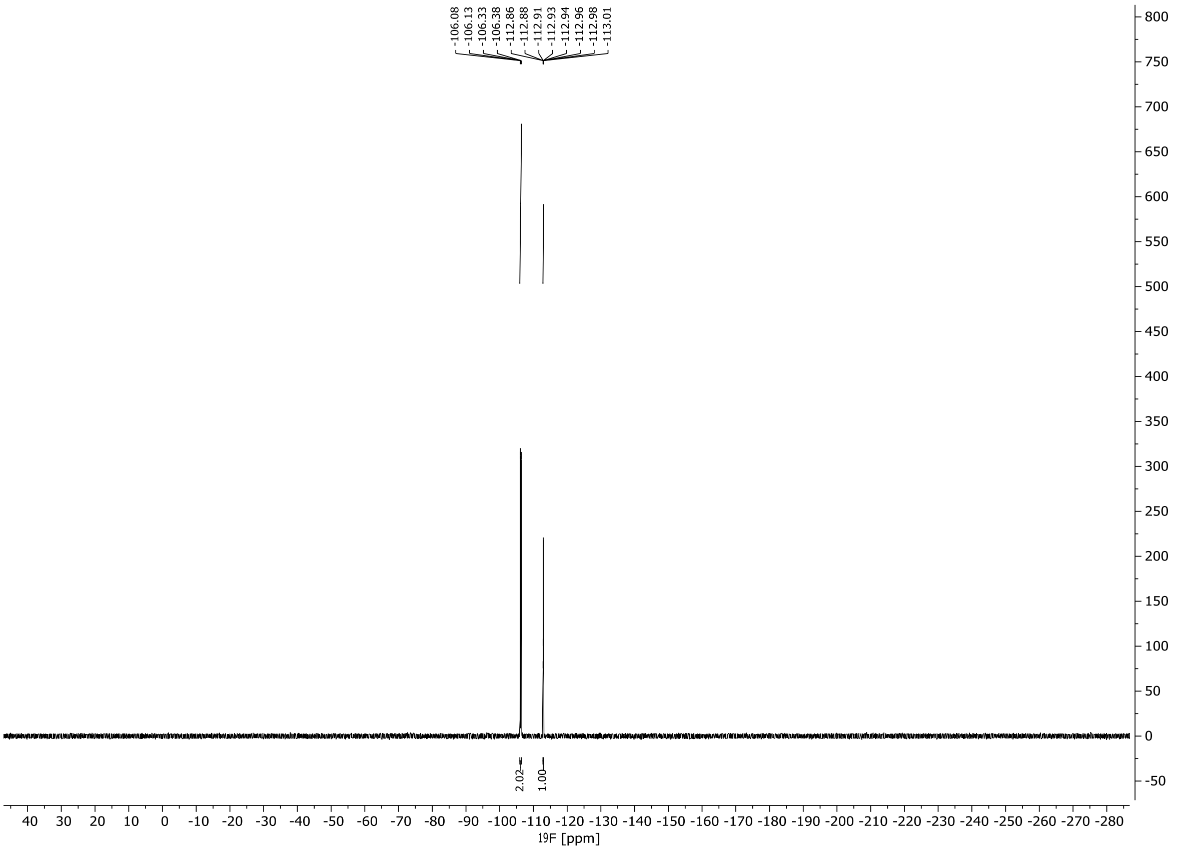

^{31}P NMR of compound **8g**

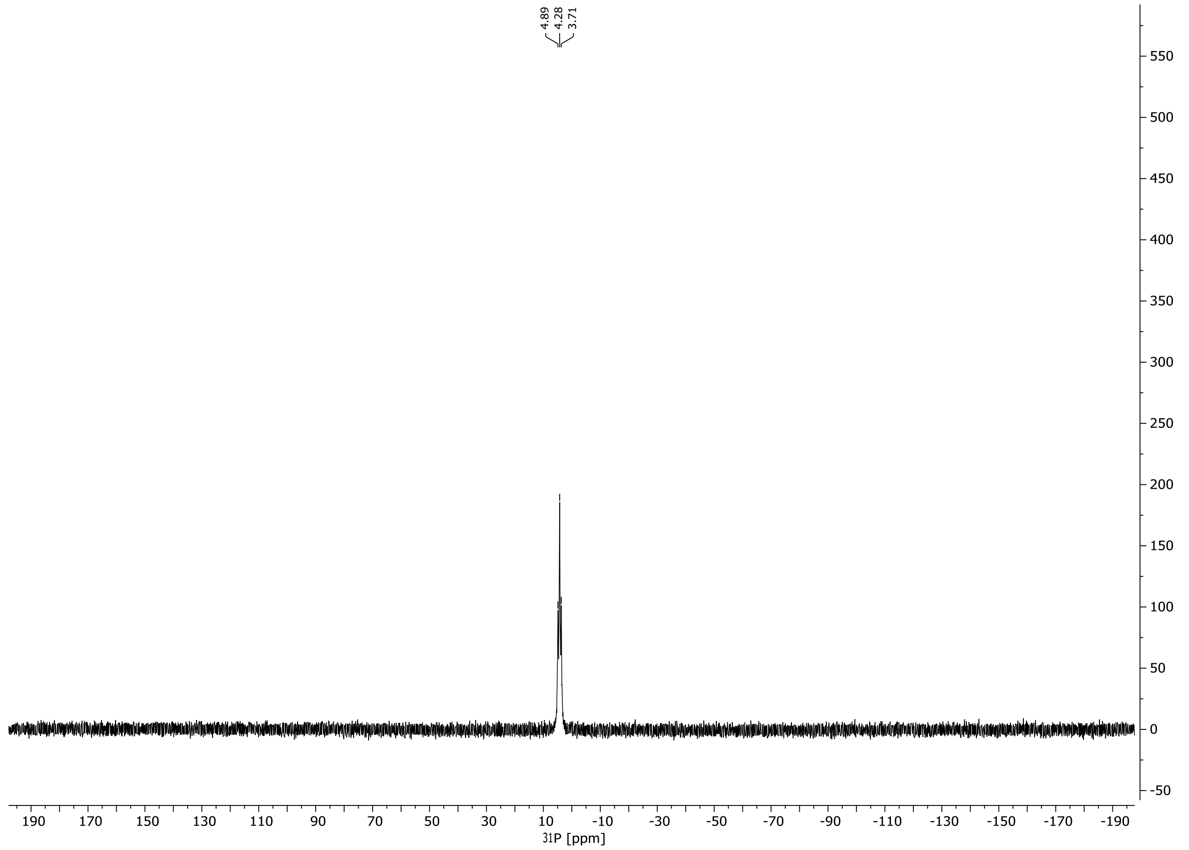

^{13}C NMR of compound **8g**

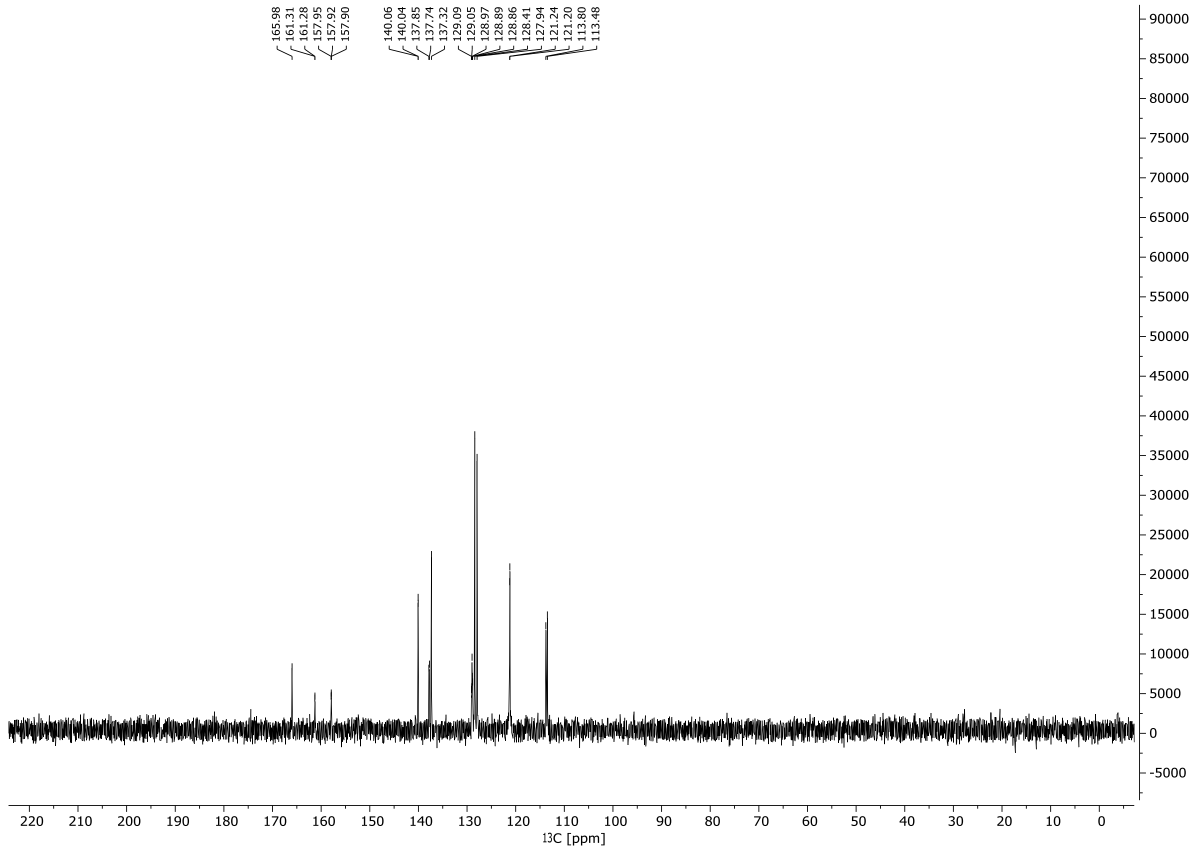

^{1}H NMR of compound **8h**

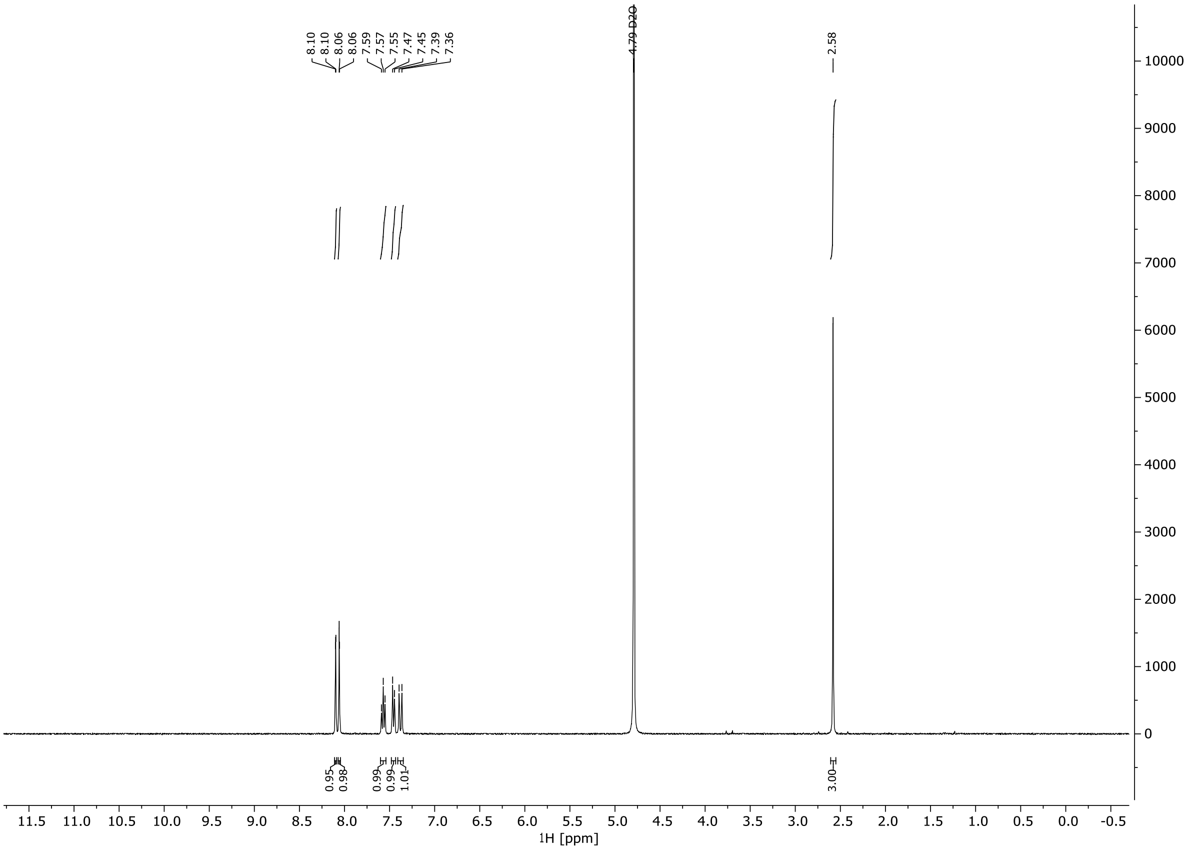

^{19}F NMR of compound **8h**

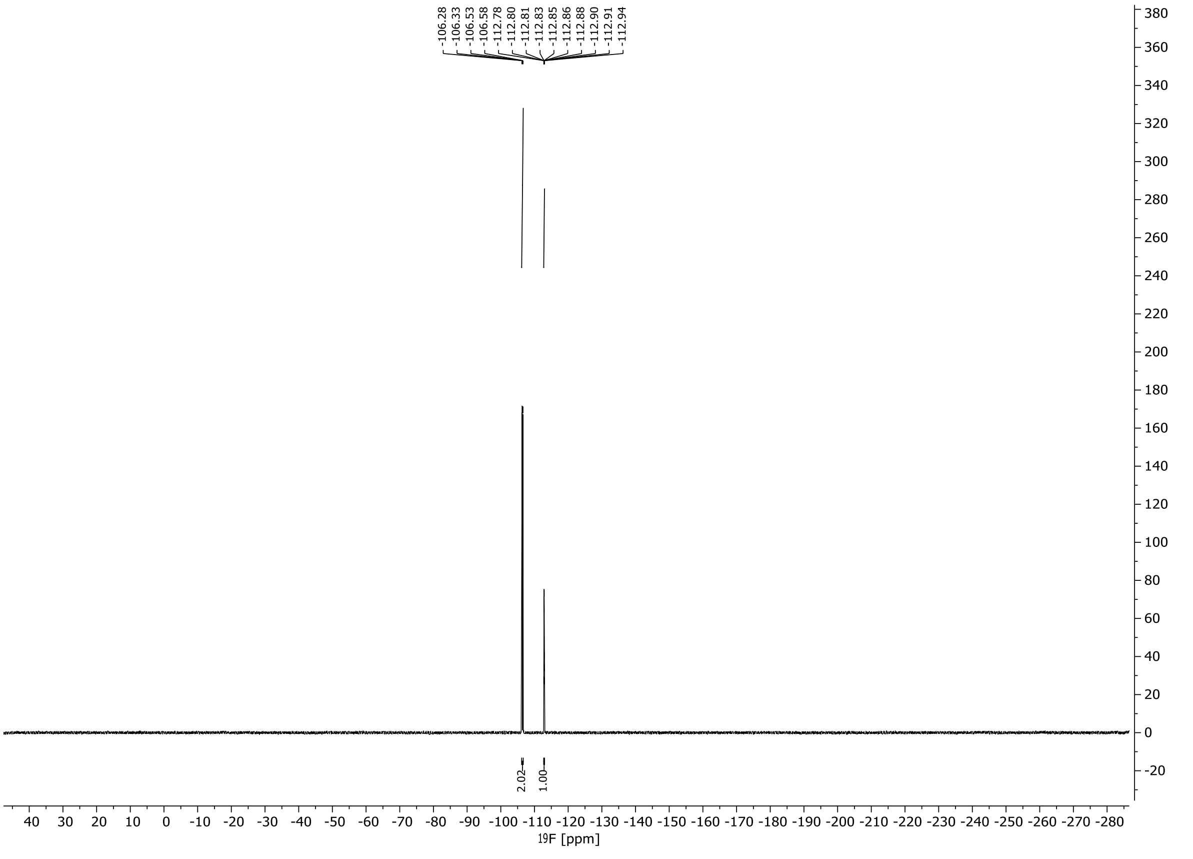

^{31}P NMR of compound **8h**

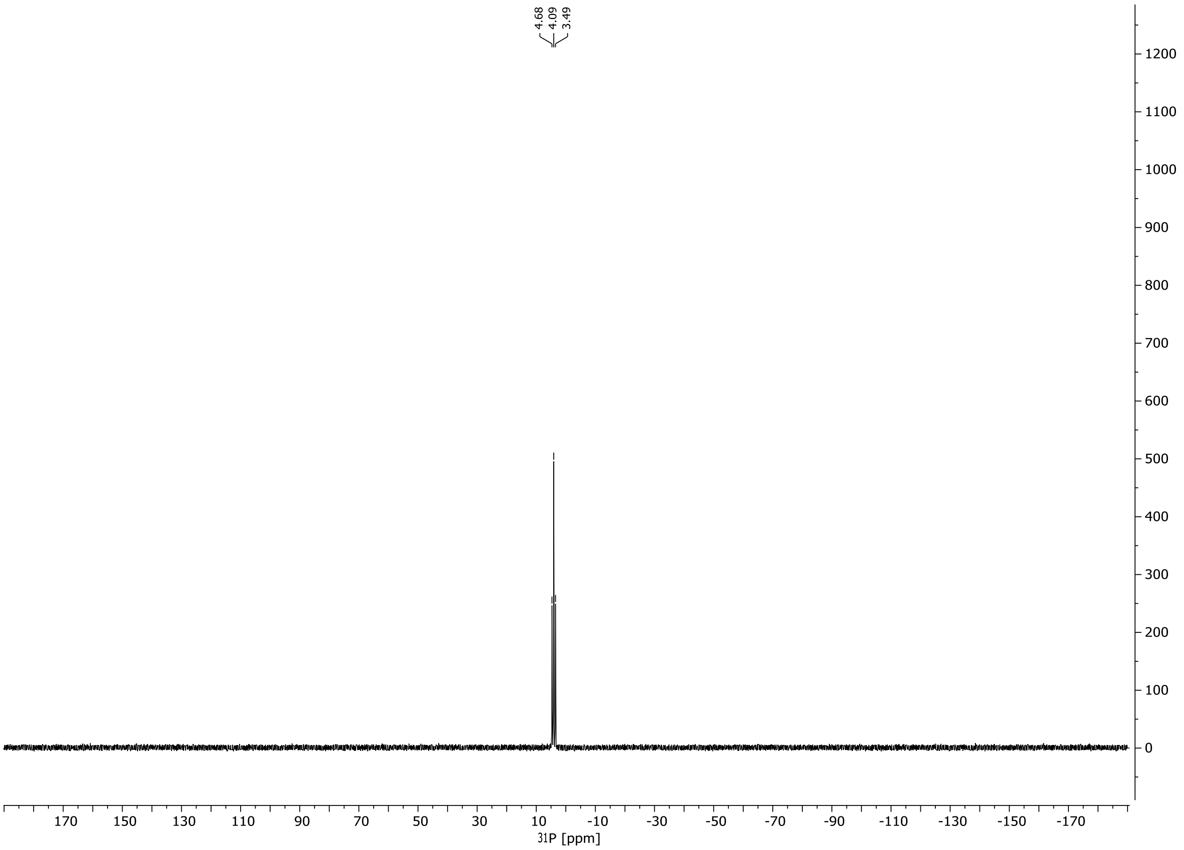

^{13}C NMR of compound **8h**

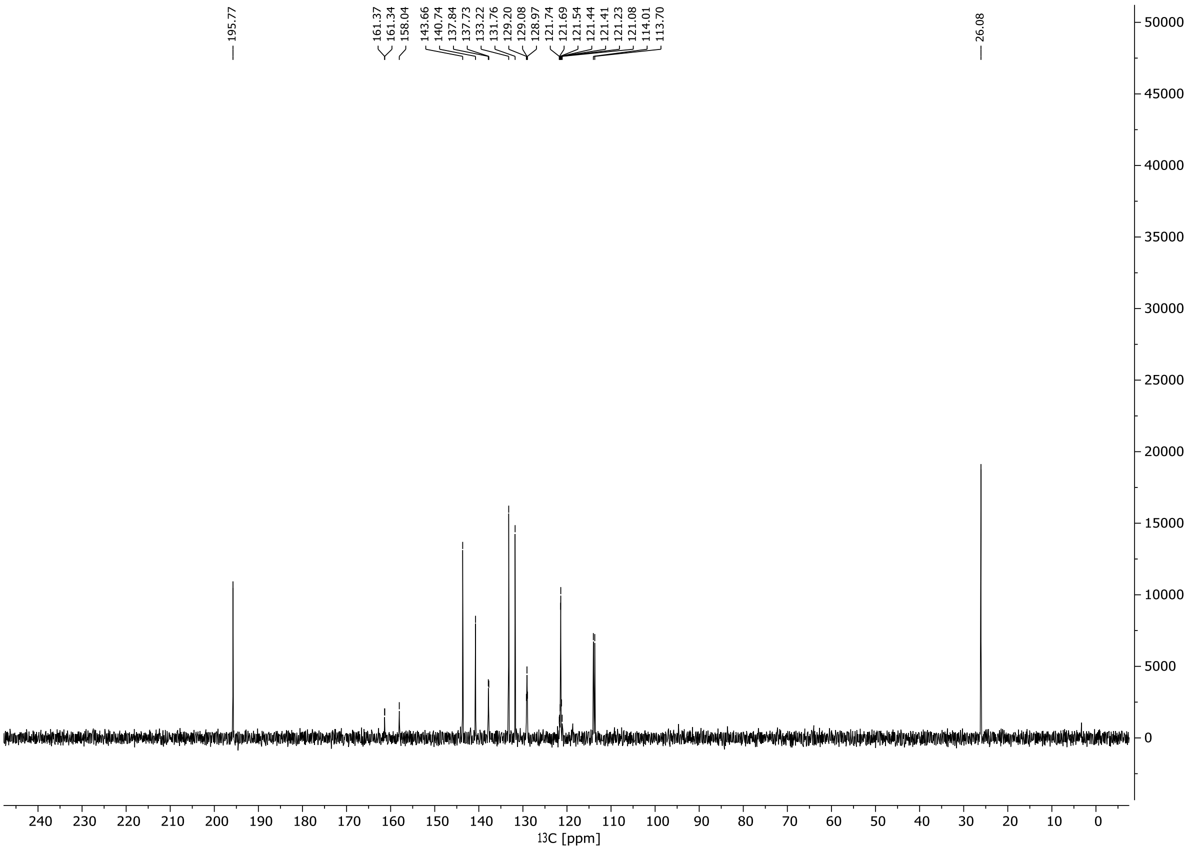

^1H NMR of compound **8i**

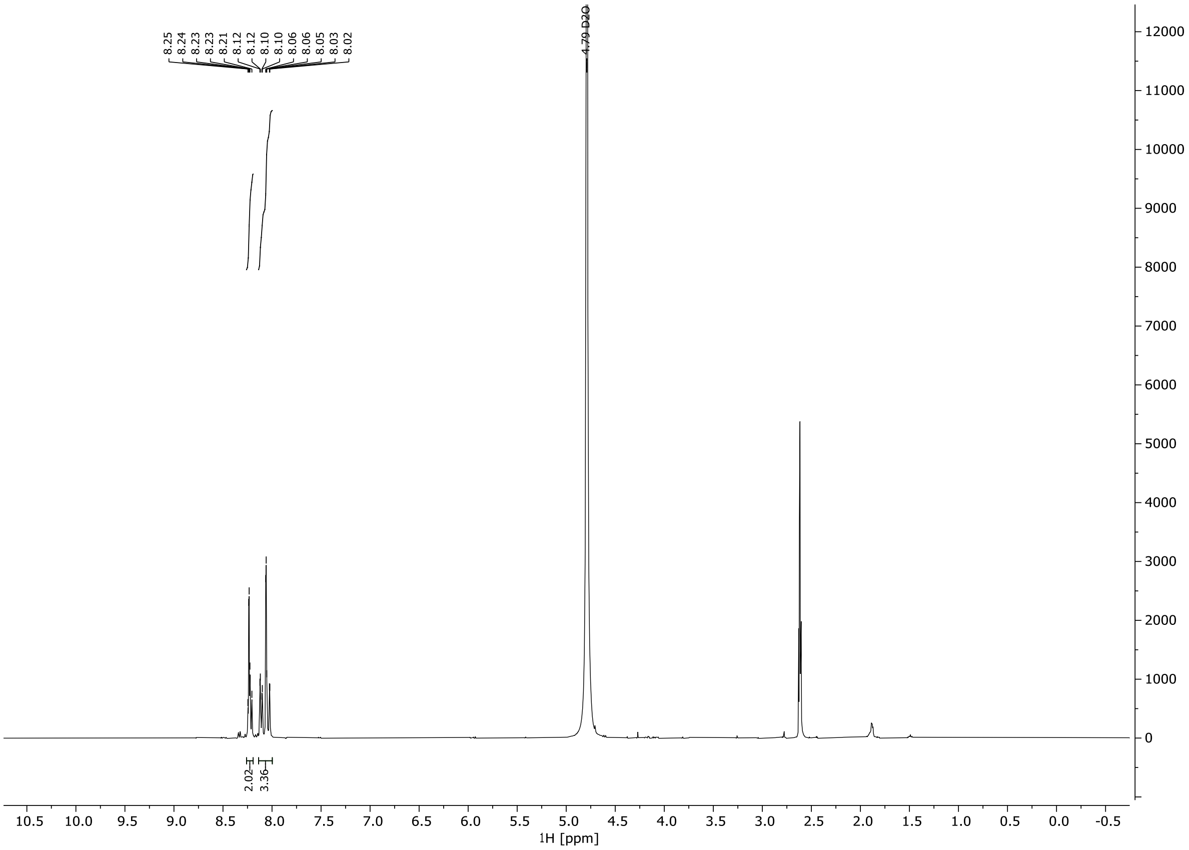

^{19}F NMR of compound **8i**

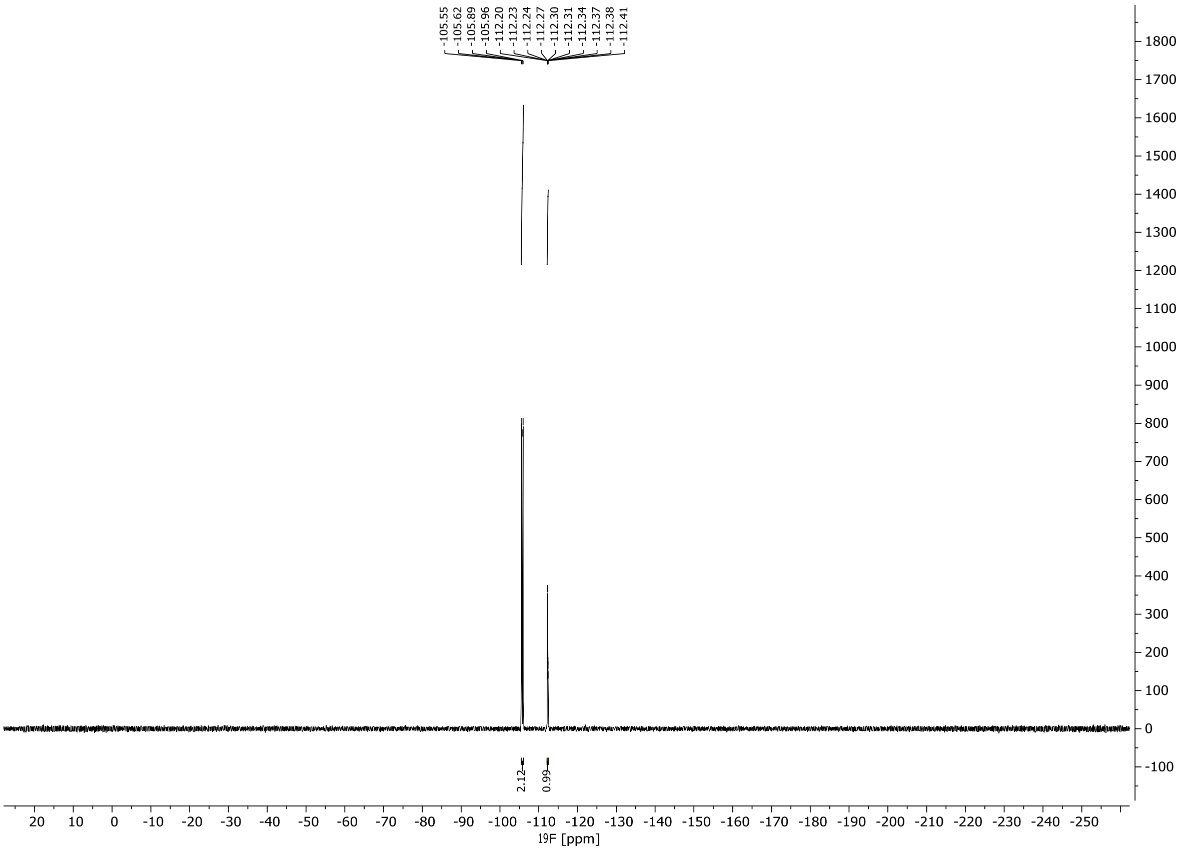

^{31}P NMR of compound **8i**

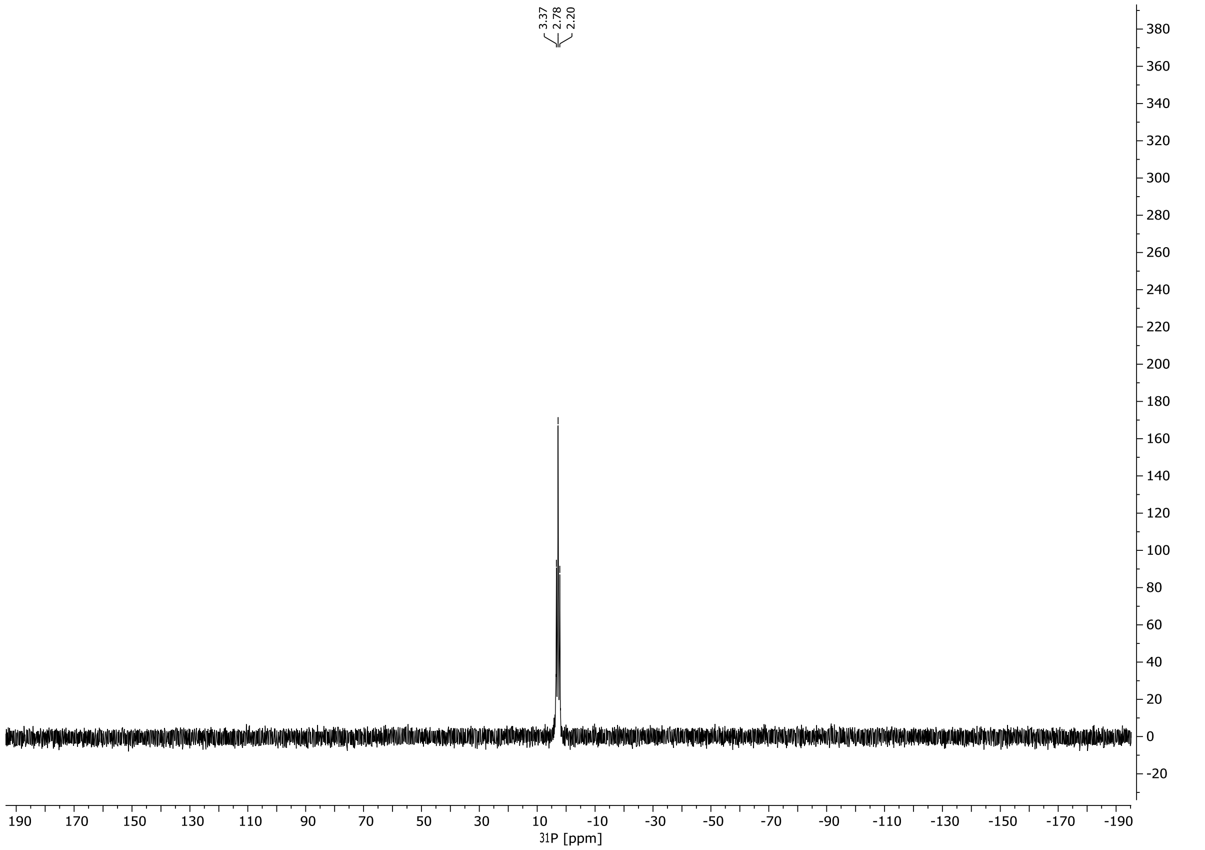

^{13}C NMR of compound **8i**

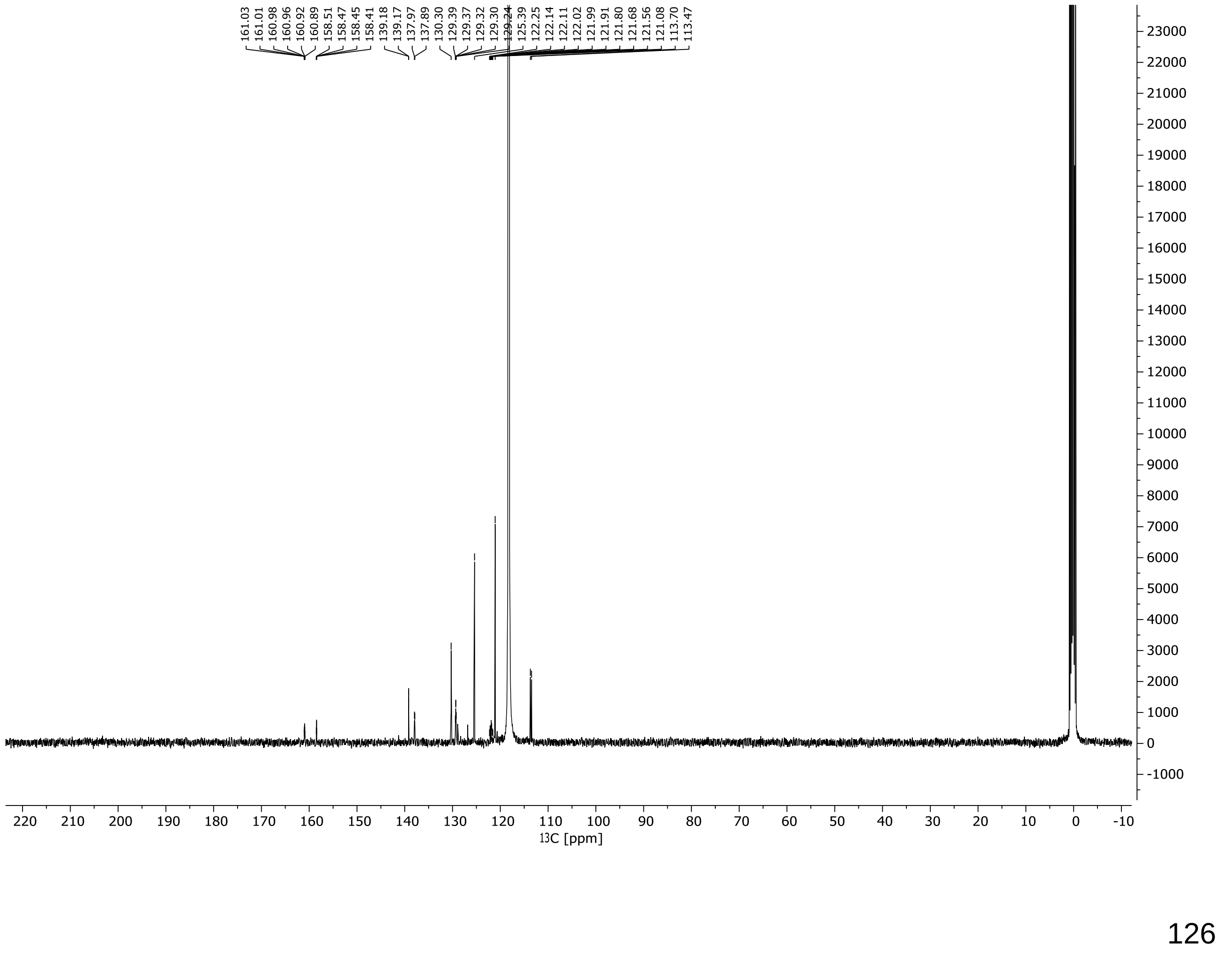

^{1}H NMR of compound **8j**

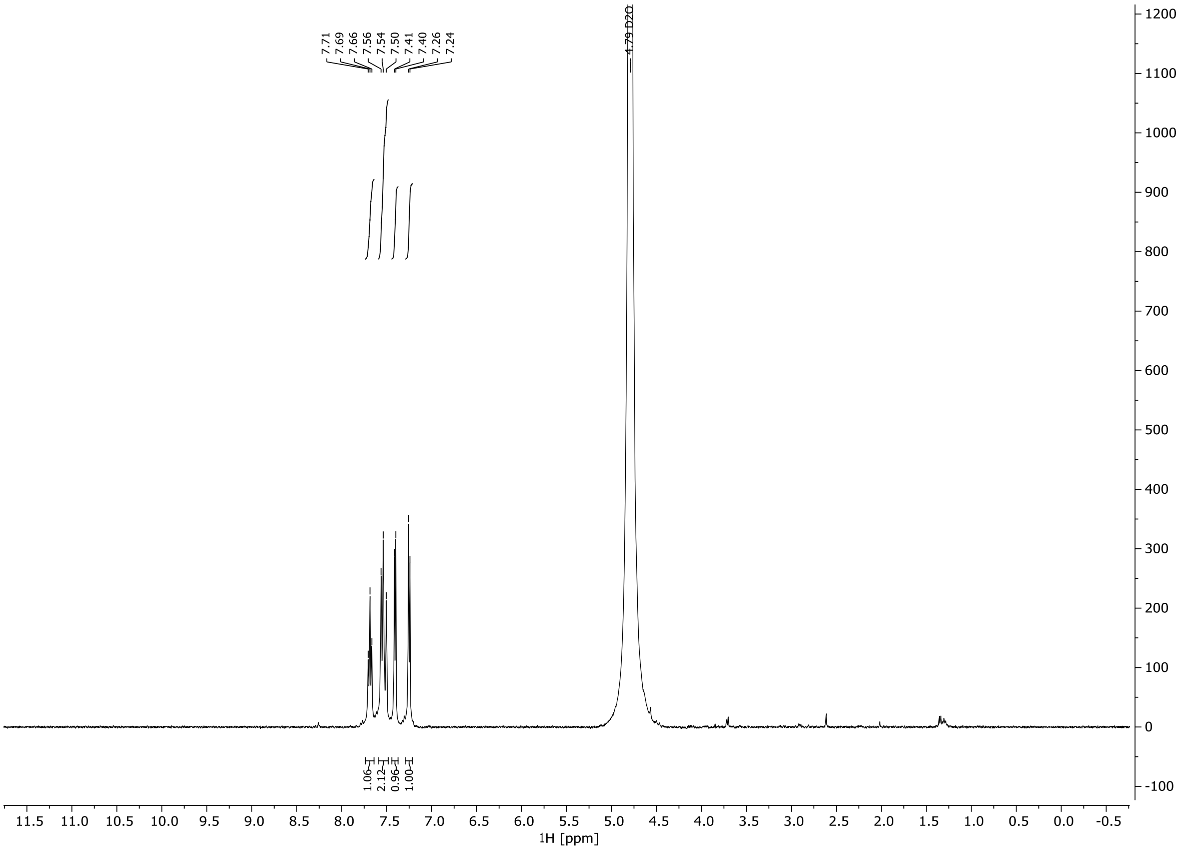

^{19}F NMR of compound **8j**

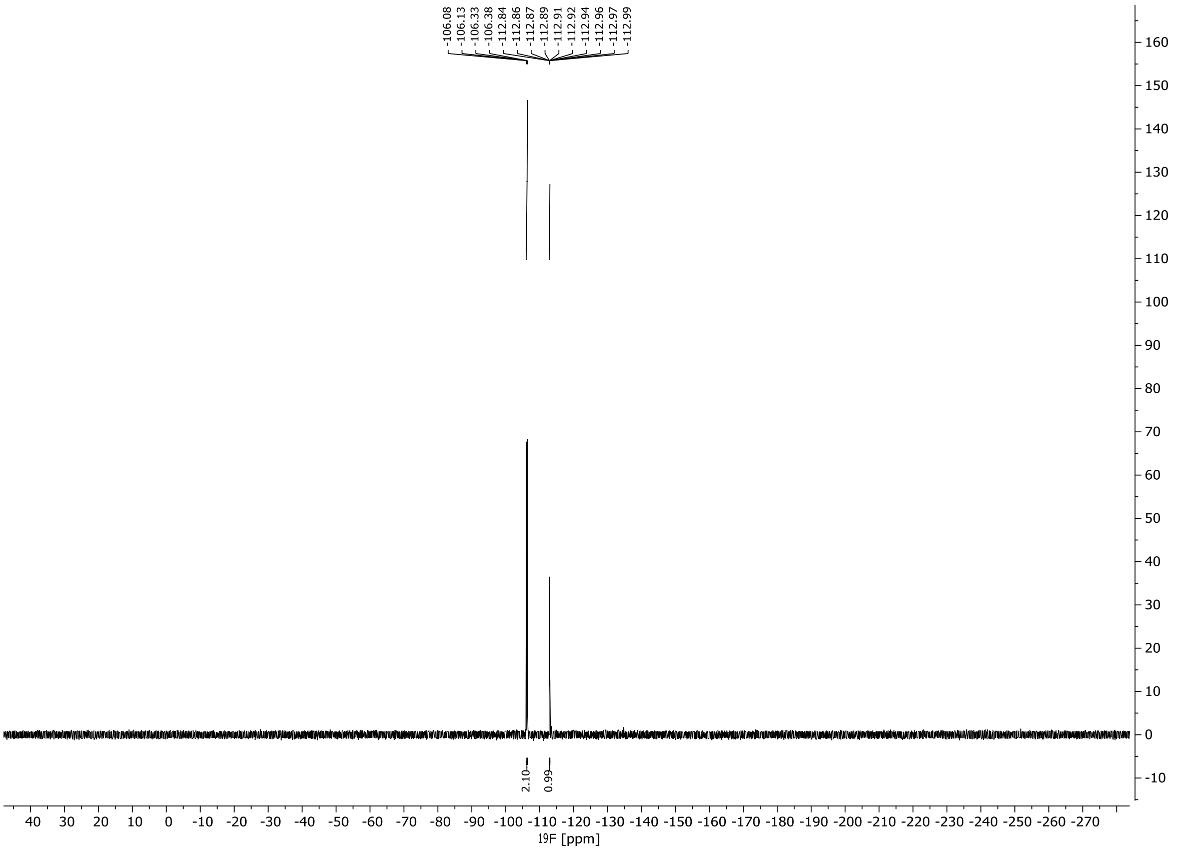

^{31}P NMR of compound **8j**

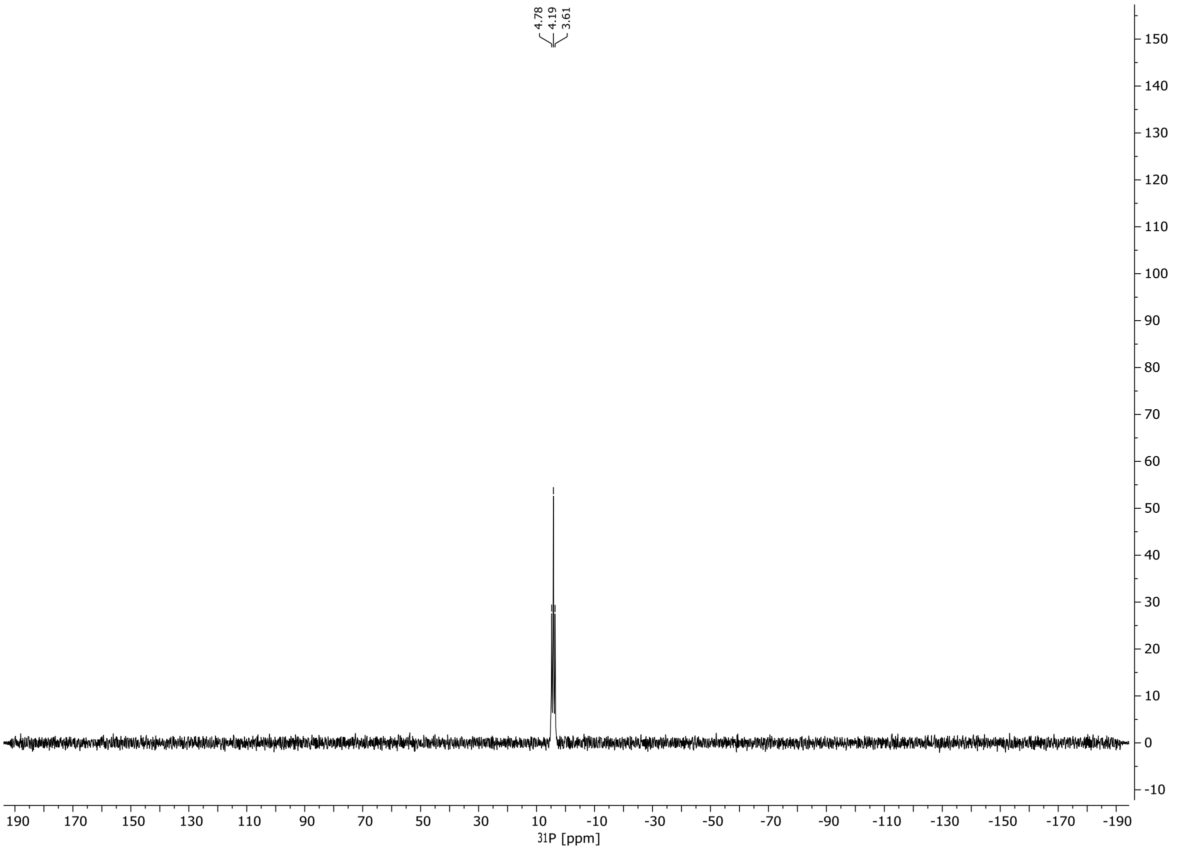

^{13}C NMR of compound **8j**

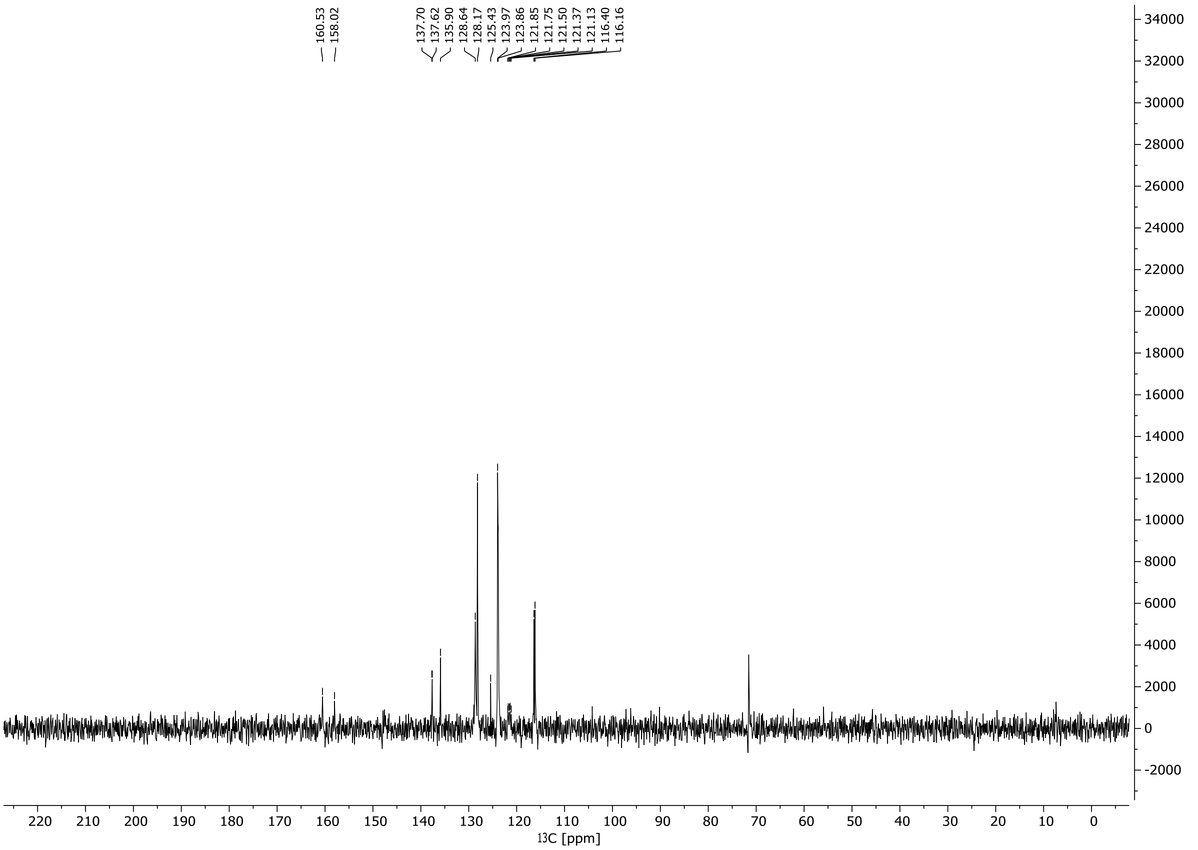

^{1}H NMR of compound **8k**

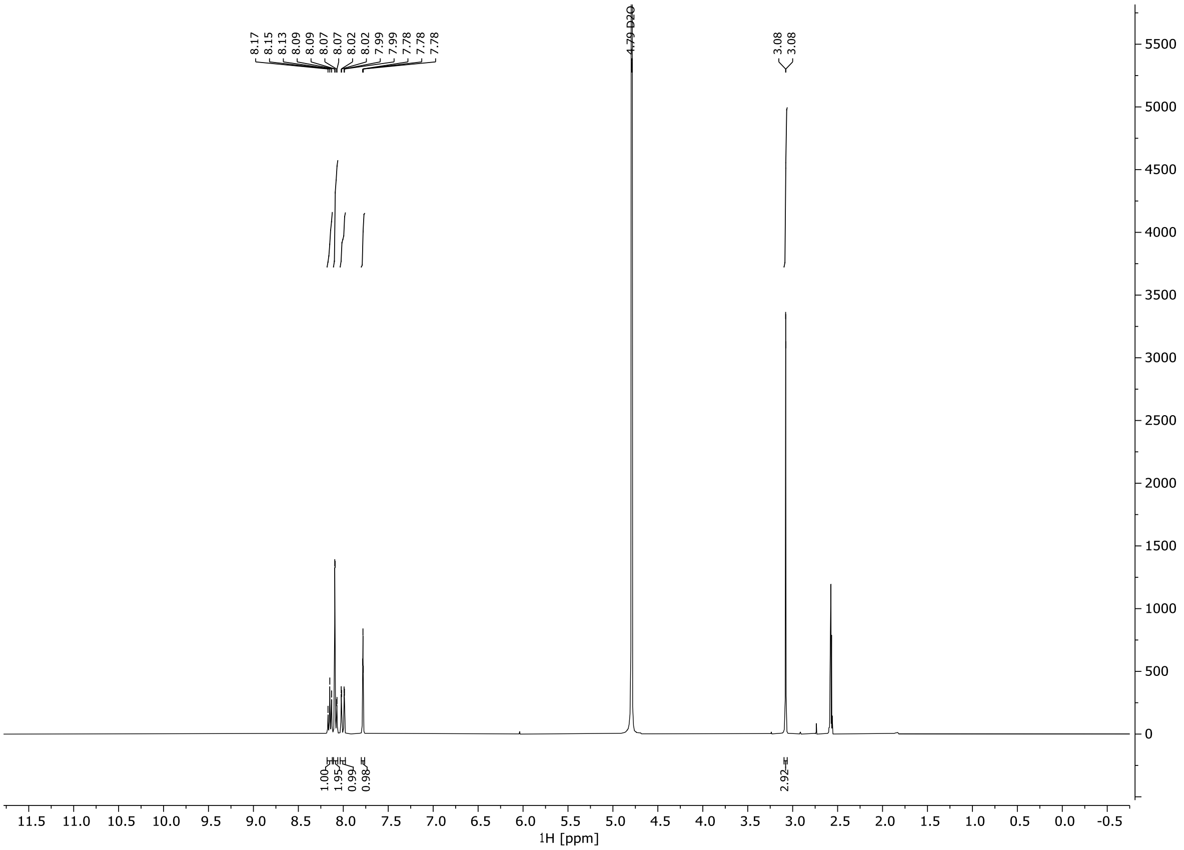

^{19}F NMR of compound **8k**

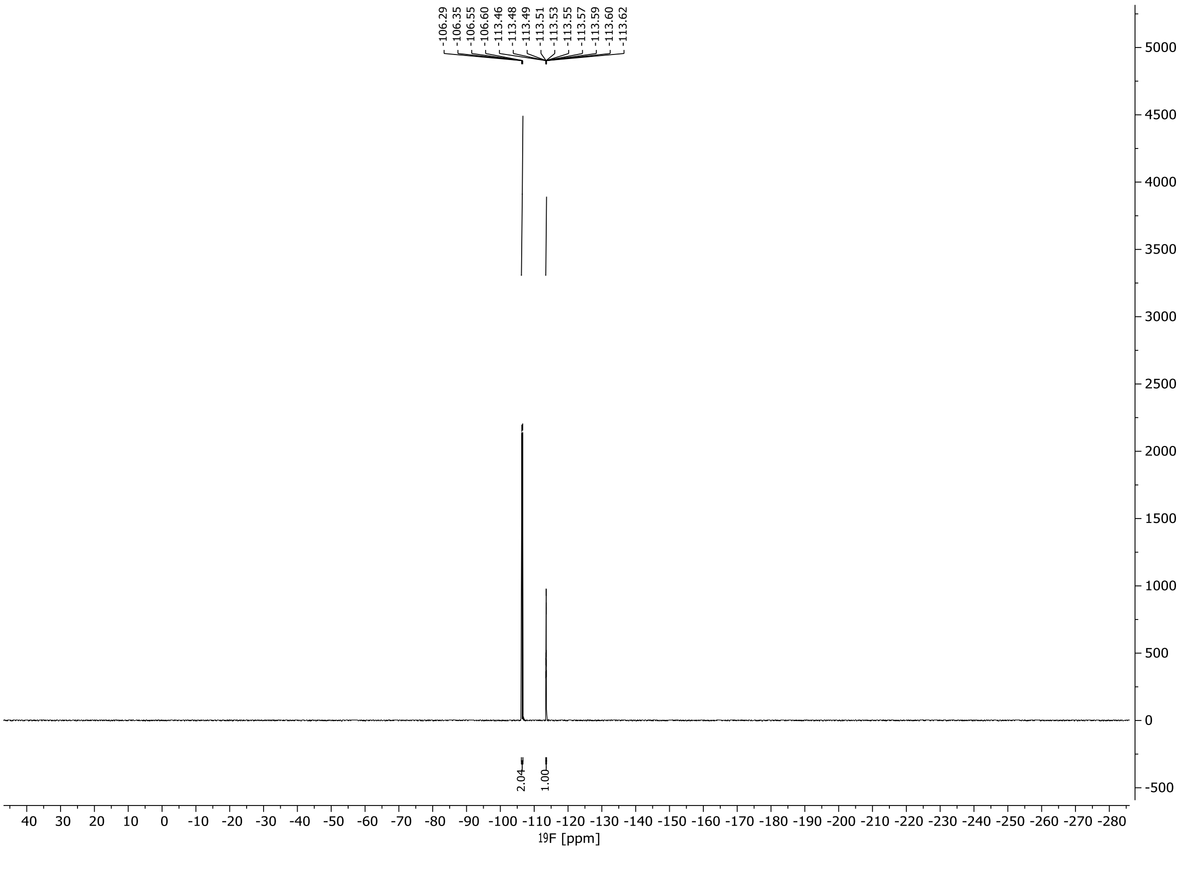

^{31}P NMR of compound **8k**

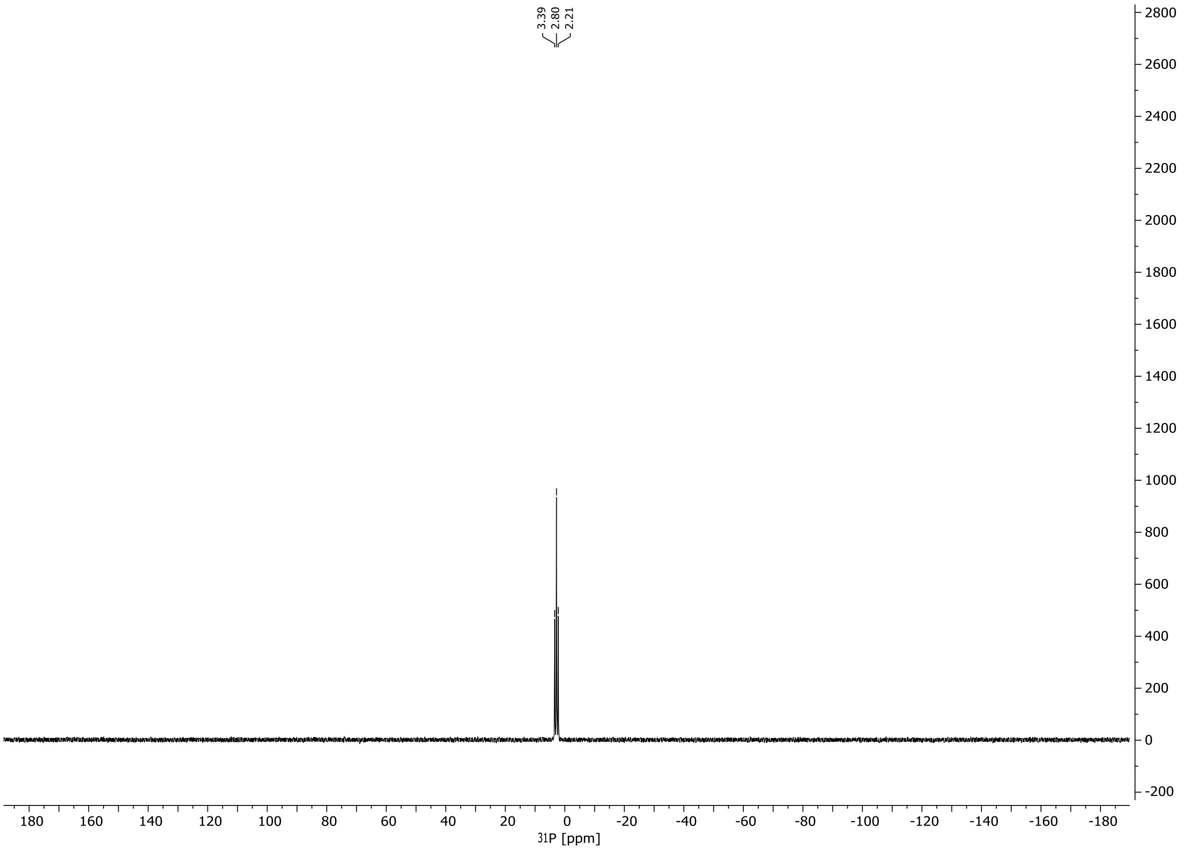

^{13}C NMR of compound **8k**

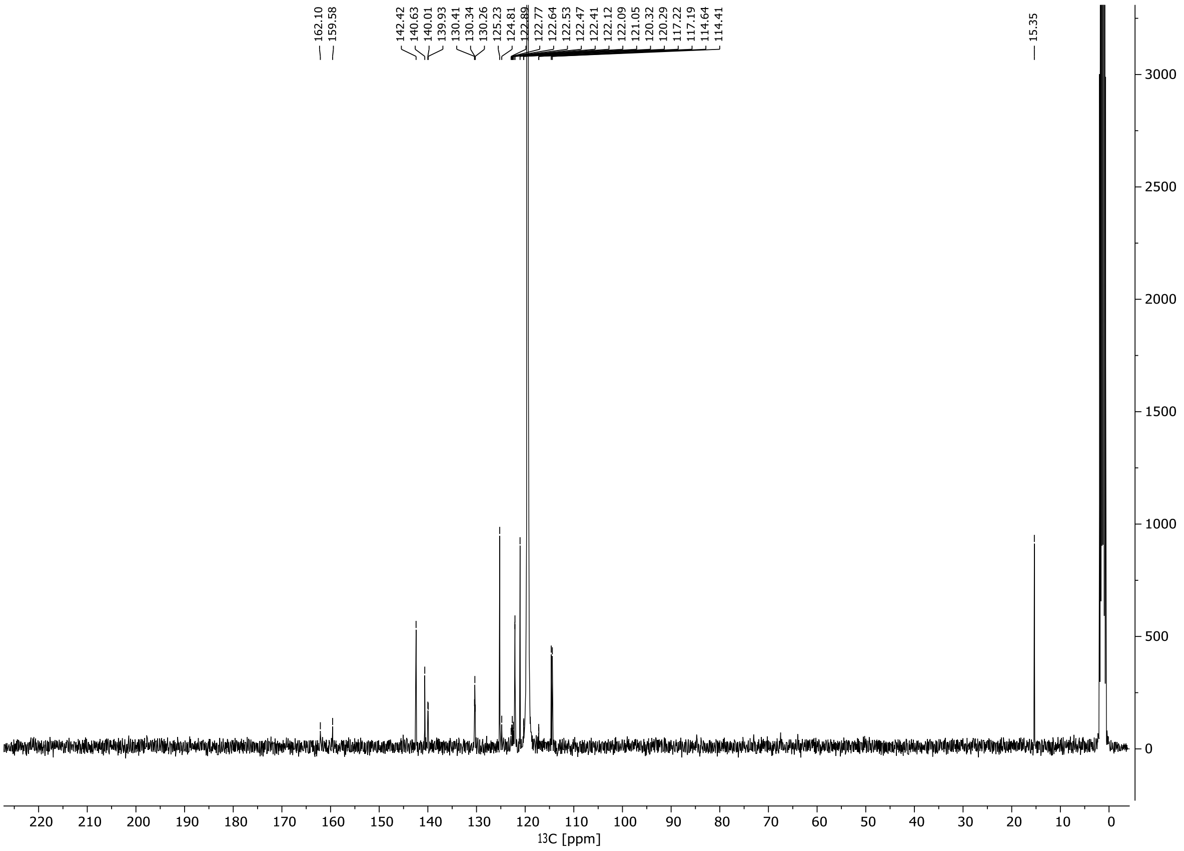

^{1}H NMR of compound **8l**

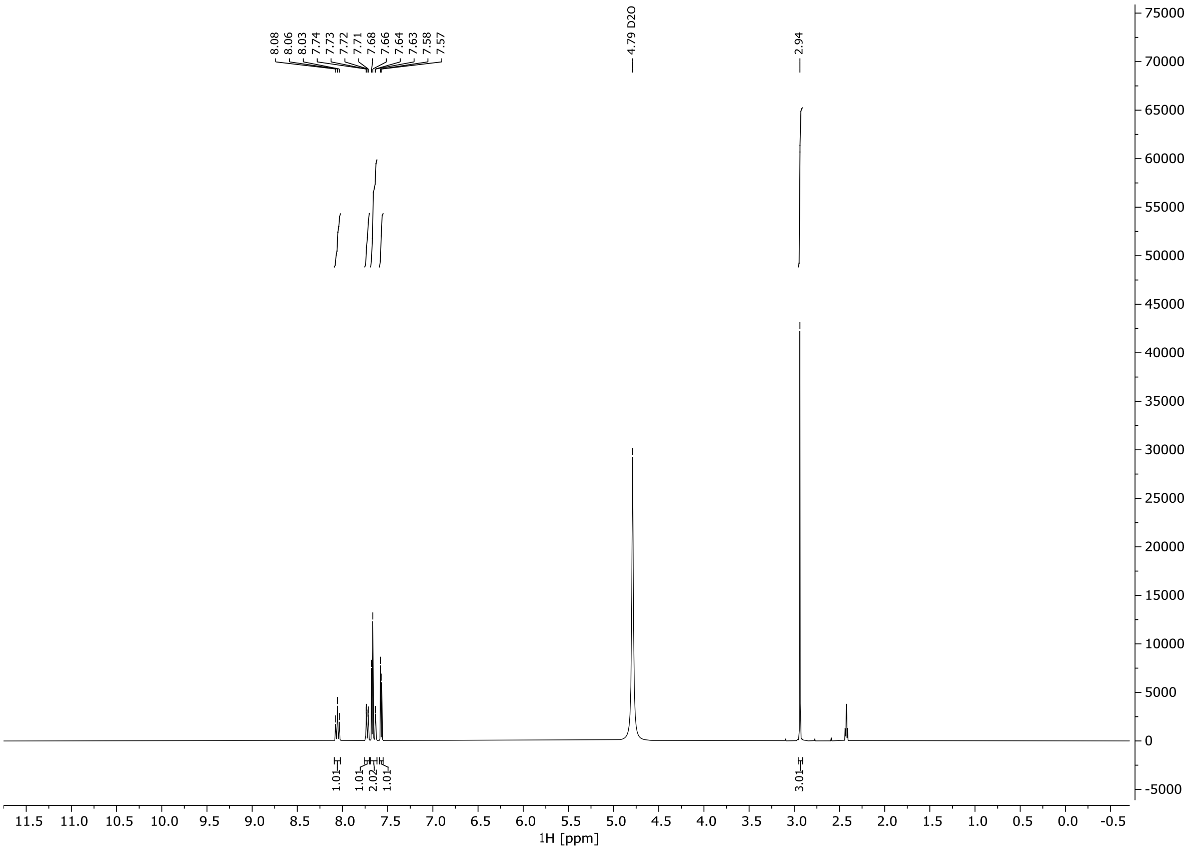

^{19}F NMR of compound **8l**

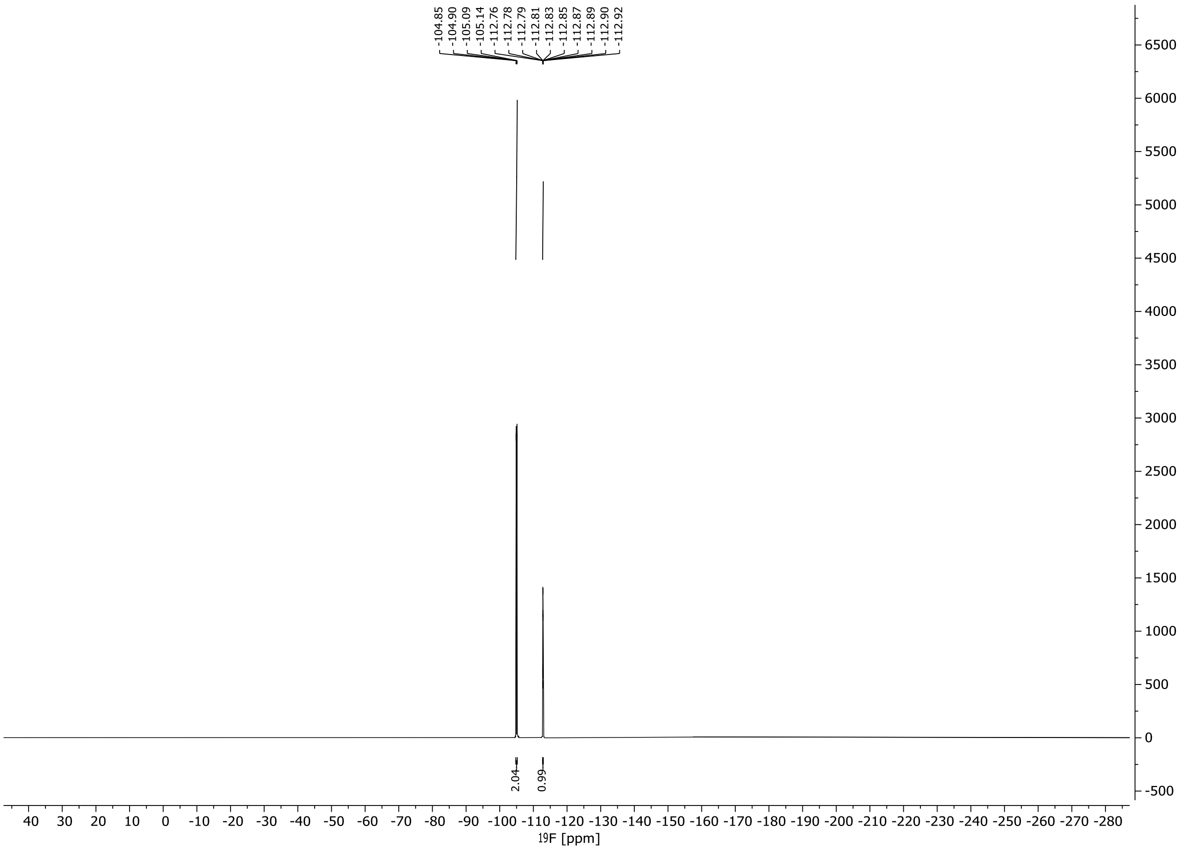

^{31}P NMR of compound **8l**

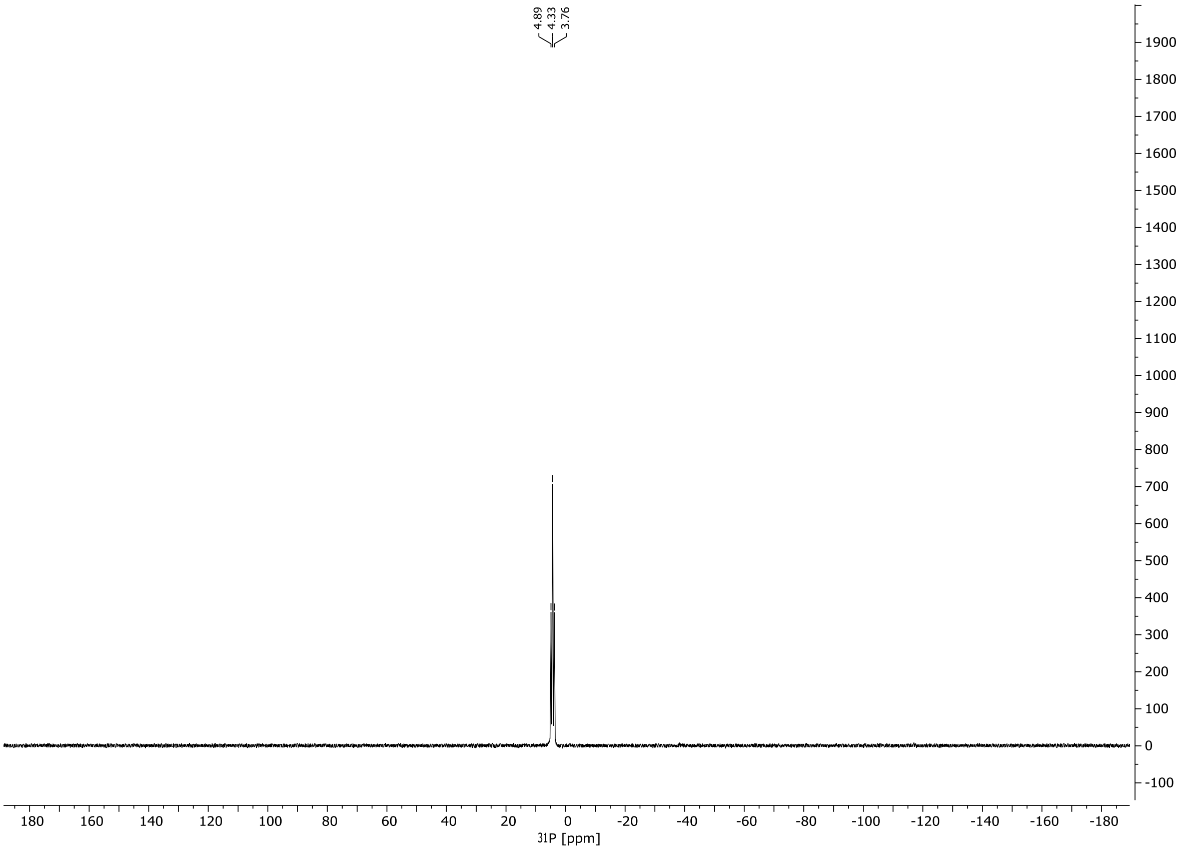

^{13}C NMR of compound **8l**

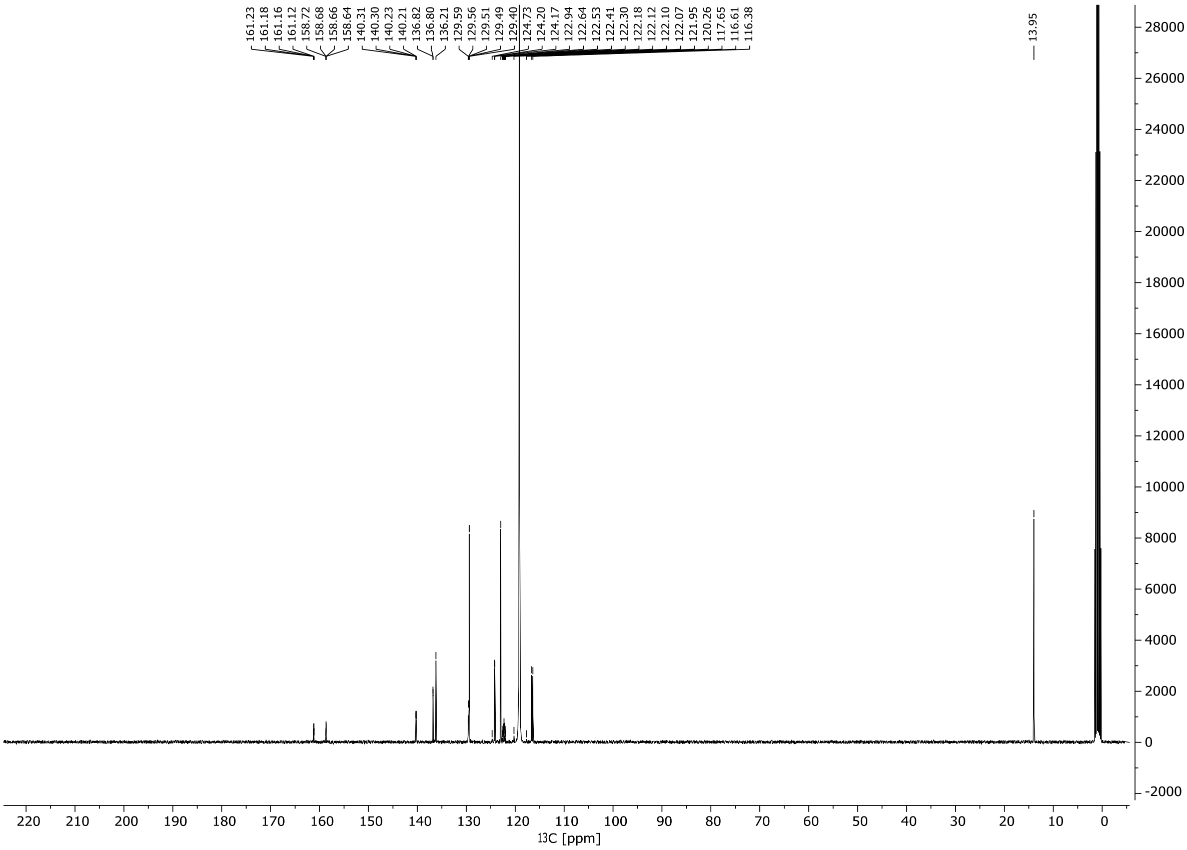

^{1}H NMR of compound **8m**

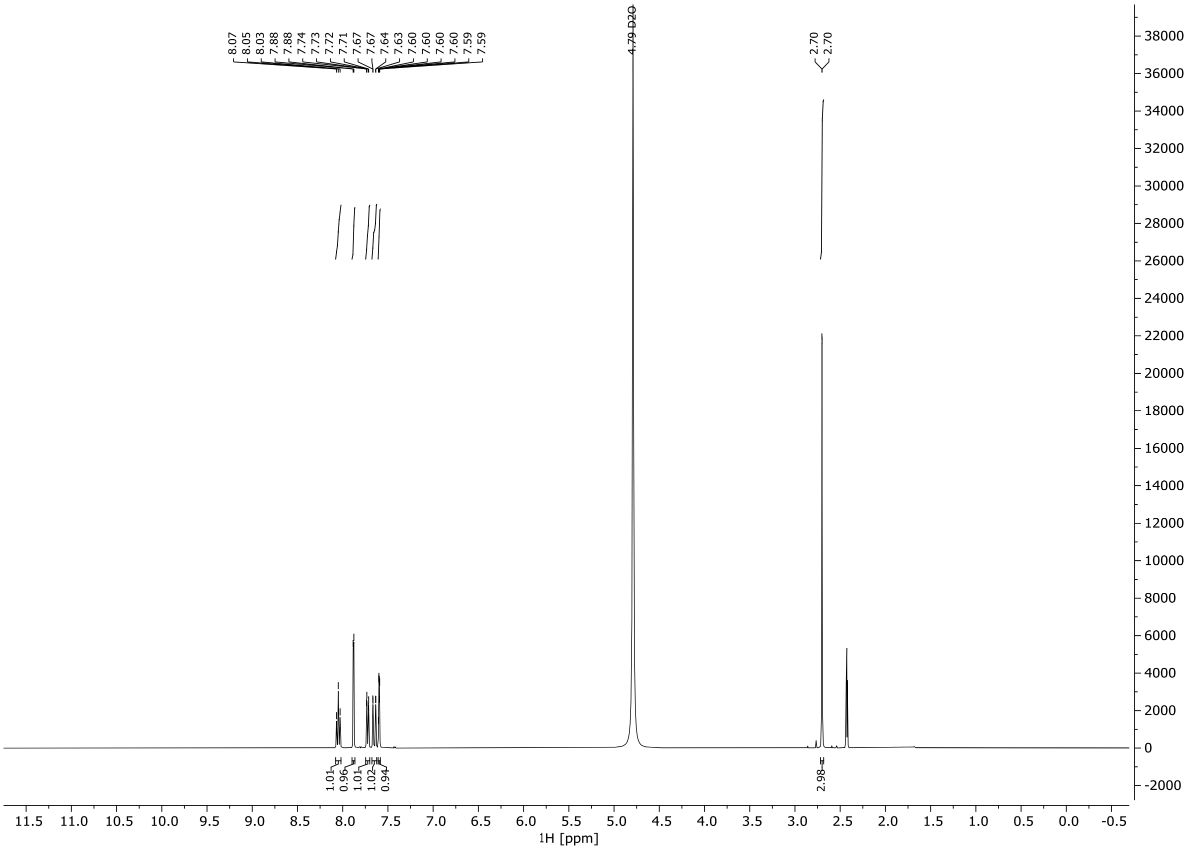

^{19}F NMR of compound **8m**

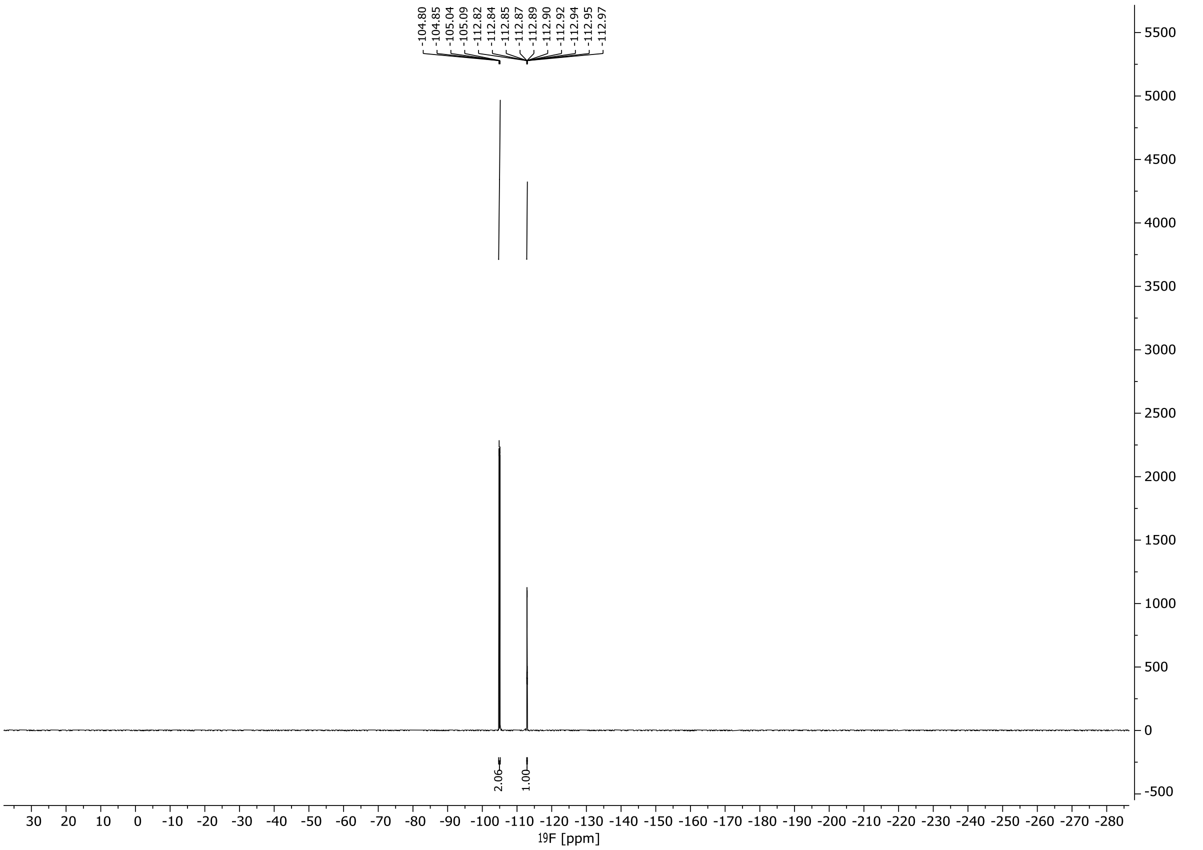

^{31}P NMR of compound **8m**

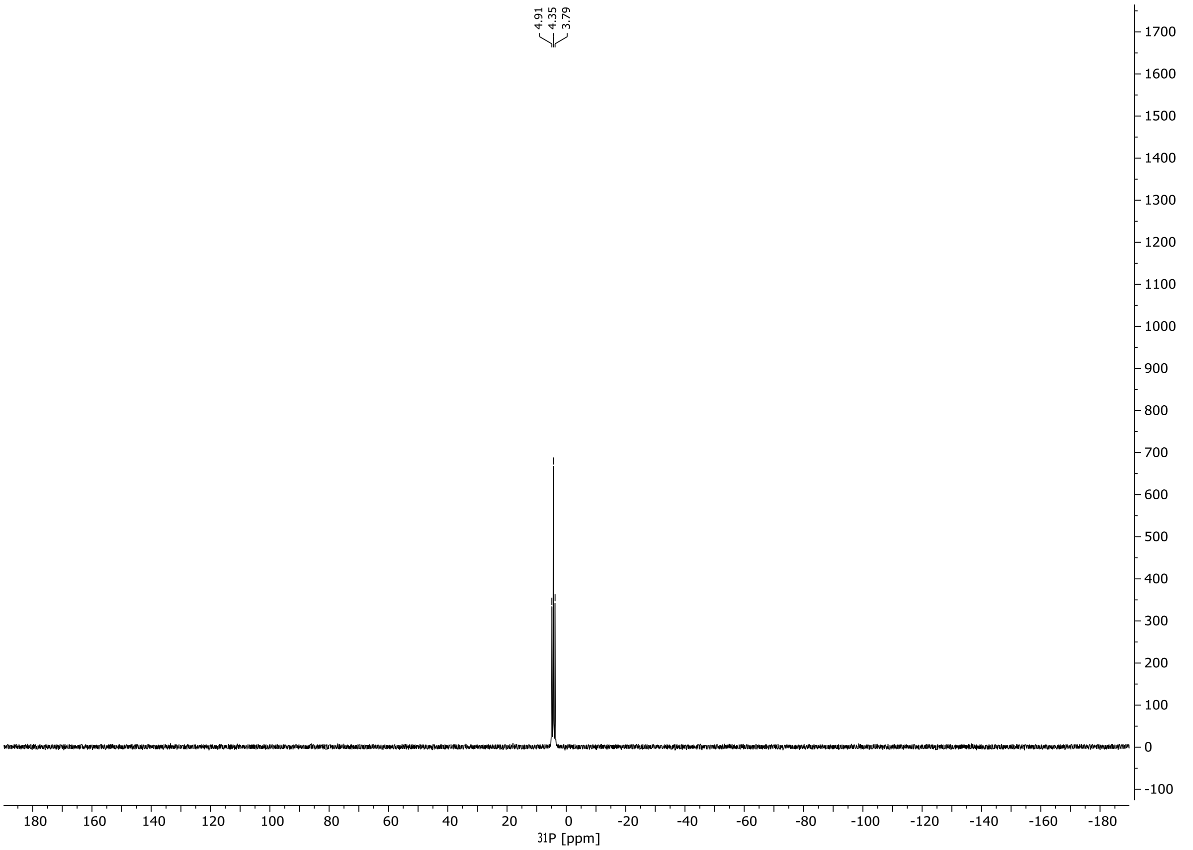

^{13}C NMR of compound **8m**

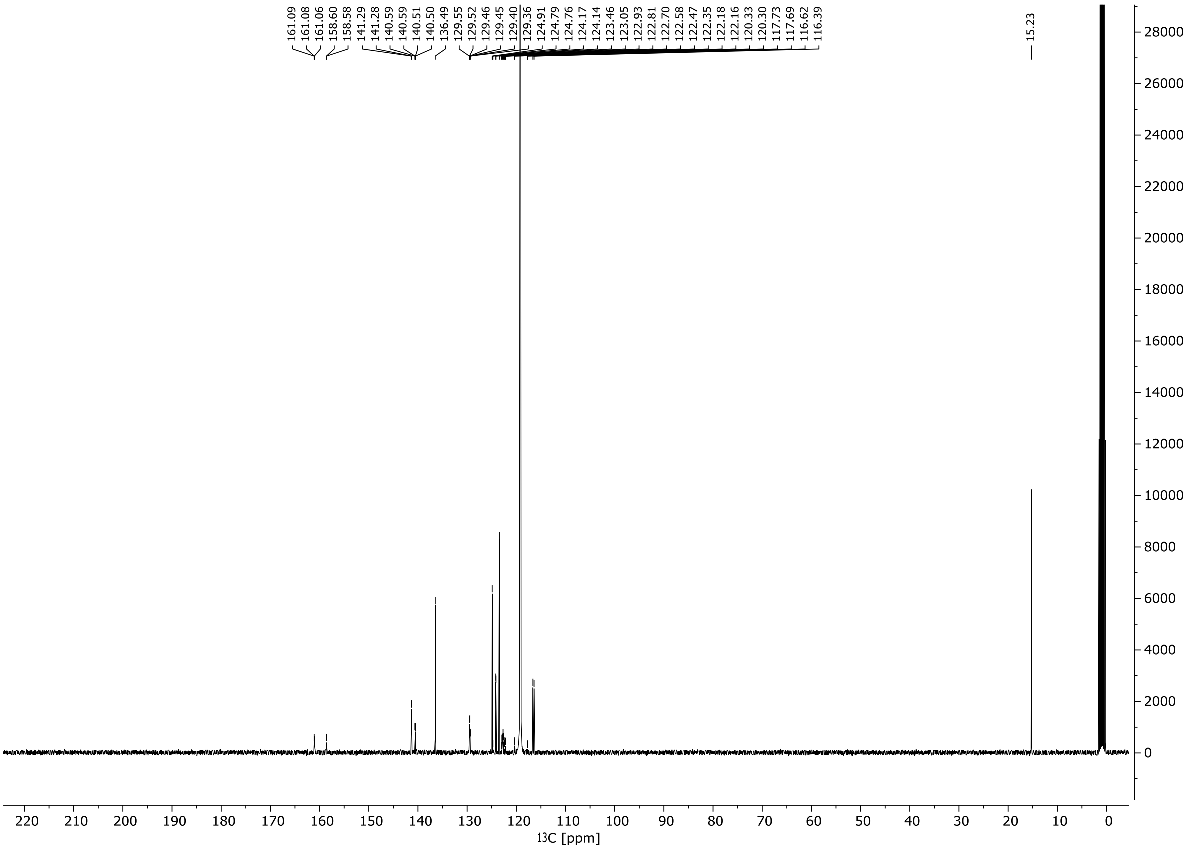

^{1}H NMR of compound **9**

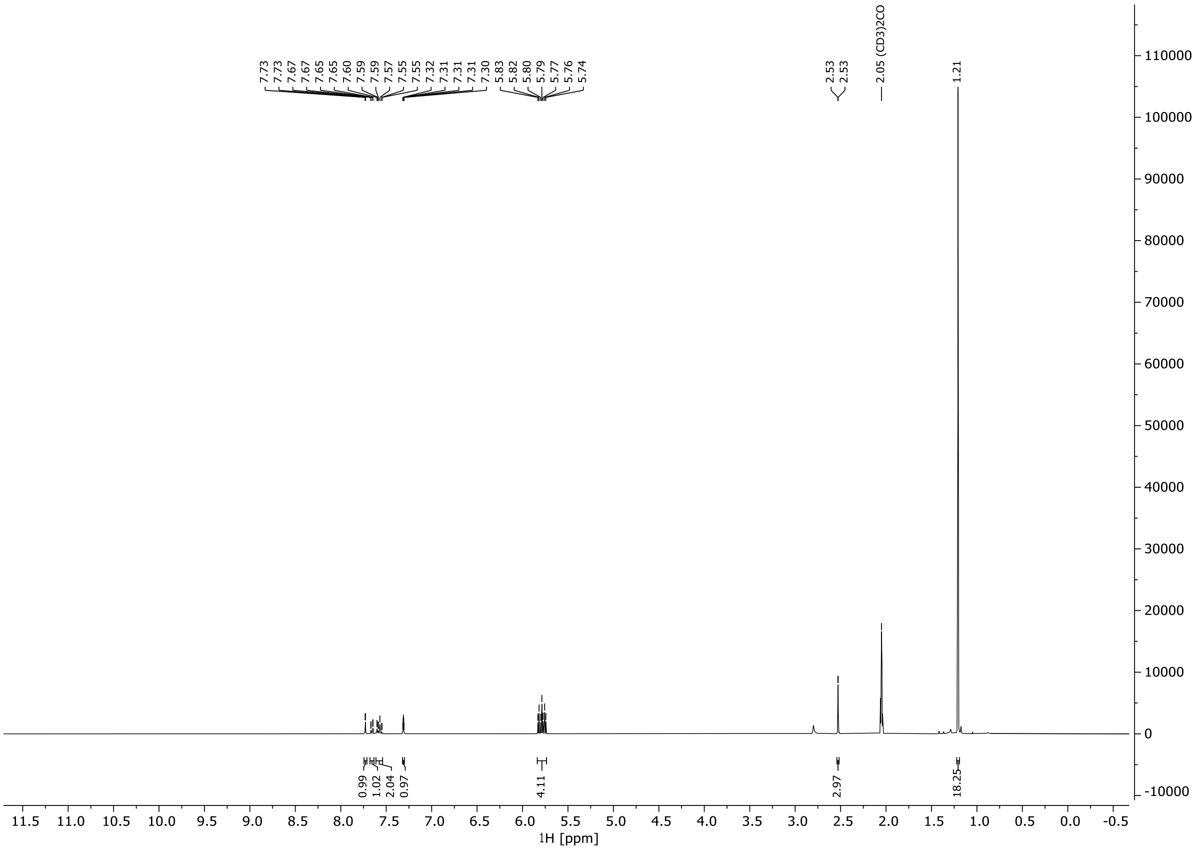

^{19}F NMR of compound **9**

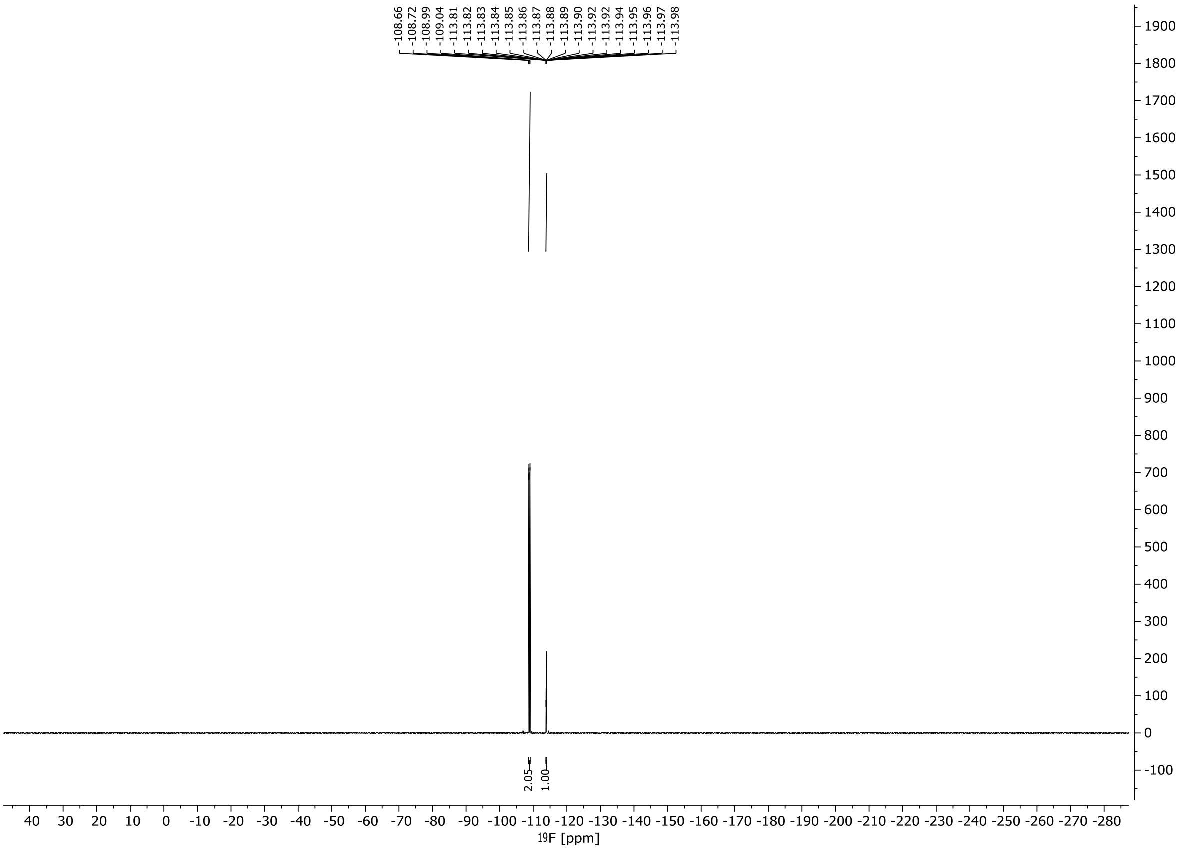

^{31}P NMR of compound **9**

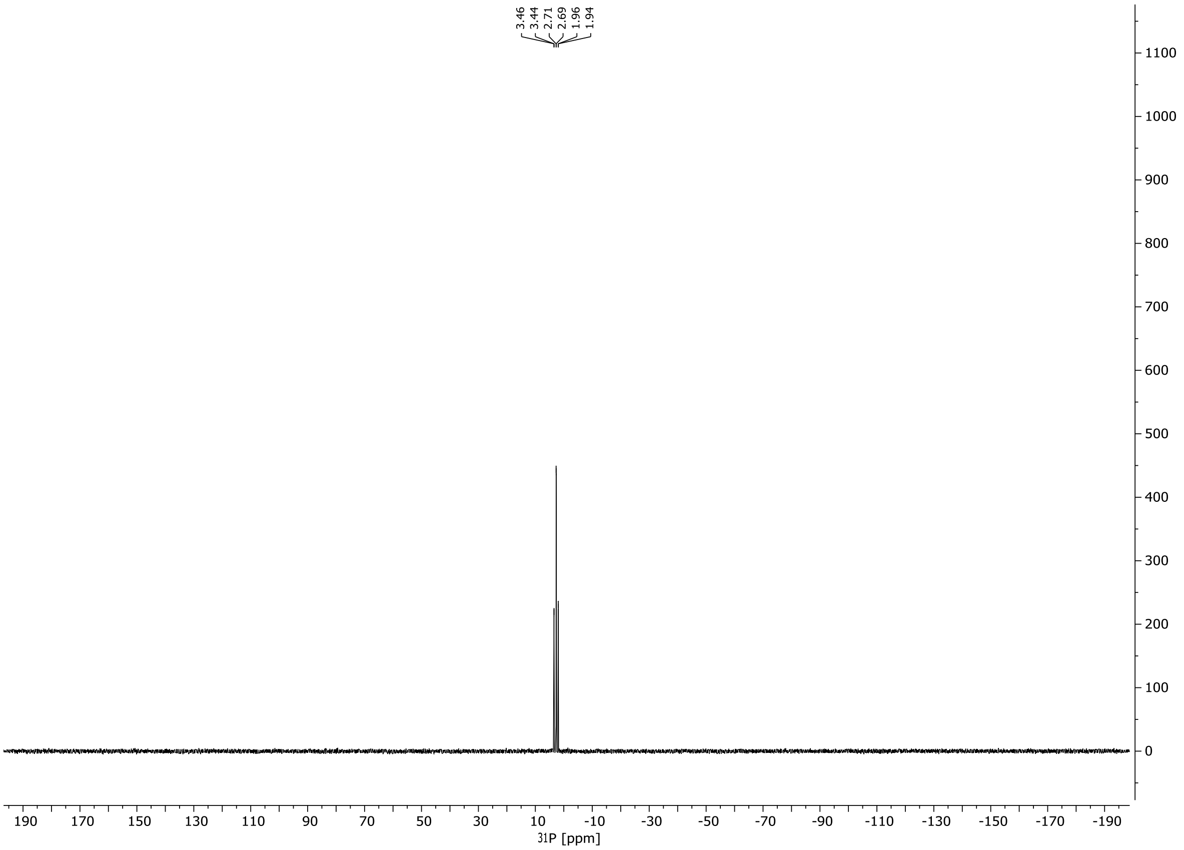

^{13}C NMR of compound **9**

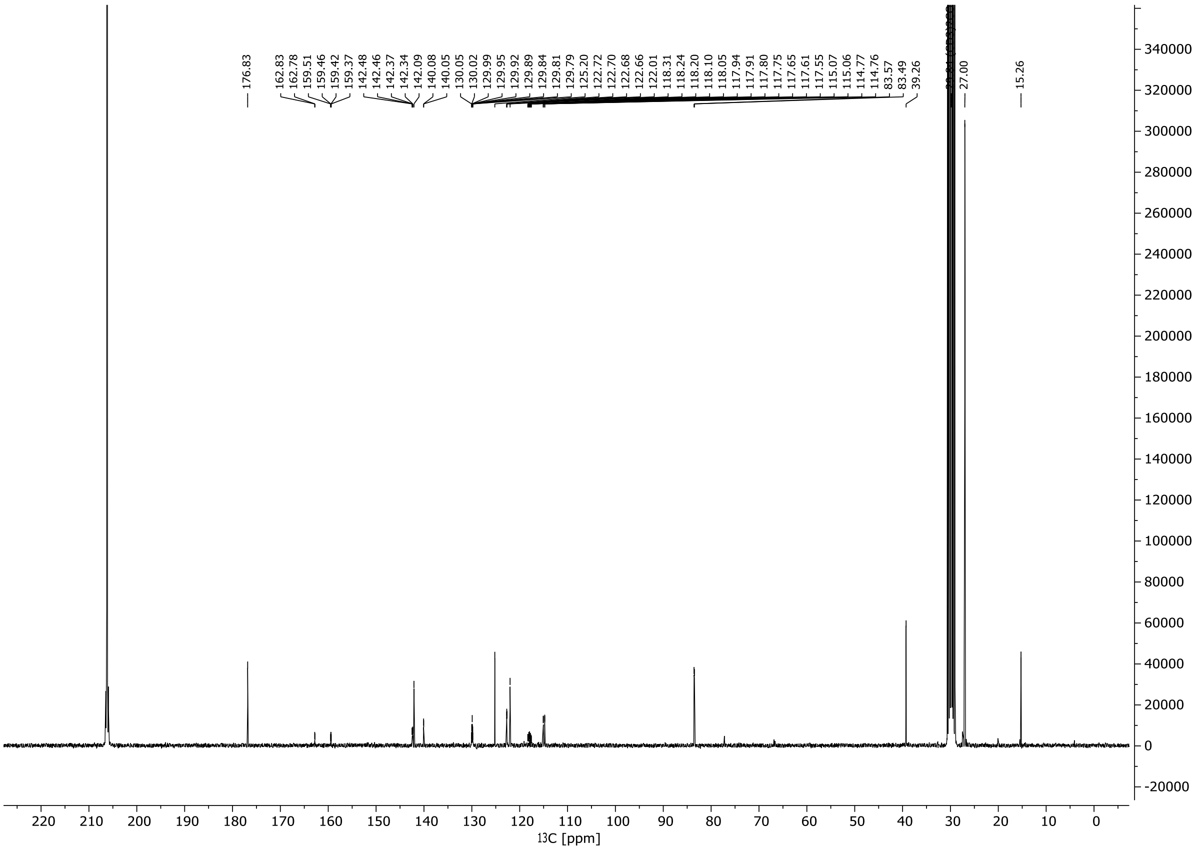

^{1}H NMR of compound **10**

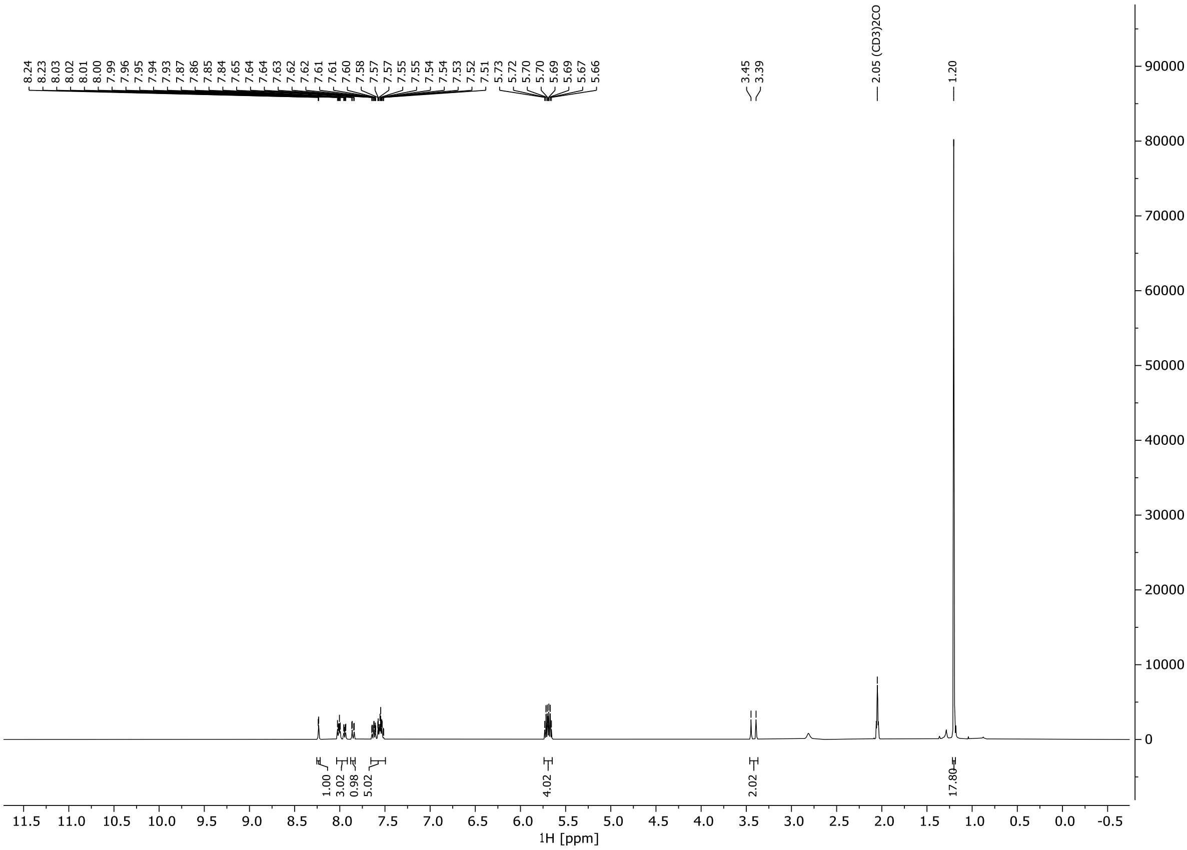

^{19}F NMR of compound **10**

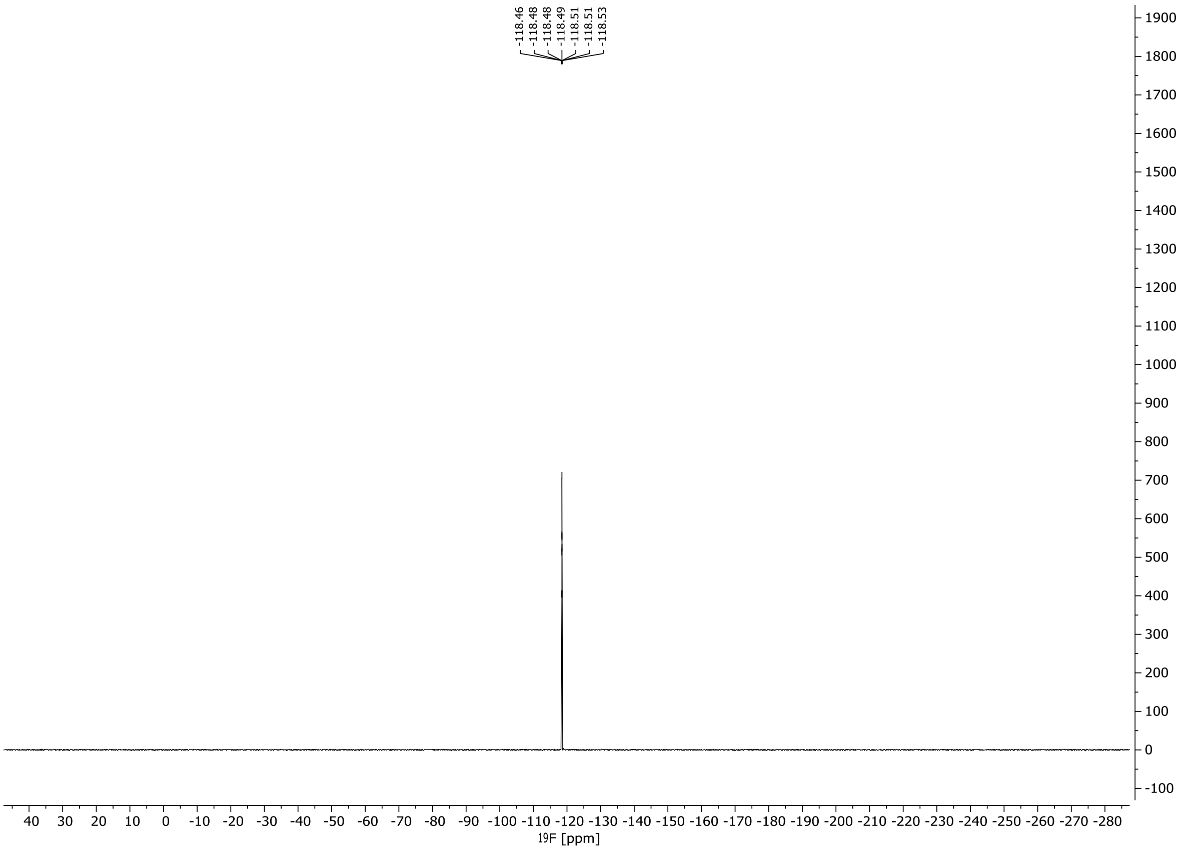

^{31}P NMR of compound **10**

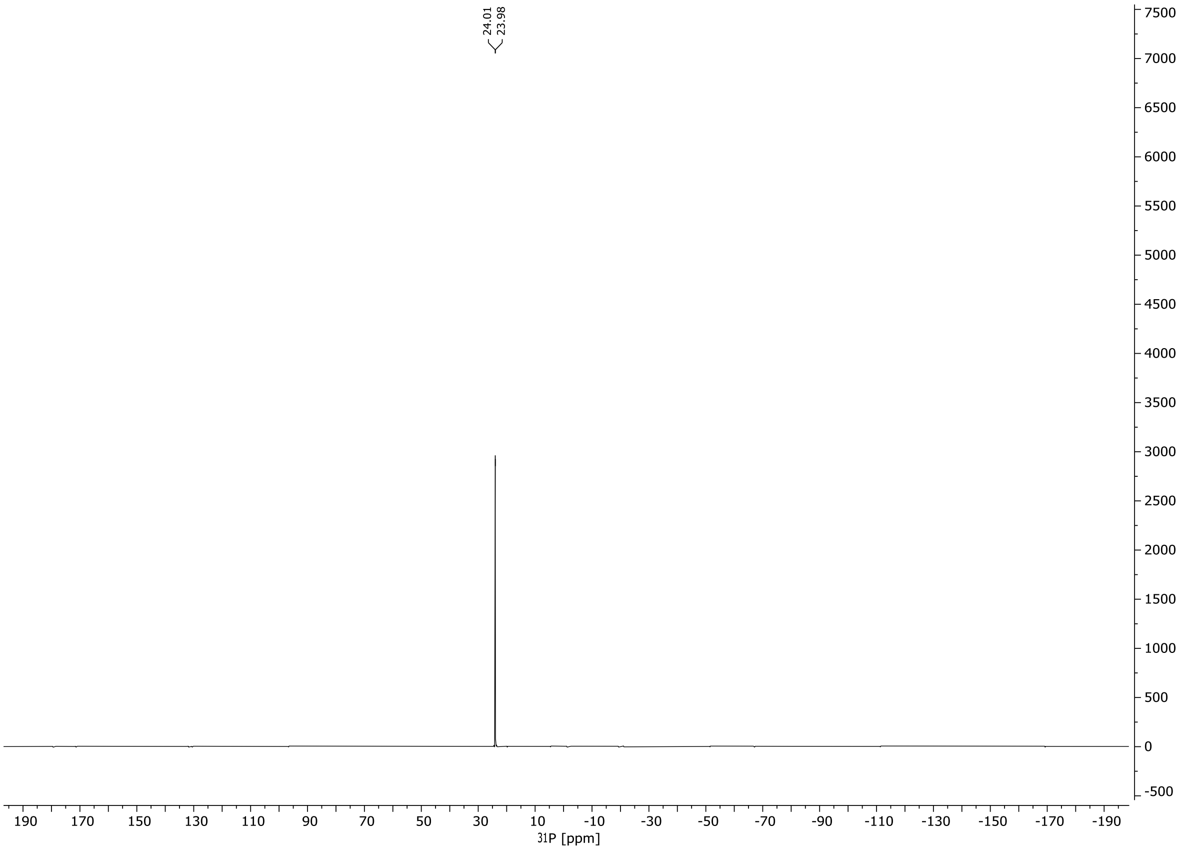

^{13}C NMR of compound **10**

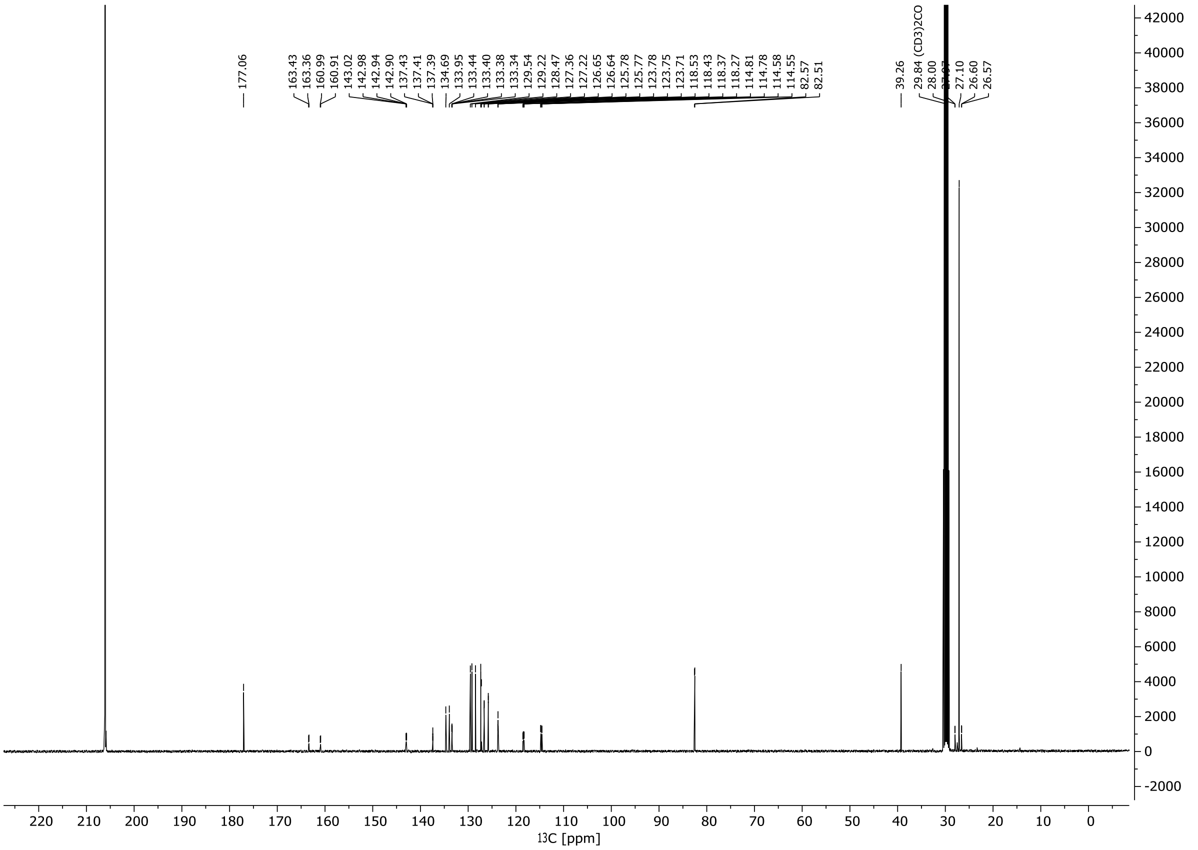

Supporting References

[1] S. Keller, C. Vargas, H. Zhao, G. Piszczek, C. A. Brautigam, P. Schuck, *Anal. Chem.* **2012**, *84*, 5066-5073.

[2] J. Gräb, A. Berg, L. Blechschmidt, B. Klüver, S. Rubner, D. Y. Fu, J. Meiler, M. Gräber, T. Berg, *ACS Chem. Biol.* **2019**, *14*, 796-805.

[3] Z. Nikolovska-Coleska, R. Wang, X. Fang, H. Pan, Y. Tomita, P. Li, P. P. Roller, K. Krajewski, N. G. Saito, J. A. Stuckey, S. Wang, *Anal. Biochem.* **2004**, *332*, 261-273.

[4] A. L. Hopkins, G. M. Keseru, P. D. Leeson, D. C. Rees, C. H. Reynolds, *Nat. Rev. Drug Discov.* **2014**, *13*, 105-121.

[5] T. H. Scheuermann, C. A. Brautigam, *Methods* **2015**, *76*, 87-98.

[6] J. C. D. Houtman, P. H. Brown, B. Bowden, H. Yamaguchi, E. Appella, L. E. Samelson, P. Schuck, *Protein Sci.* **2007**, *16*, 30-42.

[7] C. A. Brautigam, in *Methods Enzymol., Vol. 562* (Ed.: J. L. Cole), Academic Press, **2015**, pp. 109-133.

[8] R. Jafari, H. Almqvist, H. Axelsson, M. Ignatushchenko, T. Lundback, P. Nordlund, D. Martinez Molina, *Nat. Protoc.* **2014**, *9*, 2100-2122.

[9] C. A. Schneider, W. S. Rasband, K. W. Eliceiri, *Nat. Methods* **2012**, *9*, 671-675.

[10] L. Goldschmidt, D. R. Cooper, Z. S. Derewenda, D. Eisenberg, *Protein Science* **2007**, *16*, 1569-1576.

[11] G. Estenne, P. Dodey, P. Renaut, G. Leclerc, *Org. Prep. Proced. Int.* **1995**, *27*, 500-503.

[12] R. J. Edsall, H. A. Harris, E. S. Manas, R. E. Mewshaw, *Bioorg. Med. Chem.* **2003**, *11*, 3457-3474.

[13] C. Yang, R. Edsall, H. A. Harris, X. Zhang, E. S. Manas, R. E. Mewshaw, *Bioorg. Med. Chem.* **2004**, *12*, 2553-2570.

[14] R. E. Mewshaw, R. J. Edsall, C. Yang, H. A. Harris, J. C. Keith, Jr., L. M. Albert, *WO2003051805* **2003**.

[15] N. Oka, T. Yamada, H. Sajiki, S. Akai, T. Ikawa, *Org. Lett.* **2022**, *24*, 3510-3514.

[16] B. P. Bandgar, R. J. Sarangdhar, S. Viswakarma, F. A. Ahamed, *J. Med. Chem.* **2011**, *54*, 1191-1201.

[17] P. K. Mandal, F. Gao, Z. Lu, Z. Ren, R. Ramesh, J. S. Birtwistle, K. K. Kaluarachchi, X. Chen, R. C. Bast, Jr., W. S. Liao, J. S. McMurray, *J. Med. Chem.* **2011**, *54*, 3549-3563.
